# Non‐Dilute Synthesis of Macrodiolides and Macrotetrolides Enabled by Confinement Catalysis

**DOI:** 10.1002/anie.6206059

**Published:** 2026-06-21

**Authors:** Feng‐Yuan Wang, Alessandro Prescimone, Daniel Häussinger, Konrad Tiefenbacher

**Affiliations:** ^1^ Department of Chemistry University of Basel Basel Switzerland; ^2^ Department of Biosystems Science and Engineering ETH Zurich Basel Switzerland

**Keywords:** macrodiolide, macrolactonization, macrotetrolide, non‐dilute conditions, supramolecular catalysis

## Abstract

Macrodiolides are widespread motifs in functional molecules, yet their synthesis remains challenging. Existing methods typically still rely on high‐dilution conditions to suppress undesired oligomerization, limiting efficiency and scalability. Therefore, developing macrocyclization strategies under non‐dilute conditions remains a long‐standing goal. Herein, we report a confinement approach to address this challenge by utilizing the spatial restriction and conformational preorganization provided by the hexameric resorcin[4]arene capsule. Such supramolecular confinement enables the cyclization of readily accessible diols and diacyl chlorides to afford 18–32‐membered macrodiolides under non‐dilute conditions and without the need for slow‐addition procedures. Notably, the method also enables the one‐pot formation of 26–32‐membered macrotetrolides, a transformation that is difficult to achieve using conventional strategies, including high‐dilution conditions. Compared with established ring‐closing metathesis, this strategy offers higher efficiency, shorter synthetic routes, and access to more strained macrodiolides. Overall, these findings highlight the power of confinement catalysis for challenging macrocyclization reactions.

## Graphical abstract

Confinement catalysis within a hexameric resorcin[4]arene capsule enables macrolactonization under non‐dilute conditions (up to 0.1 M), overcoming the limitations of high‐dilution protocols (0.1–10 mM). This strategy provides direct access to 18–32‐membered macrodiolides and 26–32‐membered macrotetrolides in one pot and unlocks previously inaccessible strained [14.1.1.1] paddlane‐type skeleton.

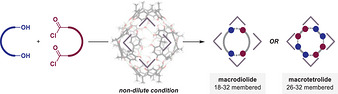

## 1 Introduction

**FIGURE 1:**
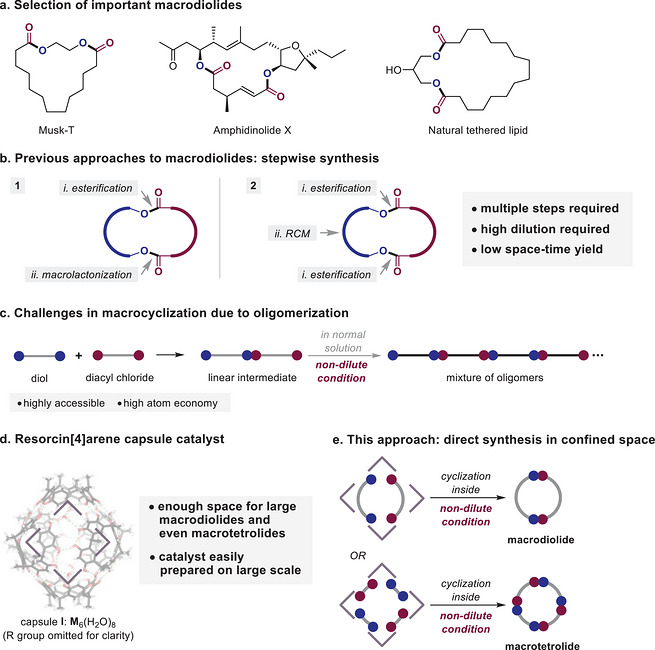
General design. (a) Selection of important macrodiolides. (b) Previous approaches to macrodiolides: stepwise synthesis. (c) Challenges in macrocyclization due to oligomerization. (d) Resorcin[4]arene capsule catalyst. (e) This approach: direct synthesis in confined space.

Macrocycles (i.e., ring structures formed by ≥12 atoms) [1] are ubiquitous and important skeletons in chemistry, biology, and materials science [2, 3, 4]. Ester‐linked macrocycles, including macrodiolides and macrotetrolides, are particularly prominent in natural products and also fragrances, which have spurred sustained interest in their synthesis (Figure 1a) [5]. Unfortunately, their synthesis remains a substantial challenge. In particular, classical macrocyclization strategies for constructing these scaffolds still heavily rely on high‐dilution conditions (typically 0.1–10 mM) [6, 7, 8], a long‐standing unresolved issue in the field. Such conditions inevitably require excessive solvent consumption, prolonged reaction times, and often impractical slow‐addition protocols. Collectively, these factors result in poor economic efficiency and low space–time yields (STY = *n*_product_ / (*V*_reaction_ × *t*_reaction_)) [9], rendering such high‐dilution macrocyclizations fundamentally unattractive for practical and sustainable synthesis.

Taking macrodiolides as an example, one of the most straightforward strategies would involve a macrolactonization between a diol and a dicarboxylic acid (or an appropriate derivative). However, to the best of our knowledge, no generally applicable macrolactonization methodology that operates under non–high‐dilution conditions has yet been developed to accomplish this transformation. In practice, alternative industrial approaches have been explored, most notably depolymerization of oligomers followed by distillative isolation of the desired product. However, this strategy is inherently limited to thermally robust and sufficiently volatile targets and is typically restricted to unsubstituted macrodiolides with ring sizes not exceeding 24 atoms [10]. On the research scale (Figure 1b), stepwise strategies such as esterification followed by macrolactonization [11, 12] or double esterification combined with ring‐closing metathesis (RCM) [13] are typically employed [14]; however, these approaches likewise depend on high‐dilution conditions. These limitations underscore the need for more efficient and versatile approaches to access functionalized macrodiolides across a broad range of ring sizes. Even more attractive would be the selective formation of macrotetrolides directly from two diols and two dicarboxylic acid derivatives in a one‐pot pseudo–four‐component reaction. However, such a process is intuitively even more challenging, as high‐dilution conditions would be counterproductive.

The key challenge in developing such direct macrolactonization reactions under non‐dilute conditions lies in the effective preorganization of the linear precursors, a factor widely considered crucial for successful macrocyclization [15, 16]. In other words, insufficient preorganization under non‐dilute conditions typically leads to undesired oligomerization (Figure 1c). While nanoconfinement has been proposed as a potential solution [17] and some successes for other reaction types have been reported [18, 19, 20, 21, 22], supramolecular catalysis for macrolactonization remains largely unexplored. A few pioneering stoichiometric studies have explored the formation of macrolactams [23] and macrodilactams [24] within cavitands. However, these processes require excess cavitand and still operate under high‐dilution conditions (1 mM). Just very recently, our group reported [25] a catalytic macroglycosylation inside the supramolecular capsule **I** (Figure 1d), formed through self‐assembly of resorcin[4]arene and water in various apolar solvents [26, 27, 28, 29]. However, the reported glycosylation is not a typical reaction for macrocyclization, unlike lactonization. Moreover, the development of macroglycosylation benefited greatly from our previous finding that glycosylation is significantly accelerated within supramolecular capsule **I** [30, 31, 32]. In stark contrast, esterification has not been demonstrated to be accelerated inside this capsule. Consequently, the first challenge for lactonization lies in the lack of precedent for esterification in such a confined environment as acceleration within the capsule is essential to outcompete oligomerization in bulk solution. From a practical and atom‐economy perspective, a direct reaction between a diol and a diacyl chloride, both commercially available or readily accessible in most cases, provides an ideal starting point to explore this concept. Supramolecular capsule **I**, has been shown to bind and activate halides within its confined space. This capability enables a variety of transformations, including terpene cyclization with HCl as a co‐catalyst [33, 34, 35, 36, 37, 38, 39], β‐selective glycosylation involving glycosyl halides [30, 31, 32], diverse C─F bond transformations [40], Friedel–Crafts reactions involving benzyl [41] or benzoyl chlorides [42], and carbocation catalysis through activation of trityl chloride [43]. Based on these precedents, we envisioned that activation of acid chlorides within the capsule could be feasible, thereby enabling the desired macrocyclization reaction. Notably, the large internal volume of **I** (approximately 1400 Å^3^) [26], which exceeds that of most other commonly employed supramolecular capsules, is expected to accommodate substrates of varying sizes. Moreover, capsule **I** is commercially available and can be prepared on a multidecagram scale without column chromatography, rendering it highly practical for synthetic applications.

Here, we report the successful realization of this macrolactonization approach, delivering macrodiolides, and even macrotetrolides, under standard, non‐dilute concentrations (Figure 1e).

## 2 Results and Discussion

### 2.1 Reaction Development

We began exploring our concept by testing the reaction between 1,3‐propanediol (**1a**) and octadecanedioyl dichloride (**2a**), which was expected to form the 23‐membered macrodiolide **3a**. Intensive optimization of the reaction conditions (see SI, Section S3 for details) led to the following reaction conditions: diol (**1a**, 1.0 equiv, 50 mM), freshly prepared diacyl chloride (**2a**, 2.5 equiv), capsule **I** (30 mol %), 9–10 equiv H_2_O/capsule, Rb_2_CO_3_ to quench the HCl formed, in PhCl, 70 °C, 1.5 h. To the best of our knowledge, no general methodology exists for macrodiolide synthesis at such concentrations to date. Because water is required for the formation of capsule **I**, which inevitably leads to hydrolysis of the acyl chloride under the reaction conditions, the diol (**1a**) was chosen as the limiting reagent. Interestingly, a small amount of byproduct **3b**, featuring a larger 27‐membered macrocycle, was also observed. The formation of **3b** requires an etherification process and may be facilitated by unquenched HCl within the system. Notably, in contrast to most established macrolactonization protocols, no slow‐addition procedure was required, enabling a convenient setup and short reaction time. Under these conditions, the desired product **3a** was isolated in 57% yield (Table 1, entry 1).

Several control experiments were conducted to learn more about the role of the supramolecular catalyst. First, in the absence of capsule **I** (entry 2), the yield of **3a** dropped dramatically (10% vs. 67%), highlighting the crucial role of the supramolecular container. In contrast, omitting the base Rb_2_CO_3_ (entry 3) still allowed efficient macrocyclization, albeit in slightly reduced yield (57% vs. 67%). These results indicate that the formation of **3a** was primarily promoted by capsule **I** rather than by Rb_2_CO_3_. These findings are consistent with extensive literature reports showing that synthetically meaningful yields of macrolactonization reactions under basic conditions are typically only achieved at high‐dilution [6, 7, 8]. Nevertheless, Rb_2_CO_3_ slightly improved the selectivity for **3a** over **3b**, in contrast to the weaker basic scavenger Al_2_O_3_ (entry 4). Next, blocking the capsule with the strongly binding ammonium guest *^n^* Bu_4_N^+^Br^−^ (TBAB) [44, 45, 46] led to background reactions outside the capsule (entry 5), affording only trace amounts of **3a**, similar to the reaction without capsule **I** (entry 2). Replacing the capsule with an equivalent amount of subunit 4,6‐diethylbenzene‐1,3‐diol gave the same result, confirming that the intact capsule structure is essential for efficient macrolactonization (entry 6). Likewise, disassembly of capsule **I** by addition of the polar solvent ethyl acetate (EA) resulted in no detectable macrodiolide products (entry 7). Finally, employing the Lewis acid ZnCl_2_, which contains the same chloride counter‐anion as the electrophile, also failed to afford either **3a** or **3b** (entry 8). Overall, these results indicate that confinement within capsule **I** is critical for achieving good macrodiolide yields.

### 2.2 Substrate Scope

**SCHEME 1:**
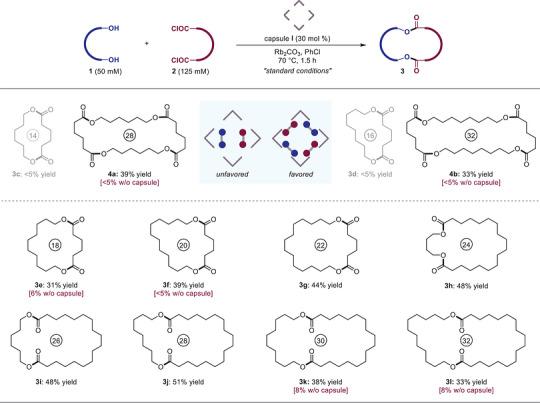
Initial exploration of substrate scope by varying ring size. Yields of isolated products were reported. Control experiments (w/o capsule) for **4a**–**b**, **3e**–**f**, and **3k**–**l** were performed under the same conditions following the same procedure.

With the optimal conditions established, we explored the formation of various macrodiolides by combining different diols and diacyl chlorides. We first aimed to define the general scope with respect to ring size, examining a range of 14–32‐membered rings centered on the 23‐membered model substrate, with two‐carbon intervals to efficiently survey multiple examples. Encouragingly, despite the pronounced size effects often observed in supramolecular catalysis [47], this method proved effective across a relatively broad range of 20–30‐membered rings (Scheme 1). Yields gradually improved as ring size increased from 18‐ to 22‐membered with the same diacyl chloride (**3e**–**g**), whereas they declined markedly from 28‐ to 32‐membered (**3j**–**l**). To our knowledge, such broad performance from a single confinement catalyst is uncommon, especially compared with known enzyme‐catalyzed macrodiolide syntheses [48]. However, as expected for a closed cavity of finite volume, substrates outside the optimal size range gave lower yields: smaller rings (< 20‐membered) likely suffer from insufficient preorganization, while larger rings (> 30‐membered) may not be encapsulated efficiently. This behavior reflects the intrinsic constraints of supramolecular confinement in this methodology. Interestingly, although 14‐ and 16‐membered macrodiolides were not formed in significant amounts, their corresponding macrotetrolides were successfully produced through the formation of four ester bonds in a single reaction (**4a** and **4b**, respectively). Established methods for macrotetrolide synthesis typically require stepwise ester formation (i. mono‐activation of diol; ii. first esterification via acylation of alcohol; iii. second esterification via nucleophilic substitution) and suffer from extremely low synthetic efficiency [49]. Even tin‐template strategies for oxygen‐rich substrates often yield unselective mixtures of cyclic oligomers [50]. Given that one‐step cyclooligomerization to form macrocycles has long been considered a formidable challenge [51], these results offer a promising new strategy for efficient and selective macrotetrolide synthesis. Finally, for macrodiolides and macrotetrolides formed in < 40% yield, control experiments without the capsule were performed but only produced trace amounts (≤ 8%), underscoring the essential role of confinement catalysis in these transformations.

**SCHEME 2:**
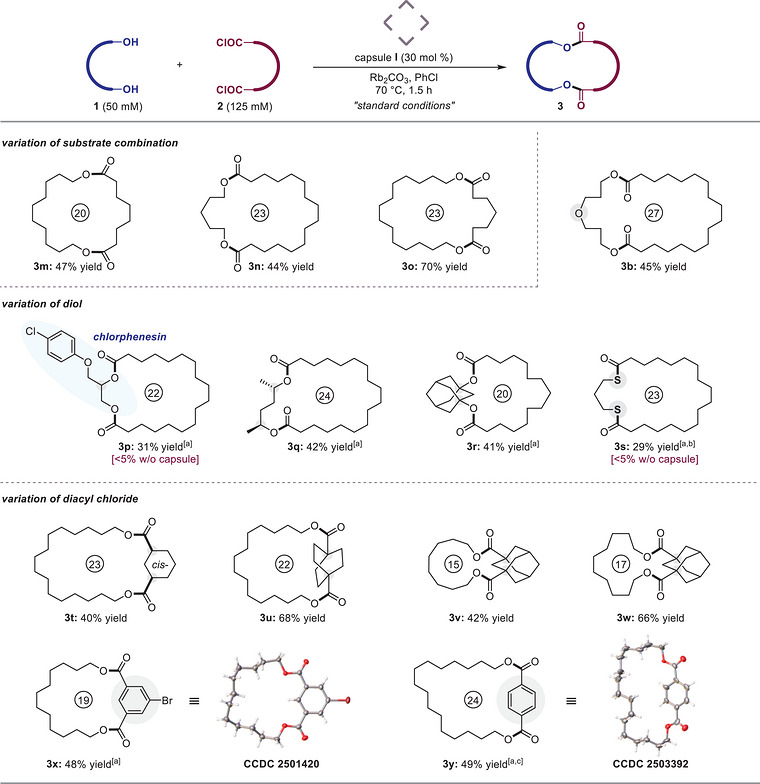
Further exploration of substrate scope. ^[a]^ 90 °C instead of 70 °C. ^[b]^ 3 h instead of 1.5 h. ^[c]^ Diacyl chloride from commercial source applied. The ellipsoids were plotted at 50% probability level in XRD structures. Some parts of alkyl chain in **3y** are orientationally disordered over two positions (50%/50% occupancy) and one of them was shown for clarity (see Supporting Information, Appendix A for details). Yields of isolated products were reported. Control experiments (w/o capsule) for **3p** and **3s** were performed under the same conditions by following the same procedure.

Encouraged by these initial results, we next explored additional substrates to demonstrate the full potential of this method (Scheme 2). First, we showed that, for a given ring size, different combinations of diols and diacyl chlorides are feasible (e.g., **3f** and **3m** for 20‐membered rings; **3a**, **3n**, and **3o** for 23‐membered rings). Next, we explored the scope of diols. Beyond simple carbon‐chain linkages, ether linkages in the diol component were well tolerated (**3b**, using 3,3′‐oxybis(propan‐1‐ol)). In addition, sterically hindered alcohols were compatible: secondary alcohols (**3p**–**q**) and even tertiary alcohols (**3r**), which are regarded as more challenging substrates [52, 53], underwent macrocyclization at 90 °C. While phenols failed to produce the desired products (see SI, Section S6 for details), thiols were tolerated, affording macrocyclic dithioester **3s** at 90 °C for 3 h, a scaffold that has been rarely explored [54, 55]. Finally, we explored the scope of the diacyl chloride. Steric hindrance α to the carbonyl is well tolerated, including secondary (**3t**) and tertiary centers (**3u**–**w**). Notably, the additional bulk of substituted diacyl chlorides enabled efficient formation of rings (15‐ and 17‐membered for **3v**–**w**) that lie outside the optimal range for unsubstituted substrates (Scheme 1) as the substituents occupy the empty space within capsule **I** and promote effective preorganization. While we primarily investigated aliphatic diacyl chlorides, aromatic examples were also effective, yielding **3x** and **3y** (both confirmed by x‐ray diffraction (XRD)) with only a modest increase in reaction temperature (90 °C). Notably, literature methods for synthesizing the same **3y** skeleton by reacting the diol and diacyl chloride under DABCO (1,4‐diazabicyclo[2.2.2]octane)–Et_3_N conditions afforded only 22% yield [56], even when employing slow‐addition to achieve high‐dilution conditions, as compared to 49% yield using this methodology. Remarkably, even products formed in lower yields (29%–40%) were readily isolable, as no other macrocycles were produced in significant amounts; the remaining material consisted of oligomers likely generated from background reactions outside the capsule and could be easily separated (see also Table 1, entry 2: full conversion of diol after 1.5 h, primarily oligomers). This stands in positive contrast to previous reports, where mixtures of macrocycles were formed, leading to complicated purification [51].

**TABLE 1:** Reaction development.^a^

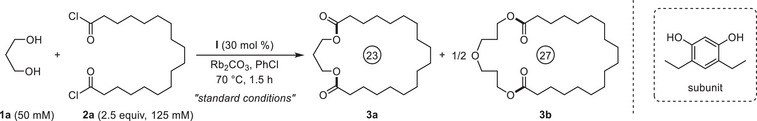			
Entry	Variations of reaction conditions	Yield of 3a^b^	Selectivity 3a/3b^b^
1	None	67 (57)	67/6
2	No capsule **I**	10 (6)	10/0
3	No Rb_2_CO_3_	57 (42)	57/10
4	Basic Al_2_O_3_ instead of Rb_2_CO_3_	51	51/10
5	2 equiv *^n^* Bu_4_N^+^Br^−^ (TBAB) added	9 (4)	9/0
6	720 mol % subunit instead of **I**	9	9/0
7	10 equiv ethyl acetate (EA) added	0	0/0
8	3 equiv ZnCl_2_ instead of **I** and Rb_2_CO_3_	0	0/0

^a^ Standard conditions: diol (**1a**, 0.10 mmol, 1.0 equiv), diacyl chloride (**2a**, 0.25 mmol, 2.5 equiv, freshly prepared), C_11_‐resorcin[4]arene capsule (0.030 mmol capsule **I**, 30 mol %), finely powdered Rb_2_CO_3_ (60 mg) as the base, dry PhCl as the solvent (2.0 mL in total, 1.5 mL for dissolving **1a** and C_11_‐resorcin[4]arene, 0.5 mL for dissolving **2a**), 70 °C, 5 mL Schlenk tube, Ar atmosphere, 1.5 h.

^b^ Yields and ratios were determined by GC using *n‐*pentadecane as the internal standard and response factors calculated according to literature [57]. Isolated yields of **3a** are given in parentheses. Full conversion of **1a** was confirmed by ^1^H NMR analysis for all entries.

### 2.3 Comparison With Prior Art

**SCHEME 3:**
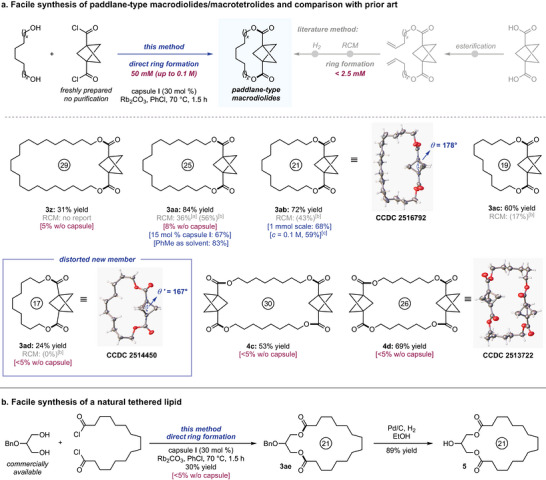
Synthetic application and comparison with prior art. (a) Facile synthesis of paddlane‐type macrodiolides/macrotetrolides and comparison with prior art. (b) Facile synthesis of a natural tethered lipid. ^[a]^ The overall yield (3 steps) [64]. ^[b]^ The yields of ring formation step (RCM) shown in parentheses [64]. ^[c]^ *c* = 0.1 M for tetradecane‐1,14‐diol and *c* = 0.25 M for bicyclo[1.1.1]pentane‐1,3‐dicarbonyl dichloride. Yields of isolated products under standard conditions were reported unless otherwise stated. Control experiments (w/o capsule) for **3z**–**aa**, **3ad**–**ae**, and **4c**–**d** were performed under the same conditions by following the same procedure. The BCP units in **4d** are orientationally disordered over two positions (85%/15% occupancy) and the major one was shown for clarity (see Supporting Information, Appendix A for details).

After having established the scope and limitations, we sought to compare this methodology to alternative established routes to macrodiolides. We selected paddlane‐type skeletons, featuring two bridgehead carbons linked by four bridges ([m.n.o.p]), as test cases [58]. These intriguing structures created a platform for both theoretical [59, 60, 61] and synthetic [62, 63, 64] studies, and small [m.1.1.1] paddlanes are notoriously difficult to synthesize [61], with very few successful examples reported. Paquette and co‐workers accessed oxa[m.1.1.1] paddlanes via RCM [64], with a minimum *m* = 16 (19‐membered ring, Scheme 3a). These structures are isosteres of the widely studied *para*‐cyclophane skeleton [65, 66]. However, the shorter ring span (1.7 Å vs. 2.8 Å) [67] and the rigidity of bicyclo[1.1.1]pentane (BCP) [61] make paddlane ring formation increasingly unfavorable as m decreases.

To our delight, this methodology enabled direct one‐step access to this substance class, replacing the conventional three‐step esterification/RCM/hydrogenation sequence and providing both a shorter and higher‐yielding route for *m* = 22 (84%, 1 step vs. 36%, 3 steps for **3aa**). Even considering only the ring‐formation step, superior efficiency was observed for both *m* = 22 (84% vs. 56% for **3aa** skeleton) and *m* = 18 (72% vs. 43% for **3ab** skeleton). It is noteworthy that the reaction of **3aa** still delivered a satisfactory yield (67%) with a reduced loading of capsule **I** (15 mol %), indicating that a higher turnover number (TON) than that observed under the standard conditions is achievable. For *m* = 16, where RCM is ineffective despite the high‐dilution conditions employed (< 20% yield), we still obtained **3ac** in 60% yield. Beyond step count, concentration advantage (50 mM vs. 2.5 mM), and yield advantages, this method demonstrates considerable practicality: a 1 mmol scale reaction of **3ab** gave a yield comparable to the smaller‐scale reaction, and the capsule could be recovered via normal column chromatography with a typical 75% recovery (see SI, Section S5). Moreover, the system produced **3ab** in 59% yield under even higher concentrations (*c* = 0.1 M for the diol and 0.25 M for the diacyl chloride), which is remarkable given the limitations of established macrolactonization methods [68, 69, 70].

Encouraged by these results, we next targeted an even more challenging skeleton with *m* = 14, for which RCM strategies fail (0% yield) and only a mixture of larger cyclic oligomers is observed, even when using 30 mol % first‐generation Grubbs catalyst and slow addition at *c* < 2.5 mM. Remarkably, this confinement strategy enables the formation of this highly strained scaffold for the first time, overcoming the size limitation of the RCM strategy and representing the smallest validated [m.1.1.1] paddlane‐type skeleton to date [71]. Although the yield of **3ad** leaves room for improvement, it forms as the sole macrocycle without any detectable macrotetrolide. The crystal structure of **3ad** unambiguously demonstrates that the BCP unit undergoes substantial distortion from its ideal *C*_3_ axis (*θ’* = 167° in **3ad** instead of *θ =* 178° in larger homologue **3ab**), providing a conclusive experimental confirmation of a long‐standing theoretical prediction [61, 62]. This work therefore offers an inspiring strategy for both the synthesis and structural investigation of strained polycyclic molecules. It also rationalizes why shorter diols lead exclusively to macrotetrolides (**4c**–**d**), avoiding further distortion and precluding even trace formation of macrodiolides. As discussed above, for products obtained in < 40% yield, control experiments without the capsule were performed. None of these products could be obtained in acceptable yield (> 8% yield) in the absence of the capsule. An additional control experiment for the highest‐yielding example (**3aa**, 84%) also gave a similarly poor yield (8%), confirming that the excellent performance observed is not due to the inherent reactivity of this particular substrate pair.

Overall, this confinement strategy outperforms conventional RCM approaches by reducing synthetic steps to a minimum, improving yields, and enabling access to smaller rings. Importantly, while BCP is a valuable phenyl bioisostere in drug design [72], its incorporation into macrocycles remains largely unexplored. The successful use of toluene, a more process‐friendly solvent [73] than chlorobenzene, with comparable yields (83% for **3aa**) further demonstrates the practicality of this method for synthesizing BCP‐embedded macrocycles for future studies.

### 2.4 Application in Natural Product Synthesis

The above findings highlight the generality of this method and suggest its potential utility in natural product synthesis. Notably, 3‐hydroxy‐1,5‐dioxacyclohenicosane‐6,21‐dione (**5**), a tethered lipid from *Thapsia garganica*, can be directly accessed from commercially available 2‐(benzyloxy)propane‐1,3‐diol and hexadecanedioyl dichloride via a direct 21‐membered ring formation followed by deprotection (Scheme 3b). This approach avoids the conventional three‐step sequence of esterification, RCM, and hydrogenation typically required for constructing similar skeletons [74].

### 2.5 Mechanistic Investigation

After establishing the synthetic utility of this method, we performed additional experiments to gain some mechanistic insight.

First, we performed DOSY measurements to evaluate the structural integrity of capsule **I** under reaction‐relevant conditions (70 °C, toluene‐*d_8_*) [75, 76]. The observation of a single diffusing species with a diffusion coefficient (*D* = 8.80 × 10^−10^ m^2^ s^−1^), which is comparable to that of the more stable [46] C_11_‐pyrogallol[4]arene analogue (*D* = 9.61 × 10^−10^ m^2^ s^−1^), but clearly distinct from that of non‐assembling *C*_4_‐symmetric C_11_‐tetramethoxyresorcin[4]arene [77] (*D* = 13.6 × 10^−10^ m^2^ s^−1^), strongly supports that capsule **I** remains assembled at 70 °C (see SI, Section 8).

**SCHEME 4:**
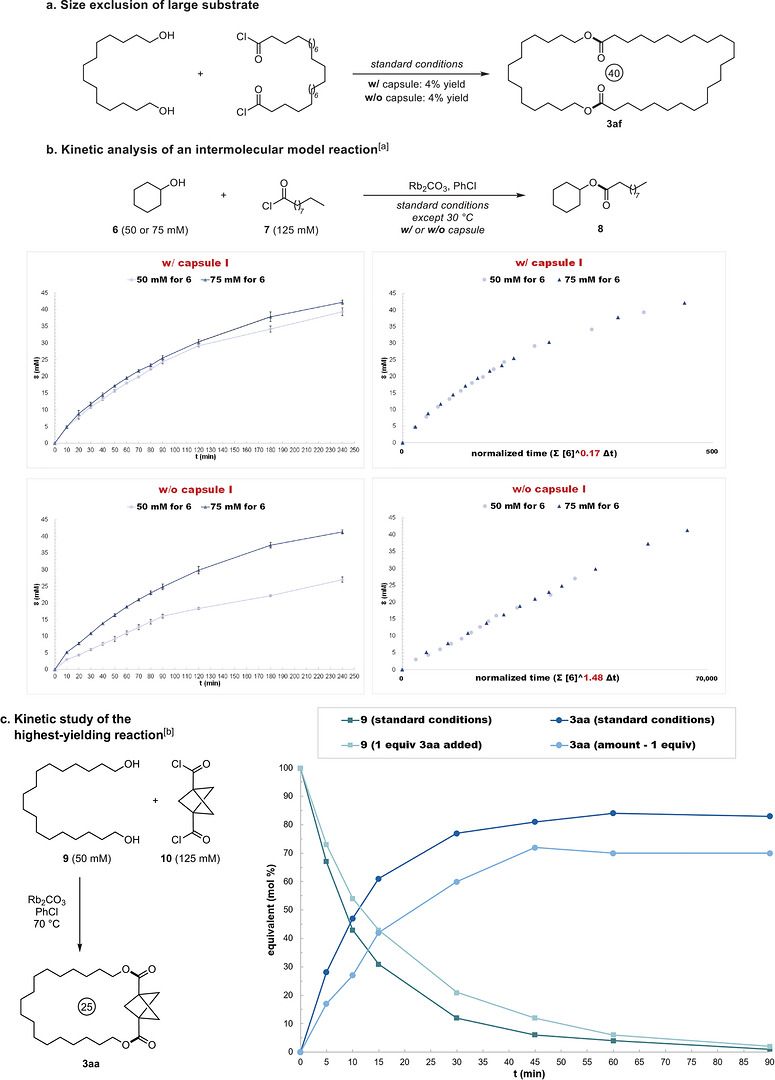
Mechanistic investigation. (a) Size exclusion of large substrate. (b) Kinetic analysis of an intermolecular model reaction. (c) Kinetic study of the highest‐yielding reaction. [a] Equivalents of **6**, **7**, and **8** were determined by ^1^H NMR (acetone‐*d_6_*) using tetraethylsilane (SiEt_4_) as the internal standard. For kinetic experiments with capsule **I**, 10 mol % was applied. [b] Equivalents of **9** and **10** were determined by ^1^H NMR (acetone‐*d_6_*) using tetraethylsilane (SiEt_4_) as the internal standard, and the equivalent of **3aa** was determined by GC using *n‐*pentadecane as the internal standard and response factors calculated according to literature [57].

Next, we investigated substantially larger substrates that would result in a 40‐membered ring (**3af**) (Scheme 4a). The yield obtained was very low (4%) and, importantly, was unaffected by the presence of the capsule, indicating that the product forms outside of the capsule. These results, combined with the size‐dependence observed in Scheme 1, are consistent with our hypothesis that the increased macrocyclization yields observed for suitably sized substrates originate from reactions occurring inside the capsule.

Furthermore, we conducted kinetic studies to assess the rate acceleration imparted by capsule **I**. For simplicity, we first monitored the intermolecular reaction between cyclohexanol (**6**) and decanoyl chloride (**7**) as a model system (Scheme 4b). While both reaction conditions (with and without capsule **I**) were found able to generate ester **8** in approximately 80% NMR yields giving enough reaction time (24 h), analysis of the reaction profiles during first 4 h indicated that capsule **I** does accelerate the esterification, although the enhancement was modest: 2.0‐fold with 10 mol % capsule **I** based on initial rates (see SI, Section 9). The capsule **I** also significantly accelerated the hydrolysis of acyl chloride **7** to the corresponding carboxylic acid as detected by crude ^1^H NMR. Thus, an excess of acyl chloride (2.5 equiv) was necessary to achieve high macrocycle yields. Although the rate acceleration induced by capsule **I** was not particularly pronounced, it revealed an intriguing kinetic phenomenon. When we examined the reaction order with respect to cyclohexanol (**6**) by increasing its concentration from the standard 50 mM to 75 mM and determining the order using the variable time normalization analysis (VTNA) [78], the presence of the capsule catalyst resulted in an approximately zero‐order dependence on **6** (measured order: 0.17). In contrast, a near‐1.5 order dependence was observed in the absence of capsule **I** (measured order: 1.48). This result suggests that within this concentration range, substrate **6** already saturates the capsule catalyst [30]. The approximately zero‐order dependence on **6** suggests that the reaction predominantly occurs within the confined cavity of capsule **I**, as a background reaction in bulk solution would be expected to display a higher reaction order, consistent with supramolecular confinement catalysis.

Lastly, the actual reaction system was examined (Scheme 4c). We monitored the reaction of 1,18‐octadecanediol (**9**) with bicyclo[1.1.1]pentane‐1,3‐dicarbonyl dichloride (**10**) under standard conditions, which afforded **3aa** with minimal side reactions (see SI, S65). An excellent ratio of **3aa** yield relative to the conversion of **9** was observed, allowing the use of a simplified kinetic model featuring bifurcation from the non‐isolable monoacylated intermediate (Figure S‐34). Analysis of the initial product formation provided an effective molarity (EM = *k*_intra_/*k*_inter_) of 0.98 M (see SI, S66), which is substantially higher than typical values for macrolactonization reported in the literature (∼ 10^−2^ M) [79]. We next investigated product inhibition. Addition of 1 equiv of **3aa** under otherwise identical conditions resulted in a modest decrease in both the conversion of **9** and the formation rate of **3aa** (0.6‐fold), as well as a reduced yield of newly formed **3aa** (70% vs. 83%), indicating moderate product inhibition. Considering the relatively high binding constant of the product (*K* ∼ 7200 M^−1^, see SI, S68), compared to the starting materials (*K* ∼ 270 M^−1^ for **9** and *K* ∼ 30 M^−1^ for **10**), the observed turnover may appear unexpected. This behavior is likely attributable to a comparably strong affinity of the non‐isolable intermediate, which enables productive catalysis despite product binding.

Overall, these mechanistic observations are in agreement with the synthetic results and collectively support a capsule‐confined reaction pathway in this study.

## 3 Conclusion

In summary, this work demonstrates that supramolecular confinement within a hexameric resorcinarene capsule provides a powerful and practical solution to the long‐standing challenge of forming macrodiolides and macrotetrolides under non‐dilute conditions. By exploiting the spatial restriction and enforced conformational preorganization inherent to the capsule's internal environment, we have enabled the direct synthesis of 18–32‐membered macrodiolides and 26–32‐membered macrotetrolides at concentrations of up to 100 mM, approximately two orders of magnitude higher than those typically employed in traditional high‐dilution protocols. The methodology is characterized by its broad substrate scope, successfully accommodating sterically hindered secondary and tertiary alcohols as well as substituted diacyl chlorides. Notably, this confinement strategy outperforms established methods such as ring‐closing metathesis (RCM) by significantly reducing synthetic steps, improving overall yields, and providing access to highly strained scaffolds that were previously considered inaccessible, such as the smallest validated [m.1.1.1] paddlane‐type skeleton. The practical utility of the approach is further highlighted by the streamlined synthesis of a natural tethered lipid and the ease of catalyst recovery.

Collectively, these results underscore the potential of confinement catalysis as a broadly applicable tool in modern synthetic chemistry. By circumventing the inefficiencies of high‐dilution conditions, this work opens new avenues for the scalable synthesis of macrodiolides and macrotetrolides.

## Conflicts of Interest

The authors declare no conflicts of interest.

## Supporting information




**Supporting File 1**: anie73213‐sup‐0001‐SuppMat.pdf.

**Supporting File 2**: anie73213‐sup‐0002‐cif.zip.

## Data Availability

The data that support the findings of this study are openly available in zenodo at https://zenodo.org/records/19869042, reference number 19869042.
