# Supplementary material for: Non‐Dilute Synthesis of Macrodiolides and Macrotetrolides Enabled by Confinement Catalysis
Source: Angew Chem Int Ed Engl. 2026 Jun 21;65(34):e6206059. doi: 10.1002/anie.6206059 (PMC13480482; doi:10.1002/anie.6206059)

Non-Dilute Synthesis of Macrodiolides and Macrotetrolides Enabled by Confinement Catalysis

Feng-Yuan Wang,[1] Alessandro Prescimone,[1] Daniel Häussinger,[1] Konrad Tiefenbacher* [1,2]

[1] Department of Chemistry, University of Basel, Mattenstrasse 22, 4058 Basel, Switzerland
[2] Department of Biosystems Science and Engineering, ETH Zurich, Klingelbergstrasse 48, 4056 Basel, Switzerland

Table of Contents

1. General information 2

2. Synthesis of catalyst and substrates 3

General procedure A: synthesis of diacyl chlorides 3

3. Reaction development 3

Initial screening (0.05 mmol-scale) 3

Further optimization (0.1 mmol-scale) 7

Control experiments for the model reaction 9

4. Synthesis and characterization of macrodiolides and macrotetrolides 12

General procedure B: synthesis of macrodiolides and macrotetrolides 12

5. Details of 1 mmol-scale reaction and recovery of resorcin[4]arene 36

6. Challenging substrates 37

Size limitation 37

Limitation for phenol-type substrate 39

7. Control experiments without capsule 40

8. DOSY experiments 55

9. Kinetic studies 62

Kinetic analysis of an intermolecular model reaction 62

Kinetic study of the highest-yielding reaction 65

10. Binding studies 67

11. References 69

Appendix A: Crystallographic details 70

Appendix B: NMR spectra of products 104

1. General information

Experimental: The preparation of fresh diacyl chlorides and all capsule-catalyzed reactions were carried out in oven-dried Schlenk tubes sealed with rubber septa under an argon atmosphere unless otherwise stated. All liquids and solutions were transferred by syringes. Reactions were stirred using Teflon-coated magnetic stir bars. Elevated temperatures for capsule-catalyzed reactions were maintained using thermostat-controlled oil bath unless otherwise stated. Organic solutions were concentrated using a rotary evaporator. Analytical TLC was performed on Supelco TLC Silica gel 60 F_{254} aluminum sheets. The TLC plates were visualized by phosphomolybdic acid (PMA) stain upon heating with a heat gun. Purification of products was accomplished by flash column chromatography on silica gel (SilicaFlash, Irregular Silica Gel, P60 40-63 µm, 60 Å, from Silicycle). All NMR experiments in the synthesis sections were performed on a Bruker Avance Neo and a Bruker Avance III HD NMR spectrometer operating at 500 MHz proton frequency. The instruments were equipped with a direct observe 5-mm BBFO smart probe (500 MHz). The probe was equipped with actively shielded z-gradients (10 A). The experiments were performed at 298 K. Chemical shifts of ^{1}H NMR and ^{13}C NMR were given in ppm. The following solvent residual signals of the deuterated solvents were used as reference: $CDCl_3$, 7.26 ppm (δ ^{1}H), 77.16 ppm (δ ^{13}C); acetone-d_6, 2.05 ppm (δ ^{1}H)[1]. Coupling constants (J) are reported in Hertz (Hz). Standard abbreviations indicating multiplicity were used as follows: s (singlet), d (doublet), t (triplet), q (quartet), p (quintet), h (sextet), dd (doublet of doublets), m (multiplet) unless otherwise stated. IR spectra were measured using a Bruker Alpha-II FT-IR Alpha instrument equipped with a Platinum ATR probe. Standard abbreviations for peak description were used as follows: s (strong), m (medium), w (weak), br (broad). GC analyses were carried out on a Shimadzu GC-2010 Plus instrument equipped with an FID detector and an Rtx-5 capillary column (length = 30 m). Hydrogen was used as the carrier gas and the constant-flow mode was used (flow rate = 30 mL/min) with a split ratio of 1:20. The following temperature-program was used: 70 °C for 1.5 min, 40 °C/min to 330 °C, and 330 °C for 4 min. Analytical LCMS was performed on a Shimadzu LCMS-2020 instrument by using MeOH as the solvent. High-resolution mass spectra were obtained on a Thermo Scientific LTQ-FT Ultra via electrospray ionization (ESI) (ESI source parameters for positive polarity mode were: spray voltage, 4.0 kV; capillary temperature, 275 °C; capillary voltage, 48 V; and tube lens, –120 V). Optical rotation was measured in an Anton Paar MCP 100 Circular Polarimeter operating on the sodium D-line (589 nm) and are reported as $[\alpha]_D^T$ (concentration in g/100 mL, solvent). Sonication was performed by using a Bandelin Sonorex smart instrument.

Sources of chemicals: All diols in this work and dicarboxylic acids for preparing the corresponding diacyl chlorides are commercially available. These chemicals were typically purchased at high commercial quality (from Acros, Apollo, BLD Pharmatech, Fluorochem, TCI, Thermo Scientific) and used without further purification. If the diol is a liquid, it was dried over 4 Å molecular sieves (Sigma-Aldrich, beads, 4–8 mesh, activated in a 120 °C oven for at least 3 days) in 4 mL capped vials before using. Rubidium carbonate was purchased from Strem as finely powdered form and dried in a 120 °C oven for at least 3 days before using. Dry solvents dichloromethane (DCM), *N*,*N*-dimethylformamide (DMF), and toluene were directly purchased (99.8%, extra dry over molecular sieve, AcrosSeal, for all). Dry PhCl was prepared from normal-grade solvent (Thermo Scientific) by drying over 4 Å molecular sieves in several 20 mL capped vials until a low water content was reached (≤ 50 ppm, measured by Mettler Toledo Coulometric KF Titrator). Solvents used for column chromatography separations (*n*-hexane and ethyl acetate) were purchased from Sigma-Aldrich as HPLC-grade. Deuterium solvents were purchased from Eurisotop.

2. Synthesis of catalyst and substrates

Synthesis of C_{11}-resorcin[4]arene: synthesis of C_{11}-resorcin[4]arene and control of water content were performed as previously described[2]. A water content of 9–10 per capsule is suitable for this work.

Synthesis of diacyl chlorides: All diacyl chlorides in this work are known compounds in literature. They were freshly prepared from the corresponding dicarboxylic acids via a very common method: $(COCl)_2$ + DMF (cat.) in dry DCM (see general procedure A below). The only exception was terephthaloyl chloride (for macrodiolide **3y**), which was purchased from commercial source as a stable white solid and directly applied.

General procedure A: synthesis of diacyl chlorides

Dicarboxylic acid (1.00 mmol) was weighed into an oven-dried 15 mL Schlenk tube (l = 160 mm) charged with a stir bar (6 mm × 10 mm). The tube was sealed with a rubber septum, evacuated/backfilled with Ar for three cycles, and subsequently protected with an Ar balloon. At room temperature, dry DCM (5.0 mL) was first added and a turbid suspension was observed. $(COCl)_2$ (210 µL, 2.45 mmol, 2.45 equiv) was then added by syringe, followed by dry DMF (typically 2-3 drops). Bubbles were immediately observed and a part of undissolved dicarboxylic acid particles was stuck to the tube due to the impact force of the bubbles. Therefore, additional dry DCM was then added to rinse the internal face of the tube (4 × 1.0 mL, added per 15 min) to ensure sufficient dissolution and full conversion of the dicarboxylic acid. After stirring at room temperature overnight, a clear solution was finally observed. All volatile constituents in the reaction mixture were removed by a rotary evaporator to give the corresponding diacyl chloride, which was immediately applied without further purification. The yields of such diacyl chloride syntheses were typically quantitative.

3. Reaction development

Initial screening (0.05 mmol-scale)

Initial exploration of this reaction was performed on 0.05 mmol scale and a dehydration operation was applied[3]. Typically, C_{11}-resorcin[4]arene (30.0 µmol, corresponding to 5.00 µmol capsule **I**, 10 mol %) was added into a 2 mL vial charged with a stir bar (3 mm × 8 mm size) and dissolved with 0.50 mL $CDCl_3$ (neutralized with basic Al_2O_3 before using) or other solvents. Oven-dried 4 Å molecular sieves (1-2 balls) were then added. The mixture was stirred at room temperature overnight until a gel was formed. To this gel was added propane-1,3-diol (**1a**, 50.0 µmol, 1.00 equiv). After stirring for several seconds, a clear solution was formed. To this solution was added n-pentadecane (50.0 µmol, 1.00 equiv) as the internal standard. Then such solution was transferred into another 2 mL vial charged with a stir bar (3 mm × 8 mm size), octadecanedioyl diacyl chloride (**2a**, 60.0 µmol, 1.20 equiv), and scavenger (15 ± 1 mg). After stirring at 30 °C (maintained by a heating block) for 24 hours, an aliquot (approximately 10 µL) of the reaction mixture was diluted with 0.30 mL of n-heptane (containing 0.08 % DMSO) and placed in a -20 °C freezer for 30 min[4]. After centrifugation to remove the precipitate formed, the solution was further diluted with n-pentane to give a sample for gas chromatographic analysis. The GC yields were corrected by response factors[5].

Table S1. Optimization of scavengers

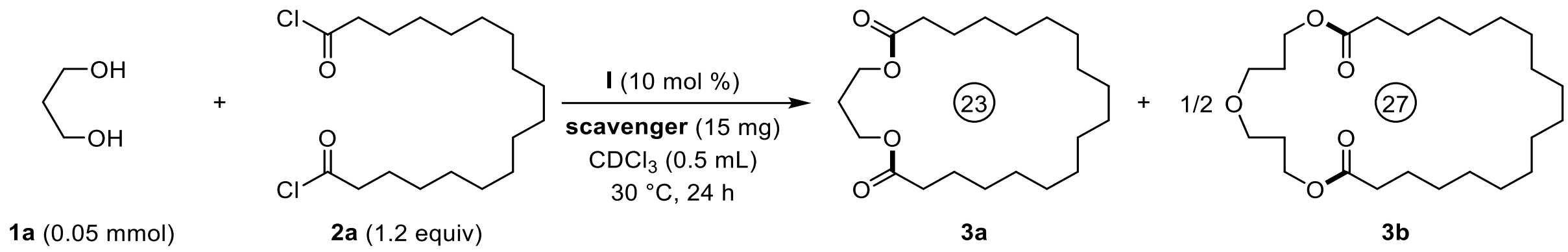

Entry	Scavenger	**3a** (%)	**3a**/**3b**
1[a]	basic Al_2O_3	12	12/4
2[a]	neutral Al_2O_3	15	15/6
3	KH_2PO_4	15	15/2
4	K_2HPO_4	16	16/6
5	K_3PO_4	18	18/2
6	Na_2HPO_4	16	16/0
7	$NaHCO_3$	17	17/0
8	$KHCO_3$	12	12/0
9	$CsHCO_3$	16	16/0
10	Li_2CO_3	13	13/0
11	Na_2CO_3	20	20/0
12	K_2CO_3	17	17/2
13	Rb_2CO_3	21	21/0
14	Cs_2CO_3	19	19/2
15	$[ZnCO_3]_2\cdot[Zn(OH)_2]_3$	6	6/0
16[b]	isobutylene oxide	2	2/0

[a]50 mg scavenger applied. [b]2 equiv scavenger applied.

Table S2. Optimization of solvents

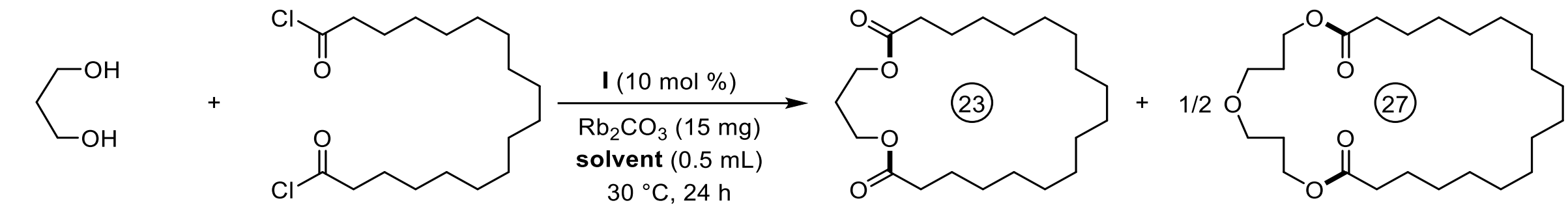

Entry	Solvent	**3a** (%)	**3a**/**3b**
1	$CDCl_3$	21	21/0
2	DCM	17	17/0
3	DCE	7	7/0
4	CCl_4	12	12/0
5	1,1,2,2-Tetrachloroethane	10	10/0
6	Trichloroethylene	16	16/0
7	Toluene	14	14/0
8	Mesitylene	24	24/0
9	1,3,5-Triethylbenzene	11	11/0
10	Xylene (mixture of isomers)	18	18/0
11	PhF	22	22/2
12	PhCl	21	21/0
13	PhBr	19	19/2
14	$PhCF_3$	16	16/14
15	*o*-DCB	15	15/2
16	*m*-DCB	17	17/2
17	1,3-Dimethyl-2-chlorobenzene	16	16/0
18	*n*-Hexane	11	11/0

From Table S2, $CDCl_3$, mesitylene, PhF, and PhCl were found to be promising solvents for further optimization (see Tables S5-S6 below).

Table S3. Optimization of reaction temperature

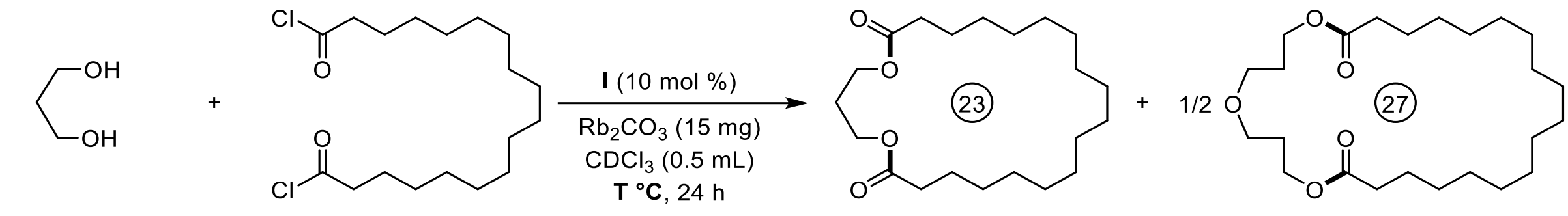

Entry	T (°C)	**3a** (%)	**3a**/**3b**
1	30	21	21/0
2	40	20	20/0
3	50	27	27/2
4	60	33	33/2

Table S4. Optimization of capsule loading

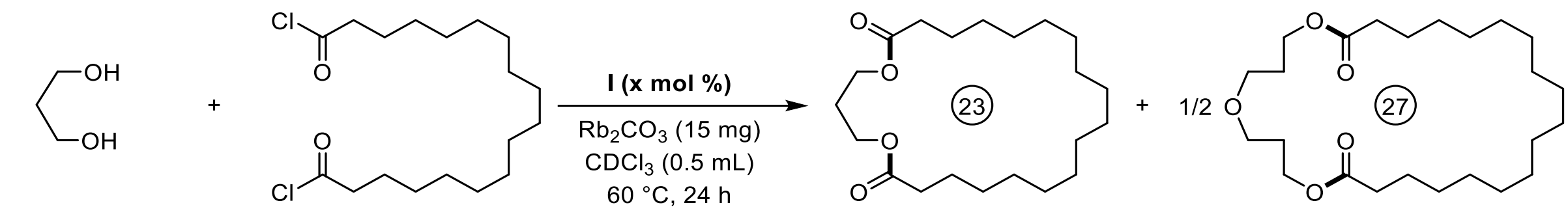

Entry	Capsule **I** (%)	**3a** (%)	**3a**/**3b**
1	10	33	33/2
2	20	44	44/2
3[a]	30	51	51/14

[a]1.0 mL solvent applied to dissolve the capsule instead of 0.50 mL (50 mM for **1a**).

Further optimization (0.1 mmol-scale)

Further experiments were performed following the same procedure in 5 mL Schlenk tubes instead of 2 mL vials unless otherwise stated. Shorter reaction time (3 h) was applied due to fast conversion of **1a**.

Table S5. Evaluation of the necessity of applying dehydration operation

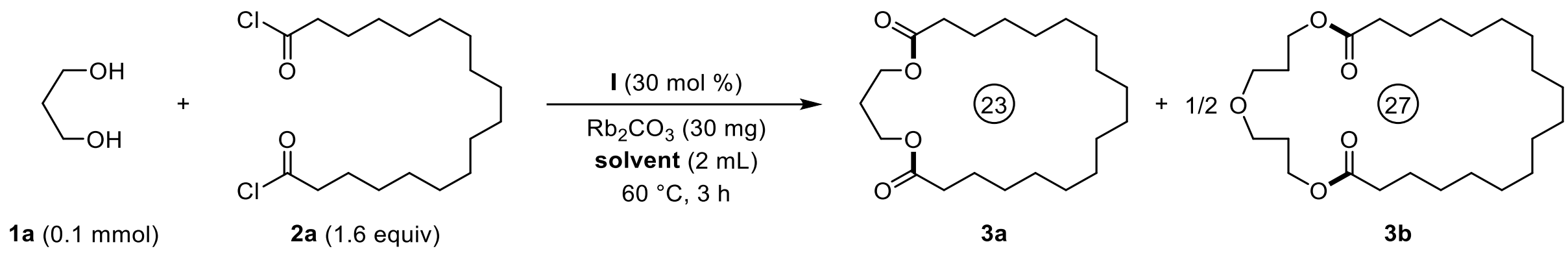

Entry	Dehydration operation	Solvent	**3a** (%)	**3a**/**3b**
1[a]	applied	$CDCl_3$	47	47/18
2[b]	not applied	$CDCl_3$	45	45/16
3[a]	applied	PhCl	46	46/14
4[b]	not applied	PhCl	56	56/12

[a] Following the aforementioned dehydration procedure for initial screening. [b] C_{11}-resorcin[4]arene directly dissolved with the solvent; detailed setup procedure can be found in Section 4.

Based on entries 3–4, dehydration operation was no longer applied for further optimization (Table S6–8).

Table S6. Further optimization of solvents

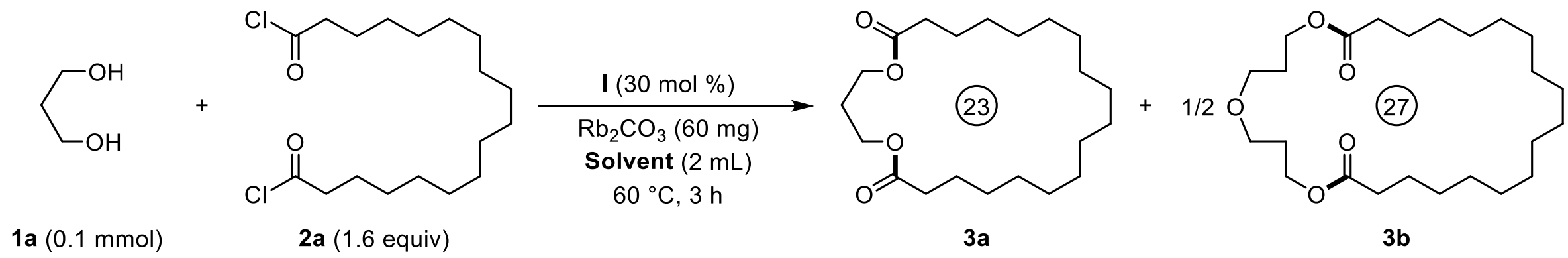

Entry	Solvent	**3a** (%)	**3a**/**3b**
1[a]	PhCl	56	56/12
2	PhCl	57	57/16
3	PhF	55	55/4
4	PhCl/PhF = 1:1	52	52/4
5	Mesitylene	40	40/2
6	PhCl/Mesitylene = 1:1	55	55/14
7	4-Chlorotoluene	45	45/2
8	3-Bromotoluene	40	40/6
9	1-Bromo-4-fluorobenzene	49	49/4
10	1-Bromo-4-butylbenzene	26	26/0

[a] 30 mg Rb_2CO_3 applied.

Table S7. Further optimization of equivalents, reaction temperature, and reaction time

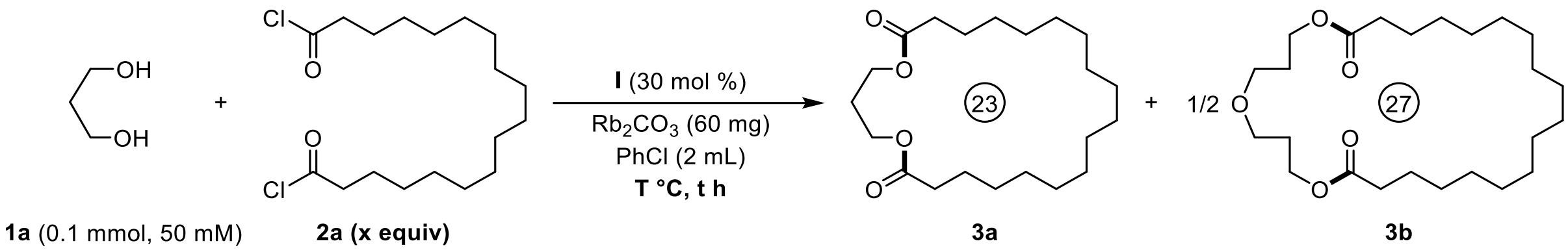

Entry	Eq. of **2a**	T (°C)	t (h)	**3a** (%)	**3a**/**3b**
1	1.6	60	3	57	57/16
2	1.6	70	3	57	57/16
3	1.8	70	2	56	56/14
4	2.0	70	1.5	58	58/12
5	2.2	70	1.5	53	53/10
6[a]	2.5	70	1.5	62 (47)	62/12
7	2.5	80	1.5	59	59/6

[a] Yield of isolated **3a** shown in parentheses.

Table S8. Final optimization: assessment of reaction setup

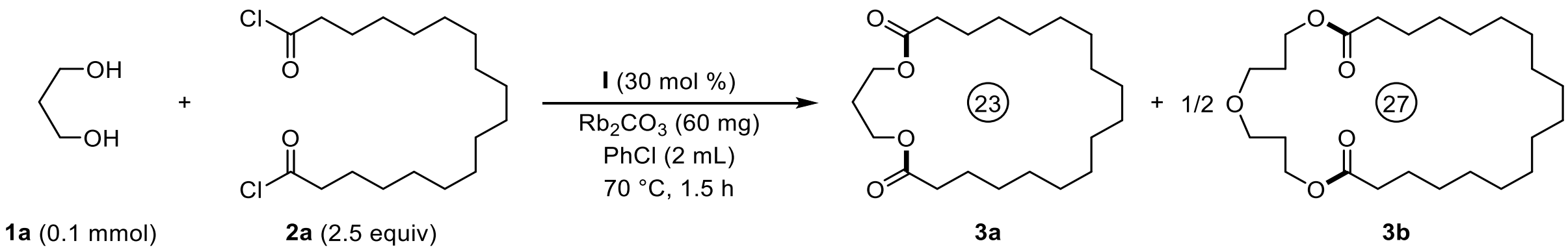

Entry	Reaction vessel	Addition of **2a**	**3a** (%)	**3a**/**3b**
1	4 mL vial with cap, air	in solid form	52	52/6
2	5 mL Schlenk tube, Ar	in solid form	62	62/12
3[a]	5 mL Schlenk tube, Ar	in PhCl solution form	60	60/10
4[abc]	5 mL preheated Schlenk tube, Ar	in PhCl solution form	67 (57)	67/6

[a] 1.5 mL PhCl for dissolving **1a** and C_{11}-resorcin[4]arene, 0.50 mL PhCl for dissolving **2a**, 2.0 mL PhCl in total. [b] For detailed procedure corresponding to optimal conditions (entry 4), see Section 4. [c] Yield of isolated **3a** shown in parentheses.

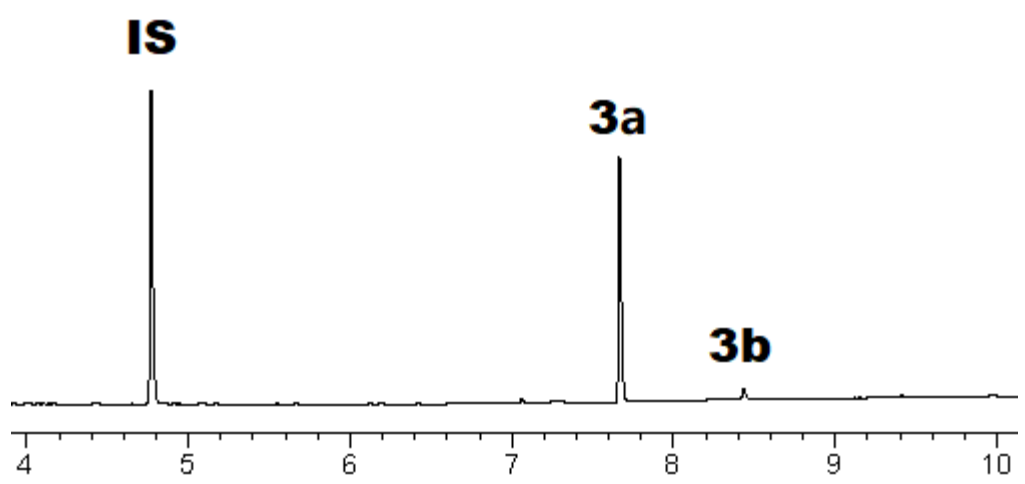

Figure S-1: GC traces of the optimal reaction conditions (Table S8, entry 4), IS: internal standard (*n*-pentadecane)

Control experiments for the model reaction

Several control experiments were carried out to elucidate the role of the capsule **I** and other components in the optimal reaction conditions. The same setup procedure was applied (see Section 4).

It is worth mentioning at this stage the internal standard for GC analysis was added after the reaction to avoid potential influence on the reaction (for all entries in Table 1 in the main text). Since our primary concern was the yield of isolated **3a**, the loss of **1a** during solution transfer process (typically 2-3% according to multiple measurements) was not taken into consideration when calculating GC yield for each control experiment. It was only taken into consideration when purifications were performed to further confirm the GC results (i.e., for (1), (2), and (4) below) by using dimethyl terephthalate as internal standard in acetone-d_6. Similarly, for substrate scope, we always measured the loss of each diol by applying this method to obtain a more precise yield (see Section 4 for details).

In general, full conversion of **1a** was detected by ^{1}H NMR analysis in acetone-d_6 for all these control experiments, and differences in both yield (**3a**) and selectivity (**3a**/**3b**) were observed. The details were described below.

(1) When capsule **I** was omitted and the reaction was performed under otherwise standard conditions (Table 1 in the main text, entry 2), the desired macrodiolide **3a** was detected in only 10% GC yield, and no detectable amount of byproduct **3b** was observed (see GC trace below).

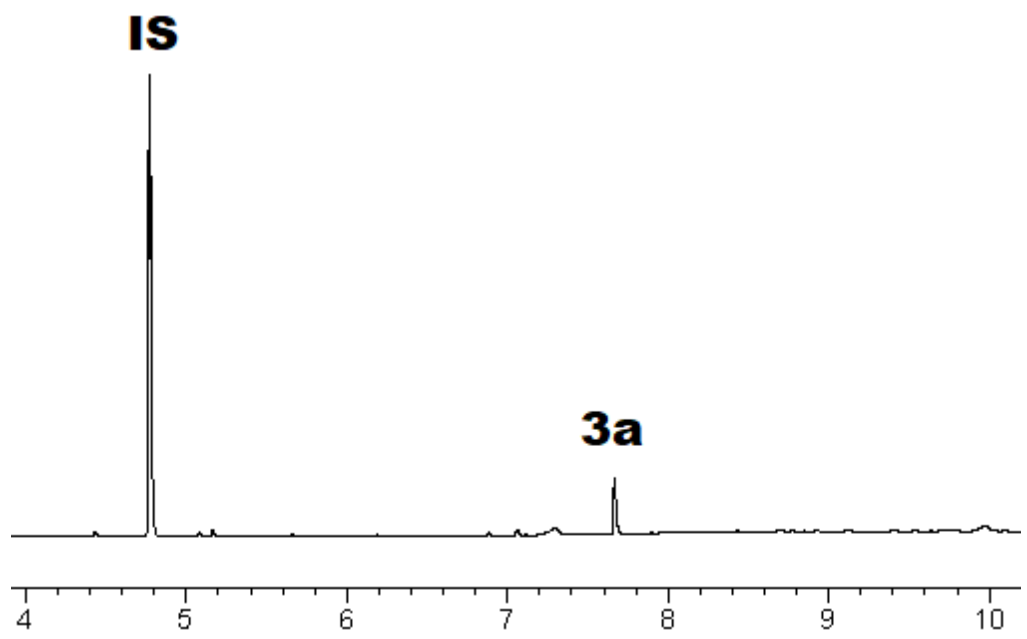

Figure S-2: GC traces of control experiment without capsule **I**, IS: internal standard (*n*-pentadecane)

To further confirm this result, purification by column chromatography was performed to give **3a** in 6% yield (1.9 mg **3a** obtained from 7.5 mg, 98.6 µmol **1a**; 2.83 µmol loss (2%) of **1a** during transfer process, indicating 95.8 µmol **1a** actually participated in the reaction).

This experiment indicated that the generation of **3a** was mainly attributed to a capsule-catalyzed process rather than a Rb_2CO_3-assisted process.

(2) When Rb_2CO_3 was omitted and the reaction was performed under otherwise standard conditions (Table 1 in the main text, entry 3), a decreased GC yield of **3a** (57%) and **3a**/**3b** ratio (57%/10%) was observed compared with standard conditions (see GC trace below).

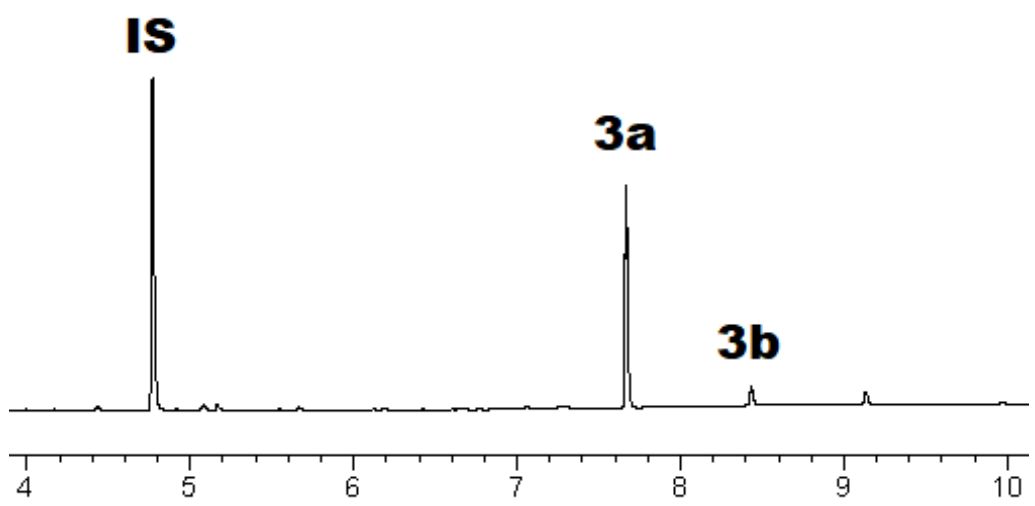

Figure S-3: GC traces of control experiment without Rb_2CO_3, IS: internal standard (*n*-pentadecane)

To further confirm this result, purification by column chromatography was performed to give **3a** in 43% yield (14.2 mg **3a** obtained from 7.4 mg, 97.2 µmol **1a**; 2.32 µmol loss (2%) during transfer process, indicating 94.9 µmol **1a** actually participated in the reaction).

This experiment further supported that the generation of **3a** was mainly attributed to a capsule-catalyzed process.

(3) When Rb_2CO_3 was replaced with nearly equal amount of Al_2O_3 (60.8 mg) and the reaction was performed under otherwise standard conditions (Table 1 in the main text, entry 4), a significantly decreased GC yield of **3a** (51%) and **3a**/**3b** ratio (51%/10%) was observed compared with standard conditions (see GC trace below).

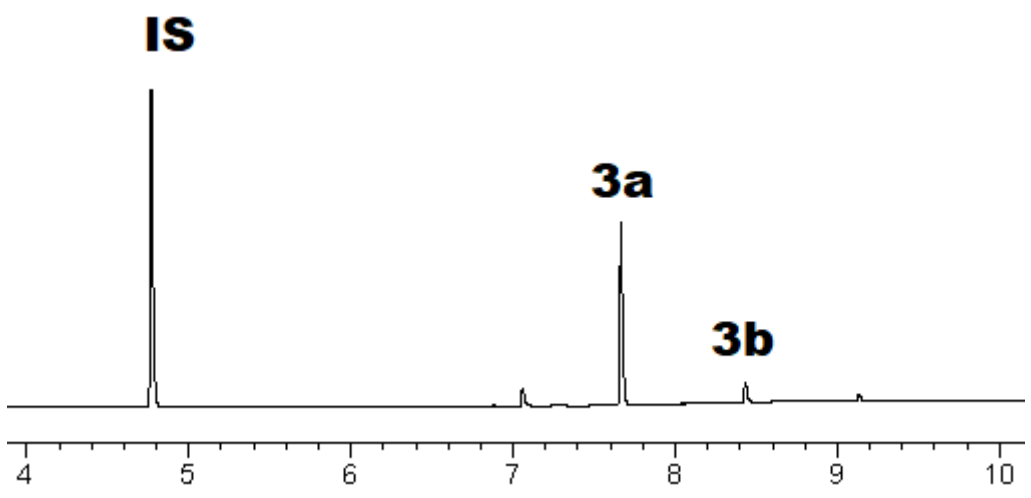

Figure S-4: GC traces of control experiment by replacing Rb_2CO_3 with basic Al_2O_3, IS: internal standard (*n*-pentadecane)

This experiment showcased a superior behavior of Rb_2CO_3 compared with basic Al_2O_3, and highlighted the importance of choosing a competent HCl scavenger for the model reaction.

(4) When additional 2 equiv of $^{n}Bu_4N^{+}Br^{-}$ (TBAB, 65.1 mg, 0.202 mmol) was applied and the reaction was performed under otherwise standard conditions (Table 1 in the main text, entry 5), the desired macrodiolide **3a** was detected in only 9% GC yield, and no detectable amount of byproduct **3b** was observed (see GC trace below).

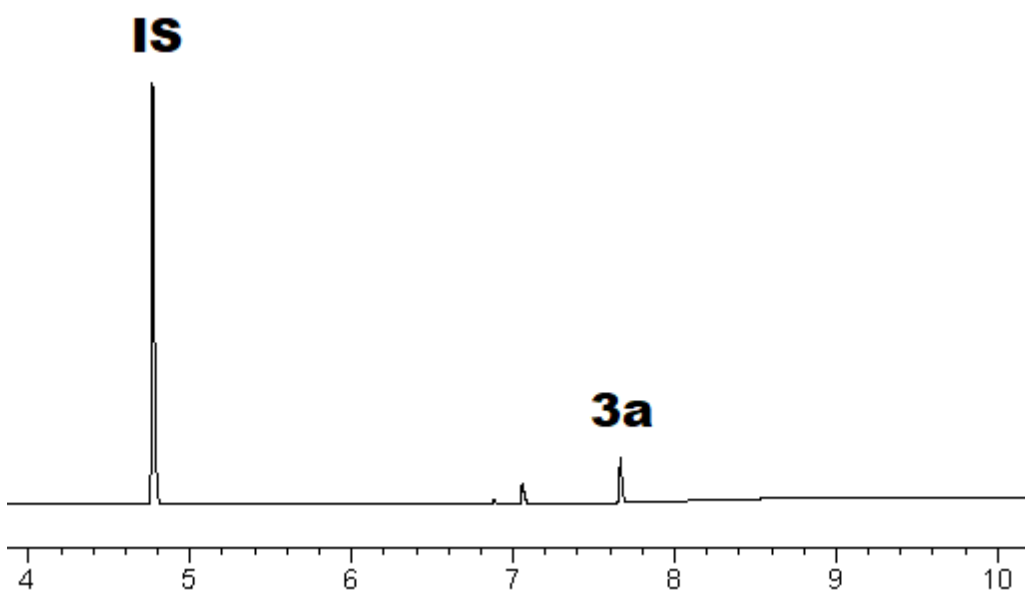

Figure S-5: GC traces of control experiment by blocking the capsule with TBAB, IS: internal standard (*n*-pentadecane)

To further confirm this result, purification by column chromatography was performed to give **3a** in 4% yield (1.5 mg **3a** obtained from 7.6 mg, 99.9 µmol **1a**; 2.75 µmol loss (3%) during transfer process, indicating 97.2 µmol **1a** actually participated in the reaction).

This experiment supported the role of the capsule as a supramolecular catalyst in the model reaction.

(5) When the capsule was replaced with 720 mol % (= 30 mol % × 6 × 4) subunit 4,6-diethylbenzene-1,3-diol[6] and the reaction was performed under otherwise standard conditions (Table 1 in the main text, entry 6), the desired macrodiolide **3a** was detected in only 9% GC yield, and no detectable amount of byproduct **3b** was observed (see GC trace below).

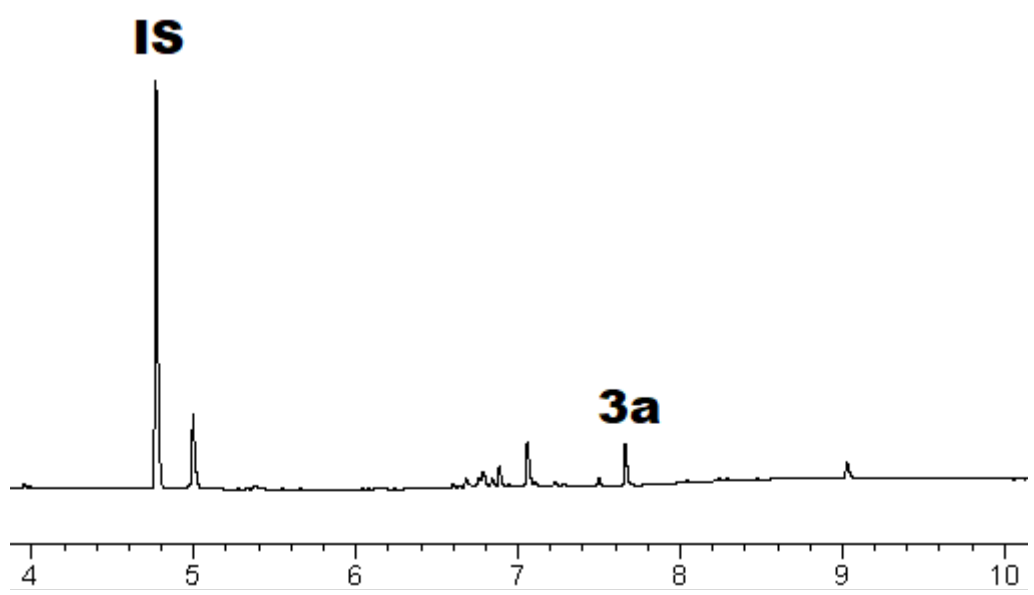

Figure S-6: GC traces of control experiment by replacing the capsule with subunit, IS: internal standard (*n*-pentadecane)

This experiment confirmed that the intact capsule structure is essential for catalysis.

(6) When additional 10 equiv of dry ethyl acetate (EA, 0.10 mL, 1.0 mmol) was applied and the reaction was performed under otherwise standard conditions (Table 1 in the main text, entry 7), neither the desired macrodiolide **3a** nor byproduct **3b** was detected (see GC trace below).

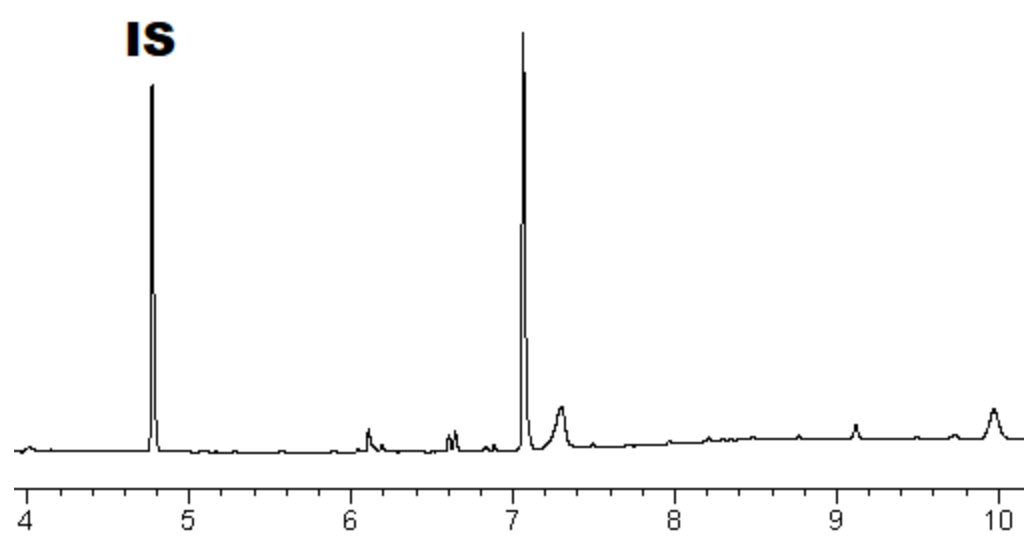

Figure S-7: GC traces of control experiment by adding EA, IS: internal standard (*n*-pentadecane)

Since EA is a polar solvent which can disassemble the capsule, this experiment supported the role of the capsule as a supramolecular catalyst in the model reaction.

(7) When the capsule and Rb_2CO_3 were replaced with 3 equiv $ZnCl_2$ (44.2 mg, 0.324 mmol, excess amount applied due to solubility issue) and the reaction was performed under otherwise standard conditions (Table 1 in the main text, entry 8), neither the desired macrodiolide **3a** nor byproduct **3b** was detected (see GC trace below). This experiment indicated the uniqueness of the capsule in catalyzing the model reaction.

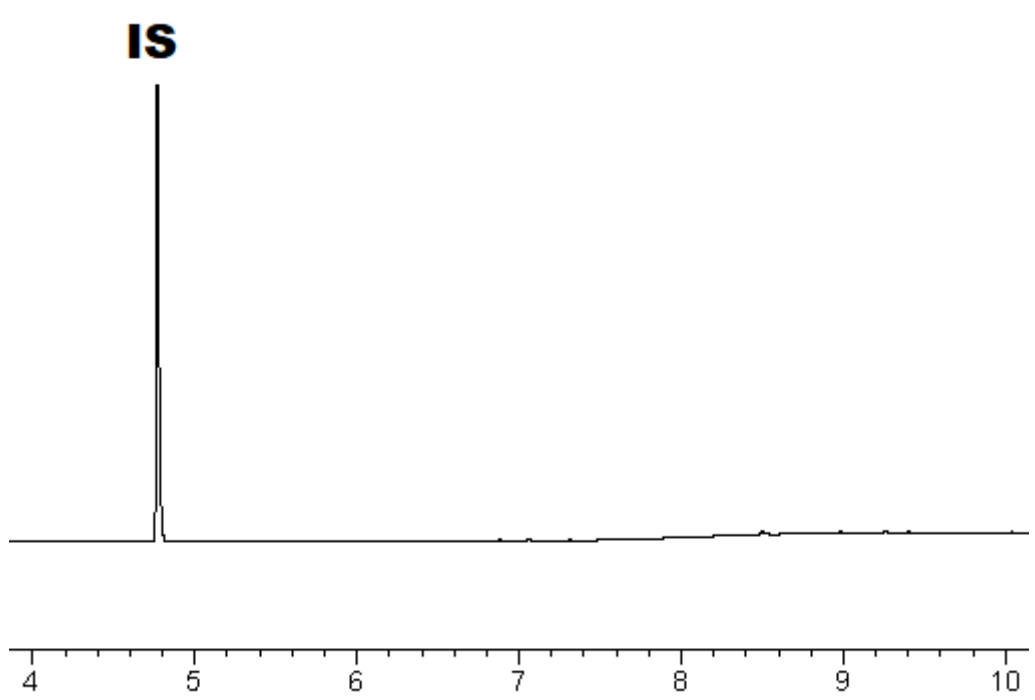

Figure S-8: GC traces of control experiment under a Lewis-acid condition, IS: internal standard (*n*-pentadecane)

4. Synthesis and characterization of macrodiolides and macrotetrolides

General procedure B: synthesis of macrodiolides and macrotetrolides

Preparation of the reaction tube: Rb_2CO_3 was dried in an oven at 120 °C for at least 3 days and then weighed (60 ± 2 mg) into a 5 mL Schlenk tube (*l* = 100 mm) charged with a stir bar (6 mm × 10 mm). The tube was sealed with a rubber septum, evacuated/backfilled with Ar for three cycles while being heated with a heat gun, and subsequently protected with an argon balloon. After cooling to room temperature, the tube was immersed into a 70 °C oil bath and preheated for at least 10 min with stirring at 800 rpm.

Preparation of solution A (diol + C_{11}-resorcin[4]arene in PhCl): C_{11}-resorcin[4]arene (0.180 mmol, 1.80 equiv, corresponding to 30 mol % hexameric resorcin[4]arene capsule **I**) was added to an oven-dried 4 mL vial charged with a stir bar (3 mm × 8 mm size), then the vial was sealed with a cap featuring a septum. 1.5 mL of dry PhCl was injected through the septum to dissolve C_{11}-resorcin[4]arene. Diol (0.100 mmol, 1.00 equiv) was then added to the solution. If the diol was a liquid, it was introduced through the septum using a microsyringe; if it was a solid, it was added directly to the solution. The resulting mixture was stirred and heated at 40–70 °C until a clear solution was obtained.

Preparation of solution B (diacyl chloride in PhCl): if the diacyl chloride is a solid, it was rapidly weighed into another oven-dried 4 mL vial (0.250 mmol, 2.50 equiv). The vial was evacuated and backfilled with Ar for three cycles, then protected with an Ar balloon. 0.50 mL of dry PhCl was added through the septum to dissolve the chloride. If the diacyl chloride was a liquid, then typically 1.00 mmol of such chloride was freshly prepared and dissolved in 2.0 mL dry PhCl under Ar protection. An aliquot of 0.50 mL of this solution, corresponding to 0.250 mmol (2.50 equiv), was then used for the reaction.

Mixing of solution A and solution B: solution A and solution B were simultaneously injected into the preheated Schlenk tube using two syringes, each delivered in a single portion without any slow-addition procedure. Therefore, the concentration of diol in the reaction mixture was 50 mM and that of diacyl chloride was 125 mM. In order to obtain precise yields on the 0.1 mmol scale, the loss of the diol in solution A during such transfer process was measured by ^1H NMR by using dimethyl terephthalate as internal standard in acetone-d_6 (typically 2-3% loss was detected). Such loss became totally negligible on larger scale (e.g. 1 mmol scale, see Section 5). The loss of the diacyl chloride in solution B was negligible since excess amount was applied.

Work-up, GC analysis (optional), and product separation: after heating and stirring for 1.5 hours, the oil bath was removed, the Schlenk tube was allowed to cool down to room temperature. If determination of a GC yield was planned, then internal standard *n*-pentadecane (28.0 µL, 21.5 mg, 0.101 mmol) was added to the reaction mixture at this stage and a GC sample was prepared by following the same procedure described in Section 3. Afterwards, the reaction mixture was diluted with DCM (5.0 mL), added 0.10 mL Et_3N to further neutralize the reaction mixture (this step was omitted if expecting to fully recover C_{11}-resorcin[4]arene, see Section 5), and filtered through a pad of Celite (ca. 4 g, and washed with 3 × 5.0 mL DCM). The solvent was removed, and the residue was purified by flash column chromatography (ca. 20 g silica, manual column, *d* = 20 mm, *l* = 150 mm) to afford the desired product (typically, *n*-hexane/ethyl acetate = 40:1 to 20:1 to 15:1 for macrodiolides, *n*-hexane/ethyl acetate = 40:1 to 15:1 to 5:1 for macrotetrolides).

1,5-dioxacyclotricosane-6,23-dione (3a)

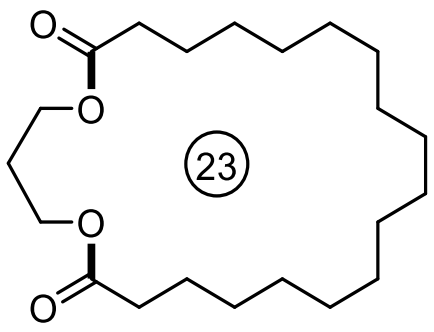

Chemical Formula: $C_{21}H_{38}O_4$
Molecular Weight: 354.53

3a

Following general procedure B, **3a** was obtained from propane-1,3-diol (7.5 mg, 98.6 μmol), octadecanedioyl diacyl chloride (88.1 mg, 0.251 mmol), C_{11}-resorcin[4]arene (201 mg, 0.182 mmol, corresponding to 30.3 μmol capsule), and Rb_2CO_3 (61.3 mg). 1.95 μmol loss (2%) of propane-1,3-diol during transfer process was detected by ^{1}H NMR. Therefore, 96.6 μmol propane-1,3-diol actually participated in the reaction.

Yield: 19.5 mg, 57%

Property: colorless oil

$\mathbf{R}_f$ 0.57 (*n*-hexane/ethyl acetate = 4:1)

IR (ATR): ν_{max} (cm^{-1}) = 2924s, 2854m, 1736s, 1460m, 1350w, 1167m, 1107w, 1047m, 907w, 731m.

^{1}H NMR (500 MHz, $CDCl_3$) δ 4.16 (t, J = 6.2 Hz, 4H), 2.31 (t, J = 7.1 Hz, 4H), 1.98 (p, J = 6.2 Hz, 2H), 1.63 (p, J = 7.1 Hz, 4H), 1.36 – 1.24 (m, 24H).

^{13}C{^{1}H} NMR (126 MHz, $CDCl_3$) δ 174.0, 61.1, 34.4, 28.6 (2 overlapping peaks), 28.5, 28.4, 28.2, 28.1 (2 overlapping peaks), 24.9.

LCMS (ESI$^+$) *m/z* calculated for $C_{21}H_{38}NaO_4^+$ [M + Na]$^+$: 377.27; found: 377.20.

HRMS (ESI$^+$) *m/z* calculated for $C_{21}H_{38}NaO_4^+$ [M + Na]$^+$: 377.2662; found: 377.2667.

1,5,9-trioxacycloheptacosane-10,27-dione (3b)

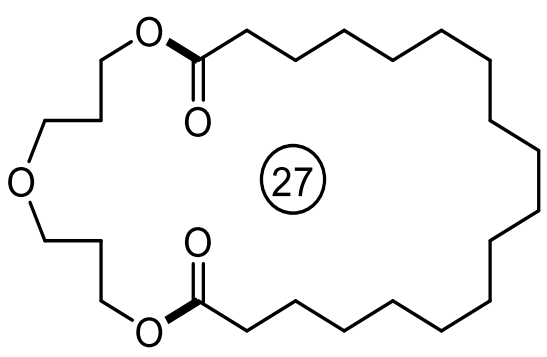

Chemical Formula: $C_{24}H_{44}O_5$
Molecular Weight: 412.61

3b

Following general procedure B, **3b** was obtained from 3,3'-oxybis(propan-1-ol) (14.0 mg, 104 μmol), octadecanedioyl diacyl chloride (93.1 mg, 0.265 mmol), C_{11}-resorcin[4]arene (199 mg, 0.180 mmol, corresponding to 30.0 μmol capsule), and Rb_2CO_3 (60.3 mg). 2.42 μmol loss (2%) of 3,3'-oxybis(propan-1-ol) during transfer process was detected by ^{1}H NMR. Therefore, 102 μmol 3,3'-oxybis(propan-1-ol) actually participated in the reaction.

Yield: 18.8 mg, 45%

Property: colorless oil

$\mathbf{R}_f$ 0.43 (*n*-hexane/ethyl acetate = 4:1)

IR (ATR): ν_{max} (cm^{-1}) = 2923s, 2854m, 1734s, 1460m, 1356w, 1245m, 1172m, 1121m, 722w.

^{1}H NMR (500 MHz, $CDCl_3$) δ 4.17 (t, J = 6.2 Hz, 4H), 3.49 (t, J = 6.3 Hz, 4H), 2.31 (t, J = 7.2 Hz, 4H), 1.89 (p, J = 6.3 Hz, 4H), 1.64 (p, J = 7.2 Hz, 4H), 1.37 – 1.23 (m, 24H).

^{13}C{^{1}H} NMR (126 MHz, $CDCl_3$) δ 174.0, 67.6, 61.4, 34.5, 29.3, 28.9, 28.8 (4 close peaks), 28.7, 25.0.

LCMS (ESI^+) *m/z* calculated for $C_{24}H_{44}NaO_5^+$ $[M + Na]^+$: 435.31; found: 435.30.

HRMS (ESI^+) *m/z* calculated for $C_{24}H_{44}NaO_5^+$ $[M + Na]^+$: 435.3081; found: 435.3090.

1,8-dioxacyclooctadecane-2,7-dione (3e)

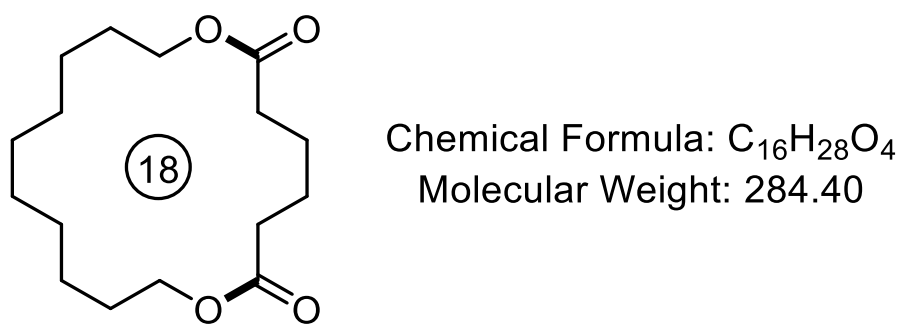

3e

Following general procedure B, **3e** was obtained from decane-1,10-diol (17.5 mg, 100 µmol), adipoyl diacyl chloride (prepared from 147 mg adipic acid and 1/4 of the material applied, corresponding to 45.9 mg diacyl chloride, 0.251 mmol), C_{11}-resorcin[4]arene (199 mg, 0.180 mmol, corresponding to 30.0 µmol capsule), and Rb_2CO_3 (60.2 mg). 2.58 µmol loss (3%) of decane-1,10-diol during transfer process was detected by ^{1}H NMR. Therefore, 97.4 µmol decane-1,10-diol actually participated in the reaction.

Yield: 8.5 mg, 31%

Property: colorless oil

R_f 0.48 (*n*-hexane/ethyl acetate = 4:1)

IR (ATR): ν_{max} (cm^{-1}) = 2929s, 2858s, 1734s, 1460m, 1387w, 1350w, 1240m, 1181m, 1080w, 492w.

^{1}H NMR (500 MHz, $CDCl_3$) δ 4.13 (t, J = 5.7 Hz, 4H), 2.38 – 2.28 (m, 4H), 1.71 – 1.65 (m, 4H), 1.64 – 1.58 (m, 4H), 1.40 – 1.34 (m, 4H), 1.33 – 1.29 (m, 8H).

^{13}C{^{1}H} NMR (126 MHz, $CDCl_3$) δ 173.5, 64.2, 34.7, 28.3, 27.5, 27.4, 25.1, 25.0.

LCMS (ESI^+) *m/z* calculated for $C_{16}H_{28}NaO_4^+$ $[M + Na]^+$: 307.19; found: 307.30.

HRMS (ESI^+) *m/z* calculated for $C_{16}H_{28}NaO_4^+$ $[M + Na]^+$: 307.1880; found: 307.1886.

1,8-dioxacycloicosane-2,7-dione (3f)

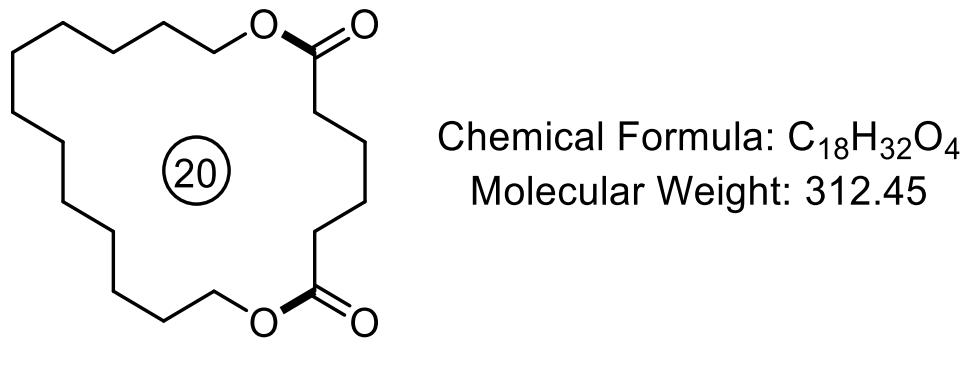

3f

Following general procedure B, **3f** was obtained from dodecane-1,12-diol (20.9 mg, 103 µmol), adipoyl diacyl chloride (prepared from 147 mg adipic acid and 1/4 of the material applied, corresponding to 45.9 mg diacyl chloride, 0.251 mmol), C_{11}-resorcin[4]arene (201 mg, 0.182 mmol, corresponding to 30.3 µmol capsule), and Rb_2CO_3 (62.7 mg). 3.65 µmol loss (4%) of dodecane-1,12-diol during transfer

process was detected by ^{1}H NMR. Therefore, 99.4 µmol dodecane-1,12-diol actually participated in the reaction.

Yield: 12.0 mg, 39%

Property: colorless oil

R*f* 0.48 (*n*-hexane/ethyl acetate = 4:1)

IR (ATR): ν_{max} (cm^{-1}) = 2926s, 2856s, 1732s, 1460m, 1389w, 1350w, 1242m, 1178m, 1140m, 1078w, 725w.

^{1}H NMR (500 MHz, $CDCl_3$) δ 4.11 (t, J = 6.0 Hz, 4H), 2.37 – 2.29 (m, 4H), 1.71 – 1.66 (m, 4H), 1.66 – 1.59 (m, 4H), 1.41 – 1.34 (m, 4H), 1.34 – 1.24 (m, 12H).

^{13}C{^{1}H} NMR (126 MHz, $CDCl_3$) δ 173.5, 64.3, 34.4, 28.2, 28.1, 27.9, 27.7, 25.5, 24.7.

LCMS (ESI$^+$) *m/z* calculated for $C_{18}H_{32}NaO_4^+$ [M + Na]$^+$: 335.22; found: 335.15.

HRMS (ESI$^+$) *m/z* calculated for $C_{18}H_{33}O_4^+$ [M + H]$^+$: 313.2373; found: 313.2379.

1,8-dioxacyclodocosane-2,7-dione (3g)

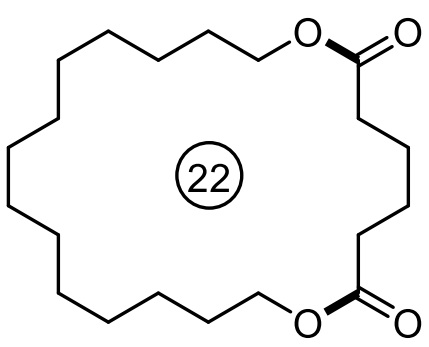

3g

Chemical Formula: $C_{20}H_{36}O_4$
Molecular Weight: 340.50

Following general procedure B, **3g** was obtained from tetradecane-1,14-diol (23.4 mg, 102 µmol), adipoyl diacyl chloride (prepared from 147 mg adipic acid and 1/4 of the material applied, corresponding to 45.9 mg diacyl chloride, 0.251 mmol), C_{11}-resorcin[4]arene (201 mg, 0.182 mmol, corresponding to 30.3 µmol capsule), and Rb_2CO_3 (62.4 mg). 3.90 µmol loss (4%) of tetradecane-1,14-diol during transfer process was detected by ^{1}H NMR. Therefore, 98.1 µmol tetradecane-1,14-diol actually participated in the reaction.

Yield: 14.7 mg, 44%

Property: colorless oil

R*f* 0.52 (*n*-hexane/ethyl acetate = 4:1)

IR (ATR): ν_{max} (cm^{-1}) = 2925s, 2854s, 1734s, 1460m, 1389w, 1351w, 1241m, 1173m, 1140m, 1077w, 723w.

^{1}H NMR (500 MHz, $CDCl_3$) δ 4.10 (t, J = 6.1 Hz, 4H), 2.36 – 2.30 (m, 4H), 1.72 – 1.65 (m, 4H), 1.65 – 1.59 (m, 4H), 1.45 – 1.24 (m, 20H).

^{13}C{^{1}H} NMR (126 MHz, $CDCl_3$) δ 173.5, 64.6, 34.3, 28.5 (2 close peaks), 28.3 (2 close peaks), 28.2, 25.7, 24.6.

LCMS (ESI$^+$) *m/z* calculated for $C_{20}H_{36}NaO_4^+$ [M + Na]$^+$: 363.25; found: 363.30.

HRMS (ESI$^+$) *m/z* calculated for $C_{20}H_{37}O_4^+$ [M + H]$^+$: 341.2686; found: 341.2691.

1,6-dioxacyclotetracosane-7,24-dione (3h)

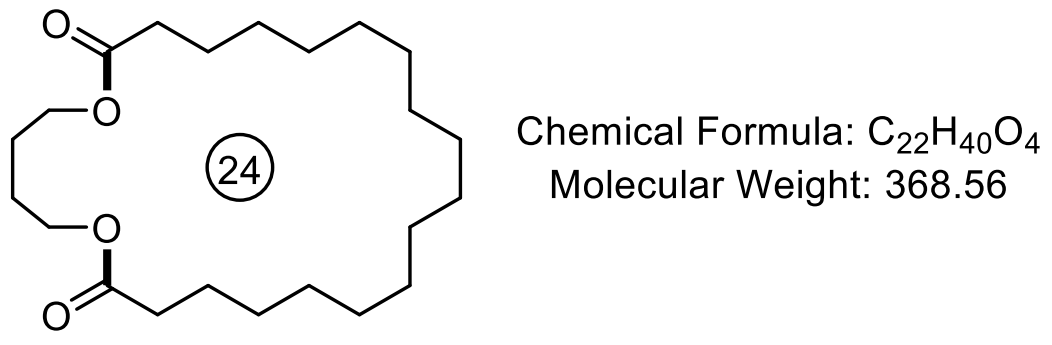

3h

Following general procedure B, **3h** was obtained from butane-1,4-diol (9.6 mg, 106 μmol), octadecanedioyl dichloride (87.7 mg, 0.250 mmol), C_{11}-resorcin[4]arene (201 mg, 0.182 mmol, corresponding to 30.3 μmol capsule), and Rb_2CO_3 (59.7 mg). 4.65 μmol loss (4%) of butane-1,4-diol during transfer process was detected by ^{1}H NMR. Therefore, 101 μmol butane-1,4-diol actually participated in the reaction.

Yield: 18.0 mg, 48%

Property: colorless oil

R*f* 0.56 (*n*-hexane/ethyl acetate = 4:1)

IR (ATR): ν_{max} (cm^{-1}) = 2924s, 2854s, 1734s, 1461m, 1388w, 1349w, 1245m, 1169m, 1106m, 1071w, 1040w, 723w.

^{1}H NMR (500 MHz, $CDCl_3$) δ 4.16 – 4.06 (m, 4H), 2.31 (t, J = 7.3 Hz, 4H), 1.75 – 1.68 (m, 4H), 1.64 (p, J = 7.2 Hz, 4H), 1.36 – 1.23 (m, 24H).

^{13}C{^{1}H} NMR (126 MHz, $CDCl_3$) δ 174.0, 63.9, 34.5, 28.6, 28.5 (2 close peaks), 28.4 (2 overlapping peaks), 28.3, 25.6, 24.9.

LCMS (ESI$^+$) *m/z* calculated for $C_{22}H_{40}NaO_4^+$ [M + Na]$^+$: 391.28; found: 391.25.

HRMS (ESI$^+$) *m/z* calculated for $C_{22}H_{41}O_4^+$ [M + H]$^+$: 369.2999; found: 369.3013.

1,8-dioxacyclohexacosane-9,26-dione (3i)

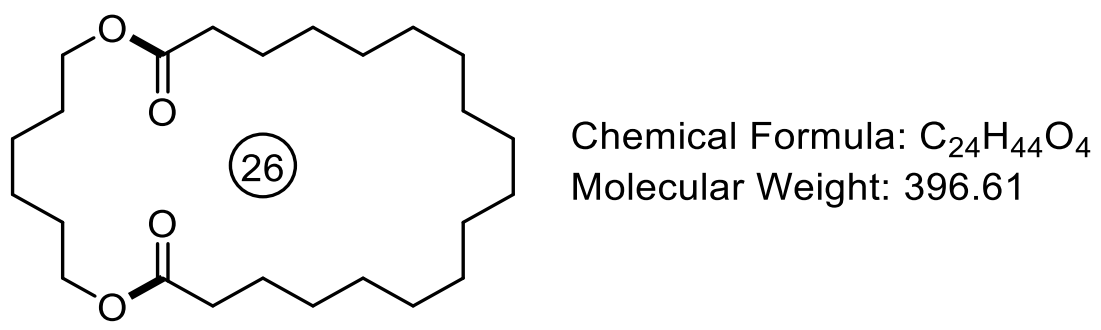

3i

Following general procedure B, **3i** was obtained from hexane-1,6-diol (11.8 mg, 99.8 μmol), octadecanedioyl dichloride (88.3 mg, 0.251 mmol), C_{11}-resorcin[4]arene (200 mg, 0.181 mmol, corresponding to 30.1 μmol capsule), and Rb_2CO_3 (61.5 mg). 3.98 μmol loss (4%) of hexane-1,6-diol during transfer process was detected by ^{1}H NMR. Therefore, 95.8 μmol hexane-1,6-diol actually participated in the reaction.

Yield: 18.1 mg, 48%

Property: colorless oil

R*f* 0.57 (*n*-hexane/ethyl acetate = 4:1)

IR (ATR): ν_{max} (cm^{-1}) = 2924s, 2854s, 1734s, 1461m, 1348w, 1242m, 1170m, 1106m, 906w, 728m.

^{1}H NMR (500 MHz, $CDCl_3$) δ 4.07 (t, J = 6.5 Hz, 4H), 2.30 (t, J = 7.2 Hz, 4H), 1.67 – 1.61 (m, 8H), 1.42 – 1.38 (m, 4H), 1.34 – 1.25 (m, 24H).

^{13}C{^{1}H} NMR (126 MHz, $CDCl_3$) δ 174.2, 60.3, 34.5, 28.8 (4 close peaks), 28.7 (2 close peaks), 28.6, 25.9, 25.0.

LCMS (ESI$^+$) *m/z* calculated for $C_{24}H_{44}NaO_4^+$ [M + Na]$^+$: 419.31; found: 419.25.

HRMS (ESI$^+$) *m/z* calculated for $C_{24}H_{44}NaO_4^+$ [M + Na]$^+$: 419.3132; found: 419.3137.

1,10-dioxacyclooctacosane-11,28-dione (3j)

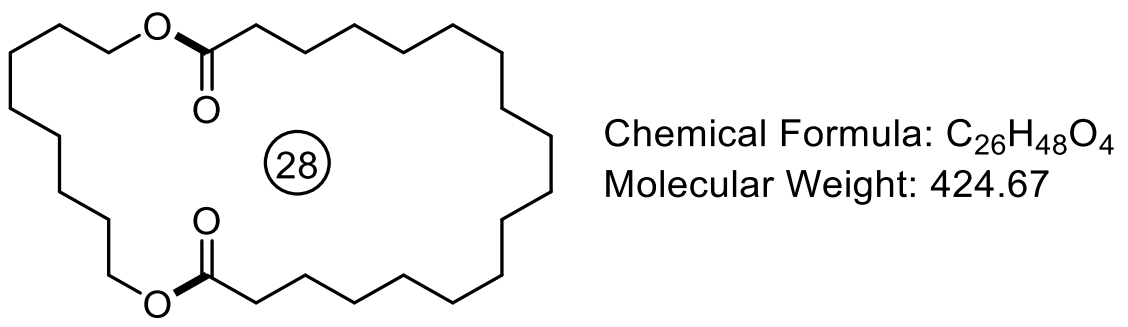

3j

Following general procedure B, **3j** was obtained from octane-1,8-diol (14.6 mg, 99.8 μmol), octadecanedioyl dichloride (88.1 mg, 0.251 mmol), C_{11}-resorcin[4]arene (200 mg, 0.181 mmol, corresponding to 30.1 μmol capsule), and Rb_2CO_3 (62.5 mg). 4.12 μmol loss (4%) of octane-1,8-diol during transfer process was detected by ^{1}H NMR. Therefore, 95.7 μmol octane-1,8-diol actually participated in the reaction.

Yield: 20.9 mg, 51%

Property: white solid (melting point: 38–39 °C)

R*f* 0.60 (*n*-hexane/ethyl acetate = 4:1)

IR (ATR): ν_{max} (cm^{-1}) = 2916s, 2851s, 1730s, 1474m, 1360m, 1273m, 1257m, 1207s, 1162s, 1116m, 1063m, 1026w, 967m, 885w, 718m, 456w.

^{1}H NMR (500 MHz, $CDCl_3$) δ 4.07 (t, J = 6.4 Hz, 4H), 2.31 (t, J = 7.1 Hz, 4H), 1.67 – 1.59 (m, 8H), 1.40 – 1.24 (m, 32H).

^{13}C{^{1}H} NMR (126 MHz, $CDCl_3$) δ 174.2, 64.4, 34.6, 29.3, 29.2 (2 close peaks), 29.1, 29.0, 28.8 (3 close peaks), 26.1, 25.1.

LCMS (ESI$^+$) *m/z* calculated for $C_{26}H_{48}NaO_4^+$ [M + Na]$^+$: 447.34; found: 447.30.

HRMS (ESI$^+$) *m/z* calculated for $C_{26}H_{48}NaO_4^+$ [M + Na]$^+$: 447.3445; found: 447.3453.

1,12-dioxacyclotriacontane-13,30-dione (3k)

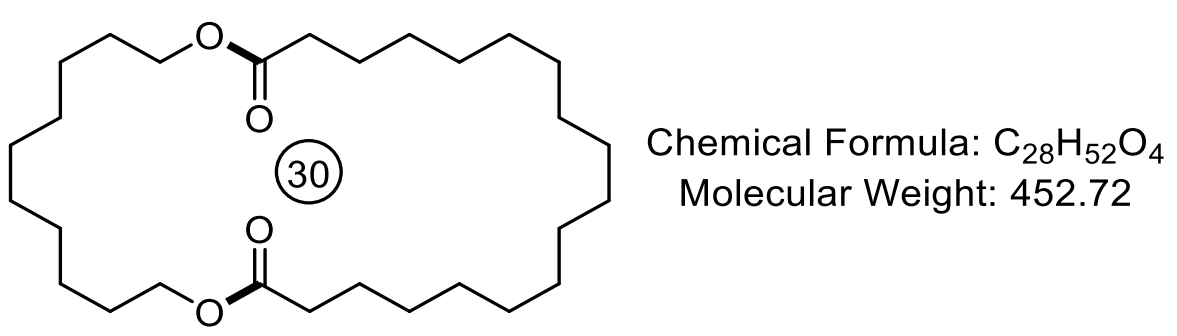

3k

Following general procedure B, **3k** was obtained from decane-1,10-diol (17.4 mg, 99.8 μmol), octadecanedioyl dichloride (87.6 mg, 0.249 mmol), C_{11}-resorcin[4]arene (201 mg, 0.182 mmol, corresponding to 30.3 μmol capsule), and Rb_2CO_3 (61.5 mg). 2.82 μmol loss (3%) of decane-1,10-diol

during transfer process was detected by ^{1}H NMR. Therefore, 97.0 µmol decane-1,10-diol actually participated in the reaction.

Yield: 16.7 mg, 38%

Property: white solid (melting point: 58–59 °C)

R*f* 0.64 (*n*-hexane/ethyl acetate = 4:1)

IR (ATR): ν_{max} (cm^{-1}) = 2915s, 2848s, 1727s, 1464m, 1387w, 1351w, 1334w, 1256s, 1215s, 1174s, 1147s, 1108s, 1087m, 1074w, 1063w, 1041w, 1020w, 1002w, 974w, 947w, 911m, 815w, 793w, 735s, 622w, 527w, 471w.

^{1}H NMR (500 MHz, $CDCl_3$) δ 4.08 (t, J = 6.4 Hz, 4H), 2.30 (t, J = 7.1 Hz, 4H), 1.67 – 1.58 (m, 8H), 1.44 – 1.18 (m, 36H).

^{13}C{^{1}H} NMR (126 MHz, $CDCl_3$) δ 174.2, 64.4, 34.7, 29.6, 29.5, 29.4 (2 close peaks), 29.3, 29.2, 29.0, 28.8 (2 overlapping peaks), 26.2, 25.2.

LCMS (ESI^{+}) *m/z* calculated for $C_{28}H_{52}NaO_4^{+}$ [M + Na]$^{+}$: 475.38; found: 475.35.

HRMS (ESI^{+}) *m/z* calculated for $C_{28}H_{52}NaO_4^{+}$ [M + Na]$^{+}$: 475.3758; found: 475.3764.

1,14-dioxacyclodotriacontane-15,32-dione (3l)

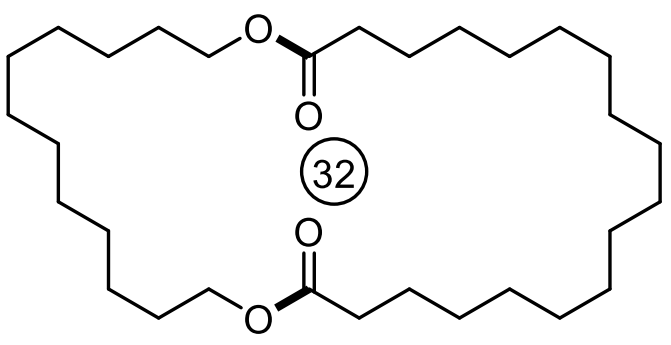

Chemical Formula: $C_{30}H_{56}O_4$
Molecular Weight: 480.77

3l

Following general procedure B, **3l** was obtained from dodecane-1,12-diol (21.0 mg, 104 µmol), octadecanedioyl dichloride (88.6 mg, 0.252 mmol), C_{11}-resorcin[4]arene (200 mg, 0.181 mmol, corresponding to 30.1 µmol capsule), and Rb_2CO_3 (61.2 mg). 3.95 µmol loss (4%) of dodecane-1,12-diol during transfer process was detected by ^{1}H NMR. Therefore, 100 µmol dodecane-1,12-diol actually participated in the reaction.

Yield: 16.0 mg, 33%

Property: white solid (melting point: 64–65 °C)

R*f* 0.60 (*n*-hexane/ethyl acetate = 4:1)

IR (ATR): ν_{max} (cm^{-1}) = 2914s, 2849s, 1732s, 1469m, 1379w, 1344w, 1251m, 1237m, 1164s, 1108s, 1063m, 1002w, 857w, 720m, 610w.

^{1}H NMR (500 MHz, $CDCl_3$) δ 4.08 (t, J = 6.3 Hz, 4H), 2.30 (t, J = 7.1 Hz, 4H), 1.67 – 1.57 (m, 8H), 1.42 – 1.19 (m, 40H).

^{13}C{^{1}H} NMR (126 MHz, $CDCl_3$) δ 174.2, 64.4, 34.7, 29.6 (4 close peaks), 29.4 (3 close peaks), 29.1, 28.9, 28.8, 26.2, 25.2.

LCMS (ESI^{+}) *m/z* calculated for $C_{30}H_{56}NaO_4^{+}$ [M + Na]$^{+}$: 503.41; found: 503.40.

HRMS (ESI^{+}) *m/z* calculated for $C_{30}H_{57}O_4^{+}$ [M + H]$^{+}$: 481.4251; found: 481.4257.

1,10-dioxacycloicosane-2,9-dione (3m)

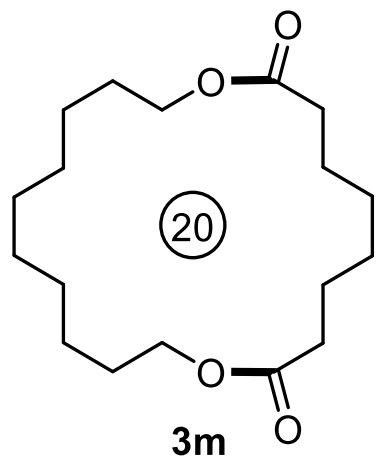

3m

Chemical Formula: $C_{18}H_{32}O_4$
Molecular Weight: 312.45

Following general procedure B, **3m** was obtained from decane-1,10-diol (17.5 mg, 100 µmol), octanedioyl diacyl chloride (prepared from 174 mg octanedioic acid and 1/4 of the material applied, corresponding to 52.8 mg diacyl chloride, 0.250 mmol), C_{11}-resorcin[4]arene (201 mg, 0.182 mmol, corresponding to 30.3 µmol capsule), and Rb_2CO_3 (61.6 mg). 1.45 µmol loss (1%) of decane-1,10-diol during transfer process was detected by ^{1}H NMR. Therefore, 98.6 µmol decane-1,10-diol actually participated in the reaction.

Yield: 14.4 mg, 47%

Property: colorless oil

$\mathbf{R}_f$ 0.56 (*n*-hexane/ethyl acetate = 4:1)

IR (ATR): ν_{max} (cm^{-1}) = 2927s, 2856s, 1732s, 1462m, 1385w, 1343w, 1253s, 1171s, 1129m, 1091m, 1040w, 724w.

^{1}H NMR (500 MHz, $CDCl_3$) δ 4.12 (t, J = 5.8 Hz, 4H), 2.30 (t, J = 7.3 Hz, 4H), 1.69 – 1.56 (m, 8H), 1.41 – 1.24 (m, 16H).

^{13}C{^{1}H} NMR (126 MHz, $CDCl_3$) δ 173.9, 64.3, 35.1, 29.0, 28.5 (3 close peaks), 25.8, 25.5.

LCMS (ESI$^+$) *m/z* calculated for $C_{18}H_{32}NaO_4^+$ [M + Na]$^+$: 335.22; found: 335.20.

HRMS (ESI$^+$) *m/z* calculated for $C_{18}H_{32}NaO_4^+$ [M + Na]$^+$: 335.2193; found: 335.2198.

1,7-dioxacyclotricosane-8,23-dione (3n)

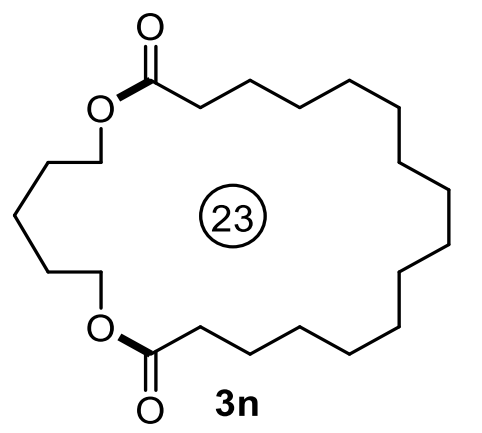

3n

Chemical Formula: $C_{21}H_{38}O_4$
Molecular Weight: 354.53

Following general procedure B, **3n** was obtained from pentane-1,5-diol (10.1 mg, 97.0 µmol), hexadecanedioyl dichloride (79.4 mg, 0.246 mmol), C_{11}-resorcin[4]arene (201 mg, 0.182 mmol, corresponding to 30.3 µmol capsule), and Rb_2CO_3 (60.2 mg). 2.12 µmol loss (2%) of pentane-1,5-diol during transfer process was detected by ^{1}H NMR. Therefore, 94.9 µmol pentane-1,5-diol actually participated in the reaction.

Yield: 14.8 mg, 44%

Property: colorless oil

$\mathbf{R}_f$ 0.56 (*n*-hexane/ethyl acetate = 4:1)

IR (ATR): ν_{max} (cm^{-1}) = 2925s, 2854s, 1734s, 1460m, 1348w, 1244m, 1167m, 1104m, 1071w, 731w.

¹H NMR (500 MHz, $CDCl_3$) δ 4.09 (t, J = 6.2 Hz, 4H), 2.31 (t, J = 7.0 Hz, 4H), 1.72 – 1.59 (m, 8H), 1.51 – 1.42 (m, 2H), 1.38 – 1.21 (m, 20H).

^{13}C{^{1}H} NMR (126 MHz, $CDCl_3$) δ 174.2, 64.1, 34.5, 28.6 (2 close peaks), 28.5 (3 close peaks), 28.3, 24.9, 22.9.

LCMS (ESI$^+$) *m/z* calculated for $C_{21}H_{38}NaO_4^+$ [M + Na]$^+$: 377.27; found: 377.25.

HRMS (ESI$^+$) *m/z* calculated for $C_{21}H_{38}NaO_4^+$ [M + Na]$^+$: 377.2662; found: 377.2669.

1,9-dioxacyclotricosane-2,8-dione (3o)

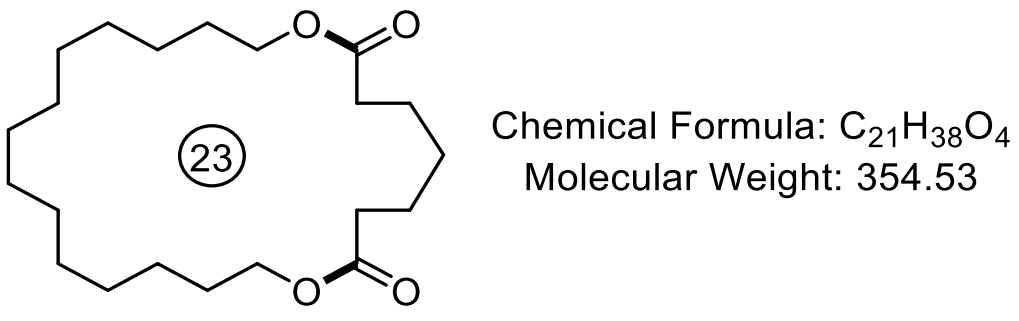

3o

Following general procedure B, **3o** was obtained from tetradecane-1,14-diol (24.1 mg, 105 µmol), heptanedioyl dichloride (prepared from 162 mg heptanedioic acid and 1/4 of the material applied, corresponding to 49.8 mg diacyl chloride, 0.253 mmol), C_{11}-resorcin[4]arene (201 mg, 0.182 mmol, corresponding to 30.3 µmol capsule), and Rb_2CO_3 (59.3 mg). 2.70 µmol loss (3%) of tetradecane-1,14-diol during transfer process was detected by ^{1}H NMR. Therefore, 102 µmol tetradecane-1,14-diol actually participated in the reaction.

Yield: 25.3 mg, 70%

Property: colorless oil

R_f 0.48 (*n*-hexane/ethyl acetate = 4:1)

IR (ATR): ν_{max} (cm^{-1}) = 2924s, 2854s, 1732s, 1460m, 1387w, 1351w, 1250s, 1172s, 1084m, 894w, 723w, 606w, 498w, 422w.

¹H NMR (500 MHz, $CDCl_3$) δ 4.11 (t, J = 5.9 Hz, 4H), 2.31 (t, J = 7.4 Hz, 4H), 1.70 – 1.59 (m, 8H), 1.41 – 1.24 (m, 22H).

^{13}C{^{1}H} NMR (126 MHz, $CDCl_3$) δ 173.8, 64.5, 34.6, 28.9, 28.8 (2 close peaks), 28.5 (2 close peaks), 28.4, 26.0, 24.9.

LCMS (ESI$^+$) *m/z* calculated for $C_{21}H_{38}NaO_4^+$ [M + Na]$^+$: 377.27; found: 377.20.

HRMS (ESI$^+$) *m/z* calculated for $C_{21}H_{39}O_4^+$ [M + H]$^+$: 355.2843; found: 355.2850.

2-((4-chlorophenoxy)methyl)-1,4-dioxacyclodocosane-5,22-dione (3p)

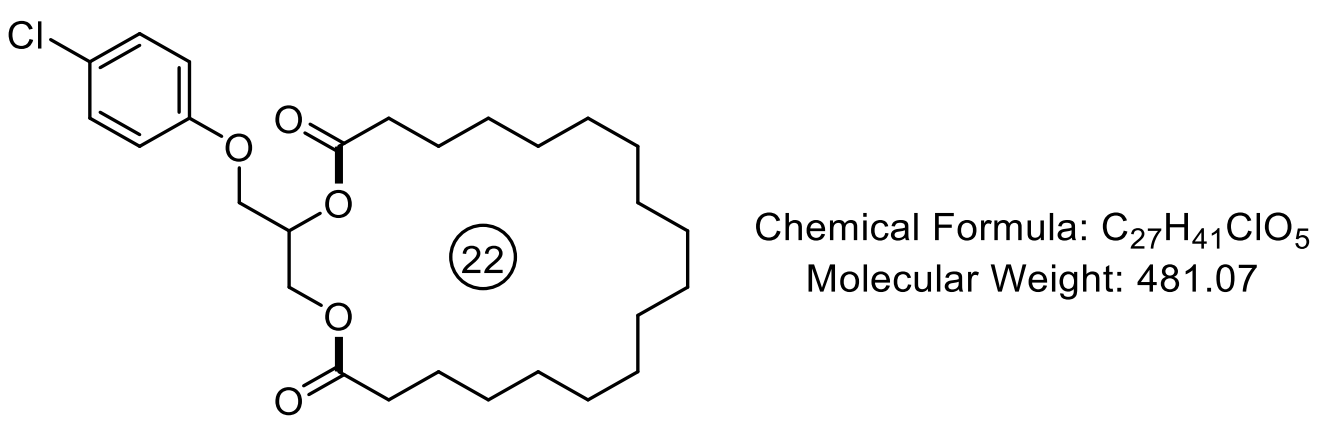

3p

Following general procedure B, **3p** was obtained from chlorphenesin (3-(4-chlorophenoxy)propane-1,2-diol, 20.5 mg, 101 µmol), octadecanedioyl dichloride (88.2 mg, 0.251 mmol), C_{11}-resorcin[4]arene (199 mg, 0.180 mmol, corresponding to 30.0 µmol capsule), and Rb_2CO_3 (60.8 mg) at 90 °C instead of 70 °C. 2.80 µmol loss (3%) of chlorphenesin during transfer process was detected by ^{1}H NMR. Therefore, 98.2 µmol chlorphenesin actually participated in the reaction.

Yield: 14.5 mg, 31%

Property: white solid (melting point: 68–69 °C)

R*f* 0.55 (*n*-hexane/ethyl acetate = 4:1)

IR (ATR): ν_{max} (cm^{-1}) = 2926s, 2855s, 1741s, 1597w, 1583w, 1493s, 1461m, 1283m, 1242s, 1171s, 1096m, 1045w, 1007w, 824m, 675w, 507w.

^{1}H NMR (500 MHz, $CDCl_3$) δ 7.26 – 7.21 (m, 2H), 6.86 – 6.81 (m, 2H), 5.42 – 5.36 (m, 1H), 4.48 (dd, *J* = 12.2, 2.8 Hz, 1H), 4.28 (dd, *J* = 12.2, 6.5 Hz, 1H), 4.13 – 4.03 (m, 2H), 2.37 – 2.29 (m, 4H), 1.70 – 1.58 (m, 4H), 1.39 – 1.22 (m, 24H).

^{13}C{^{1}H} NMR (126 MHz, $CDCl_3$) δ 173.6, 173.2, 157.0, 129.6, 126.5, 116.0, 69.5, 66.6, 62.6, 34.4, 34.3, 28.7 (2 close peaks), 28.4, 28.3 (2 close peaks), 28.2, 28.0, 27.9, 27.4 (2 close peaks), 27.3 (2 close peaks), 25.0, 24.9.

LCMS (ESI$^+$) *m/z* calculated for $C_{27}H_{41}ClNaO_5^+$ [M + Na]$^+$: 503.25; found: 503.20.

HRMS (ESI$^+$) *m/z* calculated for $C_{27}H_{41}ClNaO_5^+$ [M + Na]$^+$: 503.2535; found: 503.2540.

(2*S*,5*S*)-2,5-dimethyl-1,6-dioxacyclotetracosane-7,24-dione (3q)

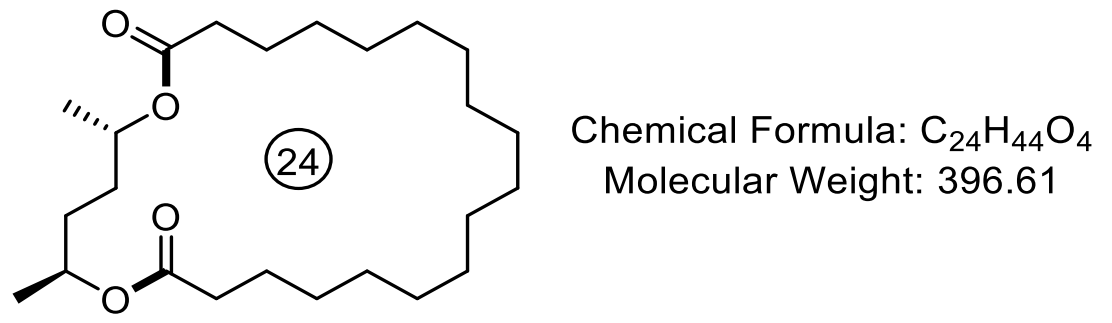

3q

Following general procedure B, **3q** was obtained from (2*S*,5*S*)-hexane-2,5-diol (11.8 mg, 99.8 µmol), octadecanedioyl dichloride (87.5 mg, 0.249 mmol), C_{11}-resorcin[4]arene (200 mg, 0.181 mmol, corresponding to 30.1 µmol capsule), and Rb_2CO_3 (60.6 mg) at 90 °C instead of 70 °C. 2.35 µmol loss (2%) of (2*S*,5*S*)-hexane-2,5-diol during transfer process was detected by ^{1}H NMR. Therefore, 97.4 µmol (2*S*,5*S*)-hexane-2,5-diol actually participated in the reaction.

Yield: 16.1 mg, 42%

Property: colorless oil

R*f* 0.64 (*n*-hexane/ethyl acetate = 4:1)

Optical rotation: $[\alpha]_D^{25}$ = -5.4 deg·cm^3·dm^{-1}·g^{-1} (c 0.72 g/100 mL, $CHCl_3$)

IR (ATR): ν_{max} (cm^{-1}) = 2924s, 2854s, 1730s, 1460m ,1376m, 1245m, 1181m, 1124m, 1104m, 1050m, 1020w, 957w, 853w, 723w, 415w.

^{1}H NMR (500 MHz, $CDCl_3$) δ 4.99 – 4.81 (m, 2H), 2.28 (t, *J* = 7.4 Hz, 4H), 1.70 – 1.55 (m, 6H), 1.52 – 1.44 (m, 2H), 1.35 – 1.24 (m, 24H), 1.21 (d, *J* = 6.2 Hz, 6H).

^{13}C{^{1}H} NMR (126 MHz, $CDCl_3$) δ 173.7, 70.6, 34.8, 32.2, 28.7, 28.5 (3 close peaks), 28.3, 28.2, 25.0, 20.3.

LCMS (ESI$^+$) *m/z* calculated for $C_{24}H_{44}NaO_4^+$ [M + Na]$^+$: 419.31; found: 419.30.

HRMS (ESI$^+$) *m/z* calculated for $C_{24}H_{44}NaO_4^+$ [M + Na]$^+$: 419.3132; found: 419.3141.

2,18-dioxatetracyclo[17.5.1.1^{1,21}.1^{19,23}]heptacosane-3,17-dione (3r)

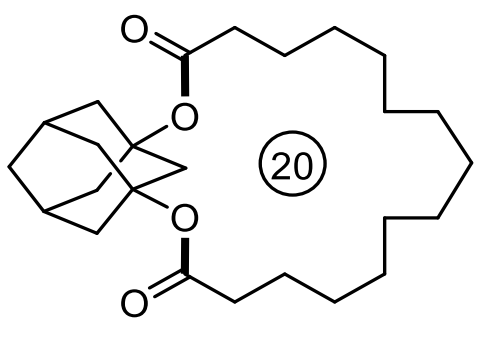

Chemical Formula: $C_{25}H_{40}O_4$
Molecular Weight: 404.59

3r

Following general procedure B, **3r** was obtained from adamantane-1,3-diol (17.0 mg, 101 μmol), pentadecanedioyl diacyl chloride (prepared from 272 mg pentadecanedioic acid and 1/4 of the material applied, corresponding to 77.3 mg diacyl chloride, 0.250 mmol), C_{11}-resorcin[4]arene (203 mg, 0.183 mmol, corresponding to 30.6 μmol capsule), and Rb_2CO_3 (60.7 mg) at 90 °C instead of 70 °C. 6.05 μmol loss (6%) of adamantane-1,3-diol during transfer process was detected by ^{1}H NMR, and more loss than typical cases here was due to the limited solubility of adamantane-1,3-diol in PhCl. Therefore, 95.0 μmol adamantane-1,3-diol actually participated in the reaction.

Yield: 15.9 mg, 41%

Property: colorless oil

R_f 0.68 (*n*-hexane/ethyl acetate = 4:1)

IR (ATR): v_{max} (cm^{-1}) = 2922s, 2855s, 1729s, 1460m, 1351m, 1238s, 1160m, 1103m, 1082m, 1043w, 988m, 730m, 648w, 467w.

^{1}H NMR (500 MHz, $CDCl_3$) δ 2.40 (s, 2H), 2.37 – 2.30 (m, 2H), 2.25 – 2.20 (m, 4H), 2.19 – 2.13 (m, 4H), 2.08 – 2.01 (m, 4H), 1.61 – 1.53 (m, 6H), 1.37 – 1.21 (m, 18H).

^{13}C{^{1}H} NMR (126 MHz, $CDCl_3$) δ 173.0, 80.6, 45.5, 40.2, 35.8, 35.0, 31.4, 29.2, 28.4 (3 close peaks), 27.8, 25.0.

LCMS (ESI$^+$) *m/z* calculated for $C_{25}H_{40}NaO_4^+$ [M + Na]$^+$: 427.28; found: 427.35.

HRMS (ESI$^+$) *m/z* calculated for $C_{25}H_{40}NaO_4^+$ [M + Na]$^+$: 427.2819; found: 427.2814.

1,5-dithiacyclotricosane-6,23-dione (3s)

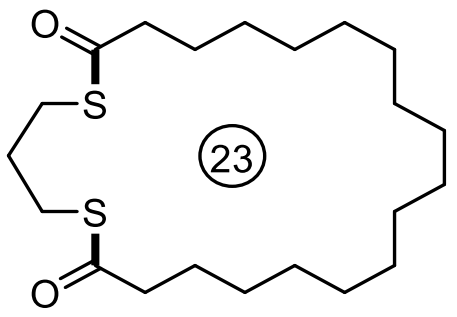

Chemical Formula: $C_{21}H_{38}O_2S_2$
Molecular Weight: 386.65

3s

Following general procedure B, **3s** was obtained from propane-1,3-dithiol (10.0 μL, 10.7 mg, 98.9 μmol), octadecanedioyl dichloride (88.7 mg, 0.252 mmol), C_{11}-resorcin[4]arene (200 mg, 0.181 mmol, corresponding to 30.1 μmol capsule), and Rb_2CO_3 (61.2 mg) at 90 °C instead of 70 °C for 3 h. 2.60 μmol loss (3%) of propane-1,3-dithiol during transfer process was detected by ^{1}H NMR. Therefore, 97.2 μmol propane-1,3-dithiol actually participated in the reaction.

Yield: 10.7 mg, 29%

Property: colorless oil

R$_f$ 0.69 (*n*-hexane/ethyl acetate = 4:1)

IR (ATR): ν_{max} (cm^{-1}) = 2922s, 2853s, 1688s, 1459m, 1444m, 1410w, 1345w, 1298w, 1258w, 1187w, 1120w, 1201m, 951m, 724m, 697m, 599m, 496w, 459w, 428w.

^{1}H NMR (500 MHz, $CDCl_3$) δ 2.93 (t, J = 7.1 Hz, 4H), 2.55 (t, J = 6.8 Hz, 4H), 1.86 (p, J = 7.1 Hz, 2H), 1.68 (p, J = 6.9 Hz, 4H), 1.36 – 1.22 (m, 24H).

^{13}C{^{1}H} NMR (126 MHz, $CDCl_3$) δ 199.7, 44.0, 30.0, 28.9, 28.6 (3 close peaks), 28.4, 28.1, 27.6, 25.7.

LCMS (ESI$^+$) *m/z* calculated for $C_{21}H_{38}NaO_2S_2^+$ [M + Na]$^+$: 409.22; found: 409.25.

HRMS (ESI$^+$) *m/z* calculated for $C_{21}H_{38}NaO_2S_2^+$ [M + Na]$^+$: 409.2205; found: 409.2212.

***cis*-3,20-dioxabicyclo[20.3.1]hexacosane-2,21-dione (3t)**

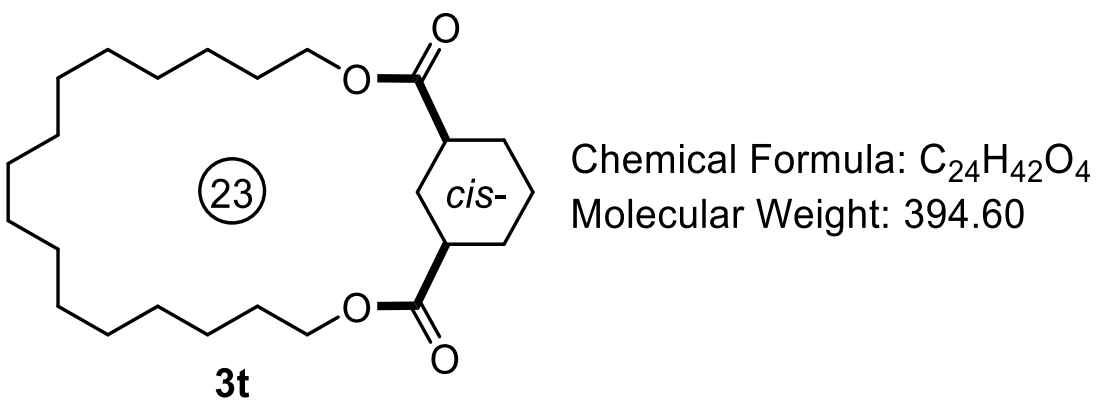

3t

Following general procedure B, **3t** was obtained from hexadecane-1,16-diol (26.0 mg, 100 µmol), *cis*-cyclohexane-1,3-dicarbonyl dichloride (prepared from 172 mg *cis*-cyclohexane-1,3-dicarboxylic acid and 1/4 of the material applied, corresponding to 52.2 mg diacyl chloride, 0.250 mmol), C_{11}-resorcin[4]arene (200 mg, 0.181 mmol, corresponding to 30.1 µmol capsule), and Rb_2CO_3 (61.9 mg). 2.08 µmol loss (2%) of hexadecane-1,16-diol during transfer process was detected by ^{1}H NMR. Therefore, 97.9 µmol hexadecane-1,16-diol actually participated in the reaction.

Yield: 15.5 mg, 40%

Property: colorless oil

R$_f$ 0.64 (*n*-hexane/ethyl acetate = 4:1)

IR (ATR): ν_{max} (cm^{-1}) = 2924s, 2854s, 1731s, 1462m, 1389w, 1370w, 1312m, 1279m, 1190s, 1164m, 1134m, 1031m, 722w, 457w.

^{1}H NMR (500 MHz, $CDCl_3$) δ 4.16 (dt, J = 10.8, 6.1 Hz, 2H), 4.02 (dt, J = 10.8, 5.9 Hz, 2H), 2.33 (tt, J = 12.2, 3.4 Hz, 2H), 2.28 – 2.21 (m, 1H), 2.00 – 1.92 (m, 2H), 1.90 (dq, J = 13.2, 3.1 Hz, 1H), 1.67 – 1.58 (m, 4H), 1.52 (q, J = 12.6 Hz, 1H), 1.44 – 1.20 (m, 27H).

^{13}C{^{1}H} NMR (126 MHz, $CDCl_3$) δ 175.4, 64.6, 43.1, 31.7, 28.8 (2 overlapping peaks + another close peak), 28.6, 28.3 (2 overlapping peaks + another close peak), 25.7, 24.8.

LCMS (ESI$^+$) *m/z* calculated for $C_{24}H_{42}NaO_4^+$ [M + Na]$^+$: 417.30; found: 417.25.

HRMS (ESI$^+$) *m/z* calculated for $C_{24}H_{42}NaO_4^+$ [M + Na]$^+$: 417.2975; found: 417.2977.

3,18-dioxatricyclo[18.2.2.2^{1,20}]hexacosane-2,19-dione (3u)

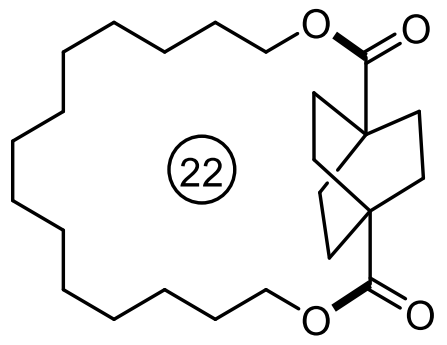

3u

Following general procedure B, **3u** was obtained from tetradecane-1,14-diol (23.2 mg, 101 µmol), bicyclo[2.2.2]octane-1,4-dicarbonyl diacyl chloride (prepared from 199 mg bicyclo[2.2.2]octane-1,4-dicarboxylic acid and 1/4 of the material applied, corresponding to 59.0 mg diacyl chloride, 0.251 mmol), C_{11}-resorcin[4]arene (200 mg, 0.181 mmol, corresponding to 30.1 µmol capsule), and Rb_2CO_3 (61.7 mg). 3.95 µmol loss (4%) of tetradecane-1,14-diol during transfer process was detected by ^{1}H NMR. Therefore, 97.0 µmol tetradecane-1,14-diol actually participated in the reaction.

Yield: 25.9 mg, 68%

Property: colorless oil

R*f* 0.52 (*n*-hexane/ethyl acetate = 4:1)

IR (ATR): ν_{max} (cm^{-1}) = 2920s, 2851s, 1725s, 1457m, 1352w, 1249s, 1214s, 1171m, 1073s, 1000m, 856w, 825w, 741m, 471w.

^{1}H NMR (500 MHz, $CDCl_3$) δ 4.10 – 4.04 (m, 4H), 1.83 (s, 12H), 1.66 – 1.58 (m, 4H), 1.51 – 1.38 (m, 8H), 1.37 – 1.24 (m, 12H).

^{13}C{^{1}H} NMR (126 MHz, $CDCl_3$) δ 177.3, 65.1, 38.9, 30.8, 29.8, 29.3, 28.8, 28.0, 27.6, 26.4.

LCMS (ESI$^+$) *m/z* calculated for $C_{24}H_{40}NaO_4^+$ [M + Na]$^+$: 415.28; found: 415.45.

HRMS (ESI$^+$) *m/z* calculated for $C_{24}H_{40}NaO_4^+$ [M + Na]$^+$: 415.2819; found: 415.2816.

3,12-dioxatetracyclo[12.5.1.1^{1,16}.1^{14,18}]docosane-2,13-dione (3v)

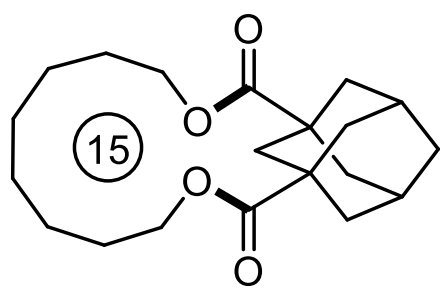

3v

Following general procedure B, **3v** was obtained from octane-1,8-diol (14.8 mg, 101 µmol), adamantane-1,3-dicarbonyl diacyl chloride (prepared from 224 mg adamantane-1,3-dicarboxylic acid and 1/4 of the material applied, corresponding to 65.3 mg diacyl chloride, 0.250 mmol), C_{11}-resorcin[4]arene (201 mg, 0.182 mmol, corresponding to 30.3 µmol capsule), and Rb_2CO_3 (61.0 mg). 1.68 µmol loss (2%) of octane-1,8-diol during transfer process was detected by ^{1}H NMR. Therefore, 99.3 µmol octane-1,8-diol actually participated in the reaction.

Yield: 13.8 mg, 42%

Property: colorless oil

R*f* 0.60 (*n*-hexane/ethyl acetate = 4:1)

IR (ATR): ν_{max} (cm^{-1}) = 2911s, 2857s, 1728s, 1454m, 1372w, 1280s, 1246s, 1219m, 1092s, 1016w, 953w, 695w, 521w.

^{1}H NMR (500 MHz, $CDCl_3$) δ 4.12 (t, J = 5.0 Hz, 4H), 2.18 (p, J = 3.2 Hz, 2H), 2.13 (br, 2H), 1.94 – 1.78 (m, 8H), 1.68 (t, J = 3.1 Hz, 2H), 1.61 – 1.52 (m, 4H), 1.49 – 1.40 (m, 4H), 1.39 – 1.32 (m, 4H).

^{13}C{^{1}H} NMR (126 MHz, $CDCl_3$) δ 176.8, 64.2, 43.1, 41.4, 37.5, 35.6, 28.8, 28.1, 27.9, 24.8.

LCMS (ESI$^+$) *m/z* calculated for $C_{20}H_{30}NaO_4^+$ [M + Na]$^+$: 357.20; found: 357.35.

HRMS (ESI$^+$) *m/z* calculated for $C_{20}H_{30}NaO_4^+$ [M + Na]$^+$: 357.2036; found: 357.2037.

3,14-dioxatetracyclo[14.5.1.1^{1,18}.1^{16,20}]tetracosane-2,15-dione (3w)

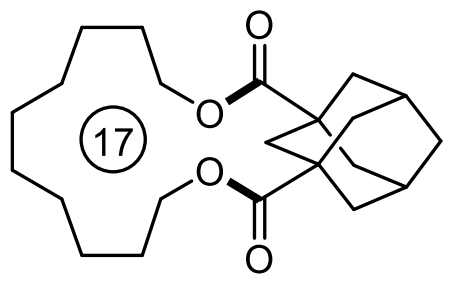

Chemical Formula: $C_{22}H_{34}O_4$
Molecular Weight: 362.51

3w

Following general procedure B, **3w** was obtained from decane-1,10-diol (17.6 mg, 101 µmol), adamantane-1,3-dicarbonyl diacyl chloride (prepared from 225 mg adamantane-1,3-dicarboxylic acid and 1/4 of the material applied, corresponding to 65.5 mg diacyl chloride, 0.251 mmol), C_{11}-resorcin[4]arene (200 mg, 0.181 mmol, corresponding to 30.1 µmol capsule), and Rb_2CO_3 (59.0 mg). 3.45 µmol loss (3%) of decane-1,10-diol during transfer process was detected by ^{1}H NMR. Therefore, 97.6 µmol decane-1,10-diol actually participated in the reaction.

Yield: 23.3 mg, 66%

Property: colorless oil

R_f 0.55 (*n*-hexane/ethyl acetate = 4:1)

IR (ATR): ν_{max} (cm^{-1}) = 2925s, 2090s, 2856s, 1721s, 1454m, 1372w, 1344w, 1319w, 1277s, 1247s, 1216m, 1203m, 1174w, 1091s, 1035w, 957w, 918w, 734m, 695m, 574w, 470w.

^{1}H NMR (500 MHz, $CDCl_3$) δ 4.10 (t, J = 5.3 Hz, 4H), 2.16 (p, J = 3.0 Hz, 2H), 2.05 (br, 2H), 1.93 – 1.81 (m, 8H), 1.67 (t, J = 3.5 Hz, 2H), 1.61 – 1.58 (m, 4H), 1.44 – 1.36 (m, 4H), 1.35 – 1.28 (m, 8H).

^{13}C{^{1}H} NMR (126 MHz, $CDCl_3$) δ 177.0, 64.5, 41.2, 40.5, 38.1, 35.4, 28.1 (2 close peaks), 28.0, 27.4, 26.2.

LCMS (ESI$^+$) *m/z* calculated for $C_{22}H_{34}NaO_4^+$ [M + Na]$^+$: 385.23; found: 385.30.

HRMS (ESI$^+$) *m/z* calculated for $C_{22}H_{34}NaO_4^+$ [M + Na]$^+$: 385.2349; found: 385.2348.

1^5-bromo-3,16-dioxa-1(1,3)-benzenacycloheptadecaphane-2,17-dione (3x)

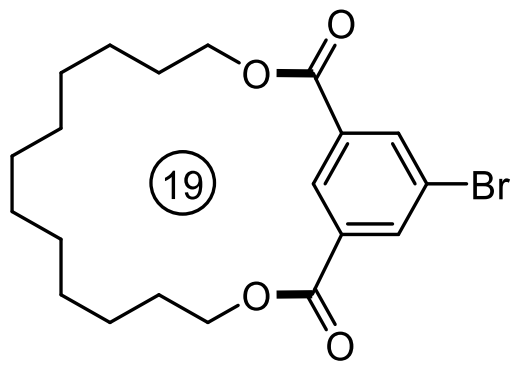

Chemical Formula: $C_{20}H_{27}BrO_4$
Molecular Weight: 411.34

3x

Following general procedure B, **3x** was obtained from dodecane-1,12-diol (20.3 mg, 100 µmol), 5-bromoisophthaloyl diacyl chloride (prepared from 245 mg 5-bromoisophthalic acid and 1/4 of the material applied, corresponding to 70.5 mg diacyl chloride, 0.250 mmol), C_{11}-resorcin[4]arene (200 mg, 0.181 mmol, corresponding to 30.1 µmol capsule), and Rb_2CO_3 (60.5 mg) at 90 °C instead of 70 °C.

3.15 μmol loss (3%) of dodecane-1,12-diol during transfer process was detected by ^{1}H NMR. Therefore, 96.8 μmol dodecane-1,12-diol actually participated in the reaction.

Yield: 19.0 mg, 48%

Property: white solid (melting point: 98–99°C)

R*f* 0.76 (*n*-hexane/ethyl acetate = 4:1)

IR (ATR): ν_{max} (cm^{-1}) = 3076w, 2928s, 2856s, 1710s, 1458m, 1387m, 1301s, 1227s, 1143s, 1102w, 1054m, 990w, 926w, 906m, 859w, 751s, 727m, 663w, 495w.

^{1}H NMR (500 MHz, $CDCl_3$) δ 8.45 (t, J = 1.5 Hz, 1H), 8.40 (d, J = 1.5 Hz, 2H), 4.39 (t, J = 5.5 Hz, 4H), 1.82 – 1.73 (m, 4H), 1.56 – 1.47 (m, 4H), 1.46 – 1.31 (m, 12H).

^{13}C{^{1}H} NMR (126 MHz, $CDCl_3$) δ 164.9, 137.2, 132.8, 127.9, 123.0, 66.2, 28.9, 28.5, 27.9, 27.3, 26.2.

LCMS (ESI$^+$) *m/z* calculated for $C_{20}H_{27}BrNaO_4^+$ [M + Na]$^+$: 433.10; found: 433.15.

HRMS (ESI$^+$) *m/z* calculated for $C_{20}H_{27}BrNaO_4^+$ [M + Na]$^+$: 433.0985; found: 433.0980.

Single crystals of **3x** were obtained by crystallization in $CDCl_3$/DCM/*n*-hexane. Vapor diffusion was employed for crystallization. Approximately 15.0 mg of the sample was dissolved in a mixture of 0.40 mL $CDCl_3$ and 0.10 mL DCM in a 2 mL GC vial. The small vial was placed inside a 20 mL vial containing 3.0 mL *n*-hexane, then the big vial was capped, allowing slow vapor diffusion to occur. X-Ray diffraction confirmed its chemical structure (see Appendix A for details).

3,20-dioxa-1(1,4)-benzenacyclohenicosaphane-2,21-dione (3y)

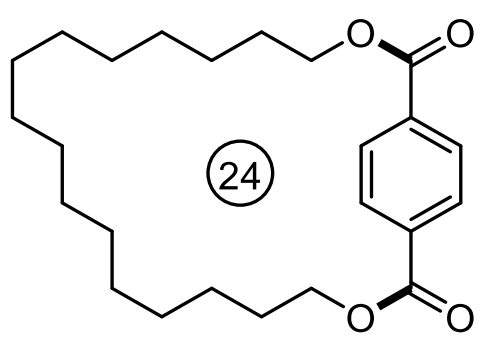

3y

Chemical Formula: $C_{24}H_{36}O_4$
Molecular Weight: 388.55

Following general procedure B, **3y** was obtained from hexadecane-1,16-diol (26.0 mg, 100 μmol), terephthaloyl dichloride (51.0 mg, 0.251 mmol), C_{11}-resorcin[4]arene (200 mg, 0.181 mmol, corresponding to 30.1 μmol capsule), and Rb_2CO_3 (60.9 mg) at 90 °C instead of 70 °C. 2.65 μmol loss (3%) of hexadecane-1,16-diol during transfer process was detected by ^{1}H NMR. Therefore, 97.4 μmol hexadecane-1,16-diol actually participated in the reaction.

Yield: 18.5 mg, 49%

Property: white solid (melting point: 88–89 °C)

R*f* 0.72 (*n*-hexane/ethyl acetate = 4:1)

IR (ATR): ν_{max} (cm^{-1}) = 2924s, 2853s, 1717s, 1578w, 1460w ,1409w, 1377w, 1270s, 1116s, 1103s, 1035w, 1019m, 911w, 874w, 728s, 498w.

^{1}H NMR (500 MHz, $CDCl_3$) δ 8.10 (s, 4H), 4.43 – 4.35 (m, 4H), 1.81 – 1.71 (m, 4H), 1.57 – 1.49 (m, 4H), 1.47 – 1.40 (m, 4H), 1.40 – 1.33 (m, 4H), 1.33 – 1.21 (m, 12H).

^{13}C{^{1}H} NMR (126 MHz, $CDCl_3$) δ 165.9, 134.5, 129.6, 65.6, 30.2, 29.9, 28.6, 28.5, 28.0, 27.9, 25.4.

LCMS (ESI$^+$) *m/z* calculated for $C_{25}H_{40}NaO_5^+$ [M + Na + CH_3OH]$^+$: 443.28; found: 443.20.

HRMS (ESI$^+$) *m/z* calculated for $C_{24}H_{36}NaO_4^+$ [M + Na]$^+$: 411.2506; found: 411.2500.

Single crystals of **3y** were obtained by crystallization in $CDCl_3$/DCM/*n*-hexane . Vapor diffusion was employed for crystallization. Approximately 15.0 mg of the sample was dissolved in a mixture of 0.40 mL $CDCl_3$ and 0.10 mL DCM in a 2 mL GC vial. The small vial was placed inside a 20 mL vial containing 3.0 mL *n*-hexane, then the big vial was capped, allowing slow vapor diffusion to occur. X-Ray diffraction confirmed its chemical structure (see Appendix A for details).

3,26-dioxatricyclo[26.1.1.1^{1,28}]hentriacontane-2,27-dione (3z)

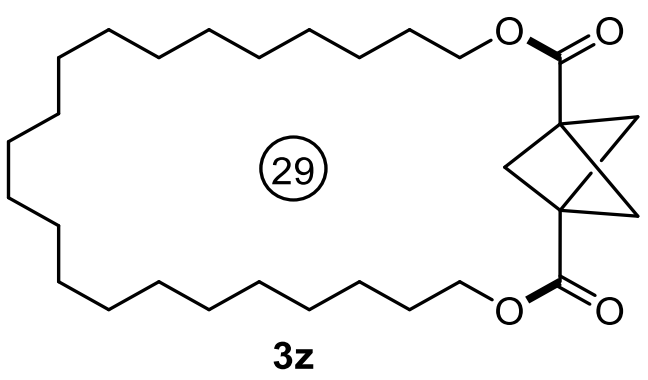

Chemical Formula: $C_{29}H_{50}O_4$
Molecular Weight: 462.72

Following general procedure B, **3z** was obtained from docosane-1,22-diol (34.0 mg, 99.2 μmol), bicyclo[1.1.1]pentane-1,3-dicarbonyl diacyl chloride (prepared from 157 mg bicyclo[1.1.1]pentane-1,3-dicarboxylic acid and 1/4 of the material applied, corresponding to 48.4 mg diacyl chloride, 0.251 mmol), C_{11}-resorcin[4]arene (200 mg, 0.181 mmol, corresponding to 30.1 μmol capsule), and Rb_2CO_3 (60.3 mg). 1.90 μmol loss (2%) of docosane-1,22-diol during transfer process was detected by ^{1}H NMR. Therefore, 97.3 μmol docosane-1,22-diol actually participated in the reaction.

Yield: 14.0 mg, 31%

Property: white solid (melting point: 54–55 °C)

R*f* 0.62 (*n*-hexane/ethyl acetate = 4:1)

IR (ATR): ν_{max} (cm^{-1}) = 2922s, 2852s, 1732s, 1461m, 1387w, 1344m, 1285s, 1203s, 1165m, 1142m, 1047m, 723w, 458w.

^{1}H NMR (500 MHz, $CDCl_3$) δ 4.09 (t, *J* = 6.0 Hz, 4H), 2.32 (s, 6H), 1.65 – 1.58 (4H, m), 1.39 – 1.24 (m, 36H).

^{13}C{^{1}H} NMR (126 MHz, $CDCl_3$) δ 169.6, 65.0, 52.9, 38.0, 29.4 (2 close peaks), 29.3 (2 close peaks), 29.2 (2 close peaks), 29.1, 29.0, 28.6, 26.1.

LCMS (ESI$^+$) *m/z* calculated for $C_{29}H_{50}NaO_4^+$ [M + Na]$^+$: 485.36; found: 485.25.

HRMS (ESI$^+$) *m/z* calculated for $C_{29}H_{50}NaO_4^+$ [M + Na]$^+$: 485.3601; found: 485.3594.

3,22-dioxatricyclo[22.1.1.1^{1,24}]heptacosane-2,23-dione (3aa)

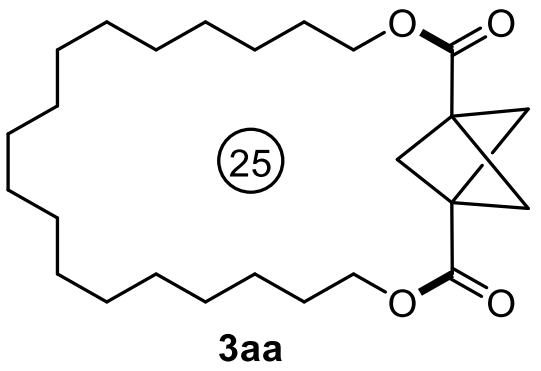

Chemical Formula: $C_{25}H_{42}O_4$
Molecular Weight: 406.61

Following general procedure B, **3aa** was obtained from octadecane-1,18-diol (28.8 mg, 101 μmol), bicyclo[1.1.1]pentane-1,3-dicarbonyl diacyl chloride (prepared from 157 mg bicyclo[1.1.1]pentane-1,3-dicarboxylic acid and 1/4 of the material applied, corresponding to 48.4 mg diacyl chloride, 0.251 mmol), C_{11}-resorcin[4]arene (201 mg, 0.182 mmol, corresponding to 30.3 μmol capsule), and Rb_2CO_3

(62.1 mg). 2.42 µmol loss (2%) of octadecane-1,18-diol during transfer process was detected by ^{1}H NMR. Therefore, 98.6 µmol octadecane-1,18-diol actually participated in the reaction.

Yield: 33.6 mg, 84%

Property: white solid (melting point: 58–59 °C)

R*f* 0.55 (*n*-hexane/ethyl acetate = 4:1)

IR (ATR): ν_{max} (cm^{-1}) = 2922s, 2852s, 1730s, 1461m, 1386w, 1343m, 1284s, 1203s, 1167s, 1141m, 1045s, 841w, 779w, 731m, 466w.

^{1}H NMR (500 MHz, $CDCl_3$) δ 4.09 (t, J = 5.6 Hz, 4H), 2.31 (s, 6H), 1.64 – 1.58 (m, 4H), 1.39 – 1.25 (m, 28H).

^{13}C{^{1}H} NMR (126 MHz, $CDCl_3$) δ 169.5, 64.8, 52.8, 37.9, 29.2, 29.0, 28.8, 28.7, 28.6, 28.4 (2 close peaks), 25.8.

LCMS (ESI$^+$) *m/z* calculated for $C_{25}H_{42}NaO_4^+$ [M + Na]$^+$: 429.30; found: 429.25.

HRMS (ESI$^+$) *m/z* calculated for $C_{25}H_{42}NaO_4^+$ [M + Na]$^+$: 429.2975; found: 429.2979.

Additional reaction (i), applying toluene instead of chlorobenzene as the solvent: following general procedure B, **3aa** was obtained from octadecane-1,18-diol (28.6 mg, 99.8 µmol), bicyclo[1.1.1]pentane-1,3-dicarbonyl diacyl chloride (48.4 mg, 0.251 mmol), C_{11}-resorcin[4]arene (199 mg, 0.180 mmol, corresponding to 30.0 µmol capsule), and Rb_2CO_3 (60.3 mg) in toluene (2.0 mL in total). 3.45 µmol loss (3%) of octadecane-1,18-diol during transfer process was detected by ^{1}H NMR. Therefore, 96.4 µmol octadecane-1,18-diol actually participated in the reaction. **Yield:** 32.6 mg, 83%.

Additional reaction (ii), applying 15 mol % capsule instead of 30 mol %: following general procedure B, **3aa** was obtained from octadecane-1,18-diol (28.9 mg, 101 µmol), bicyclo[1.1.1]pentane-1,3-dicarbonyl diacyl chloride (48.2 mg, 0.250 mmol), C_{11}-resorcin[4]arene (102 mg, 92.2 µmol, corresponding to 15.4 µmol capsule), and Rb_2CO_3 (60.2 mg) in PhCl (2.0 mL in total). 4.52 µmol loss (4%) of octadecane-1,18-diol during transfer process was detected by ^{1}H NMR. Therefore, 96.5 µmol octadecane-1,18-diol actually participated in the reaction. **Yield:** 26.3 mg, 67%.

3,18-dioxatricyclo[18.1.1.1^{1,20}]tricosane-2,19-dione (3ab)

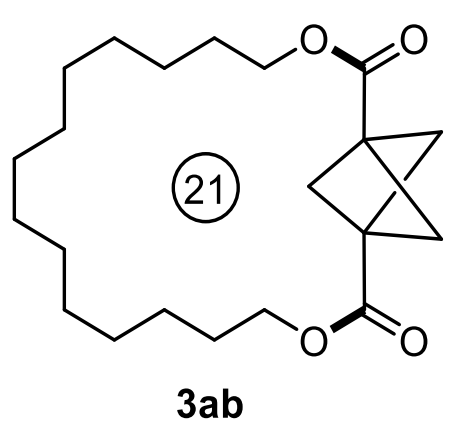

Chemical Formula: $C_{21}H_{34}O_4$
Molecular Weight: 350.50

3ab

Following general procedure B, **3ab** was obtained from tetradecane-1,14-diol (23.2 mg, 101 µmol), bicyclo[1.1.1]pentane-1,3-dicarbonyl diacyl chloride (prepared from 157 mg bicyclo[1.1.1]pentane-1,3-dicarboxylic acid and 1/4 of the material applied, corresponding to 48.4 mg diacyl chloride, 0.251 mmol), C_{11}-resorcin[4]arene (200 mg, 0.181 mmol, corresponding to 30.1 µmol capsule), and Rb_2CO_3 (61.1 mg). 2.82 µmol loss (3%) of tetradecane-1,14-diol during transfer process was detected by ^{1}H NMR. Therefore, 98.2 µmol tetradecane-1,14-diol actually participated in the reaction.

Yield: 24.7 mg, 72%

Property: white solid (melting point: 85–86 °C)

R_f 0.50 (*n*-hexane/ethyl acetate = 4:1)

IR (ATR): ν_{max} (cm^{-1}) = 2924s, 2853s, 1721s, 1461m, 1389w, 1348w, 1287s, 1206s, 1167m, 1146m, 1042m, 1008w, 864m, 802w, 773w, 731m, 547w, 457m.

^{1}H NMR (500 MHz, $CDCl_3$) δ 4.15 – 4.10 (m, 4H), 2.31 (s, 6H), 1.62 (p, *J* = 6.6 Hz, 4H), 1.44 –1.23 (m, 20H).

^{13}C{^{1}H} NMR (126 MHz, $CDCl_3$) δ 169.4, 65.1, 52.9, 38.0, 30.0, 29.1, 28.8, 28.4, 27.8, 25.9.

LCMS (ESI$^+$) *m/z* calculated for $C_{21}H_{34}NaO_4^+$ [M + Na]$^+$: 373.23; found: 373.15.

HRMS (ESI$^+$) *m/z* calculated for $C_{21}H_{34}NaO_4^+$ [M + Na]$^+$: 373.2349; found: 373.2352.

An additional reaction applying more concentrated condition (0.1 M for tetradecane-1,14-diol): following general procedure B, **3ab** was obtained from tetradecane-1,14-diol (23.5 mg, 102 μmol, dissolved in 0.60 mL PhCl under 70 °C), bicyclo[1.1.1]pentane-1,3-dicarbonyl diacyl chloride (prepared from 157 mg bicyclo[1.1.1]pentane-1,3-dicarboxylic acid, dissolved in 1.6 mL PhCl and 0.40 mL of the solution applied, corresponding to 48.4 mg diacyl chloride, 0.251 mmol), C_{11}-resorcin[4]arene (201 mg, 0.182 mmol, corresponding to 30.3 μmol capsule), and Rb_2CO_3 (62.1 mg). 7.20 μmol loss (7%) of tetradecane-1,14-diol during transfer process was detected by ^{1}H NMR, and more loss compared with standard conditions was due to the limited solubility of tetradecane-1,14-diol in less amount of PhCl here. Therefore, 94.8 μmol tetradecane-1,14-diol actually participated in the reaction. **Yield:** 19.6 mg, 59%.

Single crystals of **3ab** were obtained by crystallization in $CDCl_3$/DCM/*n*-hexane. Vapor diffusion was employed for crystallization. Approximately 20.0 mg of the sample was dissolved in a mixture of 0.40 mL $CDCl_3$ and 0.10 mL DCM in a 2 mL GC vial. The small vial was placed inside a 20 mL vial containing 3.0 mL *n*-hexane, then the big vial was capped, allowing slow vapor diffusion to occur. X-Ray diffraction confirmed its chemical structure (see Appendix A for details).

3,16-dioxatricyclo[16.1.1.1^{1,18}]henicosane-2,17-dione (3ac)

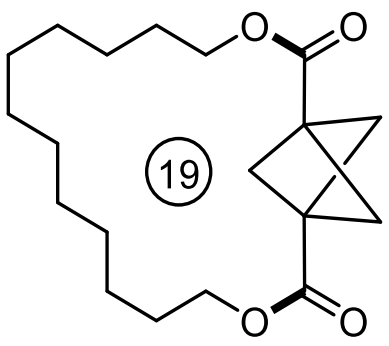

Chemical Formula: $C_{19}H_{30}O_4$
Molecular Weight: 322.45

3ac

Following general procedure B, **3ac** was obtained from dodecane-1,12-diol (20.8 mg, 103 μmol), bicyclo[1.1.1]pentane-1,3-dicarbonyl diacyl chloride (prepared from 157 mg bicyclo[1.1.1]pentane-1,3-dicarboxylic acid and 1/4 of the material applied, corresponding to 48.4 mg diacyl chloride, 0.251 mmol), C_{11}-resorcin[4]arene (200 mg, 0.181 mmol, corresponding to 30.1 μmol capsule), and Rb_2CO_3 (62.1 mg). 3.18 μmol loss (3%) of dodecane-1,12-diol during transfer process was detected by ^{1}H NMR. Therefore, 99.8 μmol dodecane-1,12-diol actually participated in the reaction.

Yield: 19.3 mg, 60%

Property: white solid (melting point: 93–94 °C)

R_f 0.57 (*n*-hexane/ethyl acetate = 4:1)

IR (ATR): ν_{max} (cm^{-1}) = 2923s, 2853s, 1733s, 1461m, 1393w, 1340s, 1285s, 1207s, 1182s, 1145w, 1113m, 1052m, 959m, 783w, 734w, 468w.

^{1}**H NMR** (500 MHz, $CDCl_3$) δ 4.16 – 4.08 (m, 4H), 2.33 (s, 6H), 1.69 – 1.62 (m, 4H), 1.48 – 1.37 (m, 8H), 1.36 –1.26 (m, 8H).

^{13}**C{^{1}H} NMR** (126 MHz, $CDCl_3$) δ 169.1, 65.9, 52.9, 38.2, 29.9 (2 close peaks), 29.7, 27.7, 27.6.

LCMS (ESI$^+$) *m/z* calculated for $C_{20}H_{34}NaO_5^+$ [M + Na + CH_3OH]$^+$: 377.23; found: 377.20.

HRMS (ESI$^+$) *m/z* calculated for $C_{19}H_{31}O_4^+$ [M + H]$^+$: 323.2217; found: 323.2220.

3,14-dioxatricyclo[14.1.1.1^{1,16}]nonadecane-2,15-dione (3ad)

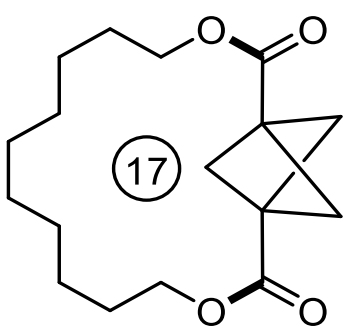

Chemical Formula: $C_{17}H_{26}O_4$
Molecular Weight: 294.39

3ad

Following general procedure B, **3ad** was obtained from decane-1,10-diol (17.5 mg, 100 µmol), bicyclo[1.1.1]pentane-1,3-dicarbonyl diacyl chloride (prepared from 157 mg bicyclo[1.1.1]pentane-1,3-dicarboxylic acid and 1/4 of the material applied, corresponding to 48.4 mg diacyl chloride, 0.251 mmol), C_{11}-resorcin[4]arene (201 mg, 0.182 mmol, corresponding to 30.3 µmol capsule), and Rb_2CO_3 (62.2 mg). 3.62 µmol loss (4%) of decane-1,10-diol during transfer process was detected by ^{1}H NMR. Therefore, 96.4 µmol decane-1,10-diol actually participated in the reaction.

Yield: 6.8 mg, 24%

Property: white solid (melting point: 104–105 °C)

$\mathbf{R}_f$ 0.54 (*n*-hexane/ethyl acetate = 4:1)

IR (ATR): ν_{max} (cm^{-1}) = 2972m, 2925s, 2854s, 1739s, 1463m, 1435w, 1390w, 1279m, 1206s, 1184s, 1146m, 1137m, 1116m, 1052m, 960m, 789w, 736w, 591w, 424w.

^{1}**H NMR** (500 MHz, $CDCl_3$) δ 4.12 – 4.07 (m, 4H), 2.39 (s, 6H), 1.70 – 1.63 (m, 4H), 1.59 – 1.51 (m, 4H), 1.49 –1.42 (m, 4H), 1.39 –1.31 (m, 4H).

^{13}**C{^{1}H} NMR** (126 MHz, $CDCl_3$) δ 168.8, 66.4, 52.7, 38.9, 31.8, 30.7, 28.7, 28.2.

LCMS (ESI$^+$) *m/z* calculated for $C_{18}H_{30}NaO_5^+$ [M + Na + CH_3OH]$^+$: 349.20; found: 349.10.

HRMS (ESI$^+$) *m/z* calculated for $C_{18}H_{30}NaO_5^+$ [M + Na + CH_3OH]$^+$: 349.1985; found: 349.1993.

Single crystals of **3ad** were obtained by crystallization in $CDCl_3$/DCM/*n*-hexane. Vapor diffusion was employed for crystallization. Approximately 10.0 mg of the sample was dissolved in a mixture of 0.40 mL $CDCl_3$ and 0.10 mL DCM in a 2 mL GC vial. The small vial was placed inside a 20 mL vial containing 3.0 mL *n*-hexane, then the big vial was capped, allowing slow vapor diffusion to occur. X-Ray diffraction confirmed its chemical structure (see Appendix A for details).

3-(benzyloxy)-1,5-dioxacyclohenicosane-6,21-dione (3ae)

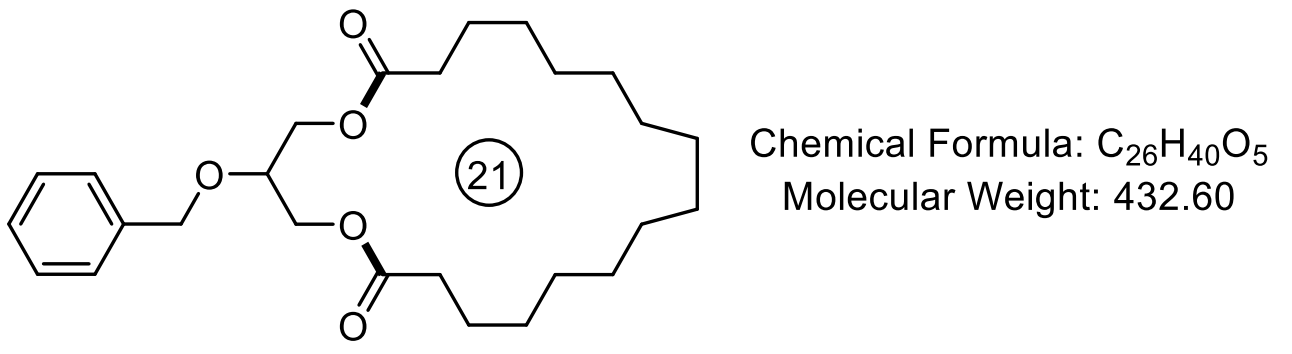

3ae

Following general procedure B, **3ae** was obtained from commercially available 2-(benzyloxy)propane-1,3-diol (18.3 mg, 100 µmol), hexadecanedioyl dichloride (82.5 mg, 0.255 mmol), C_{11}-resorcin[4]arene (202 mg, 0.183 mmol, corresponding to 30.4 µmol capsule), and Rb_2CO_3 (61.5 mg). 4.60 µmol loss (5%) of 2-(benzyloxy)propane-1,3-diol during transfer process was detected by ^{1}H NMR. Therefore, 95.4 µmol 2-(benzyloxy)propane-1,3-diol actually participated in the reaction.

Yield: 12.3 mg, 30%

Property: colorless oil

R_f 0.48 (*n*-hexane/ethyl acetate = 4:1)

IR (ATR): v_{max} (cm^{-1}) = 2926s, 2855s, 1737s, 1455m, 1348w, 1163m, 1106m, 1053m, 737m, 698m.

^{1}H NMR (500 MHz, $CDCl_3$) δ 7.38 – 7.27 (m, 5H), 4.66 (s, 2H), 4.26 (dd, J = 11.6, 5.1 Hz, 2H), 4.19 (dd, J = 11.6, 5.3 Hz, 2H), 3.80 (p, J = 5.2 Hz, 1H), 2.33 (td, J = 6.8, 2.9 Hz, 4H), 1.63 (p, J = 7.0 Hz, 4H), 1.42 – 1.21 (m, 20H).

^{13}C{^{1}H} NMR (126 MHz, $CDCl_3$) δ 173.7, 137.9, 128.6, 128.0 (2 close peaks), 74.6, 72.2, 63.0, 34.2, 28.3 (2 close peaks), 28.2, 28.1, 27.8, 24.8.

LCMS (ESI$^+$) *m/z* calculated for $C_{26}H_{40}NaO_5^+$ [M + Na]$^+$: 455.28; found: 455.30.

HRMS (ESI$^+$) *m/z* calculated for $C_{26}H_{40}NaO_5^+$ [M + Na]$^+$: 455.2768; found: 455.2769.

1,8,15,22-tetraoxacyclooctacosane-2,7,16,21-tetraone (4a)

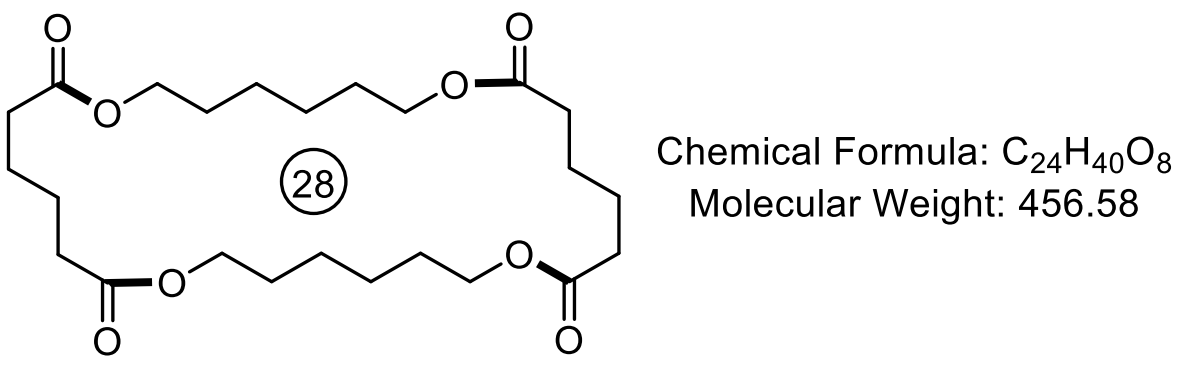

4a

Following general procedure B, **4a** was obtained from hexane-1,6-diol (11.9 mg, 101 µmol), adipoyl dichloride (prepared from 147 mg adipic acid and 1/4 of the material applied, corresponding to 46.0 mg diacyl chloride, 0.251 mmol), C_{11}-resorcin[4]arene (200 mg, 0.181 mmol, corresponding to 30.1 µmol capsule), and Rb_2CO_3 (60.2 mg). 2.50 µmol loss (2%) of hexane-1,6-diol during transfer process was detected by ^{1}H NMR. Therefore, 98.5 µmol hexane-1,6-diol actually participated in the reaction.

Yield: 8.7 mg, 39%

Property: colorless oil

R_f 0.12 (*n*-hexane/ethyl acetate = 4:1)

IR (ATR): v_{max} (cm^{-1}) = 2936m, 2863m, 1730s, 1460w, 1390w, 1350w, 1239m, 1174m, 1142m, 1078w, 988w, 756w.

^{1}H NMR (500 MHz, $CDCl_3$) δ 4.08 (t, J = 6.4 Hz, 8H), 2.37 – 2.27 (m, 8H), 1.74 – 1.66 (m, 16H), 1.44 – 1.35 (m, 8H).

^{13}C{^{1}H} NMR (126 MHz, $CDCl_3$) δ 173.5, 64.4, 34.2, 28.6, 25.8, 24.6.

LCMS (ESI$^+$) *m/z* calculated for $C_{24}H_{40}NaO_8^+$ [M + Na]$^+$: 479.26; found: 479.30.

HRMS (ESI$^+$) *m/z* calculated for $C_{24}H_{40}NaO_8^+$ [M + Na]$^+$: 479.2615; found: 479.2624.

1,8,17,24-tetraoxacyclodotriacontane-2,7,18,23-tetraone (4b)

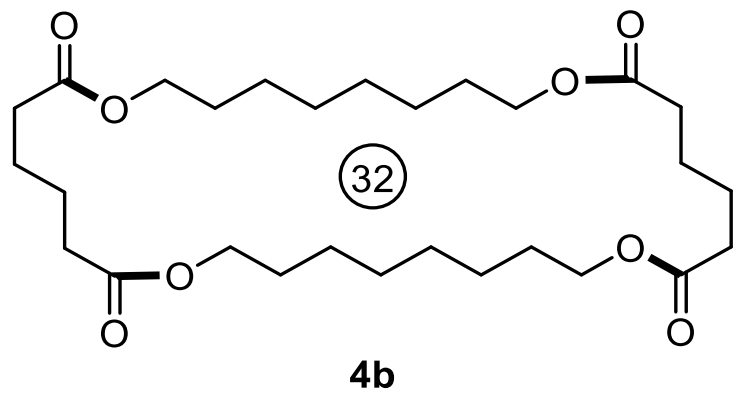

Chemical Formula: $C_{28}H_{48}O_8$
Molecular Weight: 512.68

4b

Following general procedure B, **4b** was obtained from octane-1,8-diol (14.7 mg, 100 µmol), adipoyl dichloride (prepared from 147 mg adipic acid and 1/4 of the material applied, corresponding to 46.0 mg diacyl chloride, 0.251 mmol), C_{11}-resorcin[4]arene (201 mg, 0.182 mmol, corresponding to 30.3 µmol capsule), and Rb_2CO_3 (62.1 mg). 3.88 µmol loss (4%) of octane-1,8-diol during transfer process was detected by ^{1}H NMR. Therefore, 96.1 µmol octane-1,8-diol actually participated in the reaction.

Yield: 8.2 mg, 33%

Property: colorless oil

R_f 0.20 (*n*-hexane/ethyl acetate = 4:1)

IR (ATR): ν_{max} (cm^{-1}) = 2931m, 2857m, 1731s, 1460w, 1390w, 1350w, 1240m, 1172m, 1142m, 1077w, 725w.

^{1}H NMR (500 MHz, $CDCl_3$) δ 4.07 (t, J = 6.5 Hz, 8H), 2.38 – 2.28 (m, 8H), 1.71 – 1.65 (m, 8H), 1.65 – 1.58 (m, 8H), 1.40 – 1.29 (m, 16H).

^{13}C{^{1}H} NMR (126 MHz, $CDCl_3$) δ 173.5, 64.5, 34.2, 29.2, 28.8, 26.0, 24.6.

LCMS (ESI$^+$) *m/z* calculated for $C_{28}H_{48}NaO_8^+$ [M + Na]$^+$: 535.32; found: 535.30.

HRMS (ESI$^+$) *m/z* calculated for $C_{28}H_{49}O_8^+$ [M + H]$^+$: 513.3422; found: 513.3420.

3, 12, 18, 27-tetraoxapentacyclo[27.1.1.1^{1,29}. 1^{14,16}.1^{14,16}]tetratriacontane-2,13,17,28-tetraone (4c)

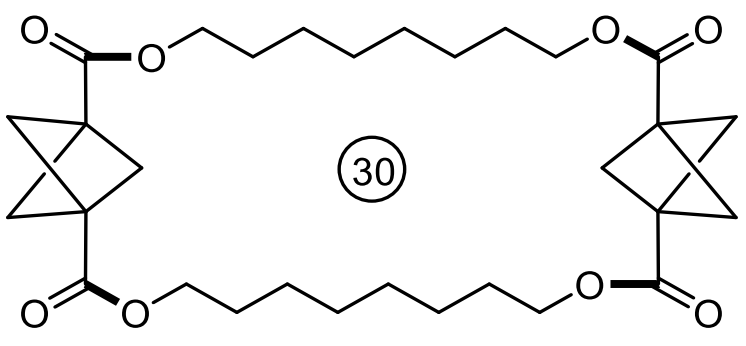

Chemical Formula: $C_{30}H_{44}O_8$
Molecular Weight: 532.67

4c

Following general procedure B, **4c** was obtained from octane-1,8-diol (14.7 mg, 100 µmol), bicyclo[1.1.1]pentane-1,3-dicarbonyl diacyl chloride (prepared from 78.6 mg bicyclo[1.1.1]pentane-1,3-dicarboxylic acid and 1/2 of the material applied, corresponding to 48.6 mg diacyl chloride, 0.252 mmol), C_{11}-resorcin[4]arene (200 mg, 0.181 mmol, corresponding to 30.1 µmol capsule), and Rb_2CO_3

(61.1 mg). 4.03 µmol loss (4%) of octane-1,8-diol during transfer process was detected by ^{1}H NMR. Therefore, 96.0 µmol octane-1,8-diol actually participated in the reaction.

Yield: 13.6 mg, 53%

Property: white solid (melting point: 102–103 °C)

R*f* 0.29 (*n*-hexane/ethyl acetate = 4:1)

IR (ATR): ν_{max} (cm^{-1}) = 2926s, 2856m, 1729s, 1462w, 1390w, 1343m, 1288s, 1208s, 1164m, 1138m, 1031m, 801w, 729w, 468w.

^{1}H NMR (500 MHz, $CDCl_3$) *δ* 4.15 – 4.06 (m, 8H), 2.32 (s, 12H), 1.67 – 1.60 (m, 8H), 1.41 – 1.28 (m, 16H).

^{13}C{^{1}H} NMR (126 MHz, $CDCl_3$) *δ* 169.5, 64.8, 52.8, 37.9, 29.0, 28.6, 25.9.

LCMS (ESI^{+}) *m/z* calculated for $C_{30}H_{44}NaO_8^{+}$ [M + Na]$^{+}$: 555.29; found: 555.30.

HRMS (ESI^{+}) *m/z* calculated for $C_{30}H_{45}O_8^{+}$ [M + H]$^{+}$: 533.3109; found: 533.3117.

3, 10, 16, 23-tetraoxapentacyclo[23.1.1.1^{1,25}. 1^{12,14}.1^{12,14}]triacontane-2,11,15,24-tetraone (4d)

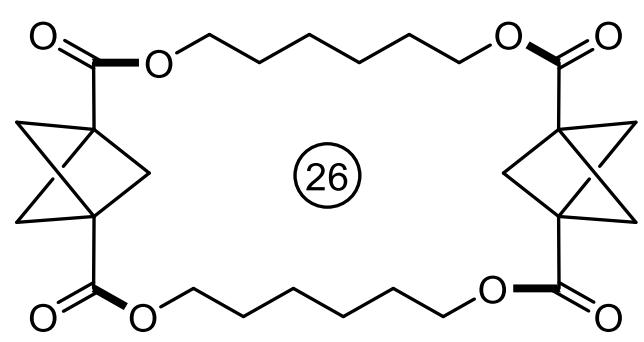

Chemical Formula: $C_{26}H_{36}O_8$
Molecular Weight: 476.57

4d

Following general procedure B, **4d** was obtained from hexane-1,6-diol (12.2 mg, 103 µmol), bicyclo[1.1.1]pentane-1,3-dicarbonyl diacyl chloride (prepared from 157 mg bicyclo[1.1.1]pentane-1,3-dicarboxylic acid and 1/4 of the material applied, corresponding to 48.4 mg diacyl chloride, 0.251 mmol), C_{11}-resorcin[4]arene (201 mg, 0.182 mmol, corresponding to 30.3 µmol capsule), and Rb_2CO_3 (61.0 mg). 2.40 µmol loss (2%) of hexane-1,6-diol during transfer process was detected by ^{1}H NMR. Therefore, 101 µmol hexane-1,6-diol actually participated in the reaction.

Yield: 16.5 mg, 69%

Property: white solid (melting point: 126–127 °C)

R*f* 0.14 (*n*-hexane/ethyl acetate = 4:1)

IR (ATR): ν_{max} (cm^{-1}) = 2929s, 2849m, 1719s, 1472m, 1459m, 1434w, 1380m, 1357m, 1338s, 1279s, 1209s, 1168s, 1079m, 1038s, 1013m, 967s, 920w, 824m, 792w, 727m, 477m, 436w.

^{1}H NMR (500 MHz, $CDCl_3$) *δ* 4.20 – 4.07 (m, 8H), 2.30 (s, 12H), 1.71 – 1.58 (m, 8H), 1.48 – 1.37 (m, 8H).

^{13}C{^{1}H} NMR (126 MHz, $CDCl_3$) *δ* 169.4, 64.9, 52.8, 37.8, 28.6, 26.4.

LCMS (ESI^{+}) *m/z* calculated for $C_{26}H_{36}NaO_8^{+}$ [M + Na]$^{+}$: 499.23; found: 499.15.

HRMS (ESI^{+}) *m/z* calculated for $C_{26}H_{36}NaO_8^{+}$ [M + Na]$^{+}$: 499.2302; found: 499.2312.

Single crystals of **4d** were obtained by crystallization in $CDCl_3$/DCM/*n*-hexane. Vapor diffusion was employed for crystallization. Approximately 15.0 mg of the sample was dissolved in a mixture of 0.40 mL $CDCl_3$ and 0.10 mL DCM in a 2 mL GC vial. The small vial was placed inside a 20 mL vial

containing 3.0 mL *n*-hexane, then the big vial was capped, allowing slow vapor diffusion to occur. X-Ray diffraction confirmed its chemical structure (see Appendix A for details).

3-hydroxy-1,5-dioxacyclohenicosane-6,21-dione (5)

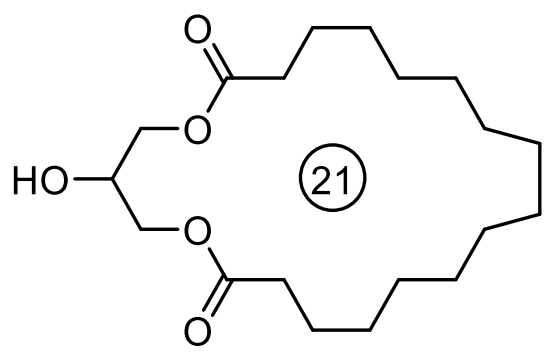

5

Chemical Formula: $C_{19}H_{34}O_5$
Molecular Weight: 342.48

Deprotection of **3ae** to obtain natural product **5**: a 10 mL heart shaped flask was charged with **3ae** (21.0 mg, 48.5 µmol), Pd/C (23.2 mg, 10 wt %, dry, 21.8 µmol) and a stir bar. After switching to an Ar atmosphere, EtOH (1.5 mL) was added by a syringe. The mixture was bubbled with a needle connected to a large H_2 balloon for 15 min, and stirred under H_2 atmosphere at room temperature for another 4 h until full conversion of **3ae** was observed (monitored by TLC). The reaction mixture was directly filtered through a pad of Celite. The filtrate was concentrated to give crude **5** as a colorless oil. Due to facile acyl migration of **5**[7], further column chromatography purification was not performed. The $CDCl_3$ used for NMR analysis of crude **5** was passed through basic Al_2O_3 prior to use. A clean NMR spectrum of crude **5** was observed (see Appendix B).

Yield: 14.7 mg, 89%

Property: colorless oil

R*f* 0.40 (*n*-hexane/ethyl acetate = 2:1)

NMR: see **Table S9** below

IR (ATR): ν_{max} (cm^{-1}) = 3468br, 2924s, 2854s, 1736s, 1459m, 1350w, 1233m, 1161s, 1104s, 1064m, 1022m, 946w, 807w, 725w, 434w.

LCMS (ESI^+) *m/z* calculated for $C_{19}H_{34}NaO_5^+$ $[M + Na]^+$: 365.23; found: 365.30.

HRMS (ESI^+) *m/z* calculated for $C_{19}H_{34}NaO_5^+$ $[M + Na]^+$: 365.2298; found: 365.2297.

Table S9. Detailed spectral comparison with natural sample

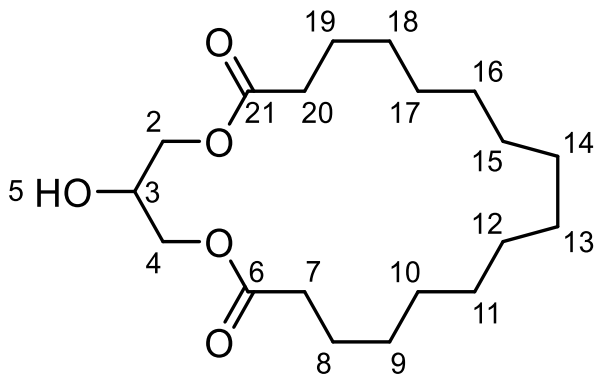

^{1}H NMR		^{13}C NMR[c]	
Synthetic (500 MHz)	Natural (300 MHz)[7]	Synthetic (126 MHz)	Natural (75 MHz)[7]
4.20 (2H, dd, ^{2}J = 11.6 Hz, ^{3}J = 5.0 Hz, H-2 and H-4)	4.15 (2H, dd, J = 12.0, 3.0 Hz, H-2 and H-4)[a]	174.1 (C-6 and C-21)	174.1 (C-6 and C-21)
4.18 (2H, dd, ^{2}J = 11.6 Hz, ^{3}J = 5.4 Hz, H-2' and H-4')	4.14 (2H, dd, J = 12.0, 3.0 Hz, H-2' and H-4')[a]	68.6 (C-3)	68.5 (C-3)
4.08 (1H, "quintet", ^{3}J = 5.2 Hz, H-3)	4.04 (1H, quintet, J = 3.0 Hz, H-3)[a]	65.1 (C-2 and C-4)	65.2 (C-2 and C-4)
2.37 (4H, t, J = 6.8 Hz, H7 and H-20)	2.33 (4H, t, J = 7.2 Hz, H7 and H-20)	34.2 (C-7 and C-20)	34.3 (C-7 and C-20)
1.70 – 1.61 (4H, m, H-8 and H-19)	1.62 (4H, m, H-8 and H-19)	28.4, 28.3	28.5
1.37 – 1.23 (20H, m, H-9─H-18)	"2.27" (20H, m, H-9─H-18)[b]	28.2	28.4
		27.9	28.1
		24.8	25.0

[a] It is worth mentioning here that in our ^{1}H NMR spectrum measured by a 500 MHz instrument, these two "dd" peaks and one "quintet" peak were not in standard shapes. Thus, they were reported by analyzing ^{2}J and ^{3}J in details here.

[b] The chemical shifts of H-9─H-18 were originally reported as "2.27". We assumed that it is a typo of "1.27" due to the following reasons: (1) chemical shifts are typically ordered from largest to smallest; (2) in another structurally similar natural product in the same paper, i.e., 2-hydroxymethyl-1,4-dioxacycloicosane-5,20-dione, those alkyl peaks were reported as "1.30 (m, 20H)"; (3) in our synthetic Bn-protected **5** (i.e., compound **3ae**), similar chemical shifts of 1.42 – 1.21 (m, 20H) were observed.

[c] Good match of two ^{13}C NMR spectra supported the proposed chemical structure of this natural lipid.

5. Details of 1 mmol-scale reaction and recovery of resorcin[4]arene

To demonstrate the scalability of this method, the synthesis of **3ab** was scaled up to 1 mmol.

Preparation of the reaction tube: Rb_2CO_3 (603 mg) was weighed into a 100 mL Schlenk tube (*l* = 170 mm) with a stir bar (5 mm × 18 mm). The same evacuating/backfilling and preheating procedure was applied as mentioned in General procedure B in Section 4.

Preparation of solution A: tetradecane-1,14-diol (230 mg, 0.998 mmol) and C_{11}-resorcin[4]arene (1.99 g, 1.80 mmol, corresponding to 0.300 mmol capsule) was weighed into a 50 mL heart shaped flask charged with a stir bar (6 mm × 10 mm). 15 mL dry PhCl was added to dissolve both materials. To facilitate such dissolution, heating and stirring at 70 °C was applied until a clear solution was obtained.

Preparation of solution B: bicyclo[1.1.1]pentane-1,3-dicarbonyl diacyl chloride (prepared from 468 mg bicyclo[1.1.1]pentane-1,3-dicarboxylic acid, corresponding to 579 mg diacyl chloride, 3.00 mmol) was dissolved in 6.0 mL dry PhCl, and 5.0 mL of such solution was applied for the reaction (corresponding to 482 mg diacyl chloride, 2.50 mmol). To facilitate dissolution of the diacyl chloride, sonication was applied for approximately 5 min.

Mixing of solution A and solution B: solution A and B were injected into the preheated Schlenk tube simultaneously. The loss of tetradecane-1,14-diol during transfer was negligible on such scale.

Work-up and product separation: the same reaction time was applied (1.5 h). To avoid potential loss of C_{11}-resorcin[4]arene, the crude reaction mixture was directly filtered with a pad of Celite without further neutralizing with Et_3N. Then, **3ab** was obtained by column chromatography (ca. 150 g silica manual column, *d* = 50 mm, *l* = 180 mm, eluted with *n*-hexane/EA = 40:1 to 15:1) as a white solid. **Yield:** 238 mg, 68%

Recovery of resorcin[4]arene: further elution of the column with *n*-hexane/EA = 1:2 and removing the solvent gave a crude light-brown solid containing C_{11}-resorcin[4]arene, and it was then washed with MeCN (10 × 5.0 mL) to give pure recovered C_{11}-resorcin[4]arene as a pale-yellow powder (1.50 g, 75% recovery rate). The purity of the recovered C_{11}-resorcin[4]arene was good, as compared with the ^1H NMR spectrum of the C_{11}-resorcin[4]arene applied for the reaction (shown below).

^1H NMR (500 MHz, acetone-d_6, 298K) of C_{11}-resorcin[4]arene applied for the reaction

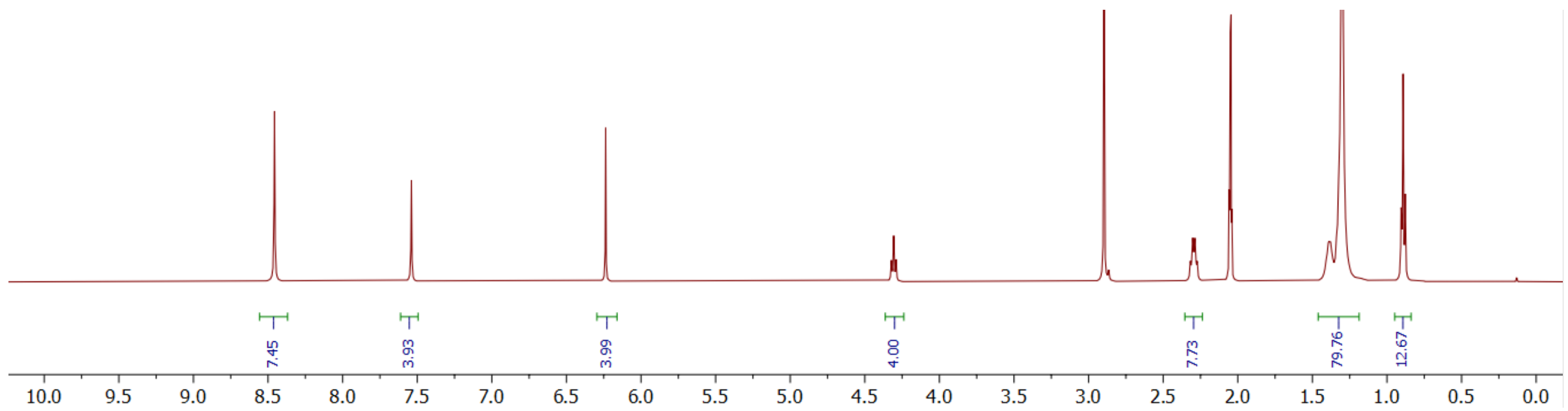

^1H NMR (500 MHz, acetone-d_6, 298K) of C_{11}-resorcin[4]arene recovered after the reaction

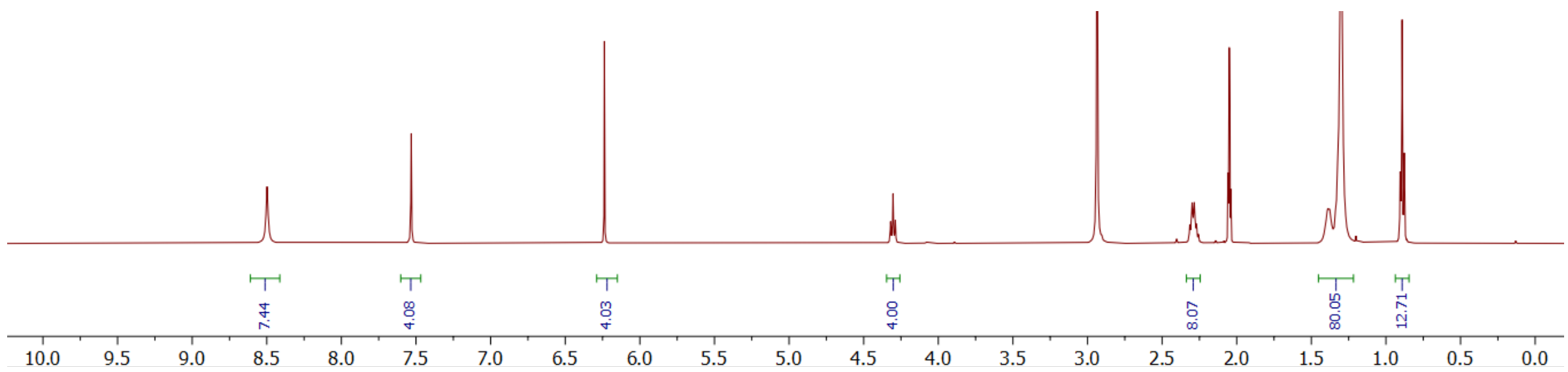

6. Challenging substrates

We believe it is important to clarify the limitation of this method by showing representative challenging substrates here. We hope it will inspire future advancement in this field.

Size limitation

As shown in the main text, this method is generally suitable for macrodiolides/macrotetrolides from 20- to 30-membered size. We found that ring sizes smaller than 20-membered or larger than 30-membered exhibited significantly lower yields (see examples below).

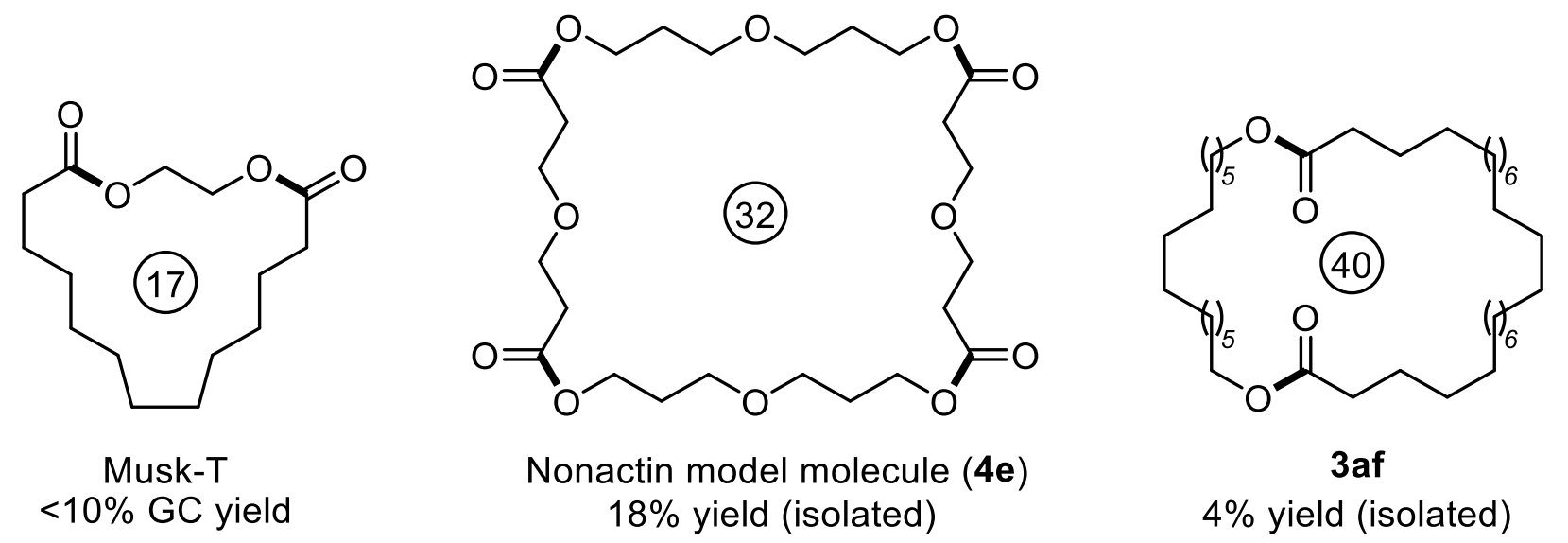

(i) Musk-T

We tested the reaction between ethane-1,2-diol and tridecanedioyl dichloride under standard conditions. We also tested several solvents with more bulky molecular volumes, attempting to improve the yield. Unfortunately, such endeavor was found not fruitful (see Table S10 below).

Table S10. Performance of different solvents for synthesizing Musk-T

Solvent	GC yield (%)[b]
chlorobenzene (standard conditions)	9
1-chloronaphthalene[a]	6
1-chloro-2-methylbenzene	7
1-chloro-4-methylbenzene	8

[a] The commercial source from Thermo Scientific is 85% purity, the rest 15% is 2-chloronaphthalene. [b] Determined by using *n*-pentadecane as the internal standard and response factors calculated according to Ref. 5.

(ii) 1,5,9,13,17,21,25,29-octaoxacyclodotriacontane-2,8,18,24-tetraone (4e)

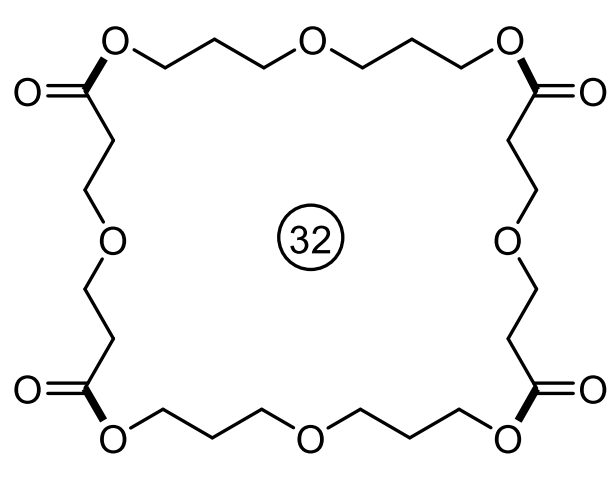

4e

Chemical Formula: $C_{24}H_{40}O_{12}$
Molecular Weight: 520.57

Following general procedure B, **4e** was obtained from 3,3'-oxybis(propan-1-ol) (14.3 mg, 106 µmol), 3,3'-oxydipropionyl chloride (prepared from 122 mg 3,3'-oxydipropionic acid and 0.50 mL from 1.5 mL PhCl solution applied, corresponding to 50.0 mg diacyl chloride, 0.251 mmol), C_{11}-resorcin[4]arene (200 mg, 0.181 mmol, corresponding to 30.1 µmol capsule), and Rb_2CO_3 (61.3 mg). 4.85 µmol loss (5%) of 3,3'-oxybis(propan-1-ol) during transfer process was detected by ^{1}H NMR. Therefore, 101 µmol 3,3'-oxybis(propan-1-ol) actually participated in the reaction. Before column chromatography, the crude material was concentrated to a volume of approximately 0.5 mL and MeCN (4.0 mL) was added, sonicated for 5 min, and placed inside the -20 °C freezer for 20 min. In this way, C_{11}-resorcin[4]arene

could be precipitated as a white solid and removed by filtration. This process was repeated once more until most C_{11}-resorcin[4]arene was removed from the reaction mixture. Then the filtrate was concentrated and the final residue was purified by flash column chromatography (*n*-hexane/ethyl acetate = 3:1 to 2:1) to give **4e**.

Yield: 4.7 mg, 18%

Property: colorless oil

IR (ATR): ν_{max} (cm^{-1}) = 2959m, 2925m, 2871m, 1733s, 1462w, 1421w, 1362m, 1323w, 1261s, 1180s, 1116s, 1067m, 714w, 601w.

R_f 0.14 (*n*-hexane/ethyl acetate = 1:1)

^{1}H NMR (500 MHz, $CDCl_3$) δ 4.19 (t, J = 6.3 Hz, 8H), 3.72 (t, J = 6.2 Hz, 8H), 3.49 (t, J = 6.3 Hz, 8H), 2.55 (t, J = 6.2 Hz, 8H), 1.89 (p, J = 6.3 Hz, 8H).

$^{13}C\{^1H\}$ NMR (126 MHz, $CDCl_3$) δ 171.7, 67.4, 66.5, 61.8, 35.3, 29.1.

4e is a molecule designed for mimicking the function of natural macrotetrolide antibiotic nonactin[8]. The NMR spectra are accordance with literature report[8].

LCMS (ESI^+) *m/z* calculated for $C_{24}H_{40}NaO_{12}^+$ $[M + Na]^+$: 543.24; found: 543.15.

HRMS (ESI^+) *m/z* calculated for $C_{24}H_{40}NaO_{12}^+$ $[M + Na]^+$: 543.2412; found: 543.2415.

Control experiment without the capsule: a control experiment was performed by applying 3'-oxybis(propan-1-ol) (13.0 mg, 96.9 µmol), 3,3'-oxydipropionyl chloride (prepared from 108 mg 3,3'-oxydipropionic acid and 0.50 mL from 1.3 mL PhCl solution applied, corresponding to 51.0 mg diacyl chloride, 0.256 mmol), and Rb_2CO_3 (61.8 mg). After 1.5 h, an aliquot (approximately 10 µL) of the reaction mixture was diluted with 1.5 mL MeOH and analyzed by LCMS. However, no desired mass corresponding to **4e** was observed. This experiment indicated that although this method was unable to form **4e** in high yield, promoting effect still existed to some extent.

It is also worth mentioning that although attempts of improving the yield of **4e** was also not fruitful, this method still displayed increased synthetic efficiency compared with the original synthetic method (18%, 1 purification vs. 7%, 3 purifications).

(iii) 1,16-dioxacyclotetracontane-17,40-dione (3af)

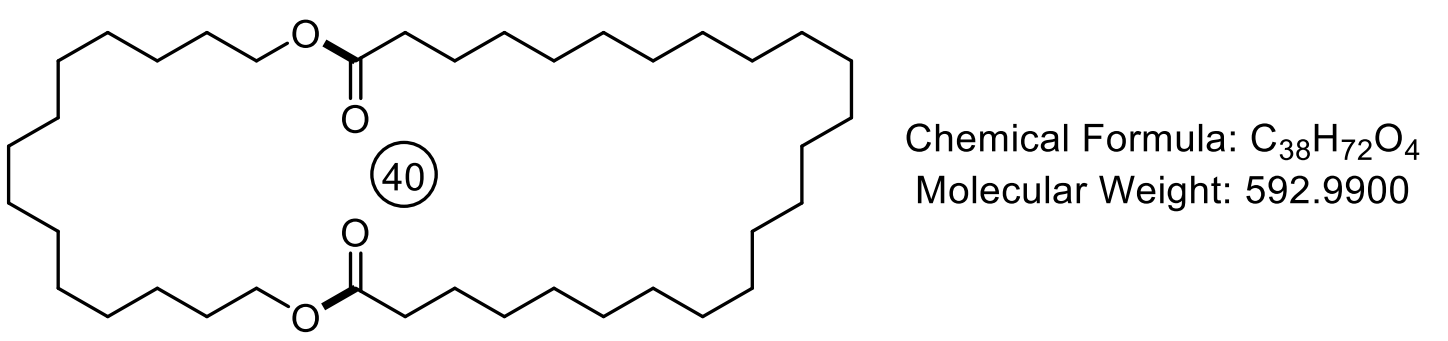

3af

Following general procedure B, **3af** was obtained from tetradecane-1,14-diol (24.3 mg, 105 µmol), tetracosanedioyl dichloride (116 mg, 0.266 mmol), C_{11}-resorcin[4]arene (200 mg, 0.181 mmol, corresponding to 30.1 µmol capsule), and Rb_2CO_3 (62.3 mg). 1.78 µmol loss (2%) of tetradecane-1,14-diol during transfer process was detected by ^{1}H NMR. Therefore, 103 µmol tetradecane-1,14-diol actually participated in the reaction.

Yield: 2.2 mg, 4%

Property: white solid (melting point: 71–72 °C)

IR (ATR): ν_{max} (cm^{-1}) = 2914s, 2850s, 1731s, 1473m, 1360w, 1323w, 1279w, 1247w, 1211w, 1164m, 1111m, 1058m, 815w, 717m, 459w, 446w, 424w.

$\mathbf{R}_f$ 0.72 (*n*-hexane/ethyl acetate = 4:1)

^{1}H NMR (500 MHz, $CDCl_3$) δ 4.07 (t, J = 6.5 Hz, 4H), 2.30 (t, J = 7.3 Hz, 4H), 1.58 – 1.67 (m, 8H), 1.39 – 1.23 (m, 56 H).

^{13}C{^{1}H} NMR (126 MHz, $CDCl_3$) δ 174.2, 64.5, 34.6, 29.7 (3 close peaks), 29.6 (4 close peaks), 29.5, 29.4 (3 close peaks), 29.1, 29.0, 28.8, 26.2, 25.2.

LCMS (ESI$^+$) *m/z* calculated for $C_{38}H_{72}NaO_4^+$ [M + Na]$^+$: 615.53; found: 615.50.

HRMS (ESI$^+$) *m/z* calculated for $C_{38}H_{72}NaO_4^+$ [M + Na]$^+$: 615.5323; found: 615.5310.

<u>Control experiment without the capsule:</u> a control experiment was performed by applying tetradecane-1,14-diol (23.2 mg, 101 µmol), tetracosanedioyl dichloride (109 mg, 0.250 mmol), and Rb_2CO_3 (61.2 mg). 5.18 µmol loss (5%) of tetradecane-1,14-diol during transfer process was detected by ^{1}H NMR. Therefore, 95.8 µmol tetradecane-1,14-diol actually participated in the reaction. **Yield:** 2.3 mg, 4%

Limitation for phenol-type substrate

We tested the reaction between (*R*)-BINOL and octanedioyl dichloride under standard conditions. However, no desired macrodiolide was detected by TLC or GC analysis. GC analysis indicated most (*R*)-BINOL remained in the reaction system (see GC traces below, ca. 70 % remained, determined by using *n*-pentadecane as the internal standard and response factors calculated according to Ref. 5).

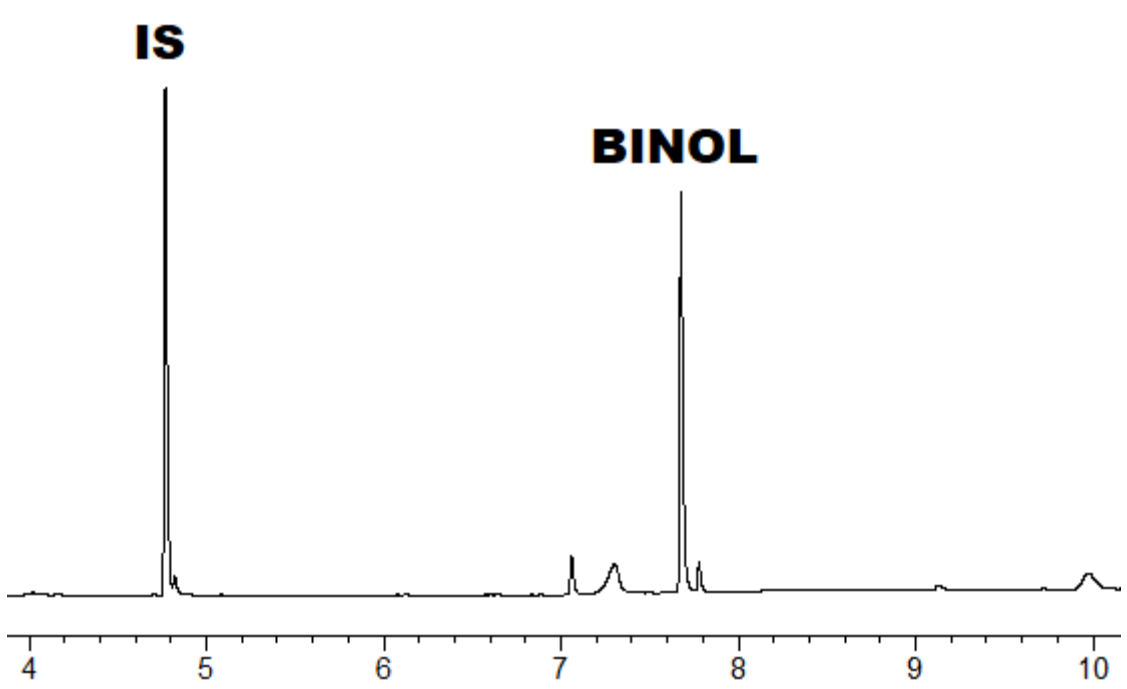

BINOL-participated reaction

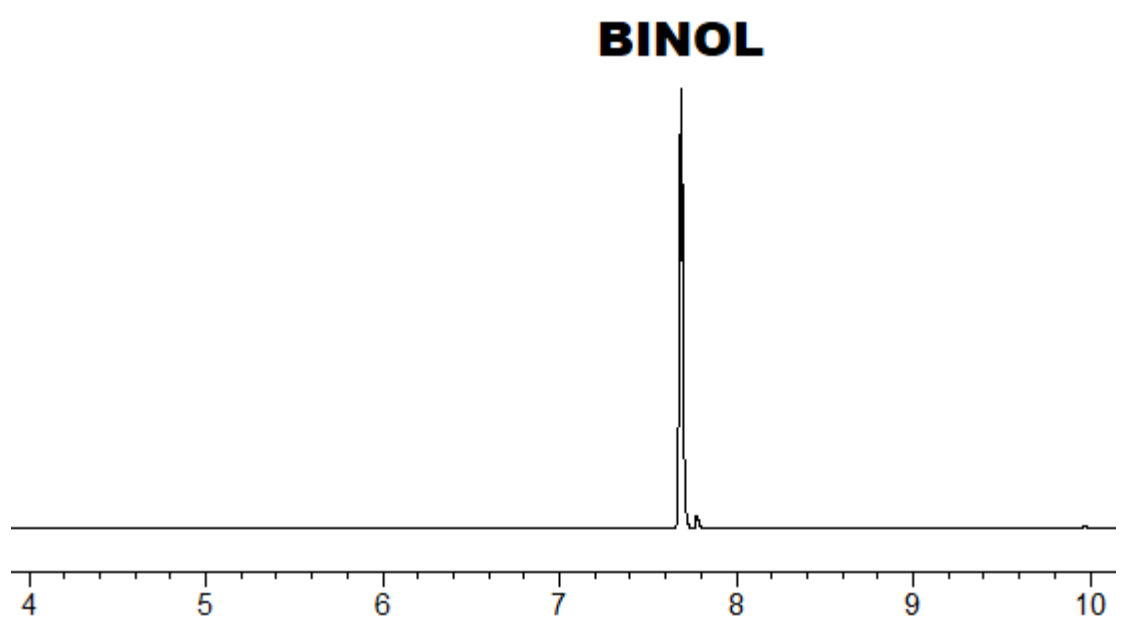

pure BINOL

Figure S-9: Comparison of GC traces (BINOL-participated reaction and pure BINOL), IS: internal standard (*n*-pentadecane)

As we showed in Section 5, in the 1 mmol-scale reaction, 75% of the C_{11}-resorcin[4]arene applied could be recovered after the reaction without being acylated. We assumed that this might serve as another evidence for lower reactivities of phenol groups in such reaction system.

7. Control experiments without capsule

To fully demonstrate the function of capsule **I** in this work, we performed more control experiments without capsule **I** by omitting it from the standard conditions while keeping others unchanged. **The same procedure** described in General procedure B was applied for all reactions.

To ensure that such controlled experiments covered substrates as diversely as possible, we considered: (1) all reactions forming macrodiolides in < 40% yields; (2) all reactions forming macrotetrolides; (3) the reaction with the highest yield. Following such rule, a total of **14 reactions** (i.e., forming **3e–f**, **3k–l**, **3p**, **3s**, **3z–aa**, **3ad–ae**, and **4a–d)** were selected for study.

Internal standard *n*-pentadecane for GC analysis was added after all reactions to avoid potential influence on the reaction, which was similar to control experiments for the model reaction (Section 3). The loss of diol during solution transfer process (typically 2-3% according to multiple measurements) was not taken into consideration when calculating GC yield for each control experiment. It was only taken into consideration when purifications were performed (when GC yields > 5%, i.e., **3e**, **3k–l**, **3p**, and **3z–aa**) by using dimethyl terephthalate as internal standard in acetone-d_6.

In all cases, significantly lower yields (≤ 8% yields) were observed compared with standard conditions (as listed in Table S11), which demonstrated the key role of capsule **I** in this methodology.

Table S11. Results of control experiments (w/o **I**) and comparison with standard conditions (w/ **I**)

Products	**3e**	**3f**	**3k**	**3l**	**3p**	**3s**	**3z**	**3aa**	**3ad**	**3ae**	**4a**	**4b**	**4c**	**4d**
GC yield (%) **w/ I**	30 (31)	33 (39)	47 (38)	33 (33)	31 (31)	26 (29)	33 (31)	quant. (84)	22 (24)	32 (30)	42 (39)	20 (33)	42 (53)	42 (69)
GC yield (%) **w/o I**	15 (6)	5	13 (8)	9 (8)	7 (3)	3	9 (5)	9 (8)	0	6 (2)	0	3	0	2

GC yields were determined by using *n*-pentadecane as the internal standard and response factors calculated according to Ref. 5. Yields of isolated products shown in parentheses.

The experimental details and comparison of GC traces (standard conditions vs. control experiments) for these reactions were shown below.

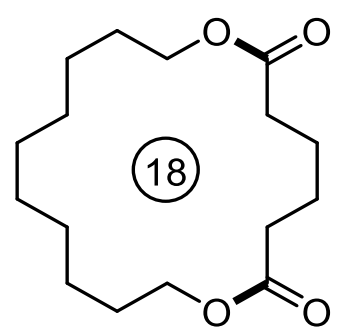

3e

Control experiments forming **3e** was performed by using decane-1,10-diol (17.4 mg, 99.8 µmol), adipoyl diacyl chloride (prepared from 147 mg adipic acid and 1/4 of the material applied, corresponding to 45.9 mg diacyl chloride, 0.251 mmol), and Rb_2CO_3 (61.6 mg). 4.90 µmol loss (5%) of decane-1,10-diol during transfer process was detected by ^{1}H NMR. Therefore, 94.9 µmol decane-1,10-diol actually participated in the reaction. **Yield:** 1.6 mg, 6%

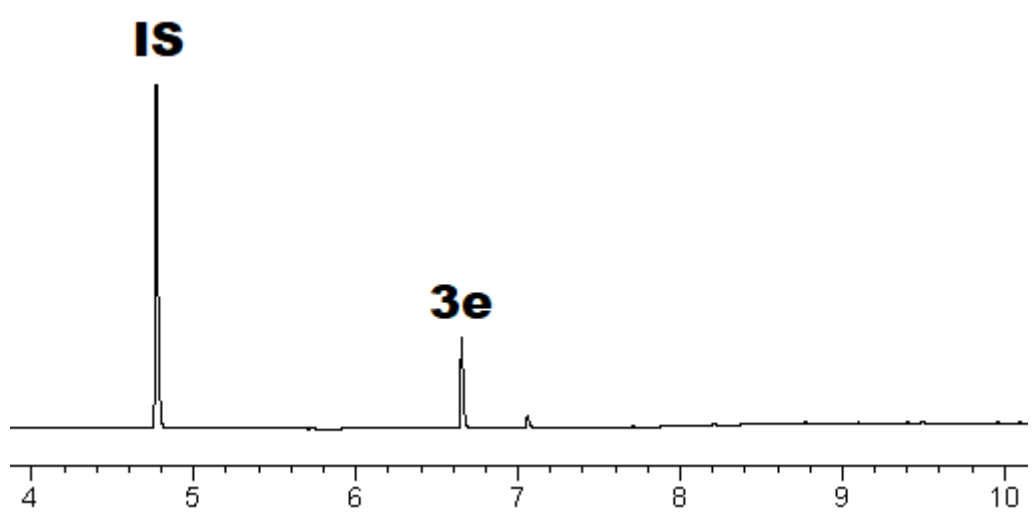

standard conditions **(w/ capsule)**

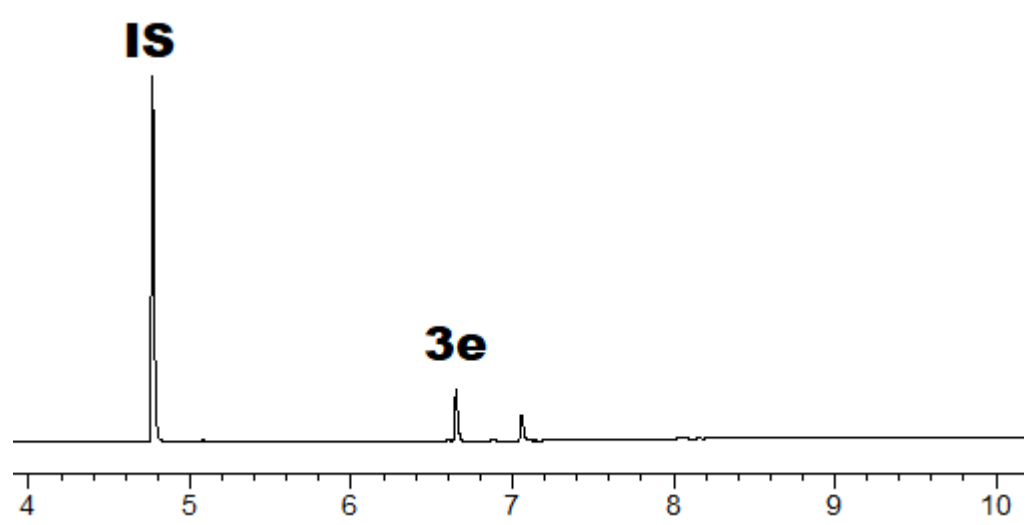

control experiment **(w/o capsule)**

Figure S-10: Comparison of GC traces (standard conditions and control experiment for **3e)**, IS: internal standard (*n*-pentadecane)

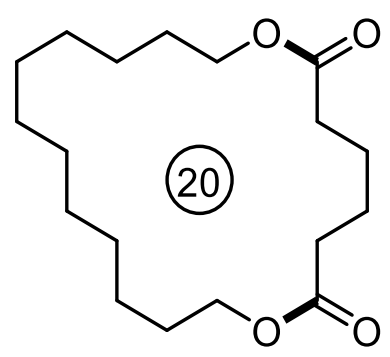

3f

Control experiments forming **3f** was performed by using dodecane-1,12-diol (20.6 mg, 102 µmol), adipoyl dichloride (prepared from 147 mg adipic acid and 1/4 of the material applied, corresponding to 46.0 mg diacyl chloride, 0.251 mmol), and Rb_2CO_3 (61.2 mg). Since **3f** was detected in only 5% GC yield, purification was not performed.

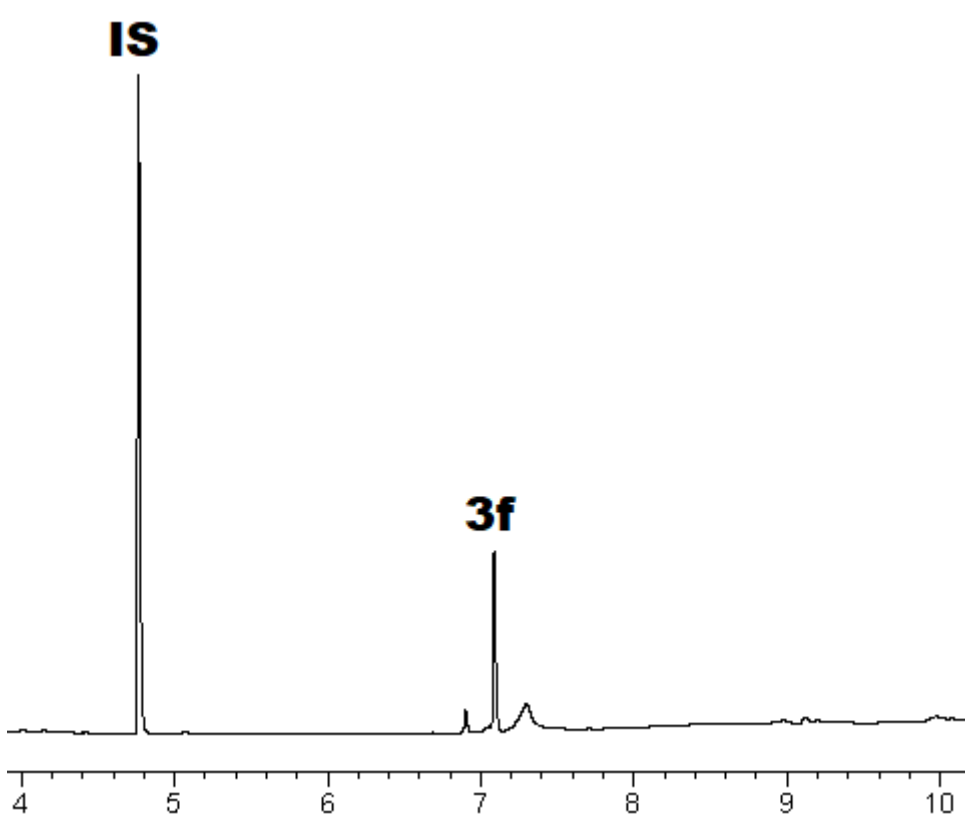

standard conditions **(w/ capsule)**

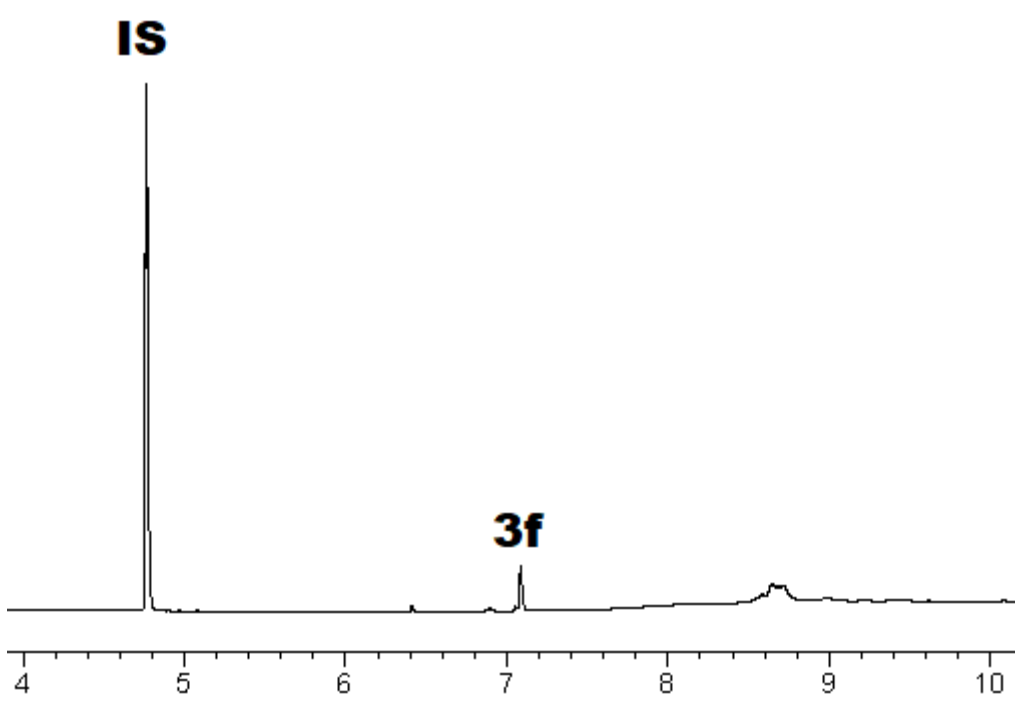

control experiment **(w/o capsule)**

Figure S-11: Comparison of GC traces (standard conditions and control experiment for **3f)**, IS: internal standard (*n*-pentadecane)

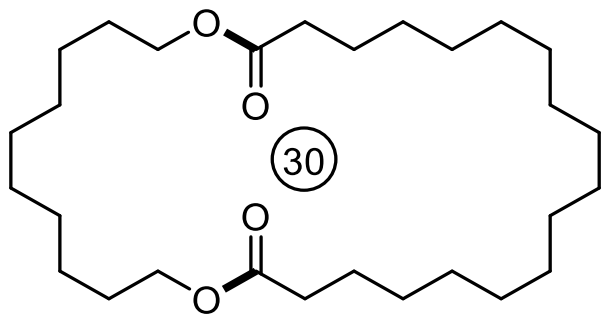

3k

Control experiments forming **3k** was performed by using decane-1,10-diol (17.5 mg, 100 µmol), octadecanedioyl dichloride (88.0 mg, 0.250 mmol), and Rb_2CO_3 (60.6 mg). 3.60 µmol loss (4%) of decane-1,10-diol during transfer process was detected by ^1H NMR. Therefore, 96.4 µmol decane-1,10-diol actually participated in the reaction. **Yield:** 3.4 mg, 8%

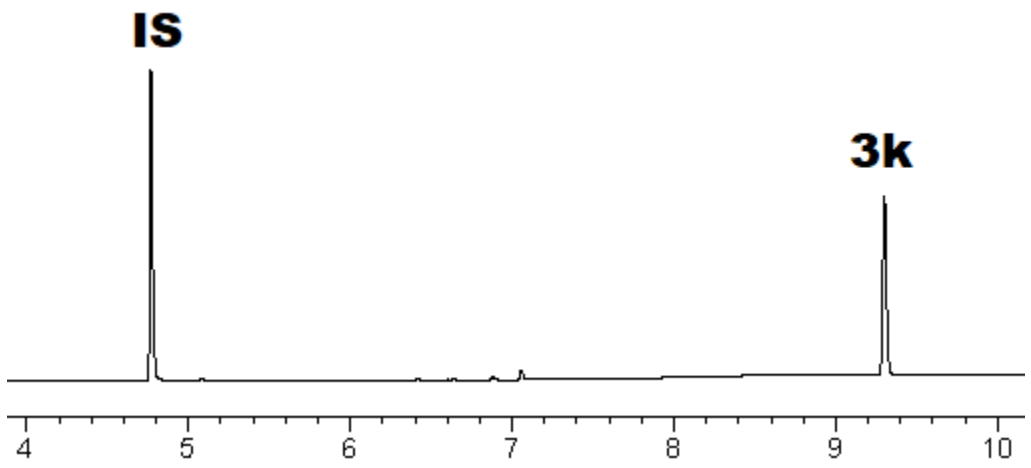

standard conditions **(w/ capsule)**

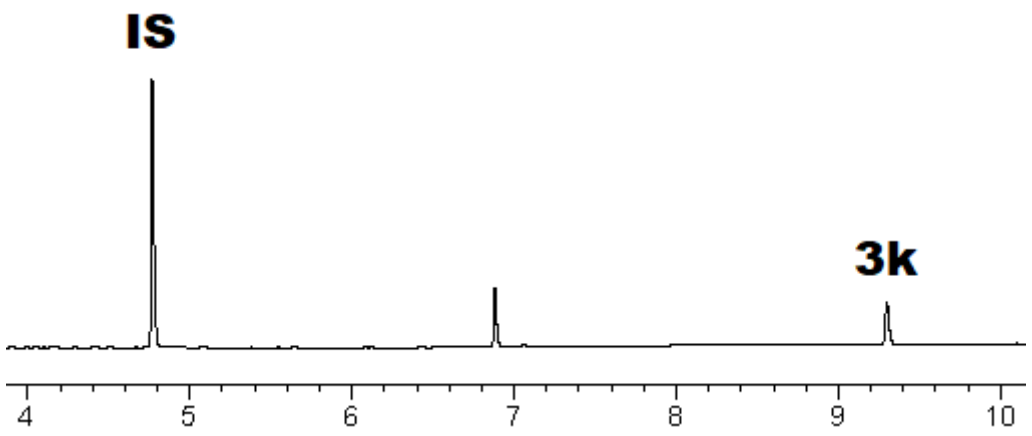

control experiment **(w/o capsule)**

Figure S-12: Comparison of GC traces (standard conditions and control experiment for **3k)**, IS: internal standard (*n*-pentadecane)

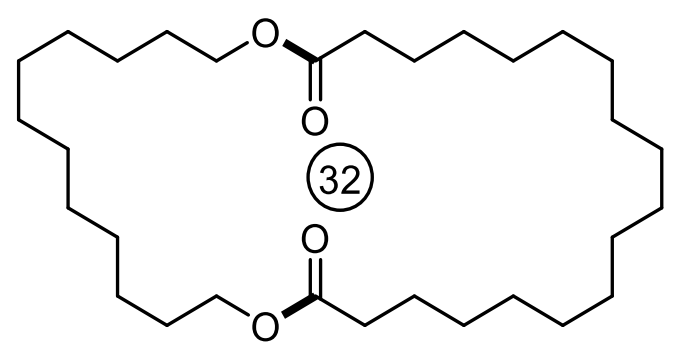

3l

Control experiments forming **3l** was performed by using dodecane-1,12-diol (20.1 mg, 99.4 µmol), octadecanedioyl dichloride (89.2 mg, 0.254 mmol), and Rb_2CO_3 (60.5 mg). 5.38 µmol loss (5%) of dodecane-1,12-diol during transfer process was detected by ^1H NMR. Therefore, 94.0 µmol dodecane-1,12-diol actually participated in the reaction. **Yield:** 3.7 mg, 8%

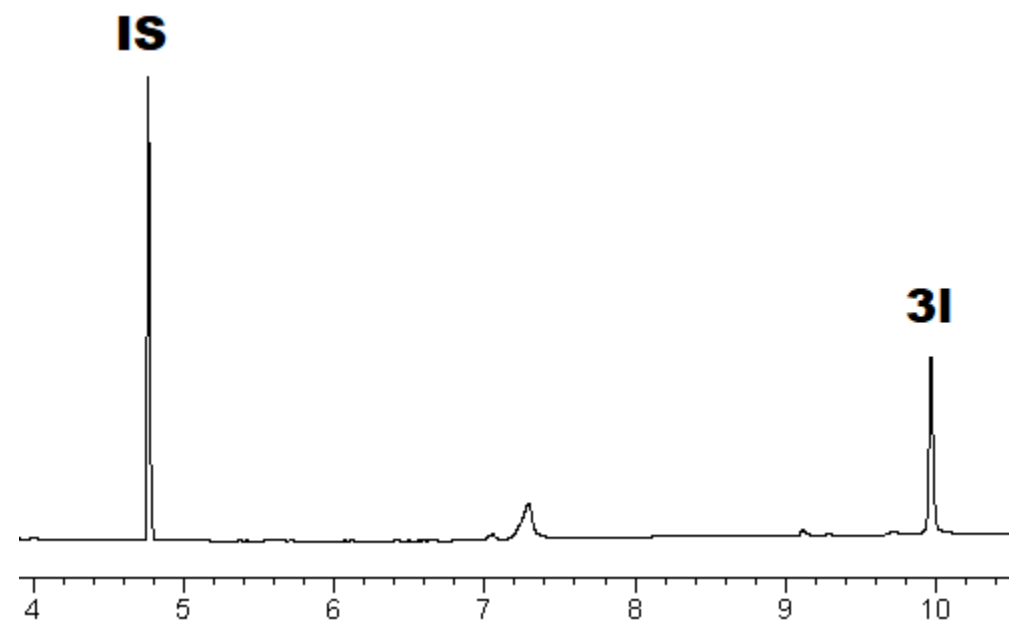

standard conditions **(w/ capsule)**

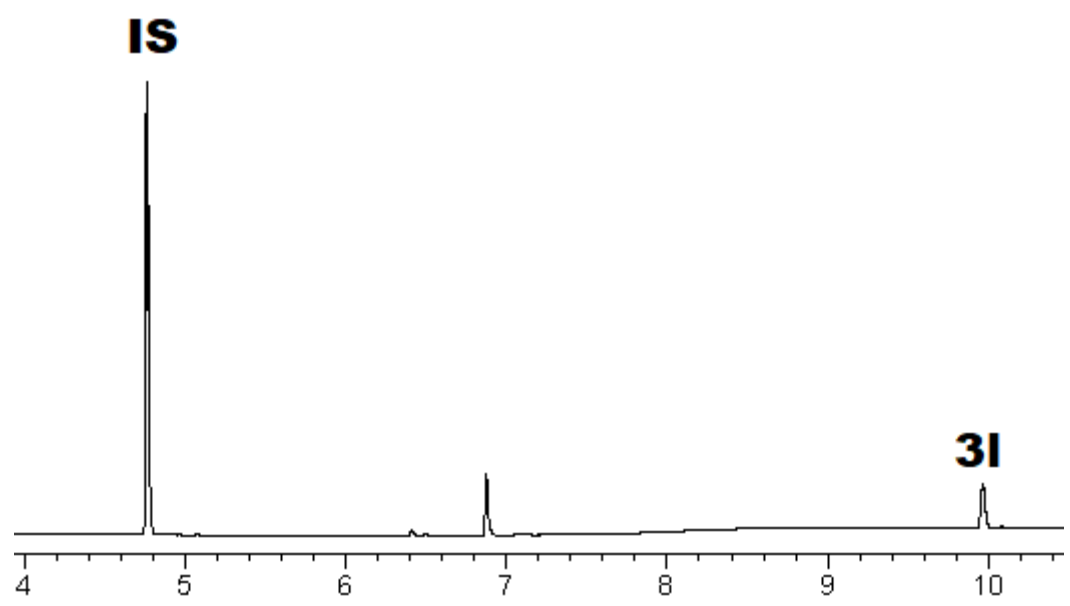

control experiment **(w/o capsule)**

Figure S-13: Comparison of GC traces (standard conditions and control experiment for **3l)**, IS: internal standard (*n*-pentadecane)

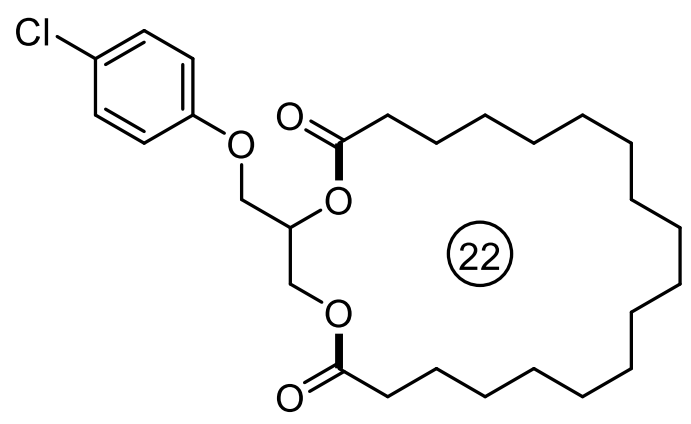

3p

Control experiments forming **3p** was performed by using chlorphenesin (3-(4-chlorophenoxy)propane-1,2-diol, 20.4 mg, 101 µmol), octadecanedioyl dichloride (88.3 mg, 0.251 mmol), and Rb_2CO_3 (61.2 mg) under 90 °C. 3.60 µmol loss (4%) of chlorphenesin during transfer process was detected by ^1H NMR. Therefore, 97.4 µmol chlorphenesin actually participated in the reaction. **Yield:** 1.3 mg, 3%

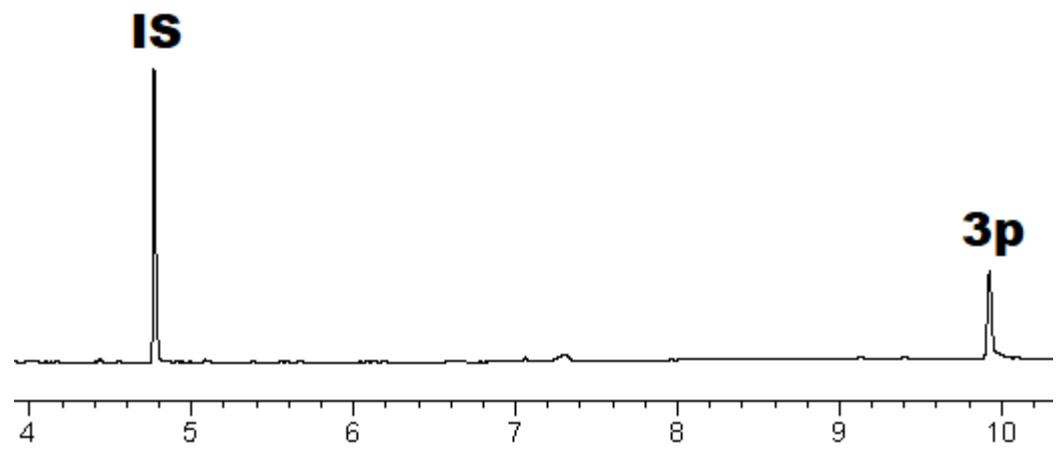

standard condition**s (w/ capsule)**

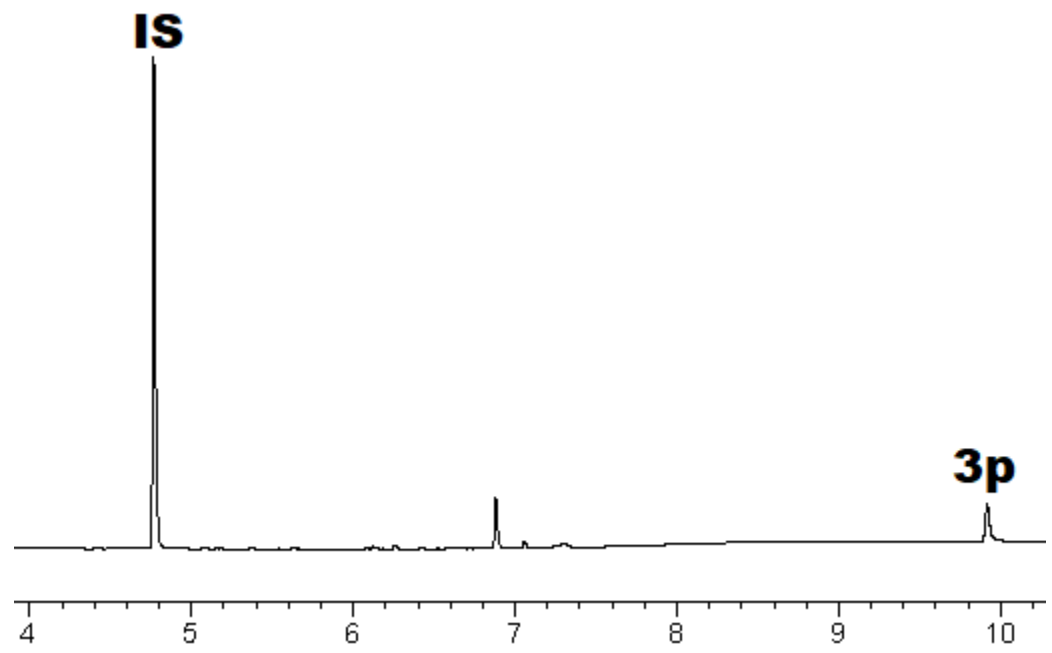

control experiment **(w/o capsule)**

Figure S-14: Comparison of GC traces (standard conditions and control experiment for **3p)**, IS: internal standard (*n*-pentadecane)

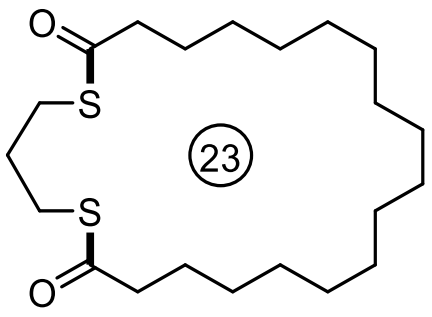

3s

Control experiments forming **3s** was performed by using propane-1,3-dithiol (10.0 µL, 10.7 mg, 98.9 µmol), octadecanedioyl dichloride (88.3 mg, 0.251 mmol), and Rb_2CO_3 (61.4 mg) under 90 °C for 3 h. Since **3s** was detected in only 3% GC yield, purification was not performed.

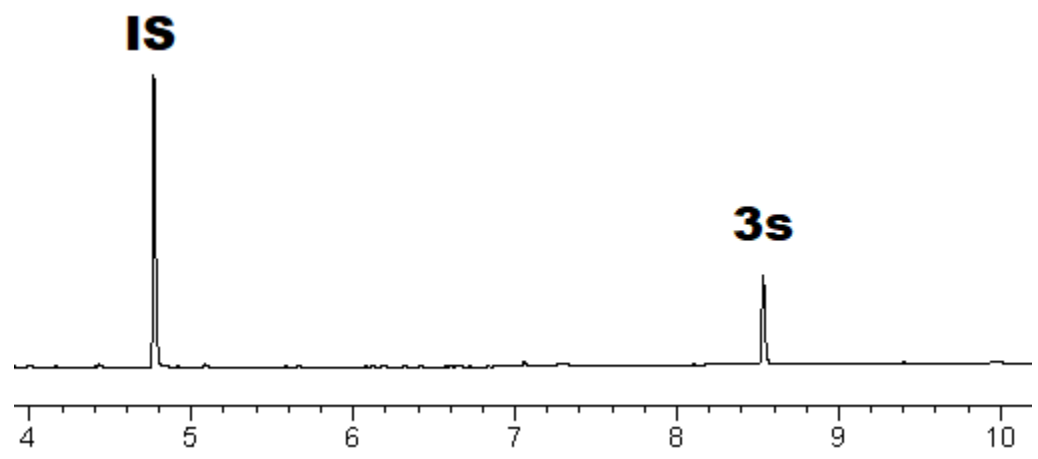

standard conditions **(w/ capsule)**

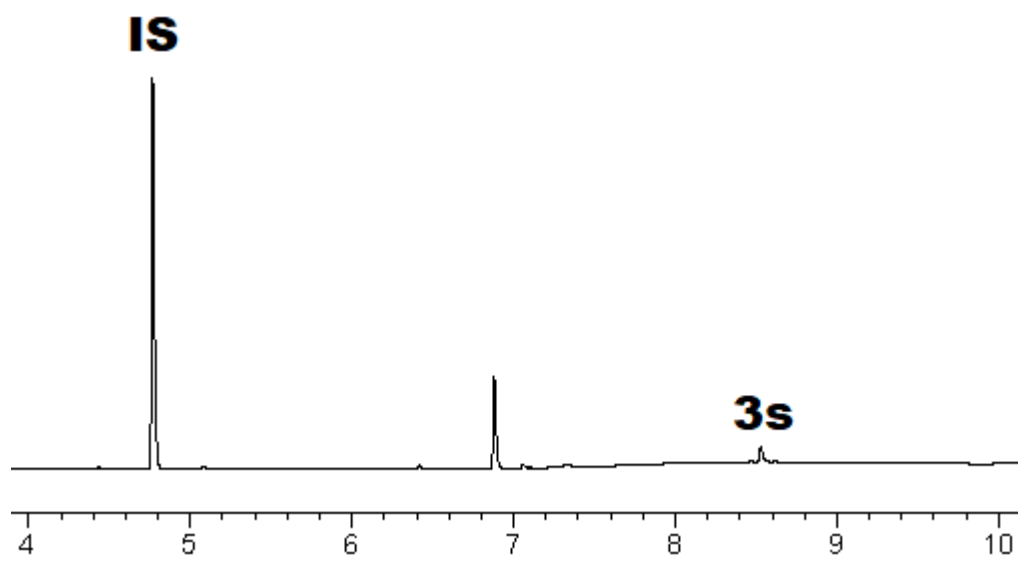

control experiment **(w/o capsule)**

Figure S-15: Comparison of GC traces (standard conditions and control experiment for **3s)**, IS: internal standard (*n*-pentadecane)

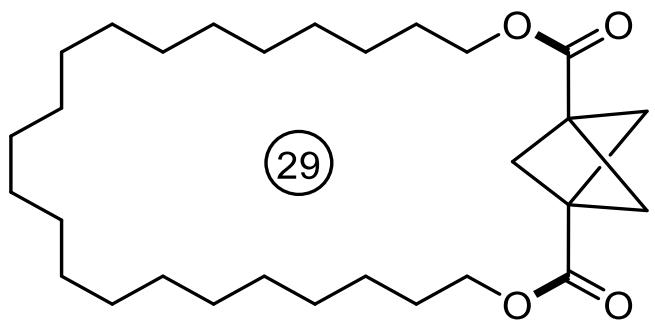

3z

Control experiments forming **3z** was performed by using docosane-1,22-diol (34.2 mg, 99.8 µmol), bicyclo[1.1.1]pentane-1,3-dicarbonyl diacyl chloride (prepared from 156 mg bicyclo[1.1.1]pentane-1,3-dicarboxylic acid and 1/4 of the material applied, corresponding to 48.2 mg diacyl chloride, 0.250 mmol), and Rb_2CO_3 (61.8 mg). 1.62 µmol loss (2%) of docosane-1,22-diol during transfer process was detected by ^1H NMR. Therefore, 98.2 µmol docosane-1,22-diol actually participated in the reaction. **Yield:** 2.1 mg, 5%

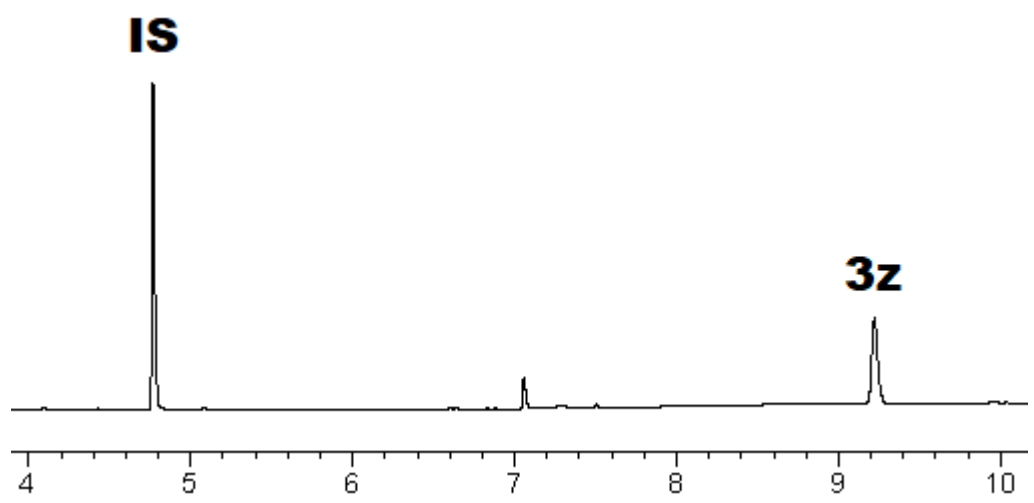

standard conditions **(w/ capsule)**

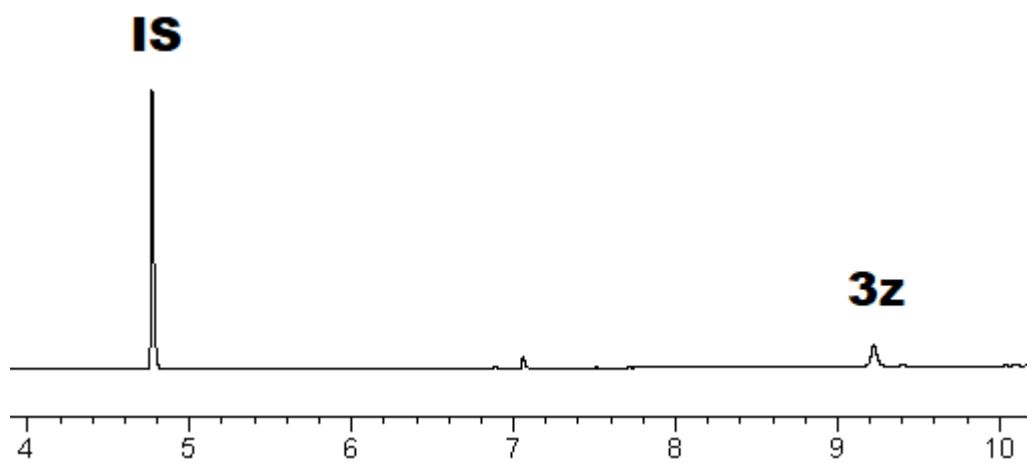

control experiment **(w/o capsule)**

Figure S-16: Comparison of GC traces (standard conditions and control experiment for **3z)**, IS: internal standard (*n*-pentadecane)

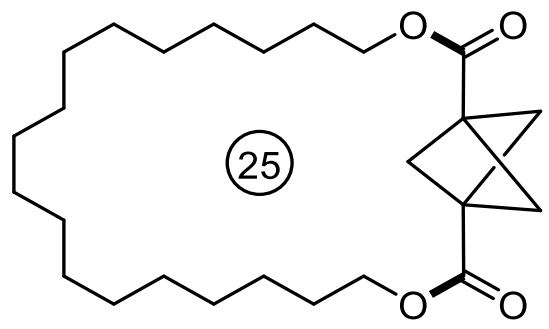

3aa

Control experiments forming **3aa** was performed by using octadecane-1,18-diol (28.6 mg, 99.8 µmol), bicyclo[1.1.1]pentane-1,3-dicarbonyl diacyl chloride (prepared from 156 mg bicyclo[1.1.1]pentane-1,3-dicarboxylic acid and 1/4 of the material applied, corresponding to 48.2 mg diacyl chloride, 0.250 mmol), and Rb_2CO_3 (60.6 mg). 4.38 µmol loss (4%) of octadecane-1,18-diol during transfer process was detected by ^{1}H NMR. Therefore, 95.4 µmol octadecane-1,18-diol actually participated in the reaction. **Yield:** 3.0 mg, 8%

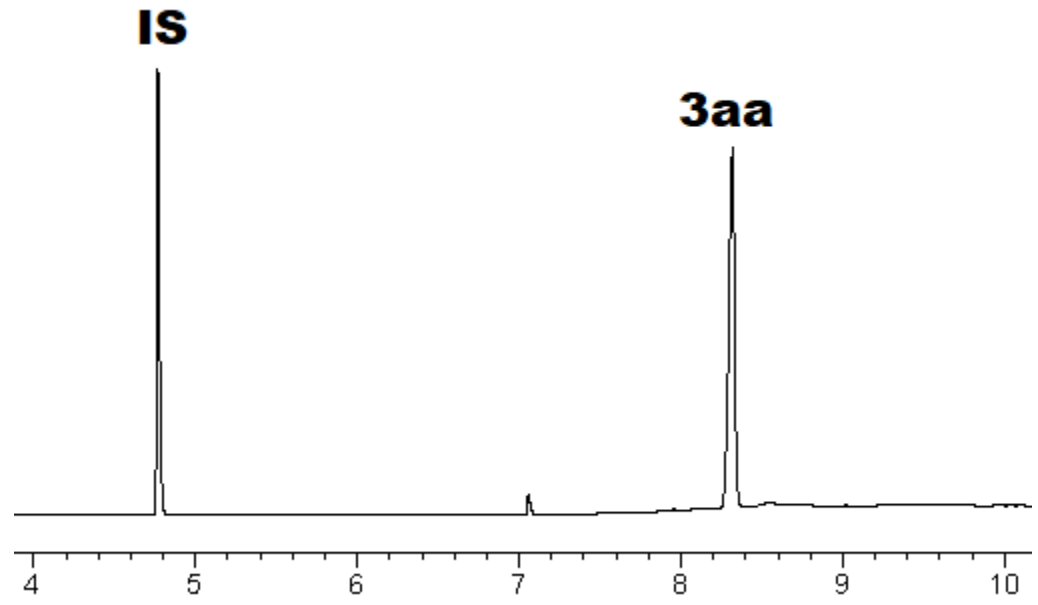

standard conditions **(w/ capsule)**

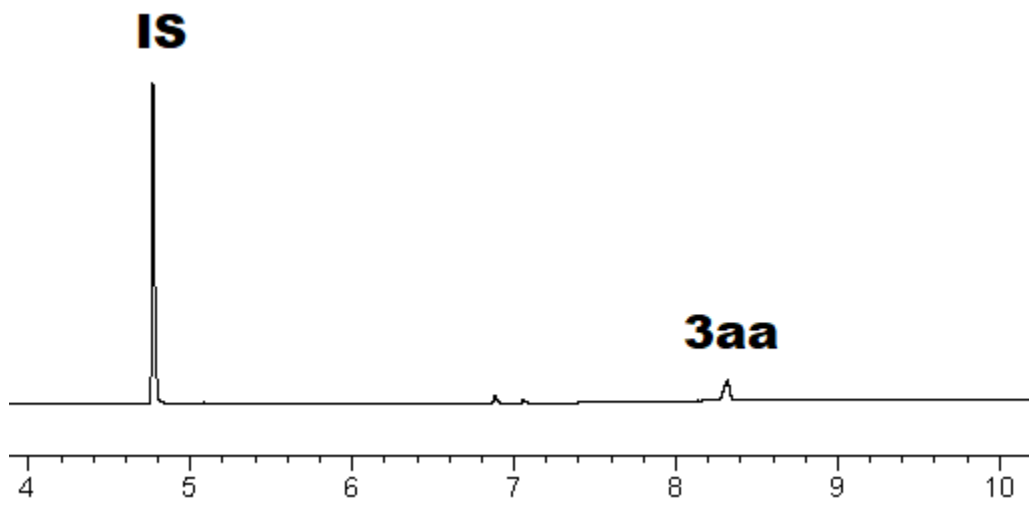

control experiment **(w/o capsule)**

Figure S-17: Comparison of GC traces (standard conditions and control experiment for **3aa)**, IS: internal standard (*n*-pentadecane)

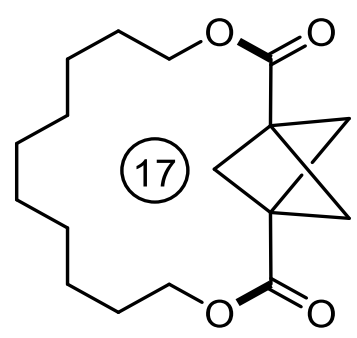

3ad

Control experiments forming **3ad** was performed by using decane-1,10-diol (17.8 mg, 102 μmol), bicyclo[1.1.1]pentane-1,3-dicarbonyl diacyl chloride (prepared from 77.5 mg bicyclo[1.1.1]pentane-1,3-dicarboxylic acid and 0.50 mL of the 1.0 mL PhCl solution applied, corresponding to 47.9 mg diacyl chloride, 0.248 mmol), and Rb_2CO_3 (61.5 mg). **3ad** was not detected by GC in this control experiment.

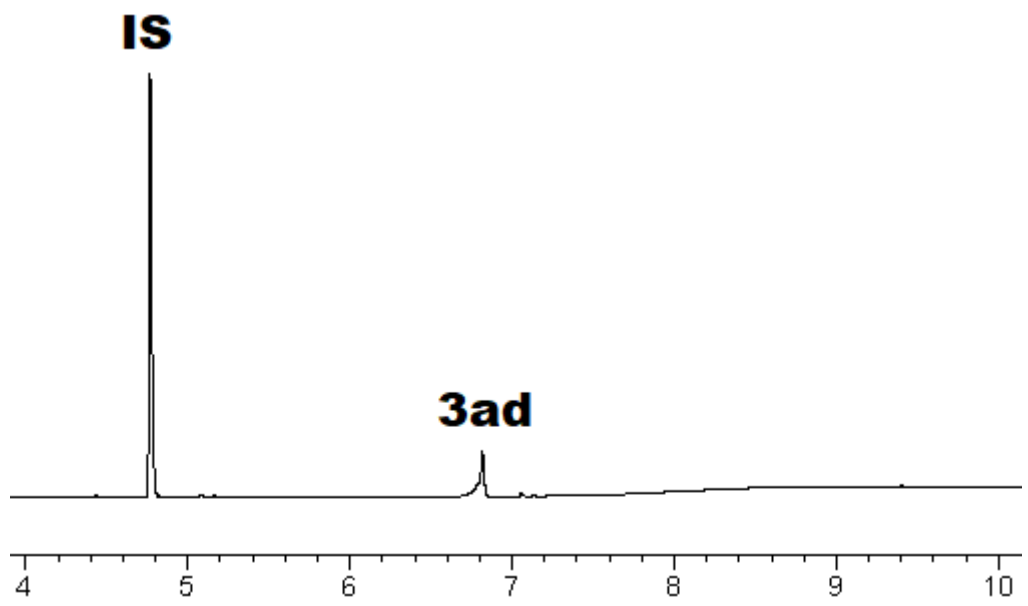

standard conditions **(w/ capsule)**

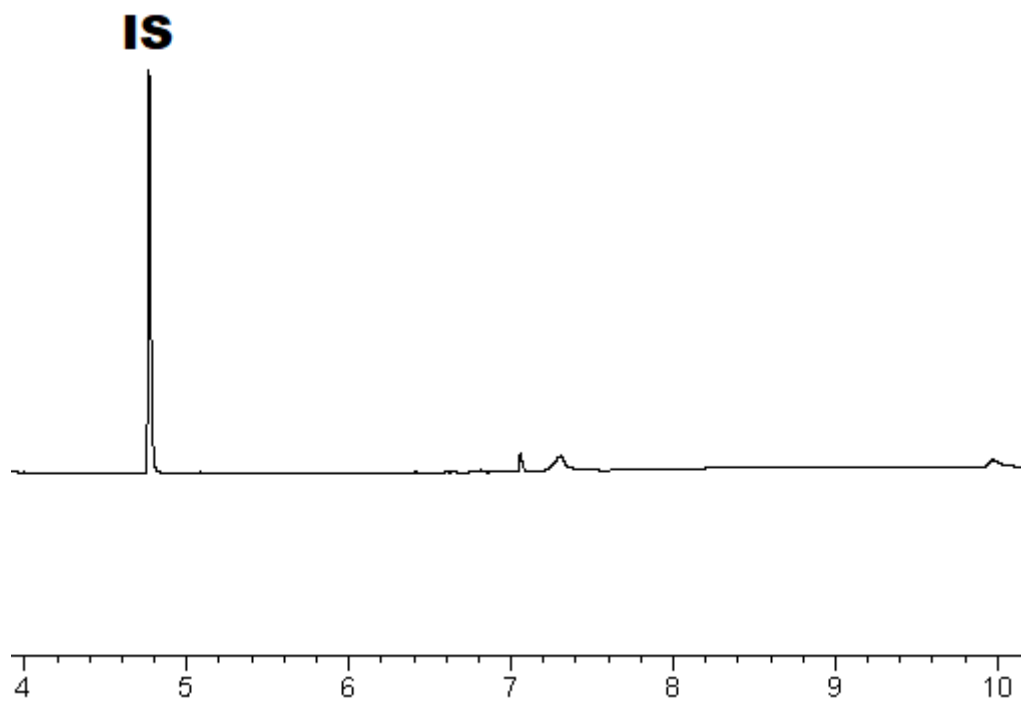

control experiment **(w/o capsule)**

Figure S-18: Comparison of GC traces (standard conditions and control experiment for **3ad)**, IS: internal standard (*n*-pentadecane)

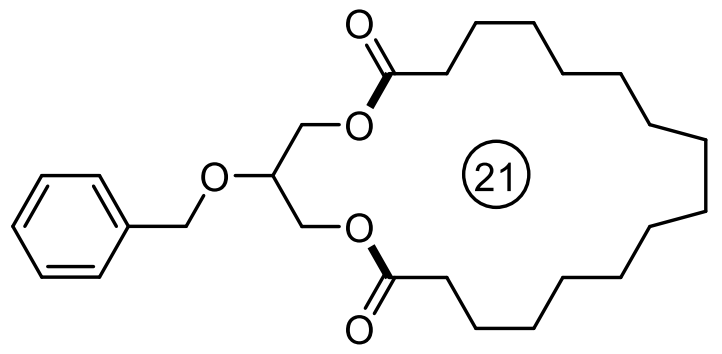

3ae

Control experiments forming **3ae** was performed by using 2-(benzyloxy)propane-1,3-diol (18.1 mg, 99.3 µmol), hexadecanedioyl dichloride (81.2 mg, 0.251 mmol), and Rb_2CO_3 (60.9 mg). 2.72 µmol loss (3%) of 2-(benzyloxy)propane-1,3-diol during transfer process was detected by ^1H NMR. Therefore, 96.6 µmol 2-(benzyloxy)propane-1,3-diol actually participated in the reaction. **Yield:** 0.7 mg, 2%

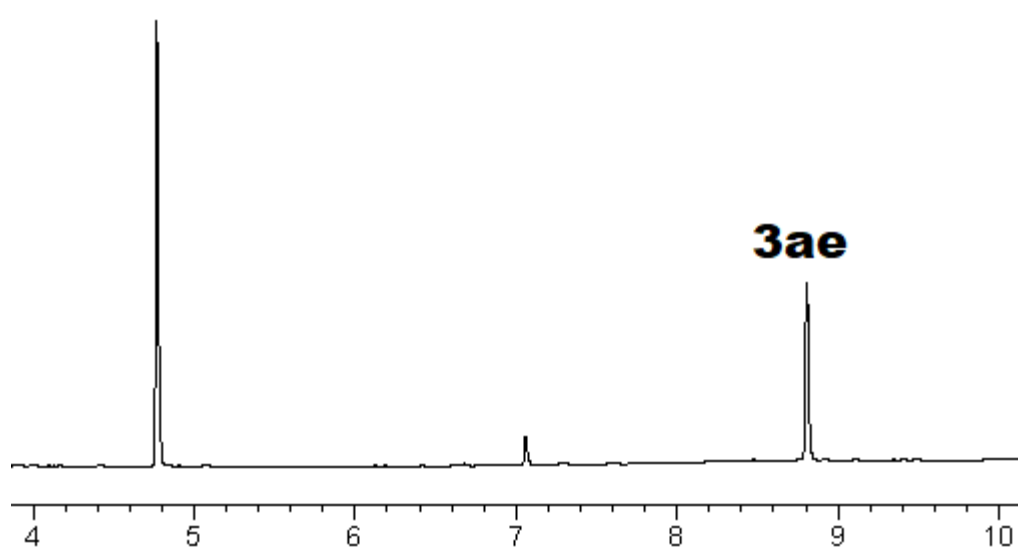

standard conditions **(w/ capsule)**

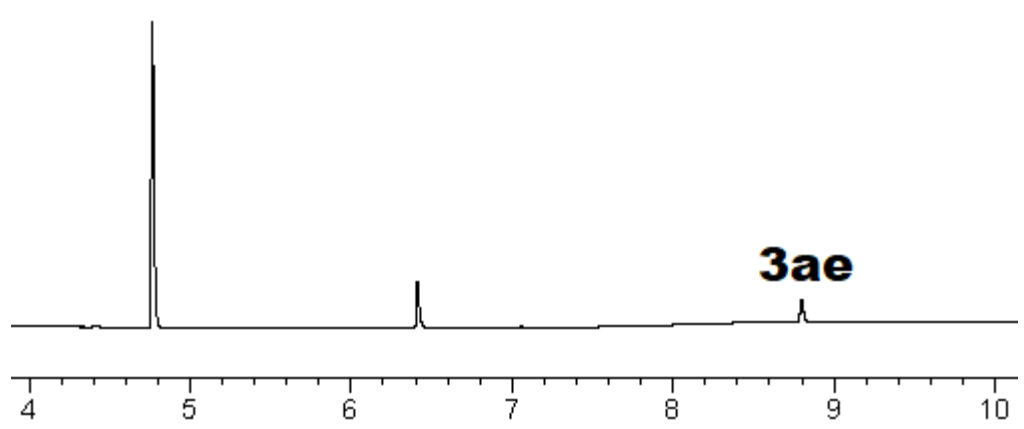

control experiment **(w/o capsule)**

Figure S-19: Comparison of GC traces (standard conditions and control experiment for **3ae)**, IS: internal standard (*n*-pentadecane)

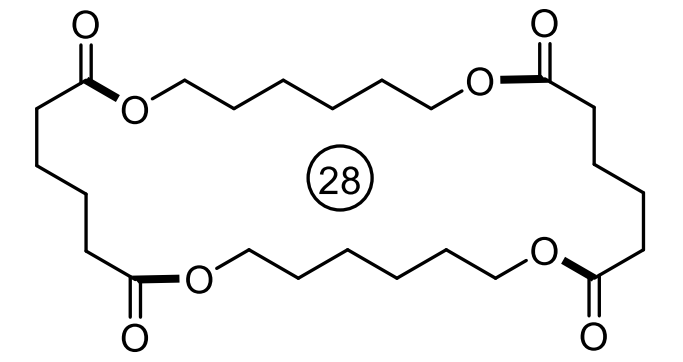

4a

Control experiments forming **4a** was performed by using hexane-1,6-diol (11.8 mg, 99.8 µmol), adipoyl dichloride (prepared from 147 mg adipic acid and 1/4 of the material applied, corresponding to 46.0 mg diacyl chloride, 0.251 mmol), and Rb_2CO_3 (61.5 mg). **4a** was not detected by GC in this control experiment.

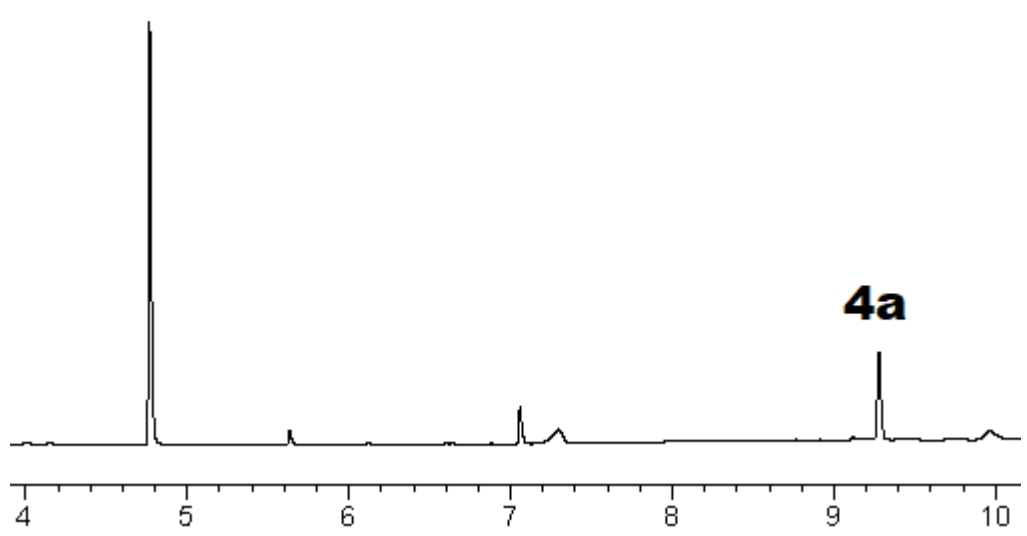

standard conditions **(w/ capsule)**

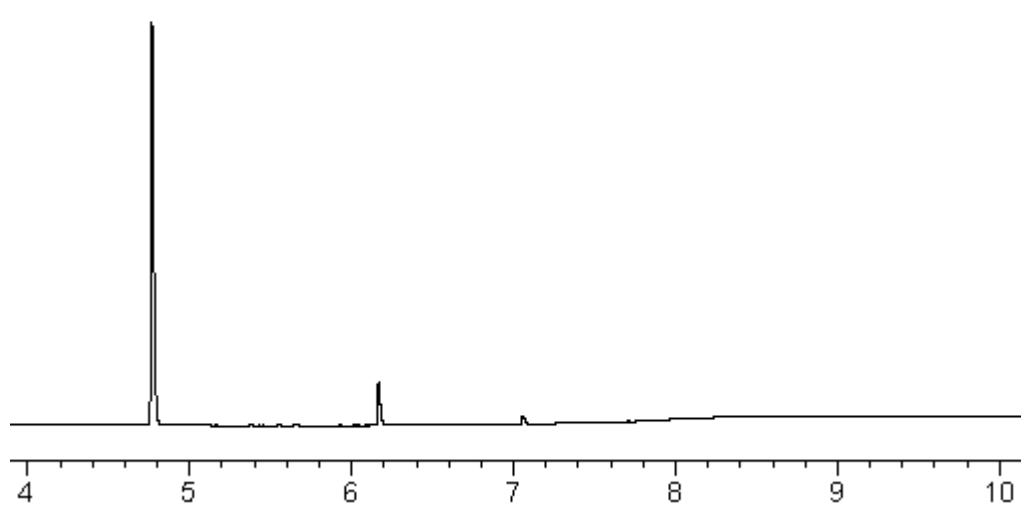

control experiment **(w/o capsule)**

Figure S-20: Comparison of GC traces (standard conditions and control experiment for **4a)**, IS: internal standard (*n*-pentadecane)

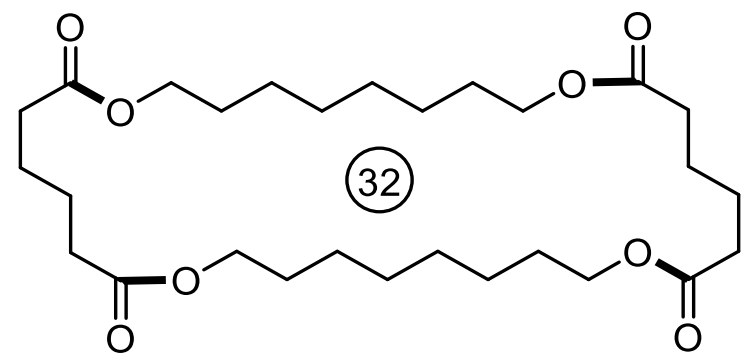

4b

Control experiments forming **4b** was performed by using octane-1,8-diol (14.8 mg, 101 µmol), adipoyl dichloride (prepared from 147 mg adipic acid and 1/4 of the material applied, corresponding to 46.0 mg diacyl chloride, 0.251 mmol), and Rb_2CO_3 (60.3 mg). Since **4b** was detected in only 3% GC yield, purification was not performed.

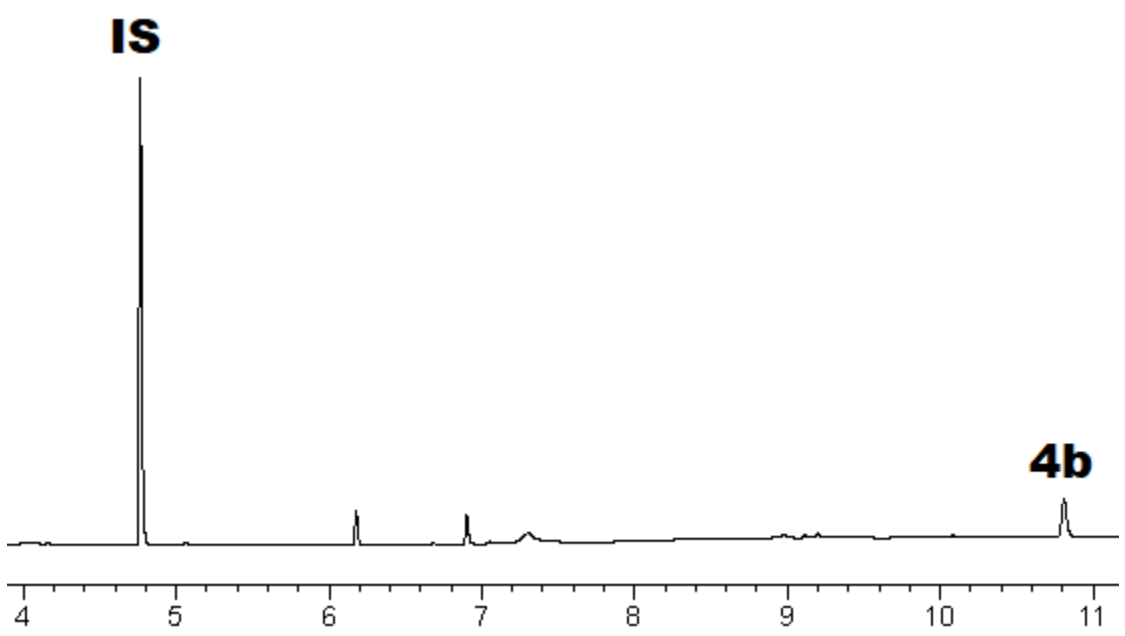

standard conditions **(w/ capsule)**

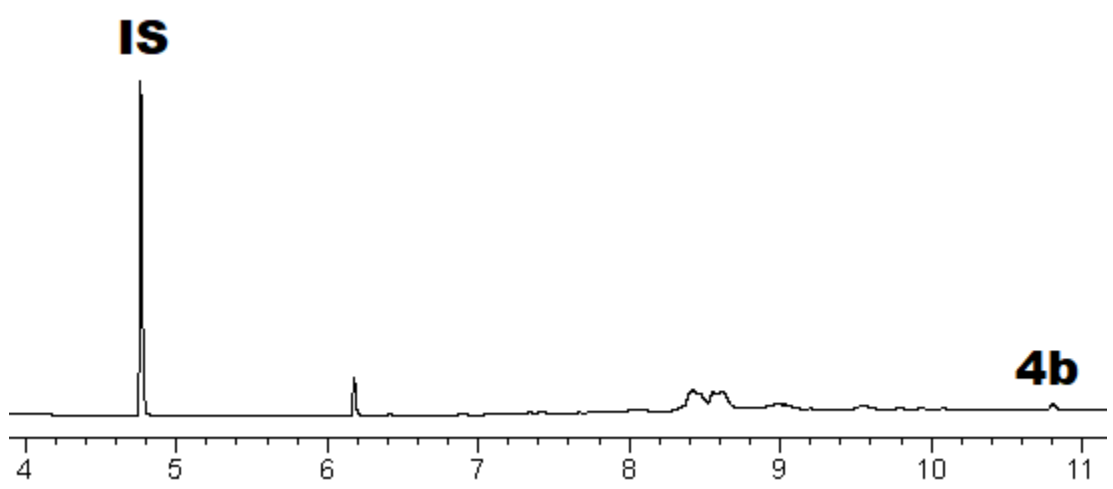

control experiment **(w/o capsule)**

Figure S-21: Comparison of GC traces (standard conditions and control experiment for **4b)**, IS: internal standard (*n*-pentadecane)

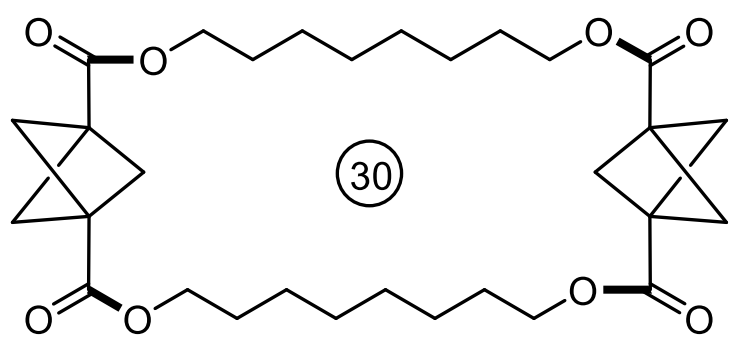

4c

Control experiments forming **4c** was performed by using octane-1,8-diol (14.6 mg, 99.9 µmol), bicyclo[1.1.1]pentane-1,3-dicarbonyl diacyl chloride (prepared from 158 mg bicyclo[1.1.1]pentane-1,3-dicarboxylic acid and 1/4 of the material applied, corresponding to 48.8 mg diacyl chloride, 0.253 mmol), and Rb_2CO_3 (60.4 mg). **4c** was not detected by GC in this control experiment.

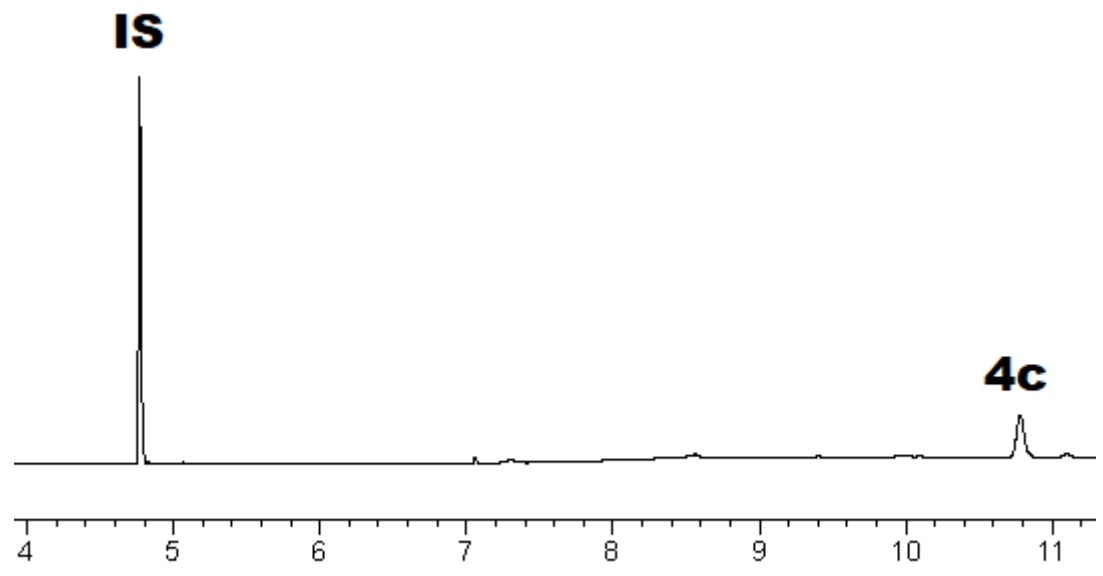

standard conditions **(w/ capsule)**

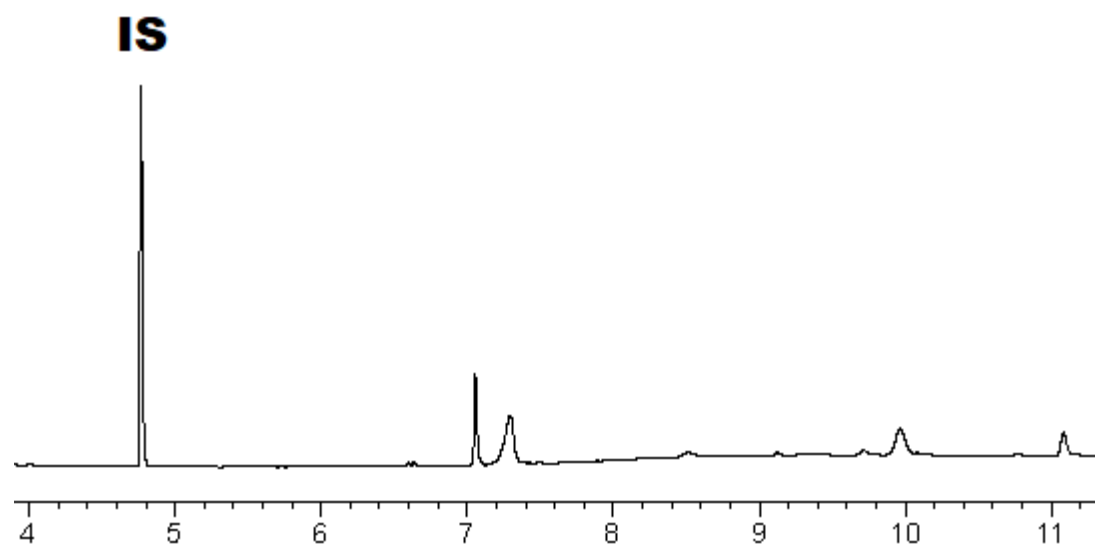

control experiment **(w/o capsule)**

Figure S-22: Comparison of GC traces (standard conditions and control experiment for **4c)**, IS: internal standard (*n*-pentadecane)

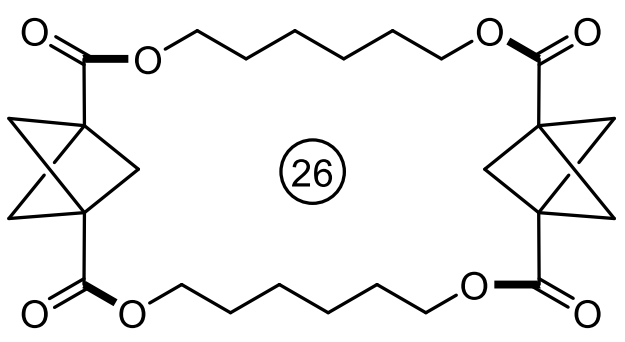

4d

Control experiments forming **4d** was performed by using hexane-1,6-diol (11.7 mg, 99.0 µmol), bicyclo[1.1.1]pentane-1,3-dicarbonyl diacyl chloride (prepared from 156 mg bicyclo[1.1.1]pentane-1,3-dicarboxylic acid and 1/4 of the material applied, corresponding to 48.2 mg diacyl chloride, 0.250 mmol), and Rb_2CO_3 (59.6 mg). Since **4d** was detected in only 2% GC yield, purification was not performed.

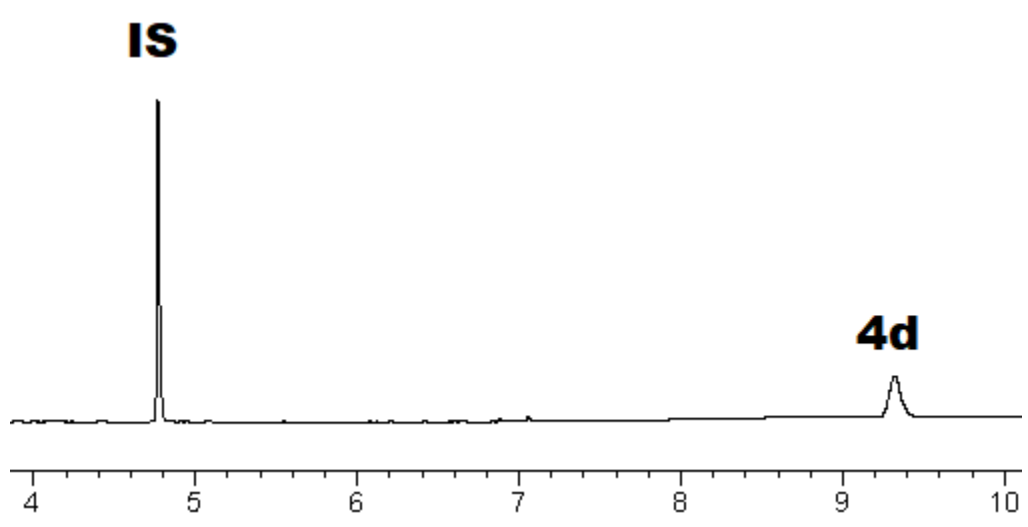

standard conditions **(w/ capsule)**

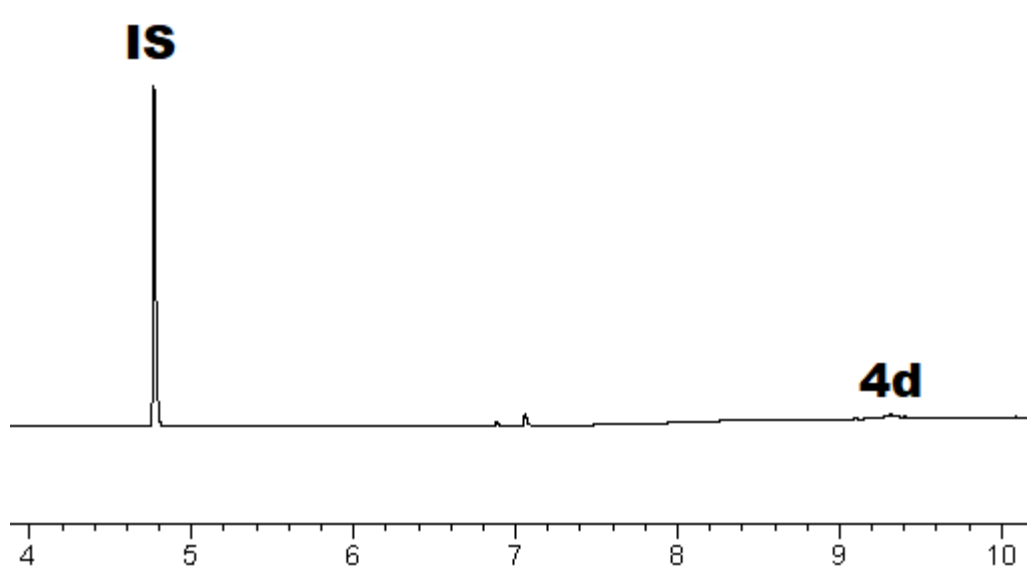

control experiment **(w/o capsule)**

Figure S-23: Comparison of GC traces (standard conditions and control experiment for **4d)**, IS: internal standard (*n*-pentadecane)

8. DOSY experiments

To study whether the resorcin[4]arene still assembles at the standard temperature (70 °C) applied in this work, we performed DOSY experiments[9] by comparing the diffusion coefficient (*D*) of C_{11}-resorcin[4]arene assembly (**I**)[10], C_{11}-pyrogallol[4]arene assembly (**II**)[11], and C_{11}-tetramethoxyresorcin[4]arene (C_4-symmetric)[12].

Materials and Methods

Self-diffusion measurements were performed on different samples of calixarenes using a Bruker Avance III NMR spectrometer operating 600.13 MHz proton frequency. The instrument was equipped with a 5-mm broadband indirect BBI probe with a shielded z-gradient coil and a GAB gradient amplifier (10 Ampere, maximum gradient strength 52.5 G/cm). A convection compensated double stimulated echo pulse sequence (dstegp3s) by Jerschow et al. was used[13], avoiding 180° pulses and bipolar gradients for optimum performance at elevated temperature. The analytes were dissolved in toluene-d8 (99.94 % D, Eurisotop, France) at 90 mM and a 7 inch long, 3 mm NMR tube (o.d. 2.97 mm, i.d. 2.37 mm, Deutero GmbH, Germany) was filled to a height of exactly 40 mm with the sample. This 3 mm tube was inserted into a slightly shortened round bottom 5 mm tube (Wilmad, USA), through an exactly 3 mm wide hole drilled into the plastic cap. The 5 mm tube had been filled with ca. 230 µL toluene-d8 beforehand, so that the space between the inner 3 mm tube and the outer 5 mm tube was homogeneously filled to exactly 40 mm height with pure solvent as a heat exchanger. Applying a thermal equilibration time of 15 min prior to each experiment, this setup delivered much more consistent results in our hands than a simple 5 mm tube, which suffers from severe turbulent convection artefacts.

The diffusion experiments were performed at 298 K or 343 K, and the temperature was calibrated using a glycerol standard showing accuracy within +/- 0.2 K. The gradient strength was calibrated using a Shigemi tube filled with H_2O to a height of 4.0 mm and imaging this water cylinder[14].

The diffusion experiments were performed by varying the gradient strength between 5% and 95% of the maximum strength in 16 single experiments using an acquisition time of 2 s while keeping the diffusion times and gradient lengths constant. The diffusion time was set to 120 ms or 60 ms (big delta, for 298 K and 343 K resp.) and a gradient duration of 2.0 ms was applied (little delta). The intensity decrease of the signal of interest was determined and fitted in a two parameter fit with the Bruker t1/t2 software package suitable for dosy experiments, which is included in the topspin 3.6 software[15]. The decay curve for a representative resonance (the Ha of the lauryl substituent) was plotted together with the resulting diffusion coefficients, the residuals squared sum (RSS) and the standard deviation (SD) given as: SD = SQRT(RSS/n). Furthermore, the datasets were processed in a two-dimensional fashion using the *dosy2d* function of topspin after applying zero-filling to 512(64 k) points in the F1(F2) dimension.

The results were listed below.

Table S12. Diffusion coefficient (10^{-10} m^2/s) obtained from DOSY experiments

T (K)	resorcinarene capsule (**I**)	pyrogallolarene capsule (**II**)	tetramethoxyresorcinarene
298	1.71	1.95	3.86
343	8.80	9.61	13.6

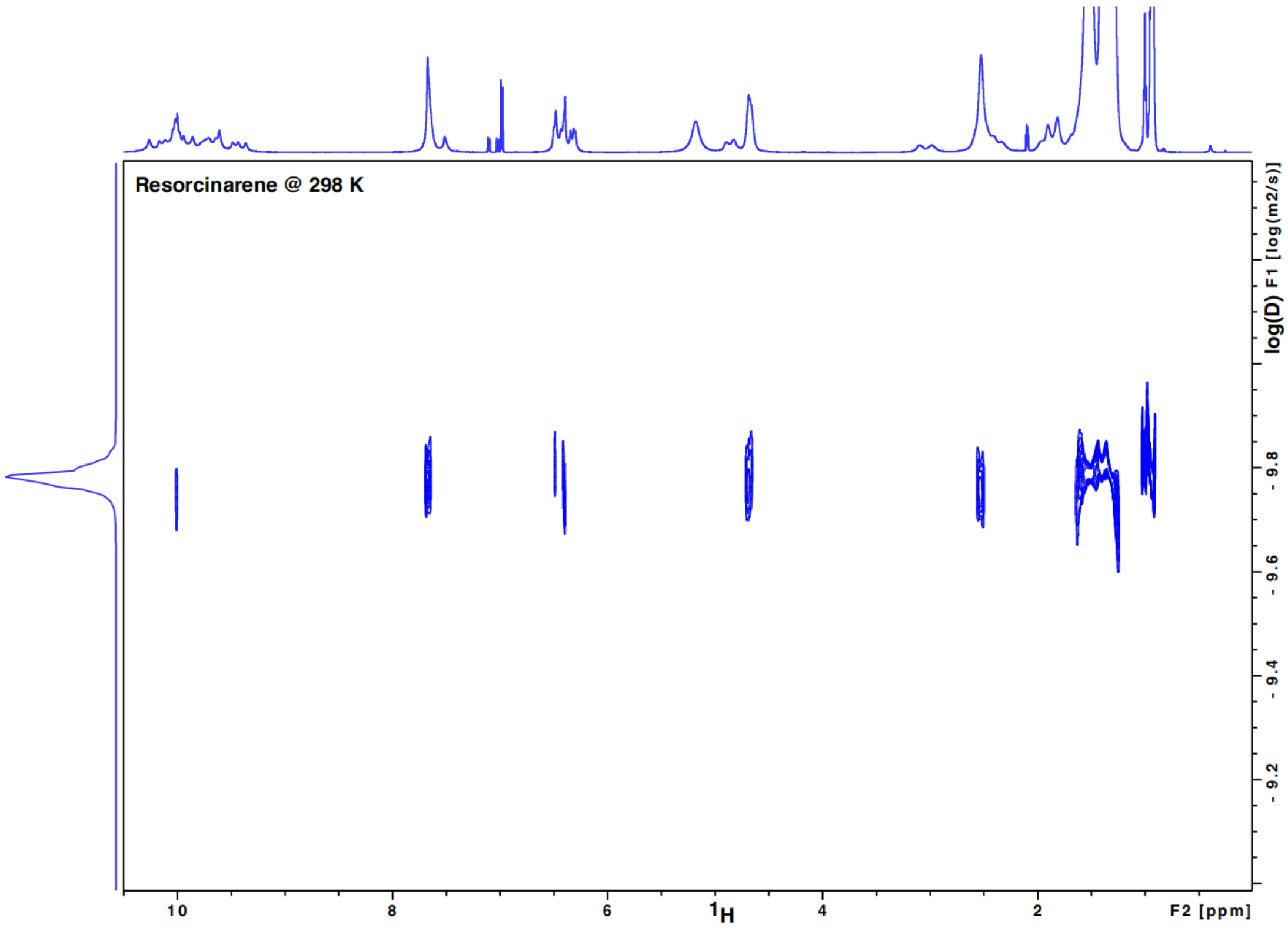

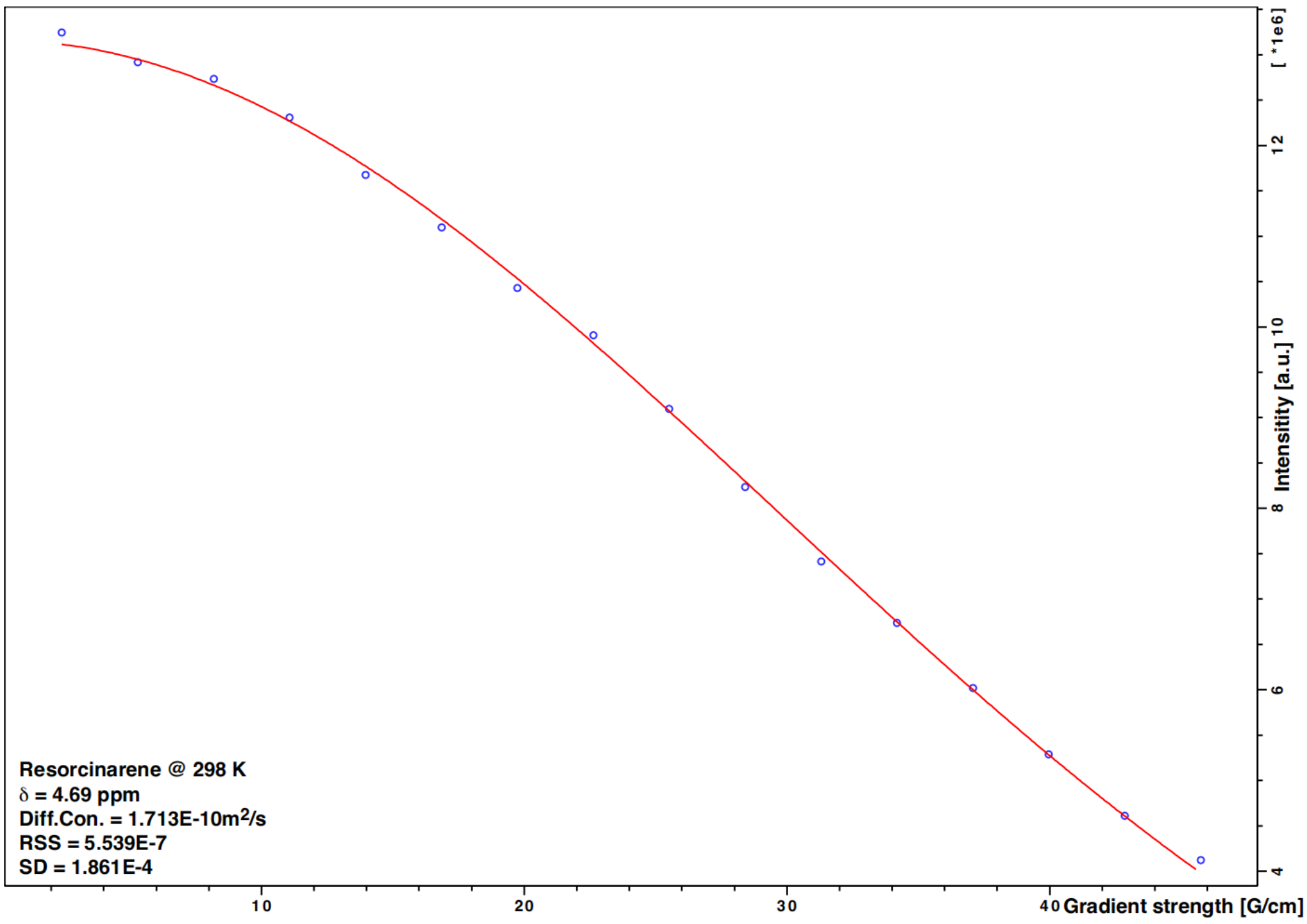

Figure S-24: DOSY-NMR (600 MHz, toluene-d_8, 298 K) of C_{11}-resorcinarene

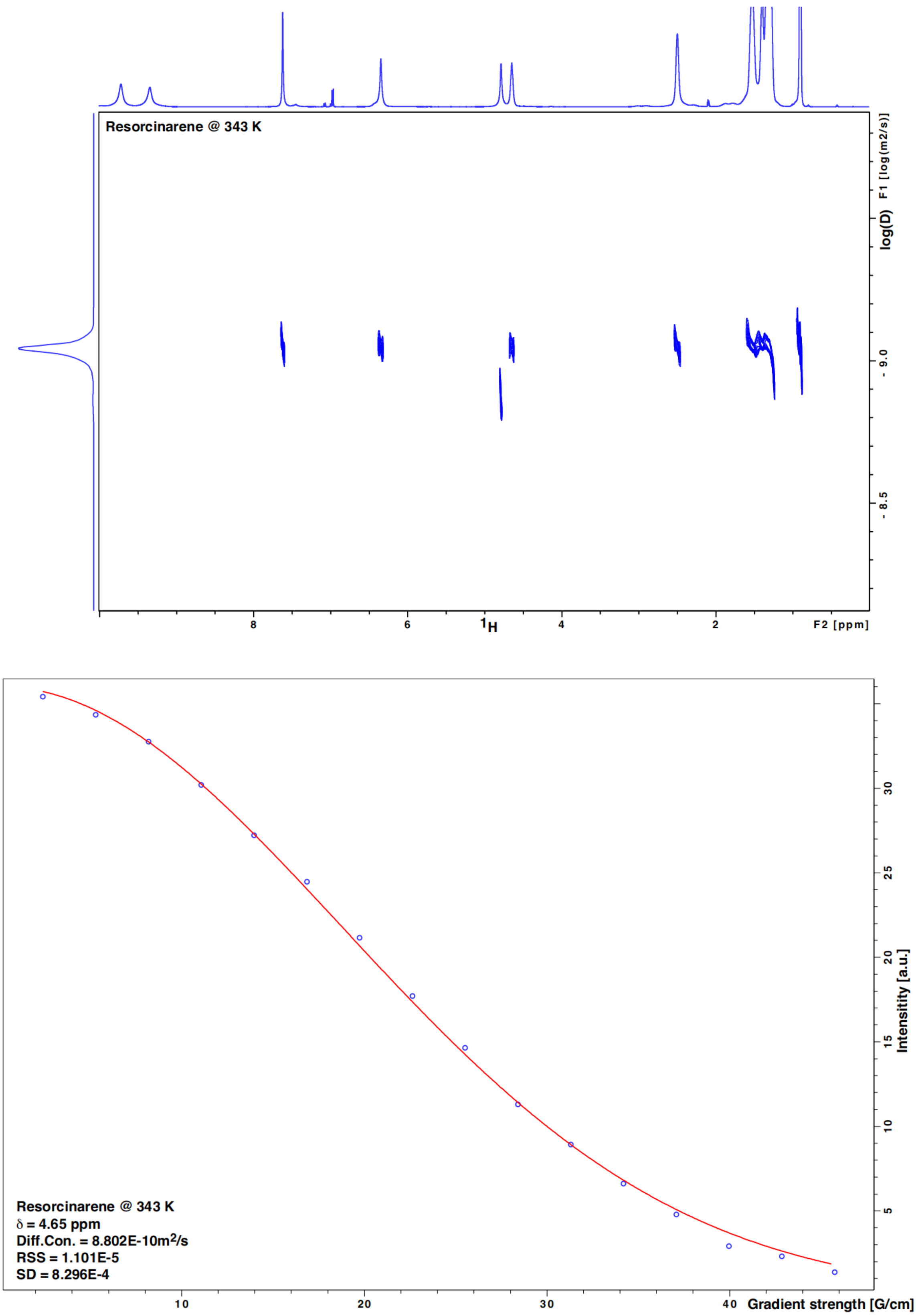

Figure S-25: DOSY-NMR (600 MHz, toluene-d_8, 343 K) of C_{11}-resorcinarene

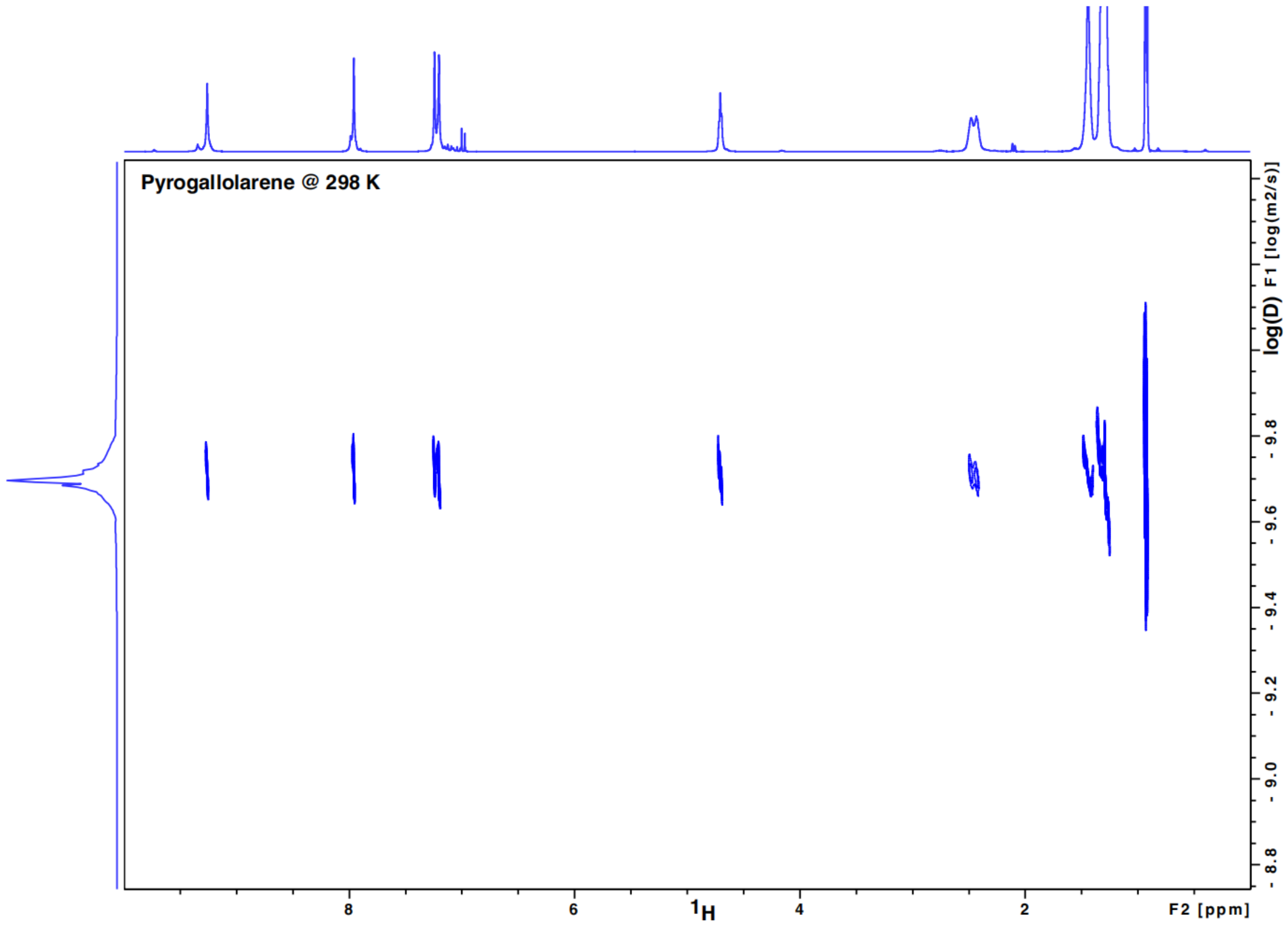

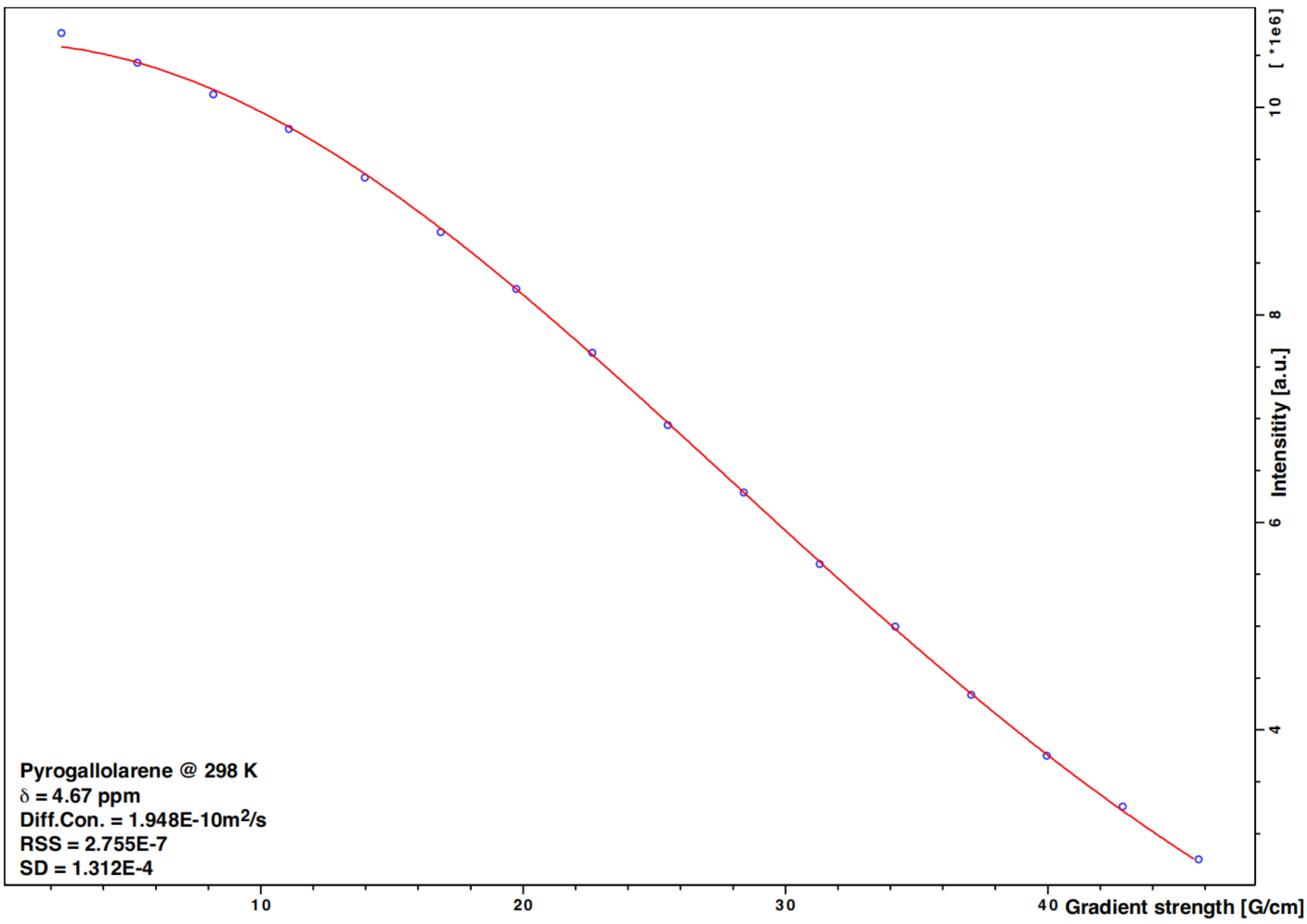

Figure S-26: DOSY-NMR (600 MHz, toluene-d_8, 298 K) of C_{11}-pyrogallolarene

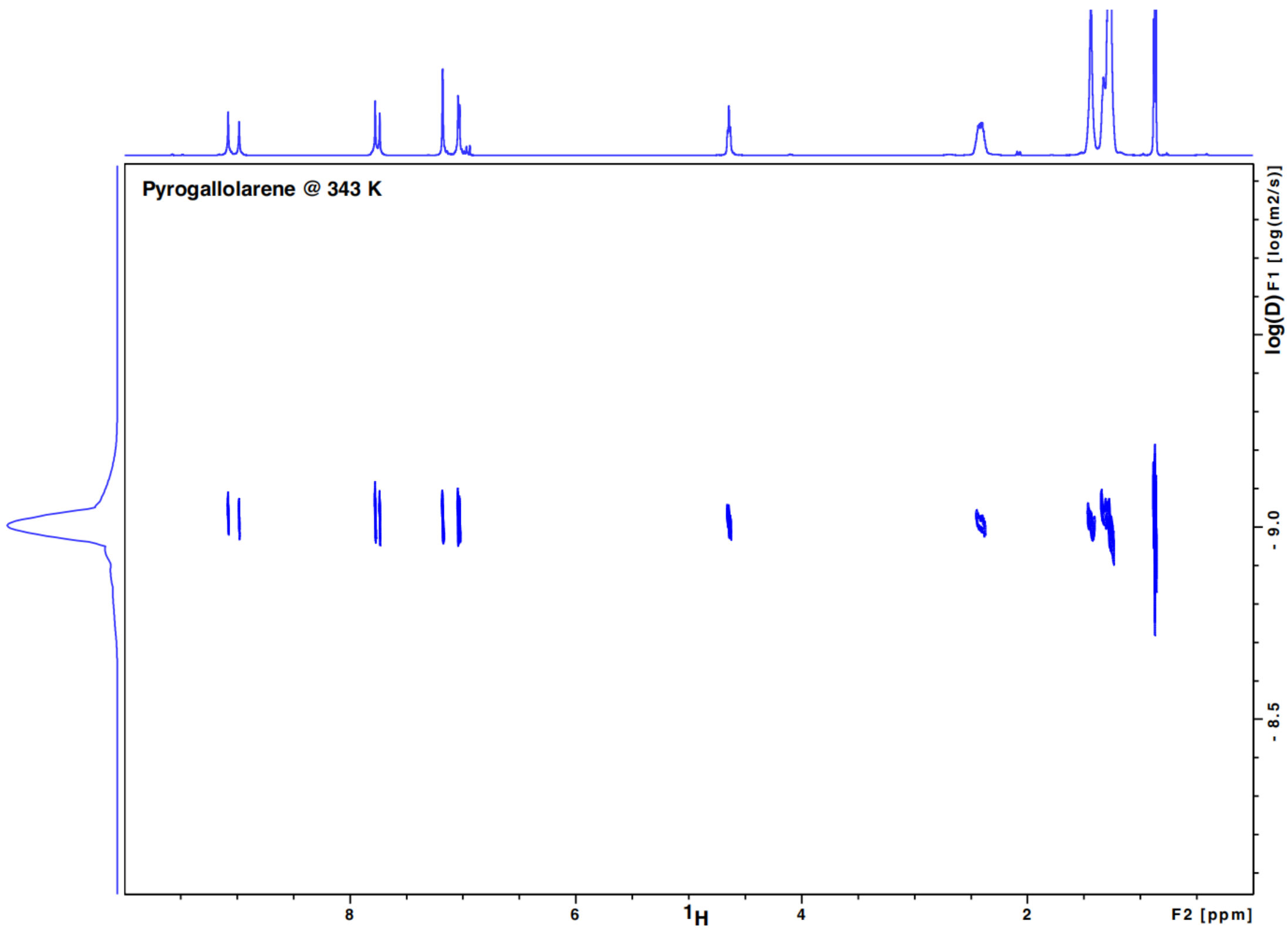

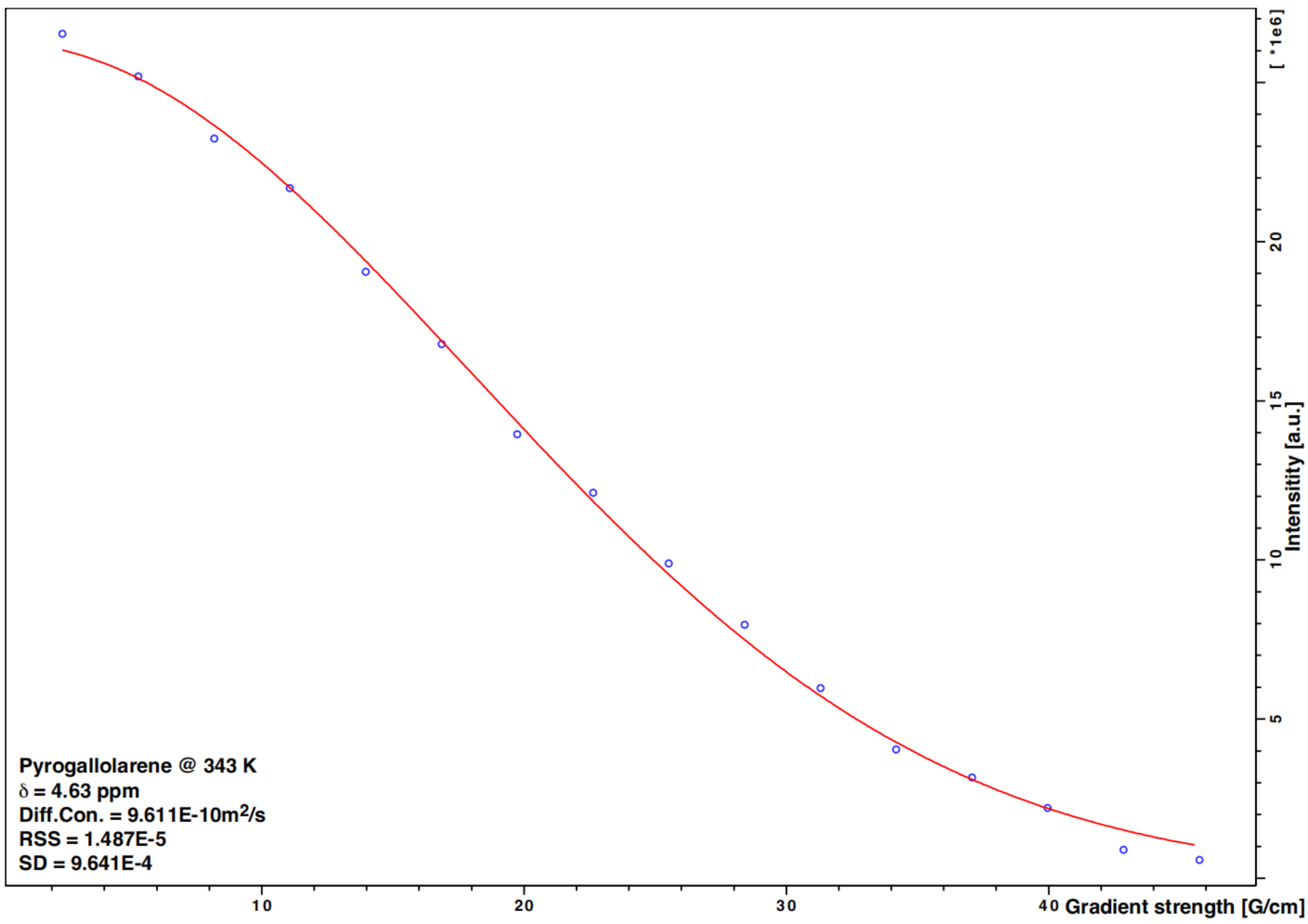

Figure S-27: DOSY-NMR (600 MHz, toluene-d_8, 343 K) of C_{11}-pyrogallolarene

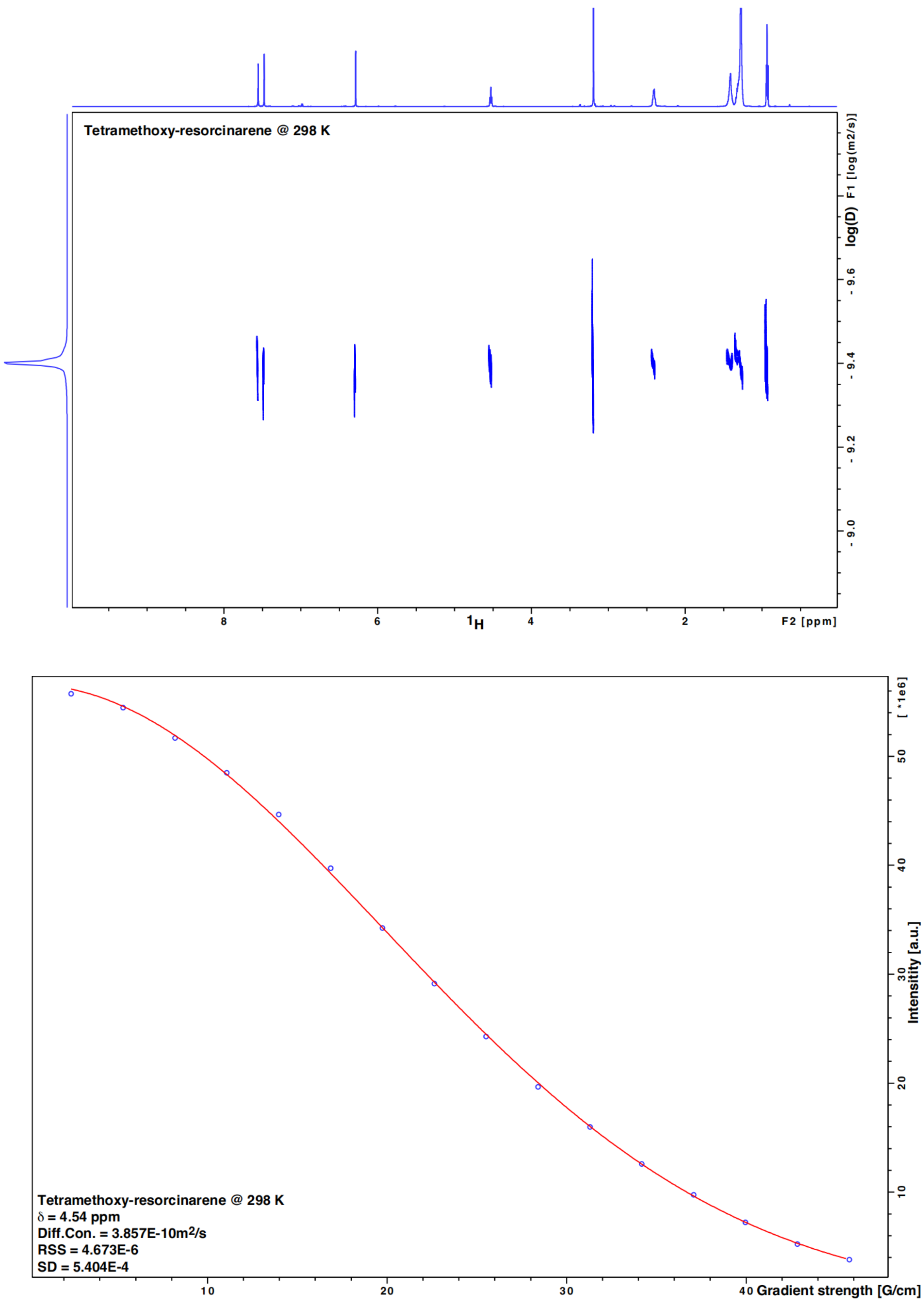

Figure S-28: DOSY-NMR (600 MHz, toluene-d_8, 298 K) of C_{11}-tetramethoxyresorcinarene

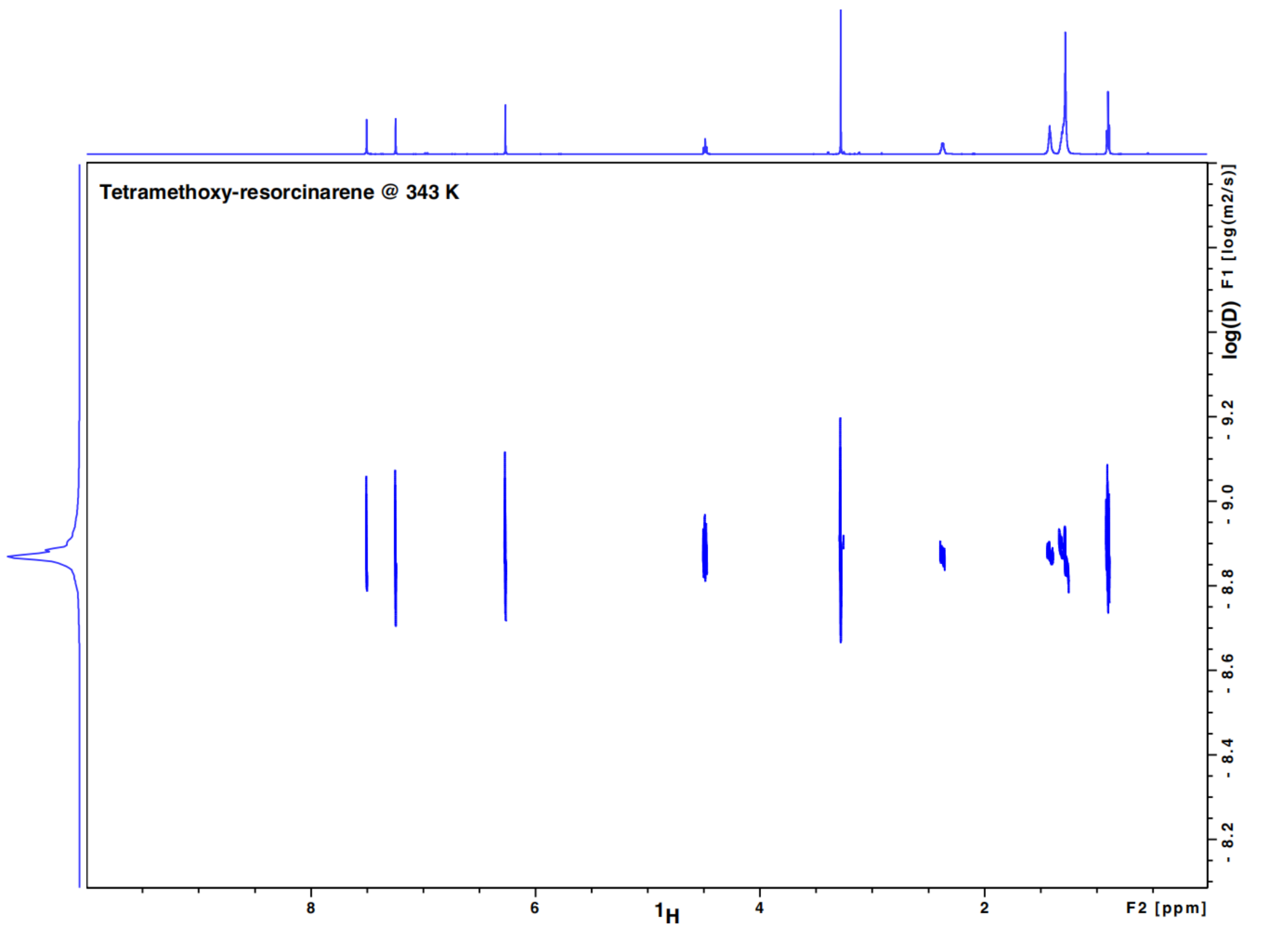

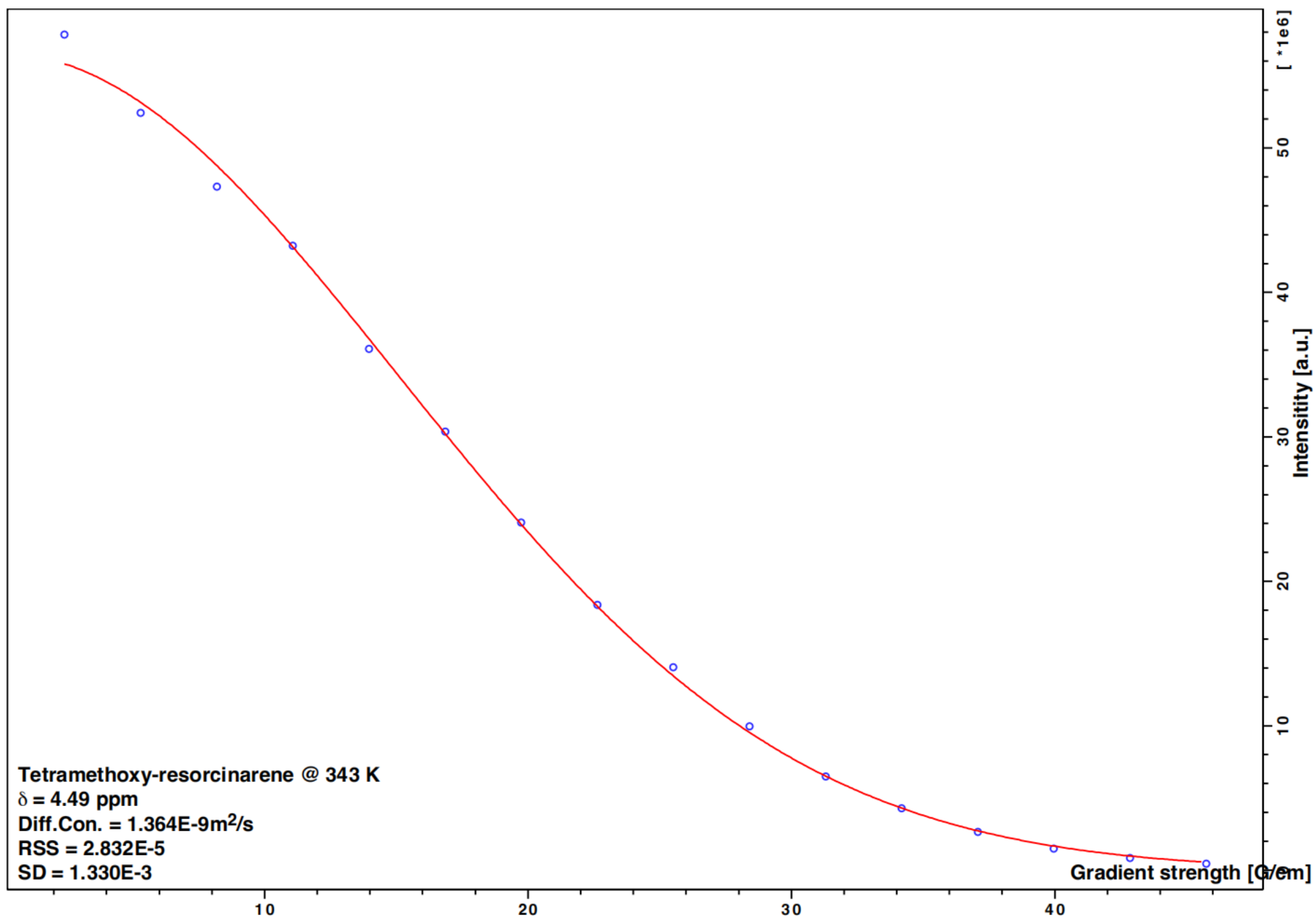

Figure S-29: DOSY-NMR (600 MHz, toluene-d_8, 343 K) of C_{11}-tetramethoxyresorcinarene

9. Kinetic studies

Kinetic analysis of an intermolecular model reaction

Following general procedure B, 0.5 mmol-scale reactions between cyclohexanol (**6**) and decanoyl chloride (**7**) were performed in 100 mL Schlenk tube and monitored by ^{1}H NMR (3 measurements for each condition). To facilitate monitoring, internal standard (IS) tetraethylsilane (1.00 equiv) was added to Solution A directly.

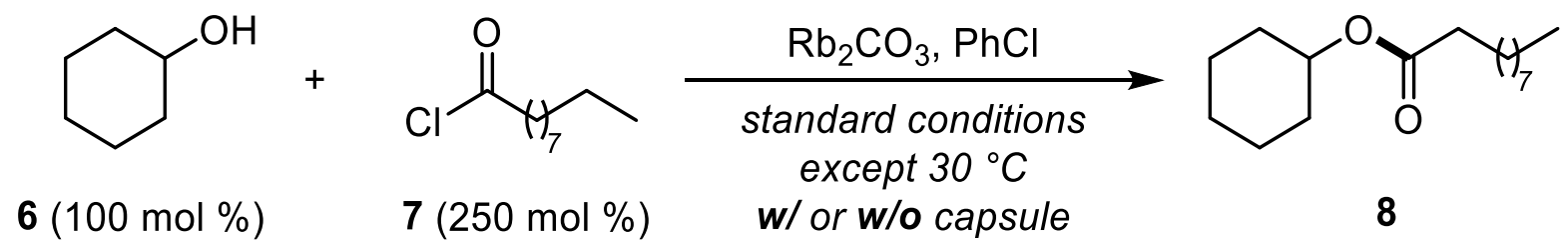

For preparing NMR samples, an aliquot (ca. 30 μL) of the reaction mixture was taken by syringe, diluted with 0.55 mL of acetone-d_6, and filtered into NMR tubes (an example of crude ^{1}H NMR given below).

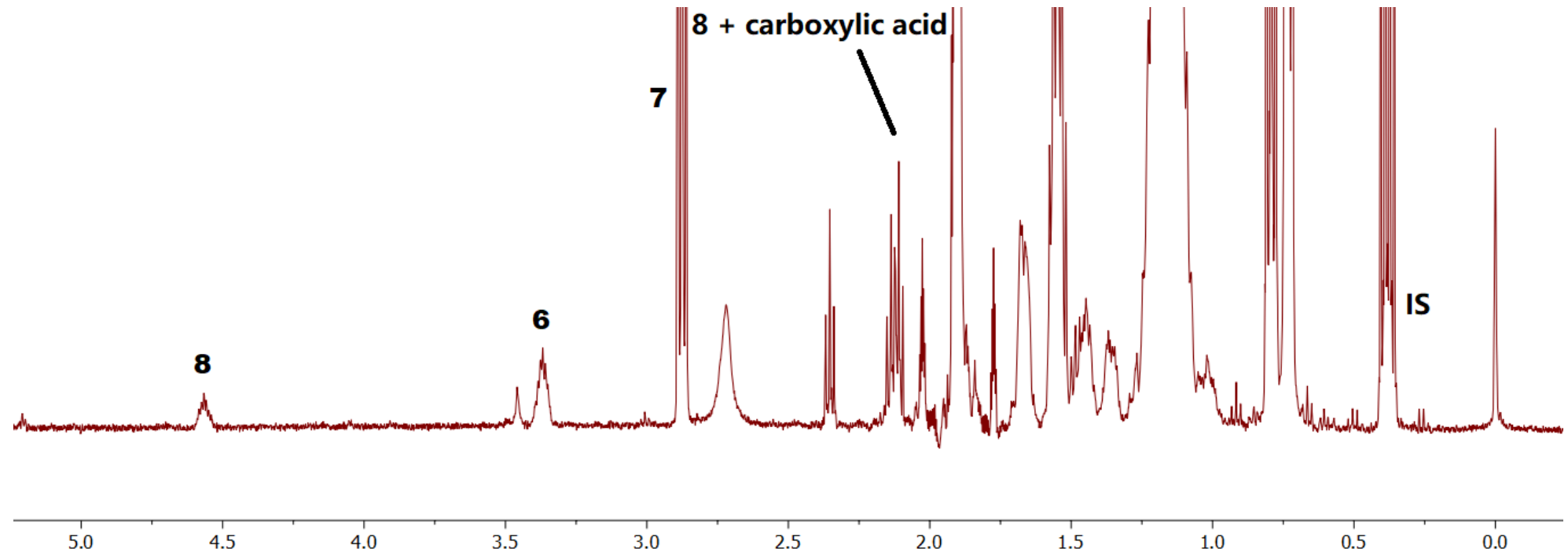

Figure S-30: A typical crude ^{1}H NMR of the reaction between **6** and **7** (w/o capsule **I**)

Table S13. Kinetic data **w/** capsule **I** (50 mM for **6**, 125 mM for **7**)[a]

t (min)	**6** (1)	**6** (2)	**6** (3)	MEAN	SEM	**7** (1)	**7** (2)	**7** (3)	MEAN	SEM	**8** (1)	**8** (2)	**8** (3)	MEAN	SEM
0	100	100	100	100.0	0.00	250	250	250	250.0	0.00	0	0	0	0.0	0.00
10	89	90	91	90.0	0.58	205	215	212	210.7	2.96	10	9	9	9.3	0.33
20	81	85	83	83.0	1.15	181	196	188	188.3	4.33	18	14	15	15.7	1.20
30	75	78	79	77.3	1.20	176	178	181	178.3	1.45	23	21	21	21.7	0.67
40	68	74	73	71.7	1.86	164	158	171	164.3	3.76	28	25	26	26.3	0.88
50	64	67	68	66.3	1.20	153	150	154	152.3	1.20	32	32	30	31.3	0.67
60	60	61	62	61.0	0.58	147	134	144	141.7	3.93	36	36	36	36.0	0.00
70	56	52	51	53.0	1.53	138	123	131	130.7	4.33	40	39	40	39.7	0.33
80	51	47	45	47.7	1.76	134	117	123	124.7	4.98	44	44	45	44.3	0.33
90	42	41	39	40.7	0.88	128	109	109	115.3	6.33	48	47	51	48.7	1.20
120	30	31	31	30.7	0.33	110	100	92	100.7	5.21	59	57	59	58.3	0.67
180	17	20	21	19.3	1.20	68	84	66	72.3	5.60	72	67	66	68.3	1.86
240	8	10	10	9.3	0.67	59	62	46	55.7	4.91	83	78	75	78.7	2.33
1440	6	8	7	7.0	0.58	0	0	0	0.0	0.00	81	78	79	79.3	0.88

[a] unit of kinetic data: **mol %**. SEM = Standard Error of the Mean.

Table S14. Kinetic data **w/o** capsule **I** (50 mM for **6**, 125 mM for **7**)[a]

t (min)	**6** (1)	**6** (2)	**6** (3)	MEAN	SEM	**7** (1)	**7** (2)	**7** (3)	MEAN	SEM	**8** (1)	**8** (2)	**8** (3)	MEAN	SEM
0	100	100	100	100.0	0.00	250	250	250	250.0	0.00	0	0	0	0.0	0.00
10	96	93	92	93.7	1.20	235	240	240	238.3	1.67	6	6	6	6.0	0.00
20	90	89	89	89.3	0.33	229	233	236	232.7	2.03	9	8	9	8.7	0.33
30	86	86	87	86.3	0.33	222	218	232	224.0	4.16	13	12	11	12.0	0.58
40	81	83	84	82.7	0.88	216	213	228	219.0	4.58	17	15	14	15.3	0.88
50	77	77	82	78.7	1.67	214	210	224	216.0	4.16	21	18	16	18.3	1.45
60	73	74	76	74.3	0.88	208	206	219	211.0	4.04	24	22	20	22.0	1.15
70	72	69	73	71.3	1.20	205	203	215	207.7	3.71	28	25	23	25.3	1.45
80	70	66	70	68.7	1.33	200	199	207	202.0	2.52	31	29	26	28.7	1.45
90	66	63	66	65.0	1.00	194	189	198	193.7	2.60	34	32	30	32.0	1.15
120	58	57	59	58.0	0.58	188	176	184	182.7	3.53	38	36	36	36.7	0.67
180	50	48	46	48.0	1.15	182	169	174	175.0	3.79	45	44	44	44.3	0.33
240	40	36	37	37.7	1.20	171	155	160	162.0	4.73	55	56	51	54.0	1.53
1440	0	0	0	0.0	0.00	114	62	114	96.7	17.33	83	83	86	84.0	1.00

[a] unit of kinetic data: **mol %**. SEM = Standard Error of the Mean.

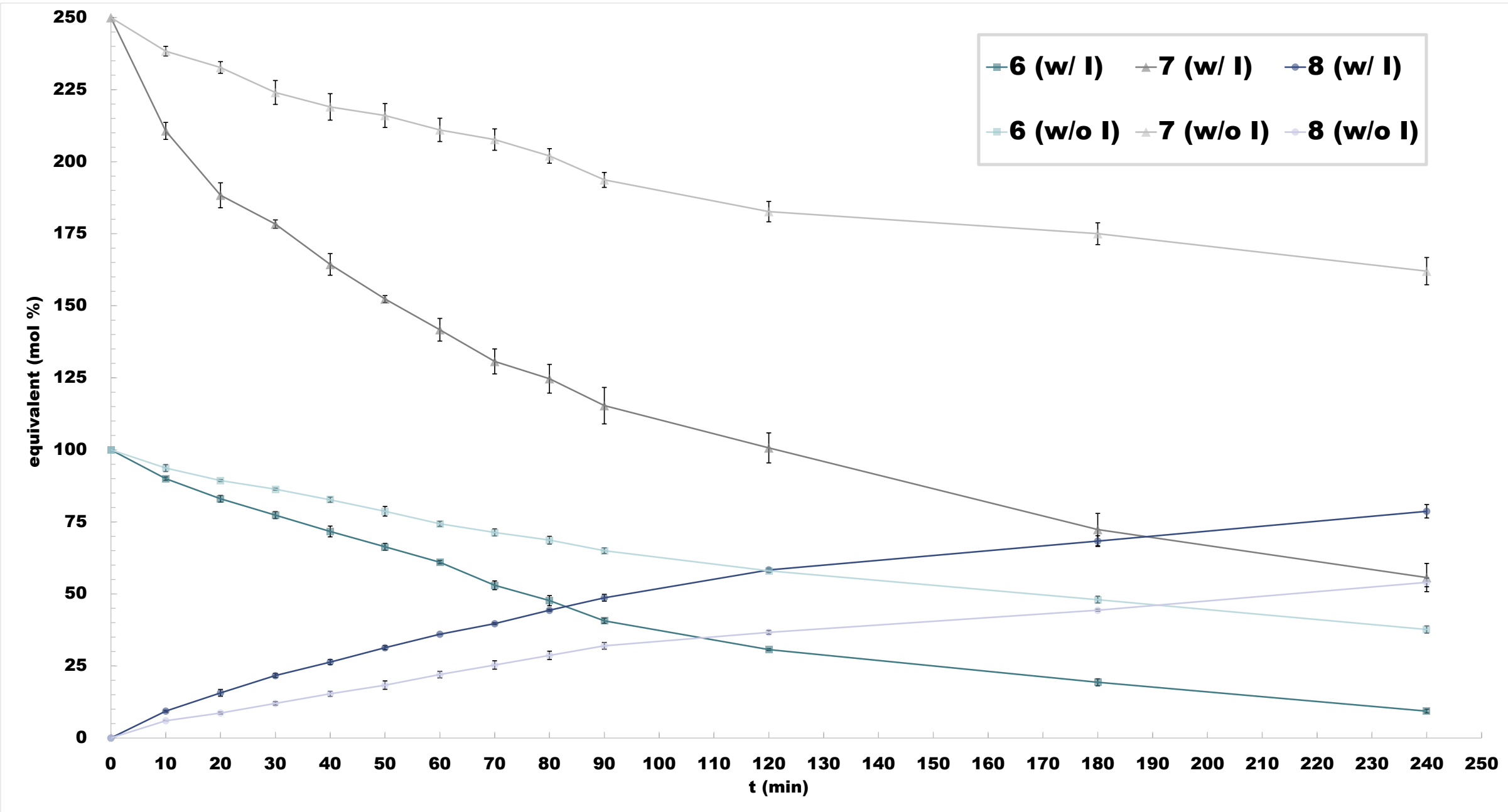

Figure S-31: Kinetic profile for the reaction between **6** and **7** (w/ and w/o capsule **I**)

Initial rates were calculated according to the generation of **8**. Linear fitting of initial spots (until ca. 20% average yields of **8** reached: 0-30 min, w/ capsule; 0-60 min, w/o capsule) and comparison of the slopes gave the rate acceleration attributed to capsule **I** (0.7133/0.3476 ≈ 2.0, see below).

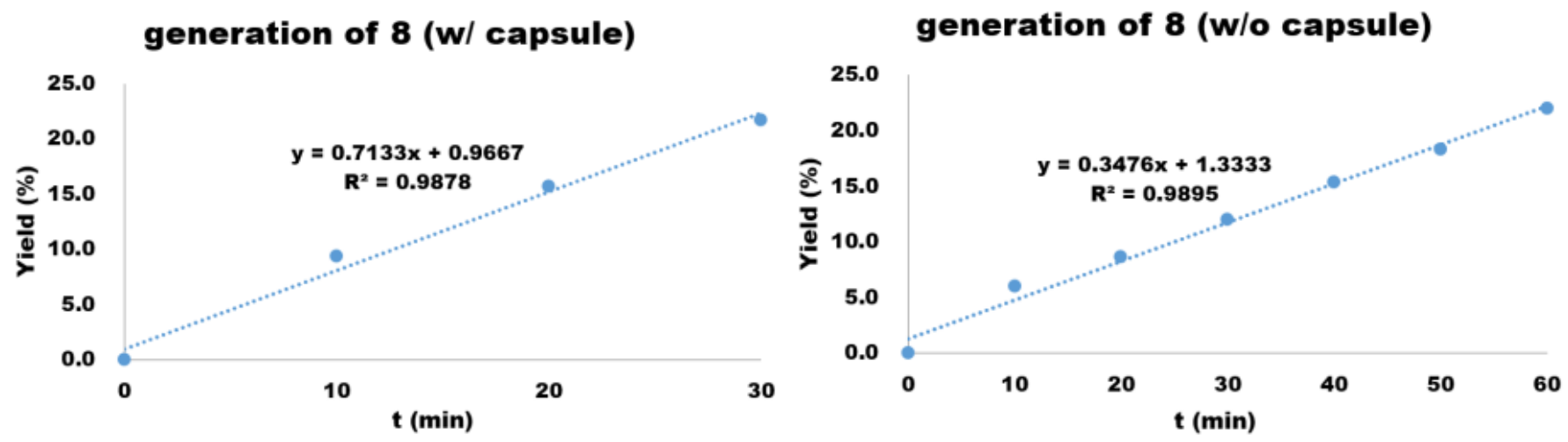

Figure S-32: Comparing the initial rates (w/ capsule and w/o capsule)

By varying the concentration of **6** from 50 mM to 75 mM, the reaction order with respect to **6** was determined using variable time normalization analysis (VTNA)[16].

Table S15. Kinetic data **w/** capsule **I** (75 mM for **6**, 125 mM for **7**)[a]

t (min)	**6** (1)	**6** (2)	**6** (3)	MEAN	SEM	**7** (1)	**7** (2)	**7** (3)	MEAN	SEM	**8** (1)	**8** (2)	**8** (3)	MEAN	SEM
0	75.0	75.0	75.0	75.0	0.00	125.0	125.0	125.0	125.0	0.00	0.0	0.0	0.0	0.0	0.00
10	68.5	67.0	69.0	68.2	0.60	113.2	113.8	112.7	113.2	0.32	5.0	5.5	4.0	4.8	0.44
20	64.5	63.0	66.5	64.7	1.01	104.0	105.0	109.8	106.3	1.79	9.5	10.0	7.0	8.8	0.93
30	60.5	61.5	60.0	60.7	0.44	101.2	96.8	104.0	101.6	2.10	12.0	12.5	10.5	11.7	0.60
40	56.5	59.5	57.5	57.8	0.88	94.0	93.8	99.8	94.9	1.97	14.5	15.5	13.5	14.5	0.58
50	53.5	55.5	56.0	55.0	0.76	91.0	91.0	94.5	92.2	1.17	17.0	17.5	17.0	17.2	0.17
60	52.0	53.0	54.0	53.0	0.58	89.0	85.2	85.2	86.5	1.27	19.5	20.0	19.0	19.5	0.29
70	50.0	49.5	51.5	50.3	0.60	80.7	77.0	79.5	79.1	1.09	21.0	22.0	22.0	21.7	0.33
80	48.0	46.0	50.0	48.0	1.15	75.5	72.5	76.5	74.8	1.20	22.5	24.0	23.5	23.3	0.44
90	46.0	44.0	47.0	45.7	0.88	62.8	70.2	73.2	68.7	3.09	24.0	26.0	26.5	25.5	0.76
120	42.5	40.5	41.0	41.3	0.60	49.0	68.0	69.5	62.2	6.60	29.0	31.0	31.0	30.3	0.67
180	34.5	33.5	30.5	32.8	1.20	24.5	48.0	50.0	40.8	8.19	35.5	37.5	40.5	37.8	1.45
240	30.0	27.5	26.0	27.8	1.17	9.8	19.0	36.0	21.6	7.67	41.5	41.5	43.5	42.2	0.67
1440	26.5	27.0	24.0	25.8	0.93	0.0	0.0	0.0	0.0	0.00	42.0	42.0	43.0	42.3	0.33

[a] unit of kinetic data: **mM**. SEM = Standard Error of the Mean.

Table S16. Kinetic data **w/o** capsule **I** (75 mM for **6**, 125 mM for **7**)[a]

t (min)	**6** (1)	**6** (2)	**6** (3)	MEAN	SEM	**7** (1)	**7** (2)	**7** (3)	MEAN	SEM	**8** (1)	**8** (2)	**8** (3)	MEAN	SEM
0	75.0	75.0	75.0	75.0	0.00	125.0	125.0	125.0	125.0	0.00	0.0	0.0	0.0	0.0	0.00
10	69.5	65.5	68.5	67.8	1.20	120.2	119.2	119.5	119.6	0.30	5.0	5.5	5.0	5.2	0.17
20	67.0	63.0	63.0	64.3	1.33	116.8	117.5	116.8	117.0	0.23	7.5	8.5	7.5	7.8	0.33
30	63.5	60.0	61.0	61.5	1.04	113.0	114.5	113.8	113.8	0.43	10.5	11.0	11.0	10.8	0.17
40	60.0	56.5	58.0	58.2	1.01	110.2	108.5	109.8	109.5	0.51	14.0	14.0	13.5	13.8	0.17
50	57.0	55.0	54.5	55.5	0.76	105.8	106.2	105.0	105.7	0.35	17.0	16.5	15.5	16.3	0.44
60	54.0	53.0	52.5	53.2	0.44	103.0	105.5	103.8	104.1	0.74	19.0	18.5	19.0	18.8	0.17
70	51.0	50.5	50.0	50.5	0.29	94.8	104.0	101.0	99.9	2.71	21.5	20.5	21.0	21.0	0.29
80	48.5	48.0	48.0	48.2	0.17	90.8	101.2	99.0	97.0	3.16	24.0	22.0	23.0	23.0	0.58
90	46.5	45.5	44.0	45.3	0.73	85.0	98.2	97.5	93.6	4.29	26.5	24.0	24.0	24.8	0.83
120	41.5	38.0	40.0	39.8	1.01	83.2	96.8	93.0	91.0	4.05	32.0	29.0	28.5	29.8	1.09
180	35.0	30.0	31.0	32.0	1.53	75.2	90.5	90.8	85.5	5.15	39.0	36.5	36.5	37.3	0.83
240	32.0	27.5	22.0	27.2	2.89	71.2	84.2	87.2	80.9	4.91	42.5	41.0	40.5	41.3	0.60
1440	13.0	4.0	4.0	7.0	3.00	46.5	29.0	22.0	32.5	7.29	55.0	65.5	61.0	60.5	3.04

[a] unit of kinetic data: **mM**. SEM = Standard Error of the Mean.

Table S17. Normalization of the reaction time by VTNA[a]

t (min)	*t'* (50 mM for **6**, w/ **I**)	*t'* (75 mM for **6**, w/ **I**)	*t'* (50 mM for **6**, w/o **I**)	*t'* (75 mM for **6**, w/o **I**)
0	0.00	0.00	0.00	0.00
10	19.28	20.67	3117.40	5541.45
20	38.25	41.08	5984.08	10481.55
30	56.98	61.28	8682.38	15075.37
40	75.47	81.30	11230.53	19339.94
50	93.73	101.15	13609.46	23291.90
60	111.74	120.85	15808.81	26989.32
70	129.41	140.40	17853.96	30437.75
80	146.72	159.79	19782.46	33642.90
90	163.64	179.02	21583.26	36602.79
120	212.60	236.00	26360.01	44336.65
180	304.78	346.89	34025.77	56359.00
240	388.64	454.06	39619.01	65380.96

[a] w/ **I**, order (**6**) = 0.17 applied for calculation; w/o **I**, order (**6**) = 1.48 applied for calculation.

Kinetic study of the highest-yielding reaction

Following general procedure B, 0.1 mmol-scale reactions between 1,18-octadecanediol (**9**) and bicyclo[1.1.1]pentane-1,3-dicarbonyl dichloride (**10**) were performed in 5 mL Schlenk tube and monitored by both ^{1}H NMR (for the conversion of **9** and **10**) and GC (for the yield of **3aa**). To facilitate monitoring, internal standard (IS) tetraethylsilane (for ^{1}H NMR, 1.00 equiv) and *n*-pentadecane (for GC, 1.00 equiv) were added to Solution A directly.

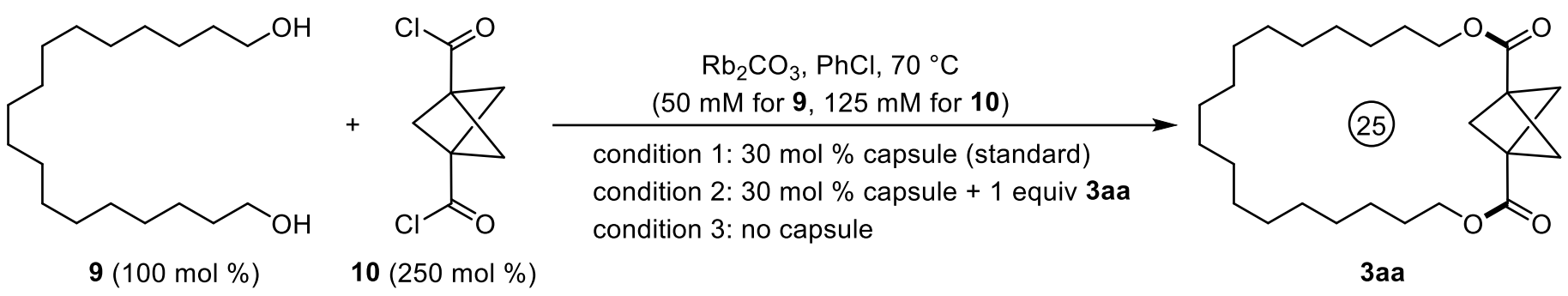

For preparing NMR samples, an aliquot (ca. 30 μL) of the reaction mixture was taken by syringe, diluted with 0.50 mL of acetone-d_6, and filtered into NMR tubes (an example of crude spectra given below, standard conditions, 10 min). GC samples were prepared by following the same method in Section 3.

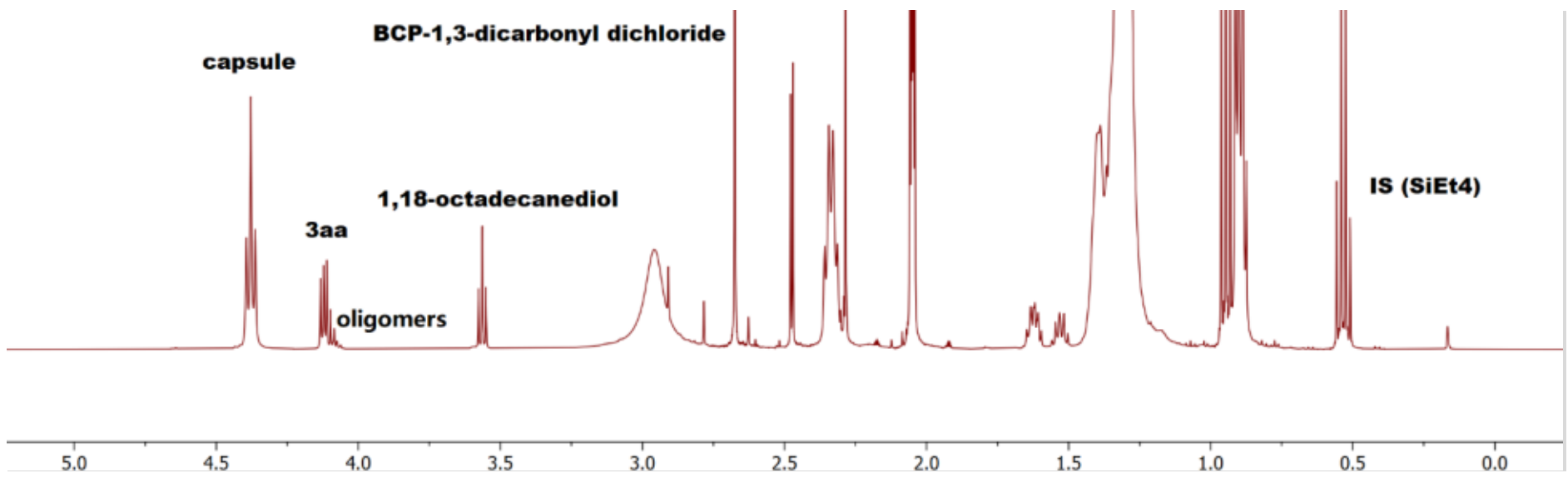

Figure S-33: A typical crude ^{1}H NMR of the reaction generating **3aa** (w/ capsule **I**, 10 min)

Table S18. Kinetic data of the reaction generating **3aa**[a]

t	30 mol % **I**			30 mol % **I** + 100 mol % **3aa**			no capsule		
(min)	**9**	**10**	**3aa**	**9**	**10**	**3aa**	**9**	**10**	**3aa**
0	100	250	0	100	250	100	100	250	0
5	67	130	28	73	136	117	34	80.2	3.5
10	43	114	47	54	123	127	18	69.3	5.2
15	31	107	61	43	117	142	13	62.5	5.6
30	12	75.7	77	21	91.8	160	5	61.1	7.3
45	6	59.3	81	12	88.5	172	4	59.7	7.4
60	4	55.0	84	6	71.0	170	2	56.5	7.7
90	1	46.0	83	2	49.3	170	1	16.7	7.9

[a] unit of kinetic data: **mol %**.

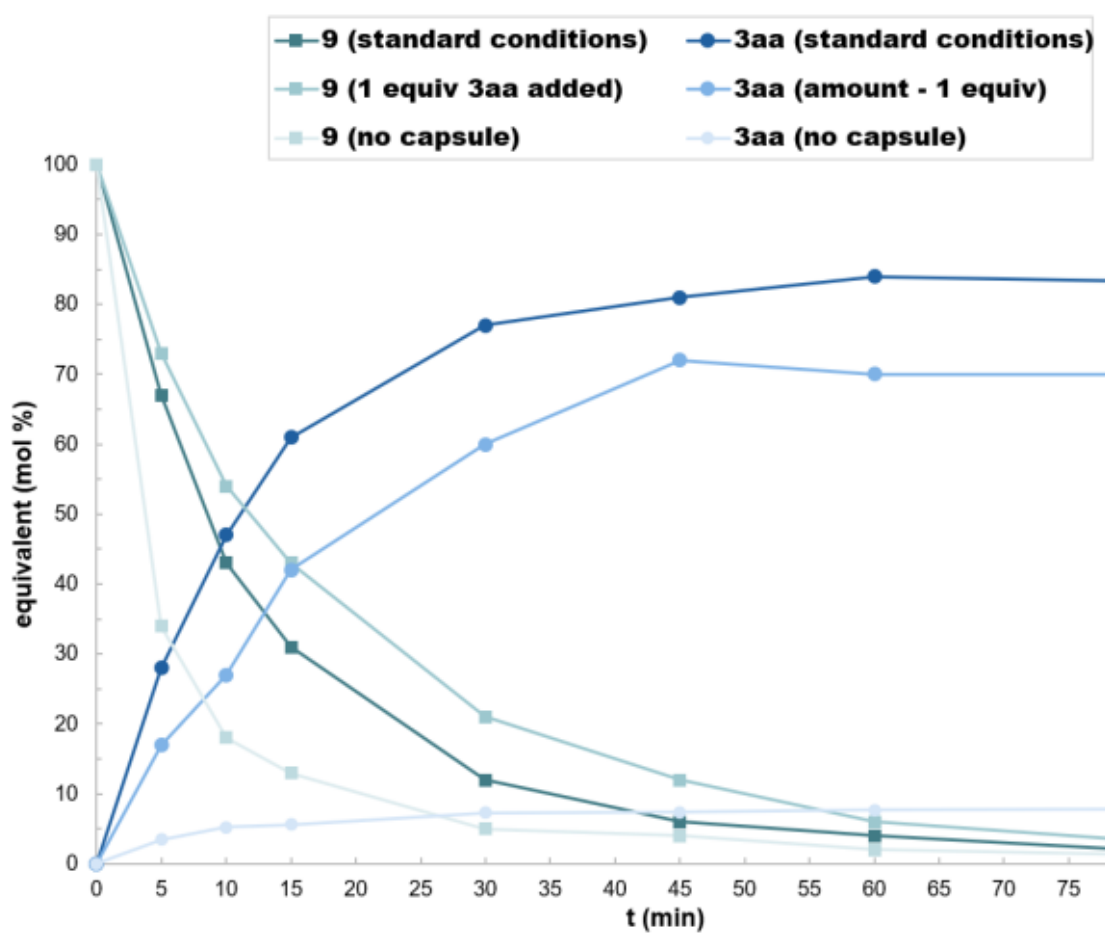

The product inhibition was evaluated by the initial rate of generating **3aa** (standard conditions vs. 1 equiv **3aa** added to standard conditions, 0-5 min).

To evaluate the promotion of macrocyclization inside the capsule, we proposed the kinetic model below.

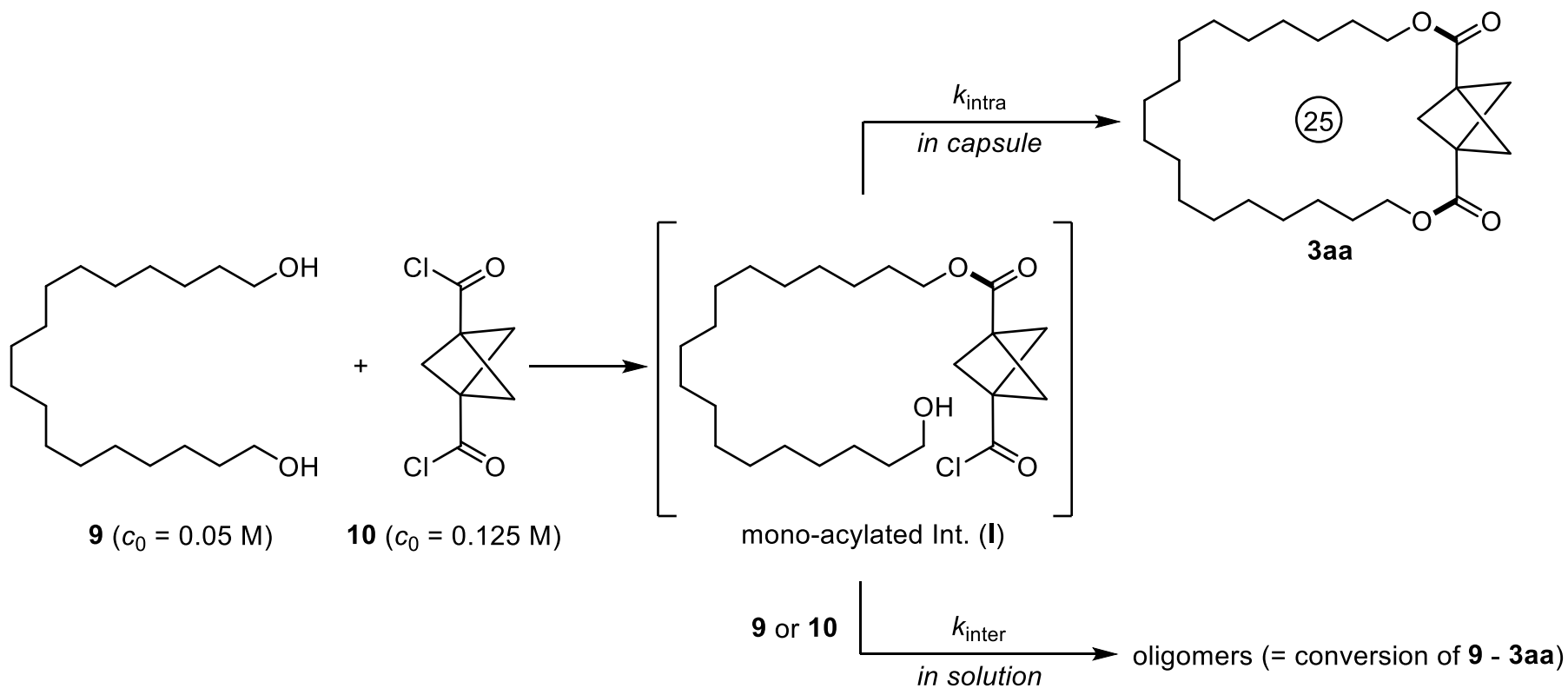

Figure S-34: Kinetic model for estimating the effective molarity (EM)

According to this kinetic model, the effective molarity (EM = $k_{\text{intra}}/k_{\text{inter}}$) was estimated from the product distribution observed within the first 5 min under standard conditions.

$$\frac{yield\ (\mathbf{3aa})_{t=5\,min}}{yield\ (oligomers)_{t=5\,min}} = \frac{28\%}{33\% - 28\%} \approx \frac{initial\ rate_{I\rightarrow\mathbf{3aa}}}{initial\ rate_{I\rightarrow oligomers}} =$$

$$\frac{k_{intra}[I]}{k_{inter}\,[I](c_0(\mathbf{9}) + c_0(\mathbf{10}))} = \frac{EM}{0.175\ M}$$

$$EM = 0.175\ M \times \frac{28\%}{33\% - 28\%} = 0.98\ M$$

If the competing intermolecular pathway is alternatively considered with the total concentration of reactive functional groups rather than the molecular concentration of the substrates, a higher EM value would be obtained.

The kinetic experiment under the standard conditions also helps to explain why no net depletion of the capsule-structural water is expected, although acid chloride hydrolysis occurs as a side reaction in the reaction system.

$ClOC\text{-}C_n\text{-}COCl + \mathbf{2\ H_2O} \rightarrow HOOC\text{-}C_n\text{-}COOH + 2\ HCl$ (i)

$2\ HCl + Rb_2CO_3 \rightarrow 2\ RbCl + CO_2\uparrow + \mathbf{H_2O}$ (ii)

$HOOC\text{-}C_n\text{-}COOH + Rb_2CO_3 \rightarrow RbOOC\text{-}C_n\text{-}COORb + \mathbf{H_2O} + CO_2\uparrow$ (iii)

(i) + (ii) + (iii) gives: $ClOC\text{-}C_n\text{-}COCl + 2\ Rb_2CO_3 \rightarrow 2\ RbCl + RbOOC\text{-}C_n\text{-}COORb + 2\ CO_2\uparrow$

This suggests that H_2O is not consumed in the net stoichiometry following this sequence. Thus, in the standard reaction conditions, Rb_2CO_3 (~ 60 mg for a 0.1 mmol reaction, 2.6 equiv) would in principle permit hydrolysis of up to 1.3 equiv of diacyl chloride without net loss of water from the system.

The formation of the macrodiolide also consumes 1 equiv of Rb_2CO_3 but generates 1 equiv of water:

$ClOC\text{-}C_n\text{-}COCl + HO\text{-}C_m\text{-}OH + Rb_2CO_3 \rightarrow \text{macrodiolide} + 2\ RbCl + \mathbf{H_2O} + CO_2\uparrow$ (iv)

The resulting $RbOOC\text{-}C_n\text{-}COORb$ in (iii) could further act as an HCl scavenger if Rb_2CO_3 is fully consumed, thereby regenerating $HOOC\text{-}C_n\text{-}COOH$. By monitoring this highest-yielding reaction (Table S18), when product **3aa** reached ~ 80% yield, ~ 0.6 equiv of the diacyl chloride remained, indicating 1.9 equiv consumption from an initial 2.5 equiv. Subtracting the 0.8 equiv incorporated into **3aa** formation leaves 1.1 equiv attributed to hydrolysis (at most, due to minor incorporation into oligomers), which is below the estimated 1.3 equiv threshold.

10. Binding studies

Binding constants of the compounds involved in the highest-yielding example (i.e., 1,18-octadecanediol (**9**), bicyclo[1.1.1]pentane-1,3-dicarbonyl diacyl chloride (**10**), and macrodiolide **3aa**) were measured by ^{1}H NMR (500 MHz, toluene-d_8, 298K). The integral of host signal (capsule **I**, δ = 5.10 – 4.33 ppm, 24 H, marked light-purple) was used as the internal reference to calculate the concentration of free guest [G] (key peaks marked red). Concentration of host-guest complex [HG]= $[G]_0$-[G], concentration of free host [H] = $[H]_0$-[HG] and K = [HG] / ([G] × [H]) were calculated accordingly.

(1) Binding constant of **9** and capsule **I**

In a 2 mL vial charged with a stir bar, diol **9** (2.31 mg, 8.06 µmol) and C_{11}-resorcin[4]arene (50.9 mg, 46.0 µmol, corresponding to 7.67 µmol capsule) was dissolved in 500 µL toluene-d_6 and stirred until a clear solution was formed. Then the solution was transferred into an NMR tube for measurement. At room temperature, the solubility of **9** itself in toluene-d_6 was extremely limited (heterogenous NMR sample with precipitate) and it was greatly improved when mixed with toluene-d_6 solution of capsule **I**. The chemical shift of the protons α to OH did not change significantly when we applied different ratio of **9**/capsule **I** (1:1 or 1:2 or 3:4), which possibly indicated that the slight deviation of the chemical shift (**9** + capsule **I** vs. pure **9**) was attributed to the limited solubility of **9** in toluene-d_6. The binding constant was calculated based on the 1:1 host-guest stoichiometry. No significant changes in the spectra were observed over time.

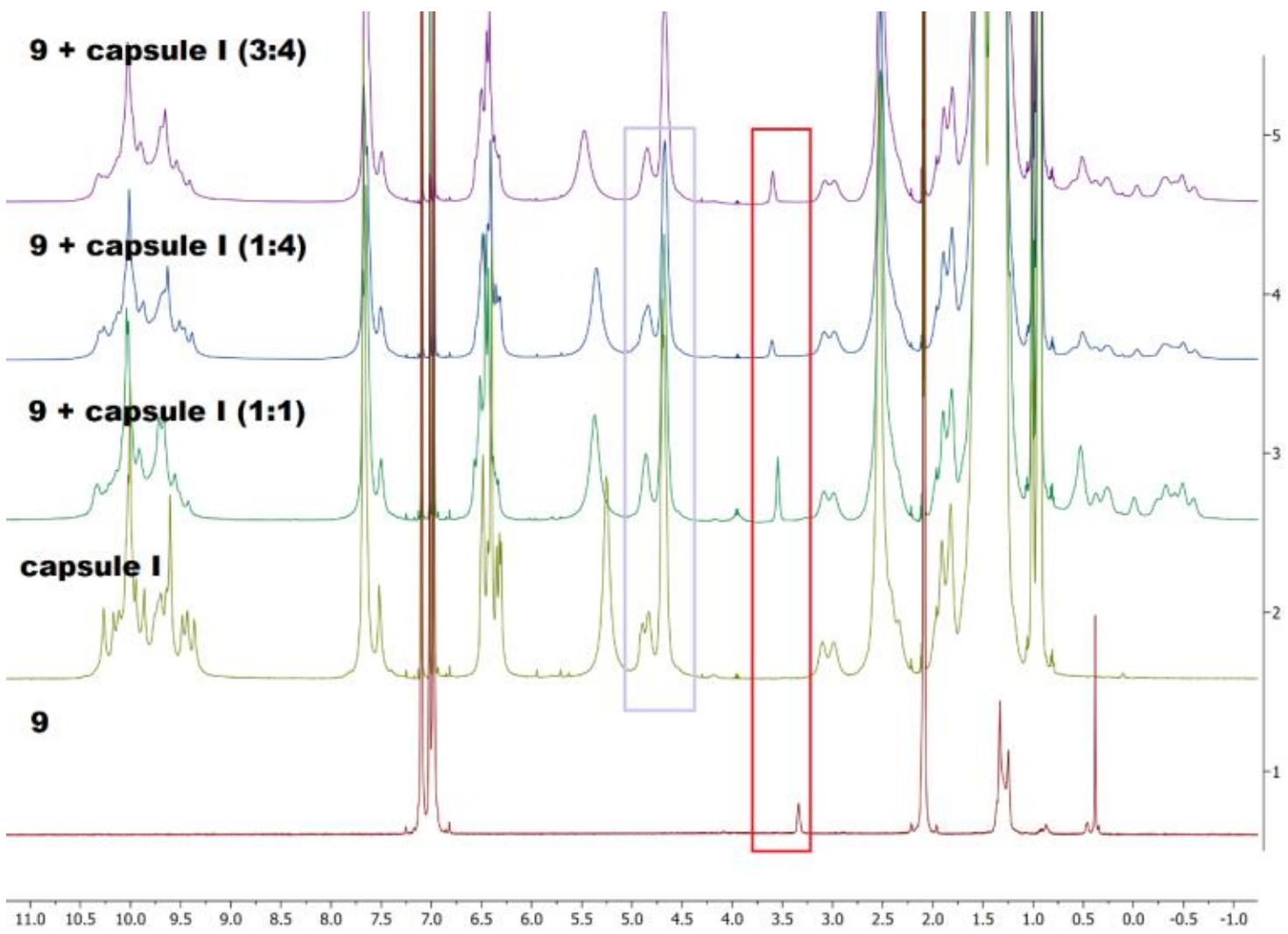

Figure S-35: Stacked ^{1}H NMR spectra (500 MHz, toluene-d_8, 298K) of free **9**, free host (capsule **I**), and host-guest complex (HG) by mixing **9** and capsule **I**

Table S19. Data for calculating the binding constant of **9**

Unbound **9** (mM)	Calculated [HG]	Calculated free host [H]	Binding constant K_1 (M^{-1})
6.40	9.72	5.62	2.70×10^{2}

(2) Binding constant of **10** and capsule **I**

In a 2 mL vial charged with a stir bar, freshly prepared **10** (1.58 mg, 8.19 µmol) was added to a solution of C_{11}-resorcin[4]arene (49.8 mg, 45.0 µmol, corresponding to 7.51 µmol capsule) in 500 µL toluene-d_6 and stirred until a clear solution was formed. Then the solution was transferred into an NMR tube for measurement. The first measurement was started as quickly as possible after preparing the sample. Two more measurements were performed after the first one (30 min and 60 min intervals) and the integral

of unbound **10** remained unchanged, which indicated that hydrolysis of **10** was probably negligible under such NMR measurement conditions.

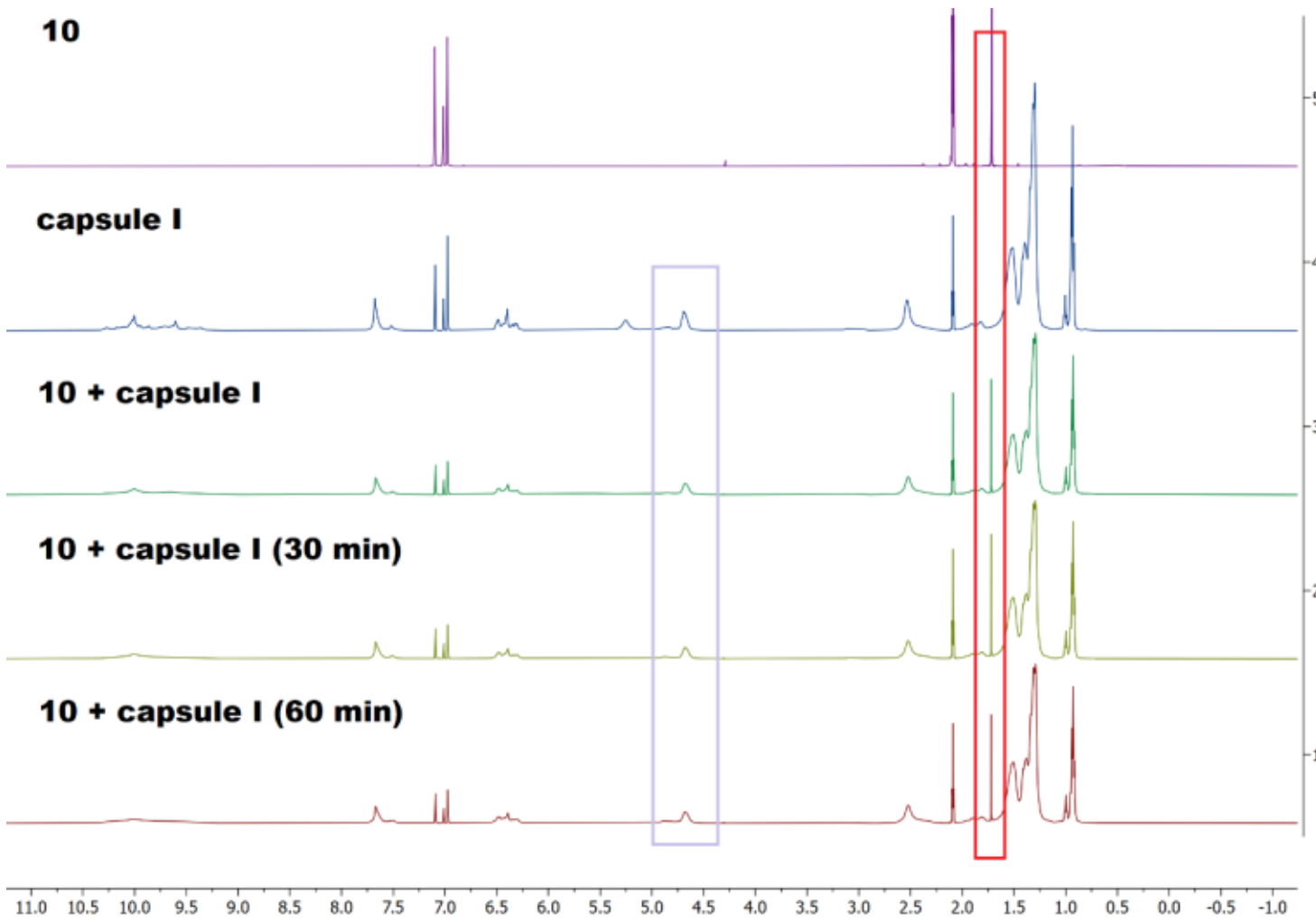

Figure S-36: Stacked ^{1}H NMR spectra (500 MHz, toluene-d_8, 298K) of free **10**, free host (capsule **I**), and host-guest complex (HG) by mixing **10** and capsule **I** in a 1:1 stoichiometry

Table S20. Data for calculating the binding constant of **10**

Unbound **10** (mM)	Calculated [HG]	Calculated free host [H]	Binding constant K_2 (M^{-1})
12.3	4.08	10.9	3.04×10^1

(3) Binding constant of **3aa** and capsule **I**

In a 2 mL vial charged with a stir bar, **3aa** (3.06 mg, 7.53 µmol) and C_{11}-resorcin[4]arene (50.3 mg, 45.5 µmol, corresponding to 7.58 µmol capsule) was dissolved in 500 µL toluene-d_6 and stirred until a clear solution was formed. Then the solution was transferred into an NMR tube for measurement. No significant changes in the spectra were observed over time.

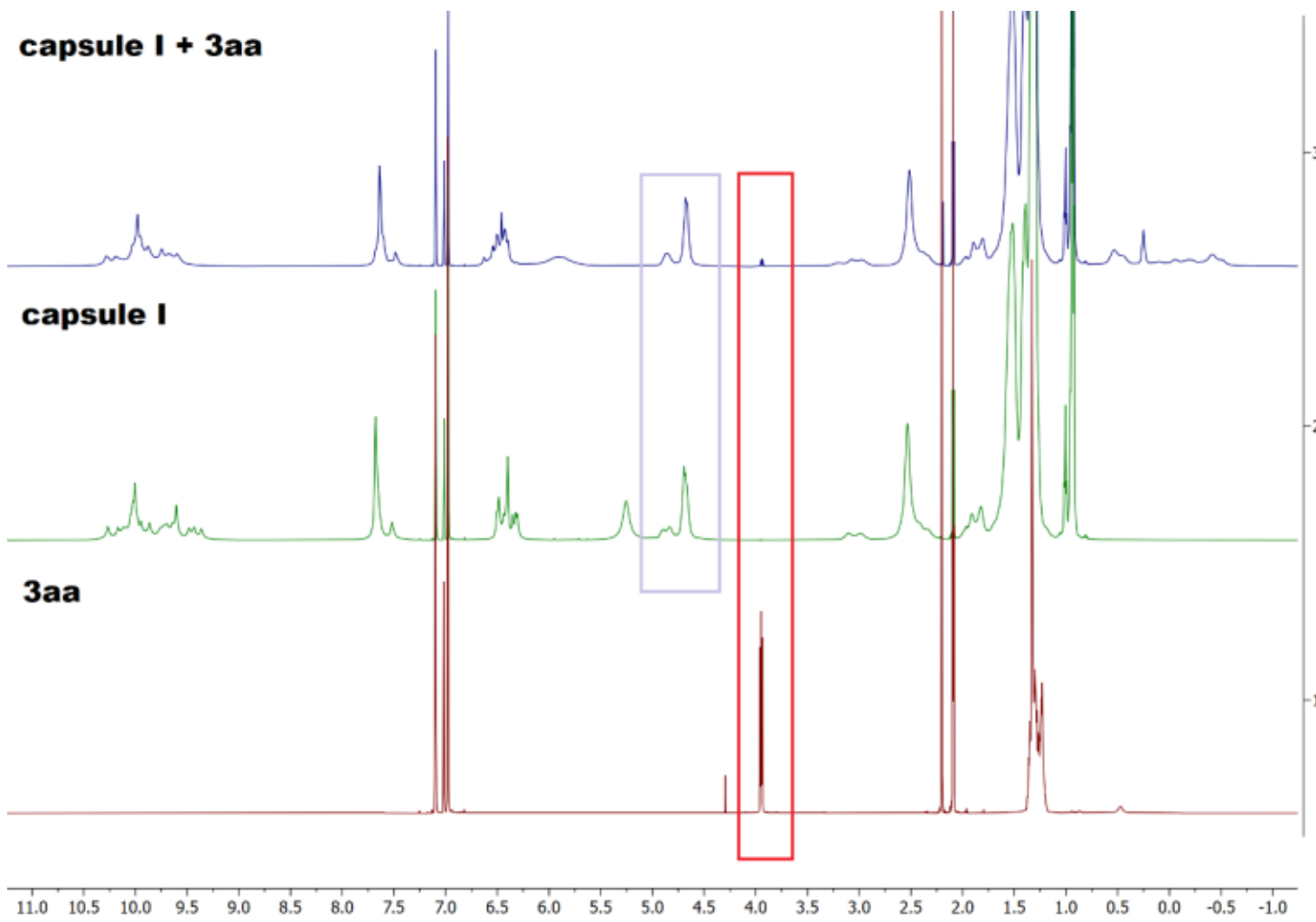

Figure S-37: Stacked ^{1}H NMR spectra (500 MHz, toluene-d_8, 298K) of free **3aa**, free host (capsule **I**), and host-guest complex (HG) by mixing **3aa** and capsule **I** in a 1:1 stoichiometry

Table S21. Data for calculating the binding constant of **3aa**

Unbound **3aa** (mM)	Calculated [HG] (mM)	Calculated free host [H] (mM)	Binding constant K_3 (M^{-1})
1.33	13.7	1.43	7.20×10^3

11. References

1. Gottlieb, H. E., Kotlyar, V. & Nudelman, A. NMR Chemical Shifts of Common Laboratory Solvents as Trace Impurities. *J. Org. Chem.* **62**, 7512–7515 (1997).
2. Catti, L., Pöthig, A. & Tiefenbacher, K. Host-Catalyzed Cyclodehydration–Rearrangement Cascade Reaction of Unsaturated Tertiary Alcohols. *Adv. Synth. Catal.* **359**, 1331–1338 (2017).
3. Li, T.-R., Huck, F., Piccini, G. & Tiefenbacher, K. Mimicry of the proton wire mechanism of enzymes inside a supramolecular capsule enables β-selective O-glycosylations. *Nat. Chem.* **14**, 985–994 (2022).
4. Cornu, I., Syntrivanis, L.-D. & Tiefenbacher, K. Biomimetic tail-to-head terpene cyclizations using the resorcin[4]arene capsule catalyst. *Nat. Protoc.* **19**, 313–339 (2024).
5. de Saint Laumer, J.-Y., Cicchetti, E., Merle, P., Egger, J. & Chaintreau, A. Quantification in Gas Chromatography: Prediction of Flame Ionization Detector Response Factors from Combustion Enthalpies and Molecular Structures. *Anal. Chem.* **82**, 6457–6462 (2010).
6. Yamashita, K. *et al.* Efficient Synthesis of Arylenedioxy-Bridged Porphyrin Dimers through Catalyst-Free Nucleophilic Aromatic Substitution. *ChemPlusChem* **85**, 217–226 (2020).
7. Liu, H., Olsen, C. E. & Christensen, S. B. Tethered Lipids from Thapsia garganica. *J. Nat. Prod.* **67**, 1439–1440 (2004).
8. Samat, A., Bibout, M. E. M. & Elguero, J. Binding and transport of alkali metal ions by synthetic analogues of nactins. *J. Chem. Soc. Perkin 1* 1717–1723 (1985) doi:10.1039/P19850001717.
9. Macho-Ávila, I., González-Ferrer, G., Ballester, P. & Aragay, G. DOSY NMR Experiments at Variable Temperature. A Thorough Analysis of Optimal Conditions for Reliable Results. *Magn. Reson. Chem.* **63**, 712–723 (2025).
10. Zhang, Q. & Tiefenbacher, K. Hexameric Resorcinarene Capsule is a Brønsted Acid: Investigation and Application to Synthesis and Catalysis. *J. Am. Chem. Soc.* **135**, 16213–16219 (2013).
11. Zhang, Q., Catti, L., Kaila, V. R. I. & Tiefenbacher, K. To catalyze or not to catalyze: elucidation of the subtle differences between the hexameric capsules of pyrogallolarene and resorcinarene. *Chem. Sci.* **8**, 1653–1657 (2017).
12. Buckley, B. R. *et al.* The Preparation and Absolute Configurations of Enantiomerically Pure C4-Symmetric Tetraalkoxyresorcin[4]arenes Obtained from Camphorsulfonate Derivatives. *Eur. J. Org. Chem.* **2006**, 5135–5151 (2006).
13. Jerschow, A. & Müller, N. Suppression of Convection Artifacts in Stimulated-Echo Diffusion Experiments. Double-Stimulated-Echo Experiments. *J. Magn. Reson.* **125**, 372–375 (1997).
14. Holz, M. & Weingartner, H. Calibration in accurate spin-echo self-diffusion measurements using 1H and less-common nuclei. *J. Magn. Reson. 1969* **92**, 115–125 (1991).
15. XWINNMR. Software Dept., Bruker Analytik GmbH, Rheinstetten, Germany.
16. Burés, J. Variable Time Normalization Analysis: General Graphical Elucidation of Reaction Orders from Concentration Profiles. *Angew. Chem. Int. Ed.* **55**, 16084–16087 (2016).

Appendix A: Crystallographic details

WFY405_150K

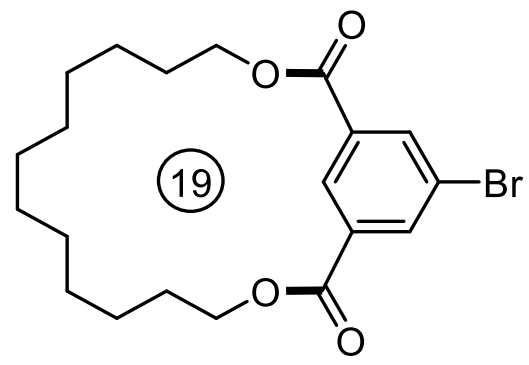

3x (CCDC 2501420)

R_1=2.97%

Submitted by: **Fengyuan Wang**
University of Basel
Solved by: **Alessandro Prescimone**
Sample ID: **WFY405_150K**

Crystal Data and Experimental

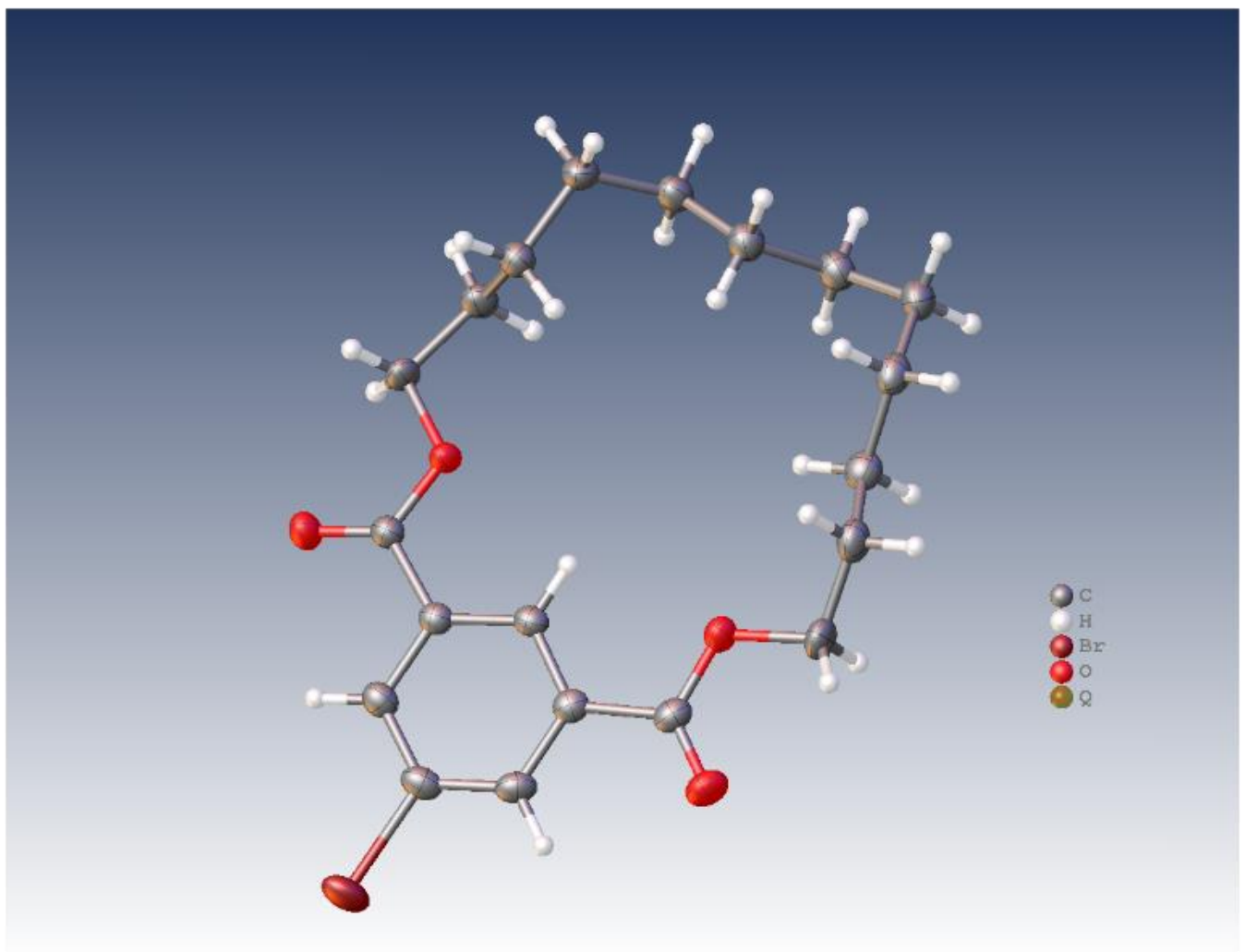

**Compound**	**WFY405_150K**
Formula	$C_{20}H_{27}BrO_4$
$D_{calc.}$/ g cm^{-3}	1.398
μ/mm^{-1}	3.035
Formula Weight	411.32
Colour	colourless
Shape	block-shaped
Size/mm	0.35×0.28×0.25
T/K	150
Crystal System	triclinic
Space Group	*P*-1
a/Å	9.4844(5)
b/Å	10.8172(6)
c/Å	10.8683(6)
α/°	87.022(4)
β/°	67.551(4)
γ/°	71.996(4)
V/Å^3	977.30(10)
Z	2
Z'	1
Wavelength/Å	1.54186
Radiation type	Cu K_α
Θ_{min}/°	4.310
Θ_{max}/°	75.013
Index range h	-8 ≥ h ≥ 11
Index range k	-13 ≥ k ≥ 12
Index range l	-13 ≥ l ≥ 13
Measured Refl's.	14923
Indep't Refl's	3921
Refl's I≥2σ(I)	3512
R_{int}	0.0200
Parameters	226
Restraints	0
Largest Peak/eÅ^3	0.241
Deepest Hole/eÅ^3	-0.633
GooF	1.070
R_1 (I≥2σ(I) / all)	0.0297 / 0.0341
wR_2 (I≥2σ(I) / all)	0.0797 / 0.0831

Experimental. Single colourless block-shaped crystals of **WFY405_150K** were used as supplied. A suitable crystal with dimensions 0.35 × 0.28 × 0.25 mm was selected and mounted on a STOE STADIVARI Cu diffractometer. The crystal was kept at a steady *T* = 150 K during data collection. The structure was solved with the ShelXT 2018/2 (Sheldrick, 2018) solution program using dual methods and by using Olex2 1.5 (Dolomanov et al., 2009) as the graphical interface. The model was refined with ShelXL 2019/3 (Sheldrick, 2015) using full matrix least squares minimisation on $|F|^2$.

Crystal Data. $C_{20}H_{27}BrO_4$, M_r = 411.32, triclinic, *P*-1 (No. 2), a = 9.4844(5) Å, b = 10.8172(6) Å, c = 10.8683(6) Å, α = 87.022(4)°, β = 67.551(4)°, γ = 71.996(4)°, *V* = 977.30(10) Å^3, *T* = 150 K, *Z* = 2, *Z'* = 1, μ(Cu K_α) = 3.035, 14923 reflections measured, 3921 unique (R_{int} = 0.0200) which were used in all calculations. The final wR_2 was 0.0831 (all data) and R_1 was 0.0297 (I≥2σ(I)).

Structure Quality Indicators

**Reflections:**	d min (CuKα) 2Θ=150.0° 0.80	I/σ(I) 60.5	Rint m=3.81 2.00%	Full 135.4° 97% to 150.0° 98.9
**Refinement:**	Shift -0.001	Max Peak 0.2	Min Peak -0.6	GooF 1.070

A colourless block-shaped crystal with dimensions 0.35 × 0.28 × 0.25 mm was mounted. Data were collected using a STOE STADIVARI Cu diffractometer equipped with an Oxford Cryosystems low-temperature device operating at T = 150 K.

Data were measured using rotation method, ω scans with Cu K_{α} radiation. The diffraction pattern was indexed and the total number of runs and images was based on the strategy calculation from the program X-Area Pilatus3_SV 1.31.170.0 (STOE, 2020) . The maximum resolution achieved was Θ= 75.013° (0.80 Å).

The unit cell was refined using X-Area Pilatus3_SV 1.31.170.0 (STOE, 2020) on 24115 reflections, 162% of the observed reflections.

Data reduction, scaling and absorption corrections were performed using X-Area Pilatus3_SV 1.31.170.0 (STOE, 2020). The final completeness is 98.90 % out to 75.013° in Θ. A multi-scan absorption correction was performed using STOE. The absorption coefficient μ of this material is 3.035 mm^{-1} at this wavelength (λ = 1.54186Å) and the minimum and maximum transmissions are 0.066 and 0.133.

The structure was solved in the space group P-1 (# 2) by ShelXT 2018/2 (Sheldrick, 2018) using dual methods. It was refined by full matrix least squares minimisation on $|F|^2$ using version 2019/3 of ShelXL 2019/3 (Sheldrick, 2015). All non-hydrogen atoms were refined anisotropically.

Hydrogen atom positions were calculated geometrically and refined using the riding model.

_exptl_absorpt_process_details: STOE X-Red32, absorption correction by Gaussian integration, analogous toP. Coppens in: F. R. Ahmed (Editor), "Crystallographic Computing", Munksgaard,Copenhagen (1970), 255 - 270. Afterwards scaling of reflection intensities was performed within STOE LANA. J. Koziskova, F. Hahn, J. Richter, J. Kozisek, Acta Chimica Slovaca, vol. 9, no. 2, 2016, pp. 136 - 140. Finally, a spherical absorption correction was done within STOE LANA.

There is a single formula unit in the asymmetric unit, which is represented by the reported sum formula. In other words: Z is 2 and Z' is 1. The moiety formula is C20 H27 Br O4.

Data Plots: Diffraction Data

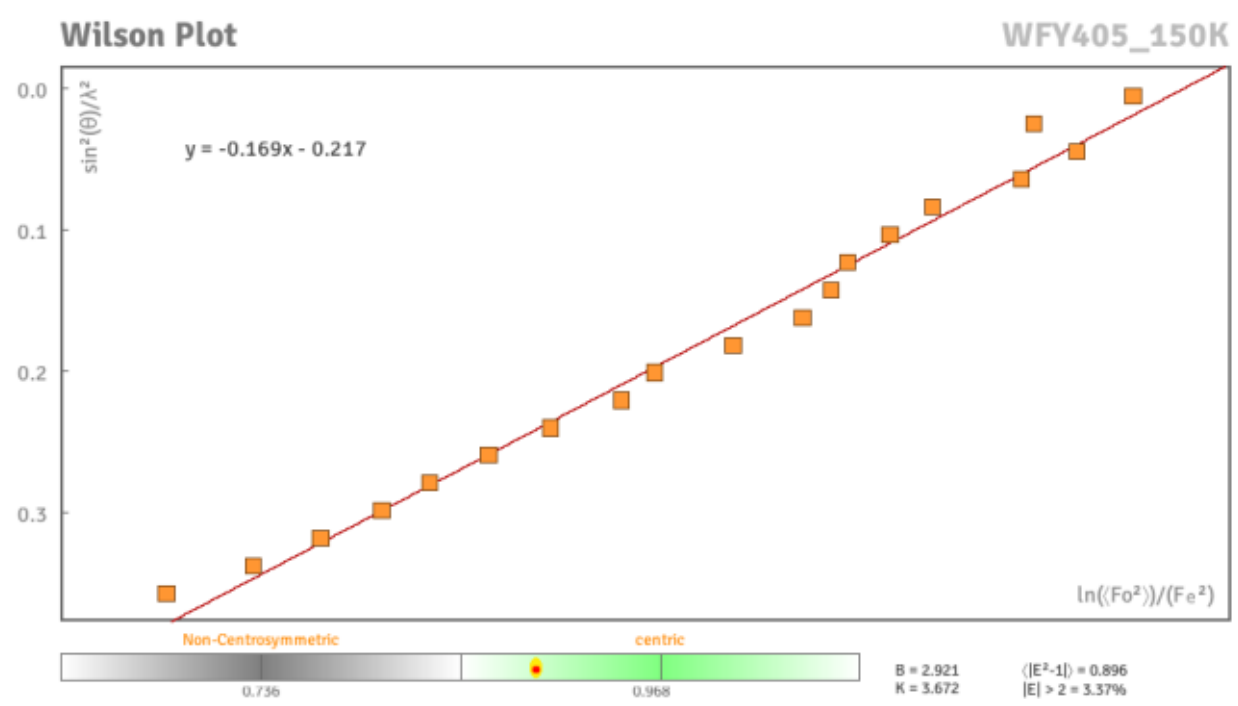

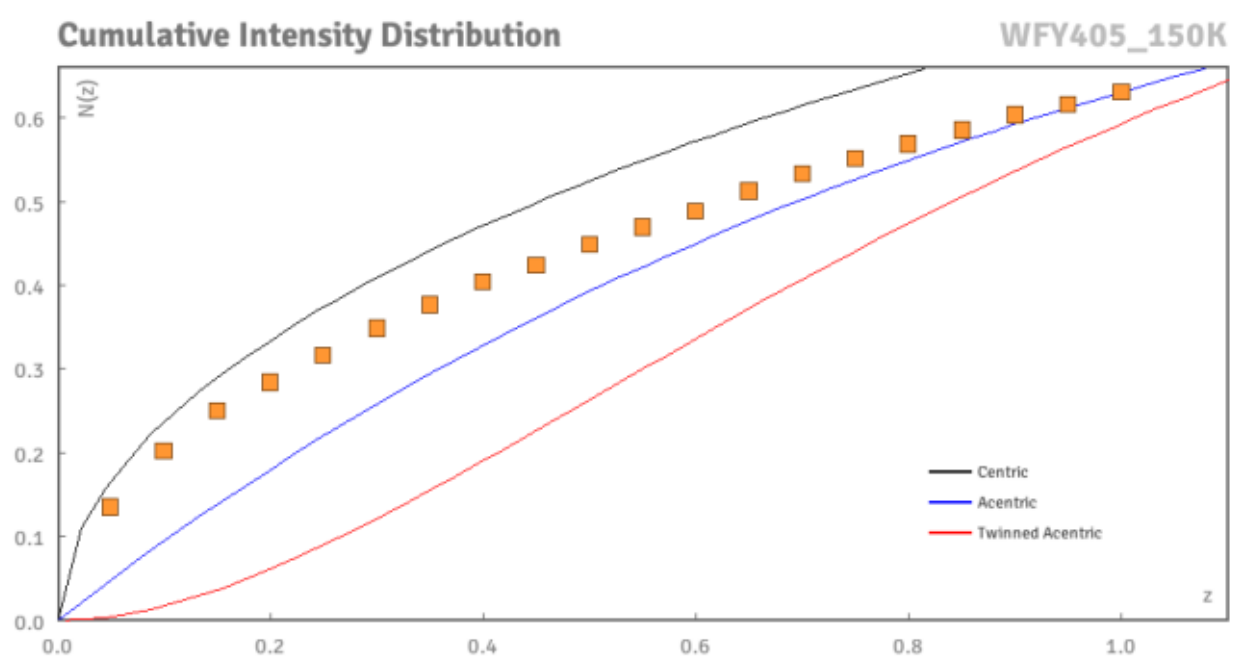

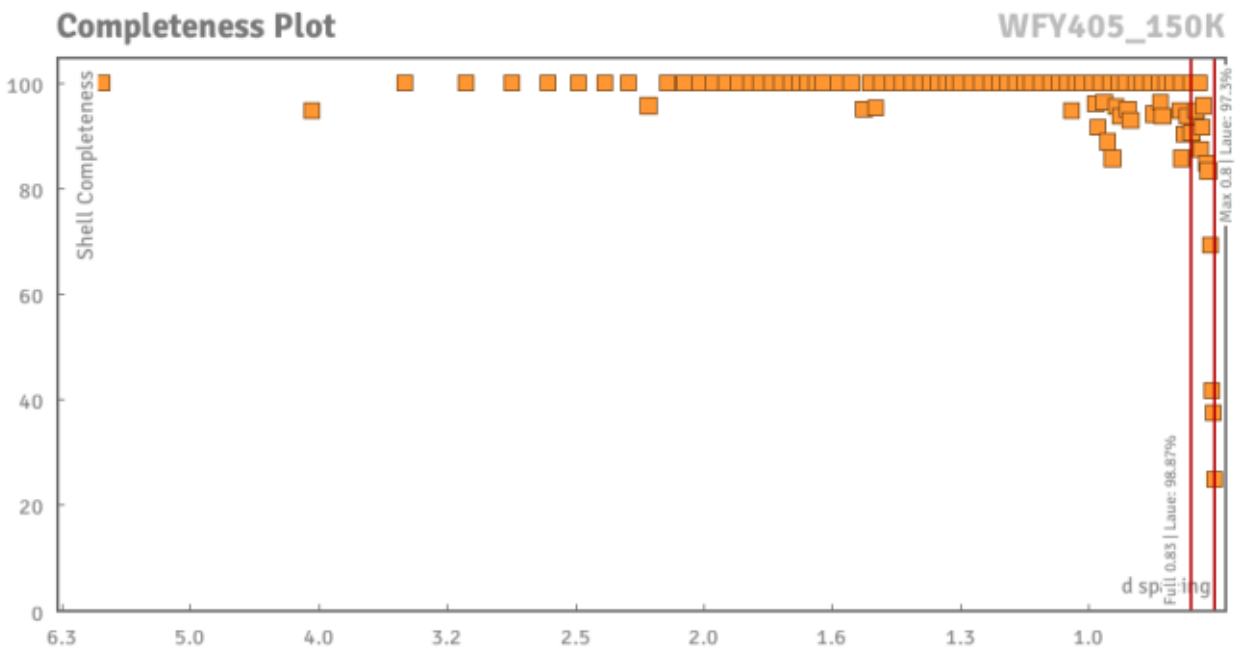

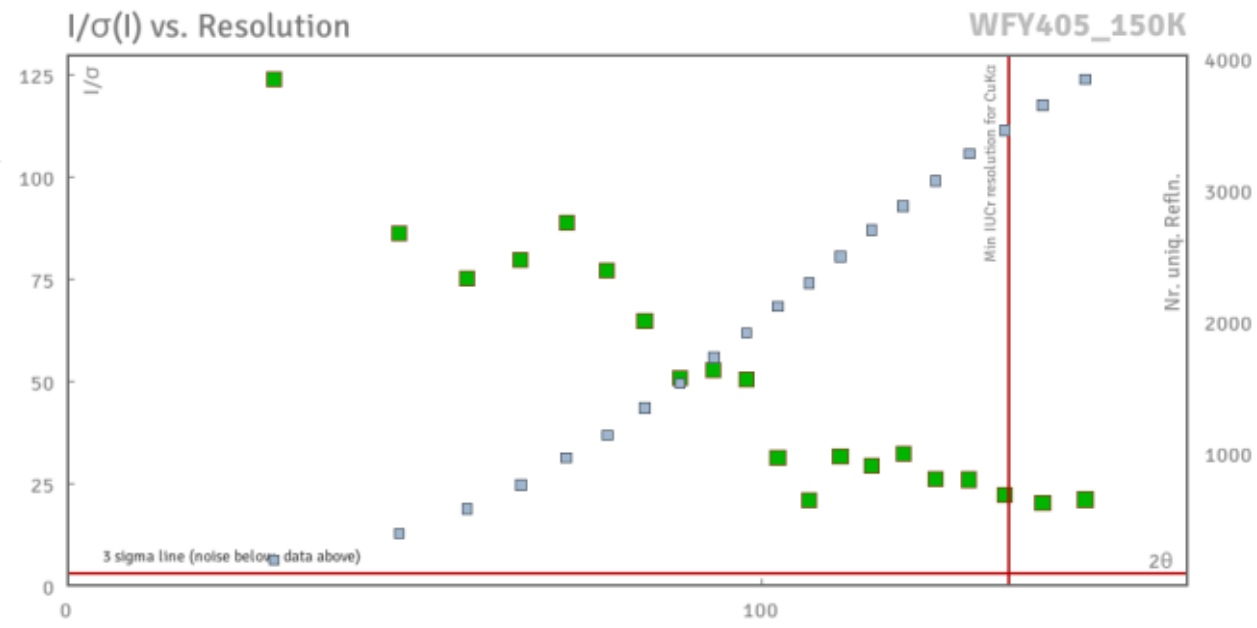

Data Plots: Refinement and Data

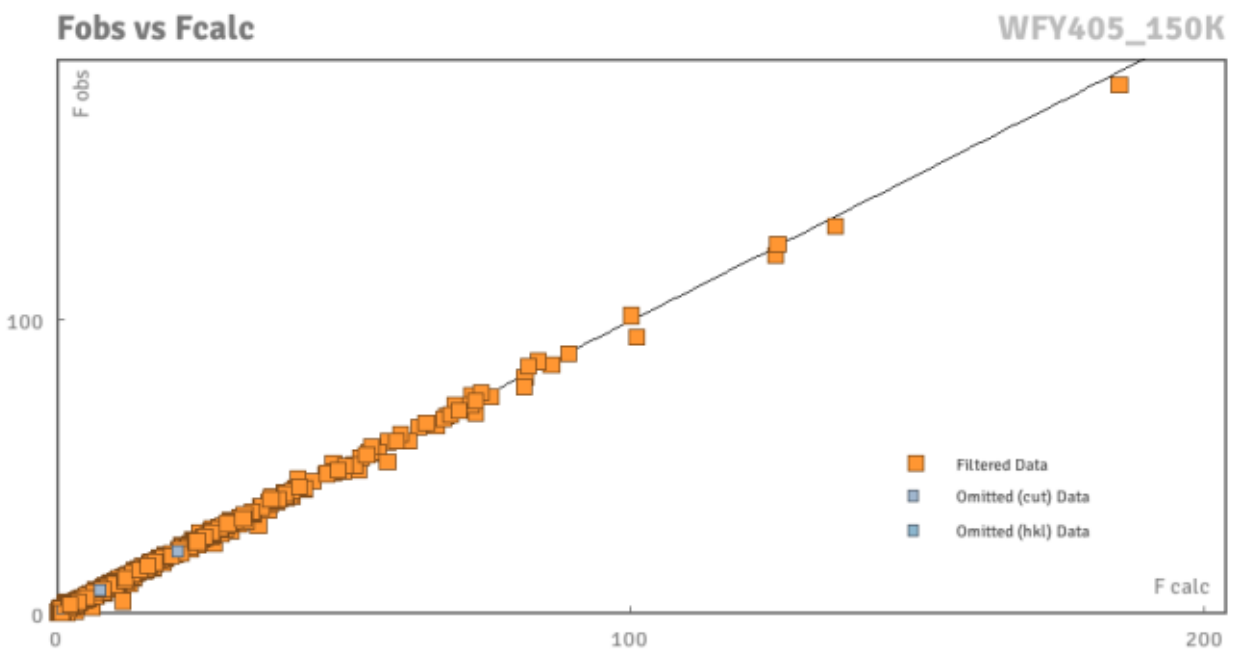

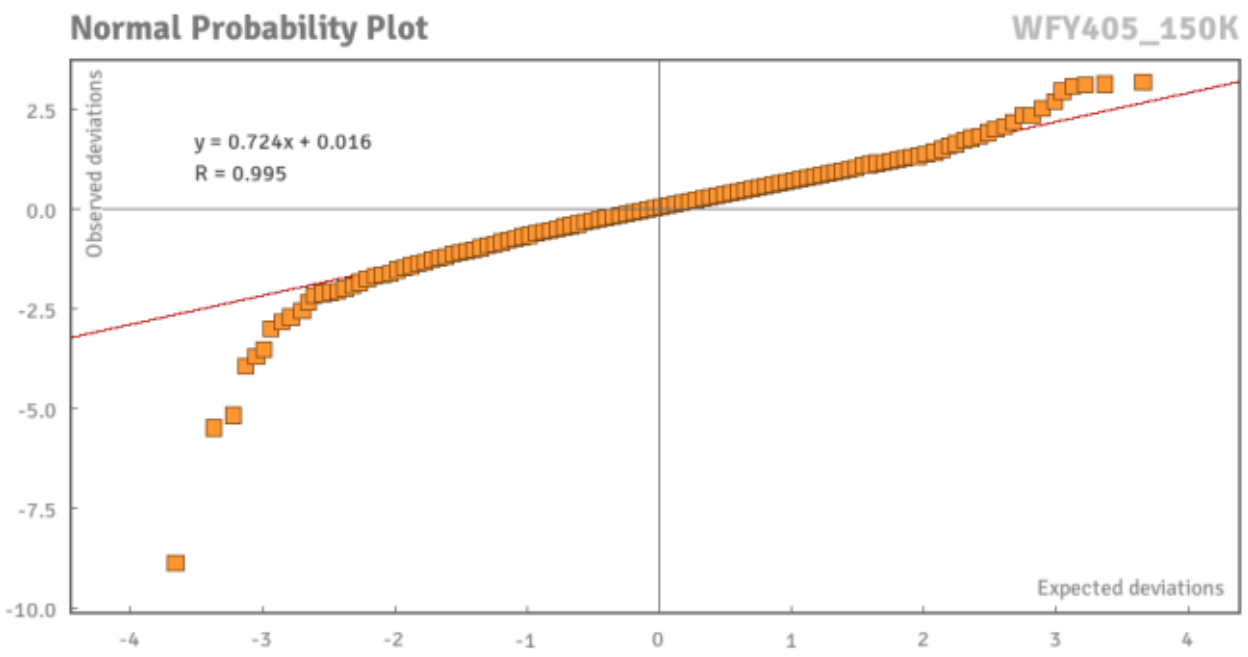

Reflection Statistics

Total reflections (after filtering)	14923	Unique reflections	3921
Completeness	0.973	Mean I/σ	51.22
hkl_{max} collected	(11, 12, 13)	hkl_{min} collected	(-8, -13, -13)
hkl_{max} used	(11, 13, 13)	hkl_{min} used	(-10, -13, 0)
Lim d_{max} collected	100.0	Lim d_{min} collected	0.77
d_{max} used	10.26	d_{min} used	0.8
Friedel pairs	628	Friedel pairs merged	1
Inconsistent equivalents	135	R_{int}	0.02
R_{sigma}	0.0165	Intensity transformed	0
Omitted reflections	0	Omitted by user (OMIT hkl)	3
Multiplicity	(967, 1183, 731, 548, 403, 308, 165, 114, 75, 33, 17, 5, 2)	Maximum multiplicity	13
Removed systematic absences	0	Filtered off (Shel/OMIT)	0

Table 1: Fractional Atomic Coordinates (×10⁴) and Equivalent Isotropic Displacement Parameters (Å²×10³) for **WFY405_150K**. U_{eq} is defined as 1/3 of the trace of the orthogonalised U_{ij}.

Atom	x	y	z	U_{eq}
Br1	9685.9(3)	2801.7(2)	5041.3(2)	44.64(9)
O2	5108.7(16)	7829.2(11)	3496.6(13)	29.0(3)
O4	4626.7(17)	3760.3(12)	2186.4(14)	32.7(3)
O1	6792.9(17)	7777.4(13)	4509.0(15)	36.3(3)
O3	6109.0(19)	1833.1(13)	2570.1(16)	41.9(3)
C4	6217(2)	7225.2(17)	3982.3(17)	25.4(3)
C5	5968(2)	5130.8(16)	3260.3(16)	23.9(3)
C6	6404(2)	3777.1(17)	3215.6(16)	25.6(3)
C3	6663(2)	5773.1(16)	3830.4(16)	24.5(3)
C2	7779(2)	5075.7(18)	4355.1(18)	28.1(4)
C1	8188(2)	3735.7(18)	4300.7(18)	29.8(4)
C7	7518(2)	3071.6(17)	3738.2(17)	28.9(4)
C8	5712(2)	3005.5(17)	2632.6(18)	28.5(4)
C9	4539(2)	9247.3(17)	3741(2)	33.3(4)

Atom	x	y	z	U_{eq}
C10	3157(2)	9778.2(18)	3297(2)	33.4(4)
C18	1819(2)	5090(2)	1286.0(19)	34.5(4)
C13	795(2)	9899.3(18)	1830(2)	34.0(4)
C14	1111(2)	8640.5(19)	1052(2)	33.9(4)
C17	1375(3)	5896(2)	219.2(19)	34.6(4)
C11	3608(2)	9623.9(19)	1799(2)	33.8(4)
C16	-199(3)	7011(2)	701(2)	37.7(4)
C15	-290(2)	8089(2)	1610(2)	35.3(4)
C12	2248(3)	10397(2)	1366(2)	37.4(4)
C20	3907(3)	3065(2)	1603(2)	39.7(5)
C19	3304(3)	3914(2)	652(2)	36.3(4)

Table 2: Anisotropic Displacement Parameters (×10^4) for **WFY405_150K.** The anisotropic displacement factor exponent takes the form: $-2\pi^2[h^2a^{*2} \times U_{11}+ ... +2hka^* \times b^* \times U_{12}]$

Atom	U_{11}	U_{22}	U_{33}	U_{23}	U_{13}	U_{12}
Br1	45.67(15)	34.91(13)	54.96(15)	1.52(9)	-34.08(11)	4.17(9)
O2	36.0(7)	18.1(6)	41.8(7)	3.1(5)	-25.4(6)	-7.1(5)
O4	37.9(7)	22.5(6)	45.9(7)	0.8(5)	-24.2(6)	-10.1(5)
O1	41.2(8)	29.4(7)	50.3(8)	1.1(6)	-30.4(7)	-10.7(6)
O3	53.4(9)	21.8(6)	56.2(9)	3.7(6)	-28.2(8)	-10.8(6)
C4	25.2(8)	24.2(8)	28.0(8)	3.1(6)	-12.2(7)	-7.2(7)
C5	23.0(8)	24.2(8)	23.6(7)	3.8(6)	-9.6(6)	-5.6(6)
C6	26.2(8)	24.7(8)	23.1(8)	1.3(6)	-7.0(7)	-7.2(7)
C3	24.3(8)	24.2(8)	24.4(8)	2.7(6)	-9.2(7)	-7.0(7)
C2	24.7(8)	29.4(9)	28.7(8)	0.7(7)	-11.8(7)	-4.5(7)
C1	26.3(9)	29.7(9)	28.0(8)	1.5(7)	-11.2(7)	-0.4(7)
C7	30.0(9)	21.5(8)	29.1(8)	1.9(6)	-10.0(7)	-1.6(7)
C8	31.1(9)	23.7(8)	28.7(8)	2.2(6)	-9.4(7)	-8.5(7)
C9	40.2(10)	18.6(8)	49.1(11)	0.4(7)	-27.5(9)	-6.3(7)
C10	36.0(10)	22.0(8)	44.3(10)	-0.3(7)	-22.0(9)	-3.3(7)
C18	36.3(10)	35.1(10)	34.3(9)	3.1(8)	-13.8(8)	-13.9(8)
C13	35.9(10)	28.0(9)	42.3(10)	0.2(8)	-22.5(8)	-5.6(8)
C14	34.1(10)	32.1(9)	38.6(10)	1.3(8)	-17.1(8)	-10.4(8)
C17	42.2(11)	37.0(10)	32.0(9)	2.4(8)	-17.1(8)	-19.0(9)
C11	34.2(10)	29.6(9)	42.6(10)	4.0(8)	-20.8(8)	-9.3(8)
C16	41.6(11)	39.0(11)	44.5(11)	6.0(9)	-25.6(9)	-17.8(9)
C15	33.4(10)	36.2(10)	39.8(10)	2.6(8)	-18.0(8)	-10.7(8)
C12	46.7(12)	29.8(9)	47.5(11)	10.4(8)	-29.7(10)	-14.4(9)
C20	48.1(12)	28.5(9)	56.7(12)	0.9(9)	-30.6(10)	-17.7(9)
C19	39.4(11)	34.0(10)	42.1(10)	-3.9(8)	-19.5(9)	-14.5(8)

Table 3: Bond Lengths in Å for **WFY405_150K**.

Atom	Atom	Length/Å
Br1	C1	1.8946(18)
O2	C4	1.332(2)
O2	C9	1.461(2)
O4	C8	1.335(2)
O4	C20	1.460(2)
O1	C4	1.208(2)
O3	C8	1.204(2)
C4	C3	1.495(2)
C5	C6	1.391(2)
C5	C3	1.394(2)
C6	C7	1.392(3)
C6	C8	1.497(2)
C3	C2	1.392(2)
C2	C1	1.378(3)
C1	C7	1.384(3)
C9	C10	1.506(3)
C10	C11	1.521(3)
C18	C17	1.524(3)
C18	C19	1.523(3)
C13	C14	1.527(3)
C13	C12	1.531(3)
C14	C15	1.525(3)

Atom	Atom	Length/Å
C17	C16	1.521(3)
C11	C12	1.530(3)

Atom	Atom	Length/Å
C16	C15	1.528(3)
C20	C19	1.505(3)

Table 4: Bond Angles in ° for **WFY405_150K.**

Atom	Atom	Atom	Angle/°
C4	O2	C9	114.03(13)
C8	O4	C20	115.22(14)
O2	C4	C3	112.74(14)
O1	C4	O2	124.30(16)
O1	C4	C3	122.95(16)
C6	C5	C3	119.25(16)
C5	C6	C7	120.42(16)
C5	C6	C8	122.99(16)
C7	C6	C8	116.58(15)
C5	C3	C4	123.22(15)
C2	C3	C4	115.98(15)
C2	C3	C5	120.74(16)
C1	C2	C3	118.83(16)
C2	C1	Br1	118.33(14)
C2	C1	C7	121.69(16)
C7	C1	Br1	119.97(14)

Atom	Atom	Atom	Angle/°
C1	C7	C6	119.07(16)
O4	C8	C6	112.55(15)
O3	C8	O4	124.31(17)
O3	C8	C6	123.14(17)
O2	C9	C10	108.02(14)
C9	C10	C11	114.89(17)
C19	C18	C17	110.84(17)
C14	C13	C12	113.03(17)
C15	C14	C13	112.40(17)
C16	C17	C18	116.71(17)
C10	C11	C12	113.05(17)
C17	C16	C15	115.36(16)
C14	C15	C16	114.32(17)
C11	C12	C13	114.83(16)
O4	C20	C19	108.90(15)
C20	C19	C18	116.07(18)

Table 5: Torsion Angles in ° for **WFY405_150K.**

Atom	Atom	Atom	Atom	Angle/°
Br1	C1	C7	C6	179.15(13)
O2	C4	C3	C5	-1.0(2)
O2	C4	C3	C2	-178.00(15)
O2	C9	C10	C11	-67.6(2)
O4	C20	C19	C18	-71.5(2)
O1	C4	C3	C5	177.98(17)
O1	C4	C3	C2	1.0(3)
C4	O2	C9	C10	-172.96(16)
C4	C3	C2	C1	177.14(16)
C5	C6	C7	C1	-0.1(3)
C5	C6	C8	O4	-0.9(2)
C5	C6	C8	O3	178.57(18)
C5	C3	C2	C1	0.0(3)
C6	C5	C3	C4	-177.07(16)
C6	C5	C3	C2	-0.2(2)
C3	C5	C6	C7	0.2(2)
C3	C5	C6	C8	179.52(16)
C3	C2	C1	Br1	-179.14(13)
C3	C2	C1	C7	0.1(3)
C2	C1	C7	C6	0.0(3)
C7	C6	C8	O4	178.41(15)
C7	C6	C8	O3	-2.1(3)
C8	O4	C20	C19	-155.98(17)
C8	C6	C7	C1	-179.45(16)
C9	O2	C4	O1	-4.3(3)
C9	O2	C4	C3	174.62(15)
C9	C10	C11	C12	-169.07(15)
C10	C11	C12	C13	-68.2(2)
C18	C17	C16	C15	62.7(2)
C13	C14	C15	C16	167.32(17)

Atom	Atom	Atom	Atom	Angle/°
C14	C13	C12	C11	-74.2(2)
C17	C18	C19	C20	177.65(16)
C17	C16	C15	C14	51.8(2)
C12	C13	C14	C15	174.63(16)
C20	O4	C8	O3	0.4(3)
C20	O4	C8	C6	179.92(16)
C19	C18	C17	C16	172.85(16)

Table 6: Hydrogen Fractional Atomic Coordinates (×10[4]) and Equivalent Isotropic Displacement Parameters (Å^2×10^3) for **WFY405_150K**. U_{eq} is defined as 1/3 of the trace of the orthogonalised U_{ij}.

Atom	x	y	z	U_{eq}
H5	5204.25	5611.93	2906.17	29
H2	8251.38	5515.72	4743.81	34
H7	7815.48	2147.21	3709.36	35
H9A	5414.72	9611.98	3233.67	40
H9B	4181.65	9491.18	4702.37	40
H10A	2648.53	10716.71	3594.11	40
H10B	2346.6	9333.54	3749.57	40
H18A	2029.24	5638.43	1855.29	41
H18B	908.3	4791.06	1860.49	41
H13A	490.36	9747.04	2789.32	41
H13B	-123.24	10579.51	1728.81	41
H14A	1310.9	8812.53	106.11	41
H14B	2089.76	7983.7	1080.79	41
H17A	2251.81	6262.13	-281.15	41
H17B	1330.01	5301.21	-417.36	41
H11A	3912.52	8689.08	1524.6	41
H11B	4557.83	9917	1331.7	41
H16A	-1082.86	6647.22	1186.24	45
H16B	-383.56	7404.8	-89.24	45
H15A	-1302.71	8808.02	1775.78	42
H15B	-332.86	7740.31	2479.22	42
H12A	2680.68	10378.66	378.79	45
H12B	1881.75	11316.73	1715.32	45
H20A	3005.79	2853.54	2316.72	48
H20B	4715.89	2238.3	1120.49	48
H19A	3061.76	3367.62	104.51	44
H19B	4180.88	4228.46	39.98	44

WFY403_150K

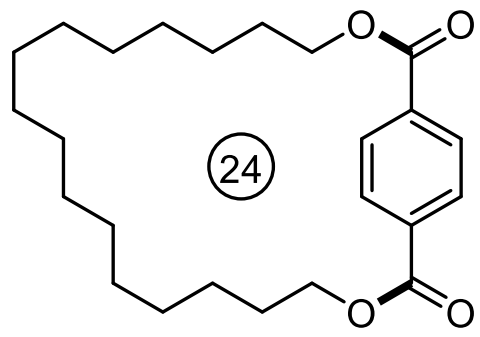

3y (CCDC 2503392)

Report created with ReportPlus

R_1=18.75%

Submitted by: **Fengyuan Wang**
University of Basel
Solved by: **Alessandro Prescimone**

Sample ID: **WFY403**

Crystal Data and Experimental

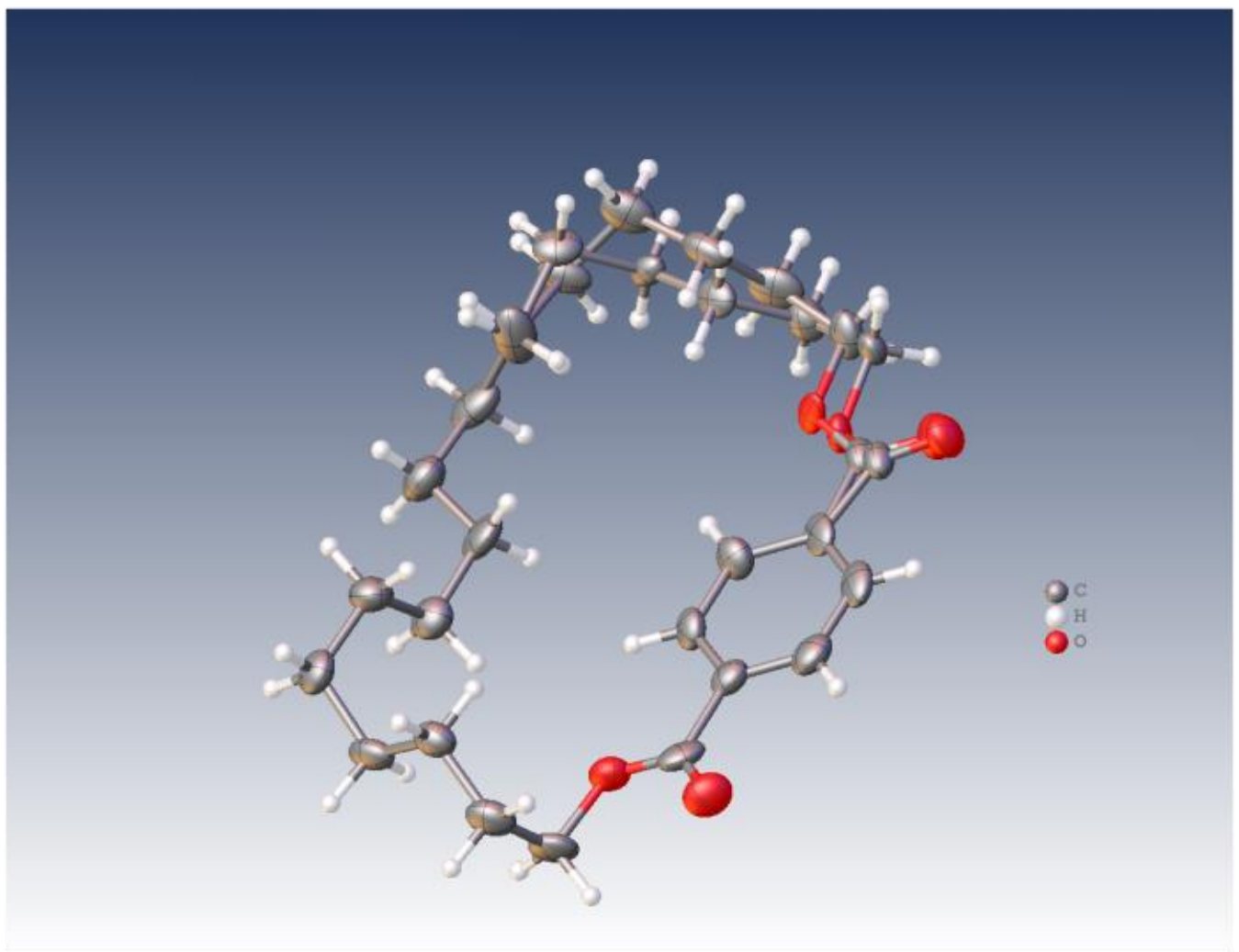

Experimental. Single colourless plate-shaped crystals of **WFY403_150K** were used as supplied. A suitable crystal with dimensions 0.40 × 0.28 × 0.10 mm was selected and mounted on a STOE STADIVARI Cu diffractometer. The crystal was kept at a steady T = 150 K during data collection. The structure was solved with the ShelXT 2018/2 (Sheldrick, 2018) solution program using dual methods and by using Olex2 1.5 (Dolomanov et al., 2009) as the graphical interface. The model was refined with ShelXL 2019/3 (Sheldrick, 2015) using full matrix least squares minimisation on $|F|^2$.

Crystal Data. $C_{24}H_{36}O_4$, M_r = 388.53, monoclinic, $P2_1/n$ (No. 14), a = 5.6831(5) Å, b = 15.1285(10) Å, c = 25.174(2) Å, β = 90.799(7)°, $\alpha = \gamma$ = 90°, V = 2164.2(3) Å³, T = 150 K, Z = 4, Z' = 1, μ(Cu K_α) = 0.628, 20487 reflections measured, 4354 unique (R_{int} = 0.0918) which were used in all calculations. The final wR_2 was 0.4244 (all data) and R_1 was 0.1875 (I≥2σ(I)).

**Compound**	**WFY403_150K**
Formula	$C_{24}H_{36}O_4$
$D_{calc.}$/ g cm^{-3}	1.192
μ/mm^{-1}	0.628
Formula Weight	388.53
Colour	colourless
Shape	plate-shaped
Size/mm	0.40×0.28×0.10
T/K	150
Crystal System	monoclinic
Space Group	$P2_1/n$
a/Å	5.6831(5)
b/Å	15.1285(10)
c/Å	25.174(2)
α/°	90
β/°	90.799(7)
γ/°	90
V/Å³	2164.2(3)
Z	4
Z'	1
Wavelength/Å	1.54186
Radiation type	Cu K_α
Θ_{min}/°	3.408
Θ_{max}/°	75.187
Index range h	-2 ≥ h ≥ 6
Index range k	-18 ≥ k ≥ 18
Index range l	-31 ≥ l ≥ 30
Measured Refl's.	20487
Indep't Refl's	4354
Refl's I≥2σ(I)	3183
R_{int}	0.0918
Parameters	319
Restraints	45
Largest Peak/eÅ³	0.578
Deepest Hole/eÅ³	-0.457
GooF	1.045
R_1 (I≥2σ(I) / all)	0.1875 / 0.2143
wR_2 (I≥2σ(I) / all)	0.4106 / 0.4244

Structure Quality Indicators

**Reflections:**	d min (CuKα) 2Θ=150.4° **0.80**	I/σ(I) **19.8**	Rint m=4.81 **9.18%**	Full 135.4° 97% to 150.4° **99.0**
**Refinement:**	Shift **0.000**	Max Peak **0.6**	Min Peak **-0.5**	GooF **1.045**

A colourless plate-shaped crystal with dimensions 0.40 × 0.28 × 0.10 mm was mounted. Data were collected using a STOE STADIVARI Cu diffractometer equipped with an Oxford Cryosystems low-temperature device operating at T = 150 K.

Data were measured using rotation method, ω scans with Cu K_{α} radiation. The diffraction pattern was indexed and the total number of runs and images was based on the strategy calculation from the program X-Area Pilatus3_SV 1.31.170.0 (STOE, 2020) . The maximum resolution achieved was Θ= 75.187° (0.80 Å).

The unit cell was refined using X-Area Pilatus3_SV 1.31.170.0 (STOE, 2020) on 24212 reflections, 118% of the observed reflections.

Data reduction, scaling and absorption corrections were performed using X-Area Pilatus3_SV 1.31.170.0 (STOE, 2020). The final completeness is 99.00 % out to 75.187° in Θ. A multi-scan absorption correction was performed using STOE. The absorption coefficient μ of this material is 0.628 mm^{-1} at this wavelength (λ = 1.54186Å) and the minimum and maximum transmissions are 0.299 and 0.687.

The structure was solved in the space group $P2_1/n$ (# 14) by ShelXT 2018/2 (Sheldrick, 2018) using dual methods. It was refined by full matrix least squares minimisation on $|F|^2$ using version 2019/3 of ShelXL 2019/3 (Sheldrick, 2015). All non-hydrogen atoms were refined anisotropically.

Hydrogen atom positions were calculated geometrically and refined using the riding model.

_refine_special_details: some parts of the molecule are orientationally disordered over 2 positions. some SADI and ISOR had to be used to treat it.

_exptl_absorpt_process_details: STOE X-Red32, absorption correction by Gaussian integration, analogous toP. Coppens in: F. R. Ahmed (Editor), "Crystallographic Computing", Munksgaard,Copenhagen (1970), 255 - 270. Afterwards scaling of reflection intensities was performed within STOE LANA. J. Koziskova, F. Hahn, J. Richter, J. Kozisek, Acta Chimica Slovaca, vol. 9, no. 2, 2016, pp. 136 - 140. Finally, a spherical absorption correction was done within STOE LANA.

There is a single formula unit in the asymmetric unit, which is represented by the reported sum formula. In other words: Z is 4 and Z' is 1. The moiety formula is C24 H36 O4.

Data Plots: Diffraction Data

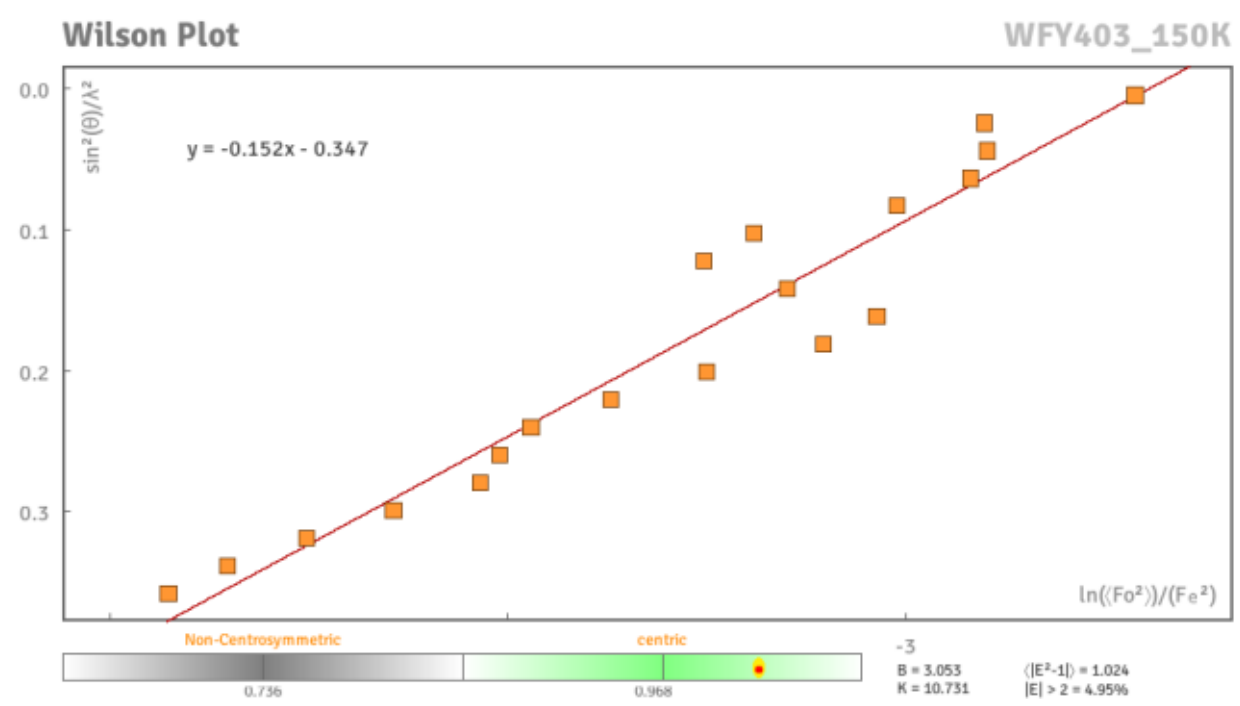

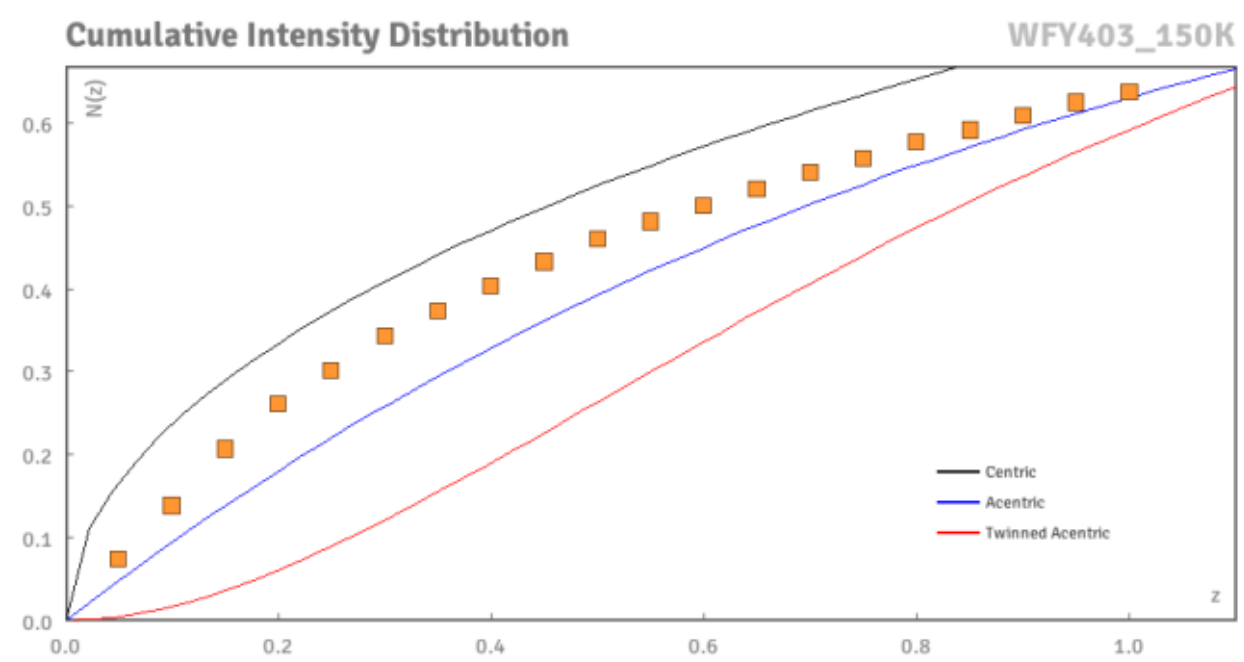

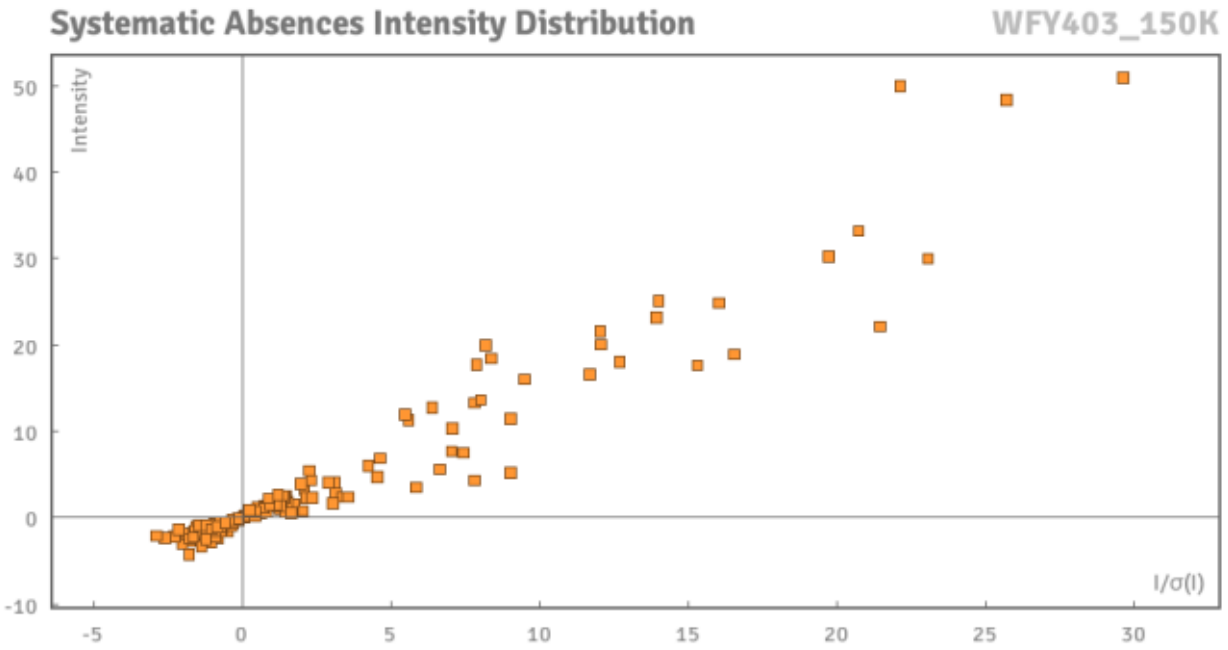

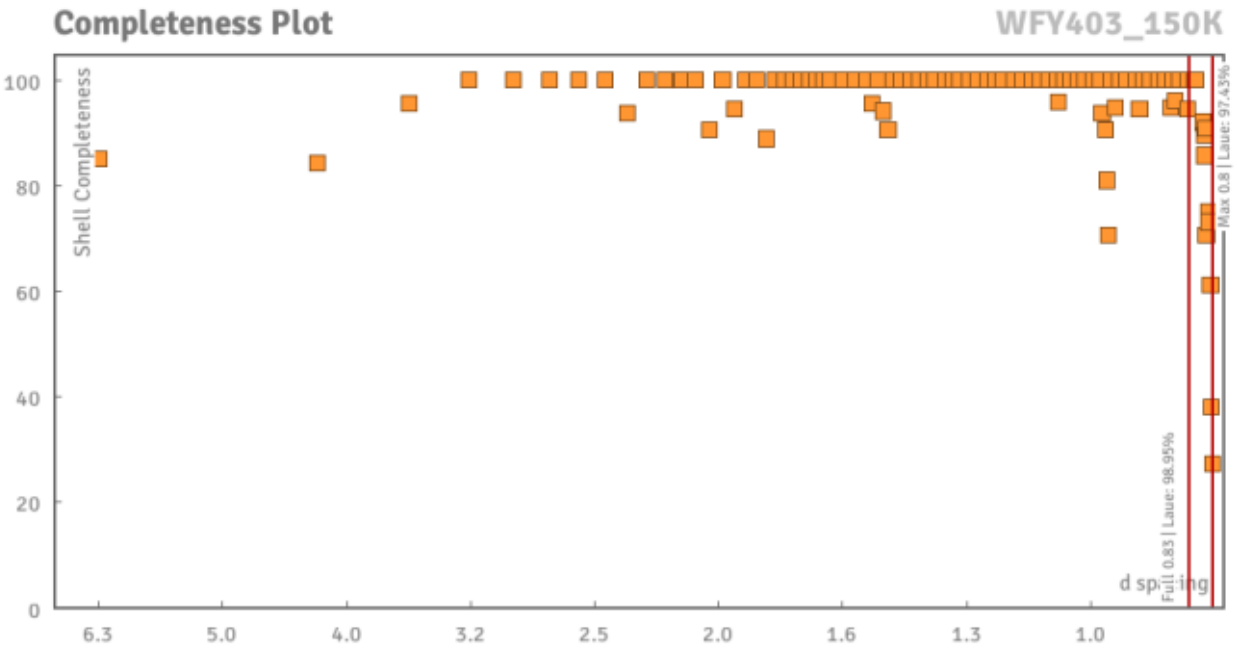

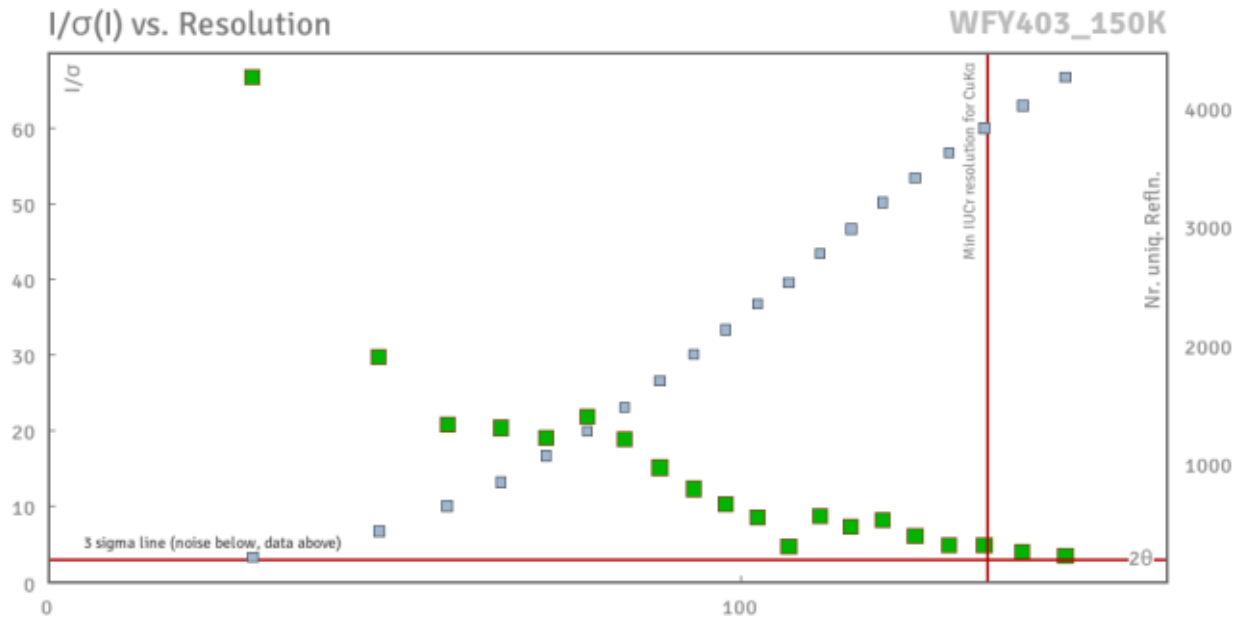

Data Plots: Refinement and Data

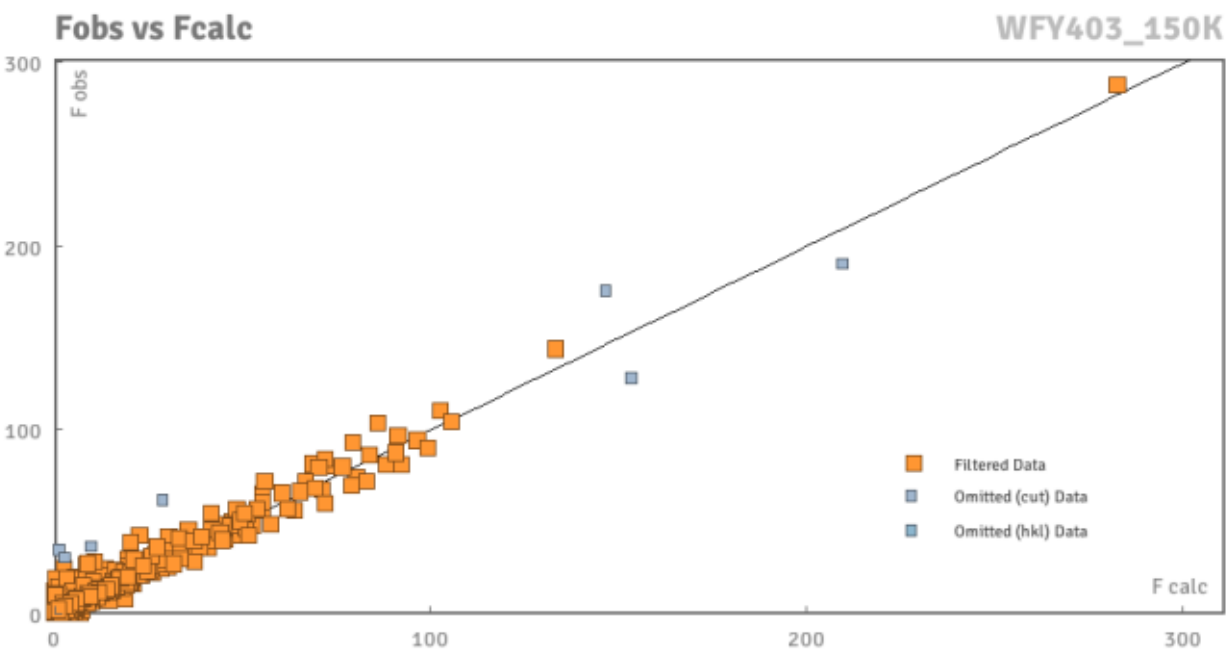

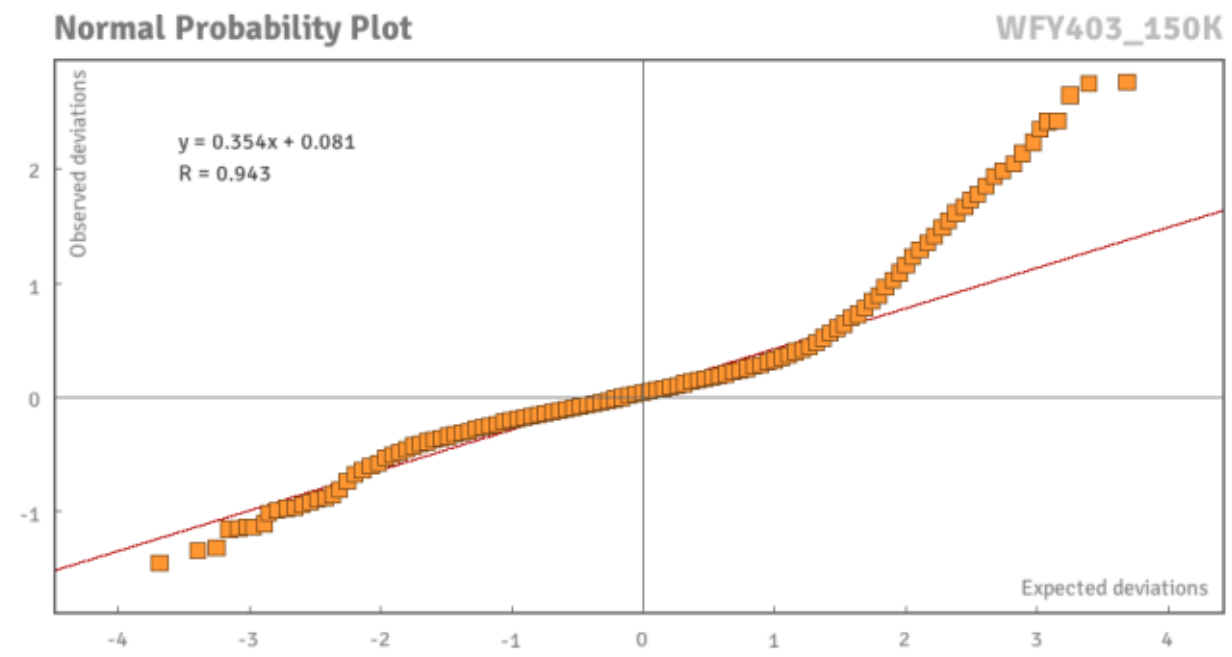

Reflection Statistics

Total reflections (after filtering)	20926	Unique reflections	4354
Completeness	0.974	Mean I/σ	14.84
hkl_{max} collected	(6, 18, 30)	hkl_{min} collected	(-2, -18, -31)
hkl_{max} used	(6, 18, 31)	hkl_{min} used	(-6, 0, 0)
Lim d_{max} collected	100.0	Lim d_{min} collected	0.77
d_{max} used	12.97	d_{min} used	0.8
Friedel pairs	261	Friedel pairs merged	1
Inconsistent equivalents	148	R_{int}	0.0918
R_{sigma}	0.0505	Intensity transformed	0
Omitted reflections	0	Omitted by user (OMIT hkl)	11
Multiplicity	(2725, 1596, 1063, 898, 709, 473, 199, 52, 3, 2)	Maximum multiplicity	17
Removed systematic absences	439	Filtered off (Shel/OMIT)	0

Table 7: Fractional Atomic Coordinates (×10^4) and Equivalent Isotropic Displacement Parameters (Å^2×10^3) for **WFY403_150K.** U_{eq} is defined as 1/3 of the trace of the orthogonalised U_{ij}.

Atom	x	y	z	U_{eq}
O2	4799(10)	7403(4)	3835(2)	43.1(14)
O1	1157(11)	7895(4)	3948(2)	53.4(16)
O5	3710(30)	7550(11)	1179(6)	31(3)

Atom	x	y	z	U_{eq}
C1	2732(15)	7668(5)	3674(3)	41.6(19)
C3	4372(14)	7318(5)	2772(3)	37.9(17)
O4	1020(30)	8075(14)	1089(7)	55(5)
C23	5308(14)	5674(5)	4355(3)	40.6(18)
C2	2591(14)	7680(5)	3069(3)	36.2(17)
C22	7953(16)	5550(5)	4396(3)	44(2)
C9	649(16)	8035(5)	2820(4)	46(2)
C5	2271(16)	7701(5)	1966(3)	42.0(19)
C6	2470(40)	7699(18)	1353(5)	34(3)
C24	4418(17)	6485(6)	4626(3)	49(2)
C8	486(16)	8054(5)	2273(4)	47(2)
C25	5283(17)	7347(6)	4408(3)	47(2)
C4	4233(15)	7332(5)	2224(3)	41.0(18)
C21	8833(17)	4730(5)	4099(3)	47(2)
C20	8474(16)	4764(6)	3498(3)	46(2)
C18	9249(19)	5601(5)	2621(4)	54(2)
C19	9502(17)	5607(5)	3236(3)	48(2)
C17	10931(18)	4993(6)	2369(4)	54(2)
C16	11008(19)	5092(6)	1756(4)	57(3)
C10	3650(30)	7394(13)	596(6)	31(4)
C15	8781(19)	4755(8)	1475(4)	71(3)
C11	5820(40)	6951(16)	475(9)	42(5)
C13	8340(30)	5577(9)	611(5)	32(3)
C28	5330(30)	5579(12)	602(6)	48(4)
C14	8590(40)	4654(11)	853(6)	43(5)
C12	6120(30)	6037(11)	720(7)	39(4)
C27	7200(30)	4911(13)	525(6)	53(5)
C26	9380(30)	4980(16)	880(5)	45(5)
C29	5970(40)	6503(17)	424(9)	55(7)
C30	4340(50)	7180(16)	556(7)	46(6)
O6	4150(40)	7253(11)	1142(6)	44(5)
O3	20(30)	8009(14)	1171(7)	47(4)
C7	1760(30)	7777(18)	1379(5)	34(3)

Table 8: Anisotropic Displacement Parameters (×10^4) for **WFY403_150K.** The anisotropic displacement factor exponent takes the form: $-2\pi^2[h^2a^{*2} \times U_{11} + \ldots + 2hka^* \times b^* \times U_{12}]$

Atom	U_{11}	U_{22}	U_{33}	U_{23}	U_{13}	U_{12}
O2	58(4)	40(3)	31(3)	-8(2)	2(2)	7(3)
O1	56(4)	55(4)	50(4)	-7(3)	13(3)	5(3)
O5	30(7)	33(10)	30(7)	7(6)	-2(5)	13(6)
C1	47(5)	26(4)	52(5)	-19(3)	4(4)	-9(3)
C3	47(4)	26(4)	40(4)	13(3)	-4(3)	2(3)
O4	64(11)	51(8)	47(8)	4(6)	-9(9)	16(10)
C23	51(5)	42(4)	29(4)	1(3)	-2(3)	-7(4)
C2	46(4)	21(3)	42(4)	-2(3)	3(3)	-1(3)
C22	73(6)	39(4)	20(3)	0(3)	-1(3)	-12(4)
C9	54(5)	23(4)	61(5)	-3(3)	0(4)	0(3)
C5	62(5)	19(3)	44(4)	16(3)	-12(4)	-6(3)
C6	34(7)	27(4)	42(4)	5(3)	-13(5)	-2(6)
C24	62(5)	59(5)	26(4)	-5(4)	3(3)	-9(4)
C8	55(5)	21(4)	64(6)	14(3)	-16(4)	-6(3)
C25	67(6)	53(5)	21(4)	-13(3)	4(3)	-5(4)
C4	51(5)	41(4)	32(4)	11(3)	2(3)	0(4)
C21	62(5)	27(4)	53(5)	8(3)	-2(4)	4(4)
C20	59(5)	40(4)	39(4)	-8(4)	-4(4)	-2(4)
C18	80(7)	21(4)	60(6)	4(4)	10(5)	10(4)
C19	60(5)	34(4)	49(5)	4(4)	1(4)	-4(4)
C17	71(6)	38(5)	54(5)	-1(4)	6(4)	12(4)

Atom	U_{11}	U_{22}	U_{33}	U_{23}	U_{13}	U_{12}
C16	86(7)	27(4)	59(6)	1(4)	25(5)	0(4)
C10	37(8)	36(8)	21(6)	2(5)	-5(5)	10(6)
C15	79(7)	100(9)	34(5)	11(5)	18(5)	42(7)
C11	42(11)	51(13)	32(11)	7(10)	6(8)	4(11)
C13	52(9)	23(7)	20(6)	2(5)	8(6)	1(6)
C28	65(12)	54(11)	26(8)	4(8)	-6(7)	-21(10)
C14	42(12)	42(11)	46(11)	-8(8)	5(8)	-18(8)
C12	55(10)	32(8)	29(8)	12(7)	4(7)	-11(8)
C27	50(10)	81(14)	29(8)	-3(8)	11(7)	-11(10)
C26	44(12)	66(15)	24(8)	-8(8)	11(7)	-17(9)
C29	64(14)	76(18)	26(10)	0(15)	16(8)	-14(15)
C30	52(10)	54(10)	33(8)	18(7)	6(7)	13(8)
O6	82(12)	37(10)	13(5)	12(6)	8(6)	28(7)
O3	39(8)	58(8)	43(8)	-2(6)	-10(7)	12(8)
C7	34(7)	27(4)	42(4)	5(3)	-13(5)	-2(6)

Table 9: Bond Lengths in Å for **WFY403_150K**.

Atom	Atom	Length/Å
O2	C1	1.301(10)
O2	C25	1.467(9)
O1	C1	1.188(10)
O5	C10	1.486(15)
O5	C7	1.267(17)
C1	C2	1.525(11)
C3	C2	1.380(11)
C3	C4	1.380(10)
O4	C6	1.195(16)
C23	C22	1.517(12)
C23	C24	1.496(12)
C2	C9	1.371(11)
C22	C21	1.535(11)
C9	C8	1.380(12)
C5	C6	1.548(15)
C5	C8	1.391(13)
C5	C4	1.399(11)
C5	C7	1.505(15)
C6	O6	1.287(17)
C24	C25	1.501(12)
C21	C20	1.523(11)
C20	C19	1.554(11)
C18	C19	1.553(12)
C18	C17	1.477(12)
C17	C16	1.552(13)
C16	C15	1.529(16)
C10	C11	1.44(2)
C15	C14	1.574(16)
C15	C26	1.577(15)
C11	C12	1.52(2)
C13	C14	1.529(17)
C13	C12	1.474(18)
C28	C27	1.48(2)
C28	C29	1.51(2)
C27	C26	1.518(18)
C29	C30	1.42(2)
C30	O6	1.487(16)
O3	C7	1.169(16)

Table 10: Bond Angles in ° for **WFY403_150K**.

Atom	Atom	Atom	Angle/°
C1	O2	C25	118.7(6)
C7	O5	C10	115.5(15)
O2	C1	C2	110.5(7)
O1	C1	O2	126.3(8)
O1	C1	C2	123.2(8)
C2	C3	C4	120.2(7)
C24	C23	C22	114.3(7)
C3	C2	C1	120.5(7)
C9	C2	C1	119.5(7)
C9	C2	C3	120.0(8)
C23	C22	C21	113.3(7)
C2	C9	C8	120.4(8)
C8	C5	C6	128.3(10)
C8	C5	C4	118.5(8)
C8	C5	C7	112.7(9)
C4	C5	C6	113.1(10)
C4	C5	C7	128.8(10)
O4	C6	C5	119.5(18)
O4	C6	O6	121.9(16)
O6	C6	C5	118.5(14)
C23	C24	C25	115.5(7)
C9	C8	C5	120.6(8)
O2	C25	C24	110.6(6)
C3	C4	C5	120.3(8)
C20	C21	C22	114.6(7)
C21	C20	C19	113.8(7)
C17	C18	C19	112.3(8)
C18	C19	C20	112.9(7)

Atom	Atom	Atom	Angle/˚
C18	C17	C16	113.2(8)
C15	C16	C17	113.2(8)
C11	C10	O5	106.1(15)
C16	C15	C14	122.6(11)
C16	C15	C26	100.4(10)
C10	C11	C12	115.3(16)
C12	C13	C14	115.4(14)
C27	C28	C29	114.5(17)
C13	C14	C15	108.2(11)

Atom	Atom	Atom	Angle/˚
C13	C12	C11	116.3(14)
C28	C27	C26	117.2(16)
C27	C26	C15	111.1(12)
C30	C29	C28	115.8(17)
C29	C30	O6	109.9(17)
C6	O6	C30	120.8(16)
O5	C7	C5	102.4(12)
O3	C7	O5	129.8(16)
O3	C7	C5	127.8(16)

Table 11: Torsion Angles in ˚ for **WFY403_150K**.

Atom	Atom	Atom	Atom	Angle/˚
O2	C1	C2	C3	9.0(10)
O2	C1	C2	C9	-172.5(7)
O1	C1	C2	C3	-172.8(8)
O1	C1	C2	C9	5.7(11)
O5	C10	C11	C12	64(2)
C1	O2	C25	C24	83.9(9)
C1	C2	C9	C8	179.9(7)
C3	C2	C9	C8	-1.5(11)
O4	C6	O6	C30	-1(4)
C23	C22	C21	C20	64.8(10)
C23	C24	C25	O2	54.2(10)
C2	C3	C4	C5	-0.9(12)
C2	C9	C8	C5	0.9(12)
C22	C23	C24	C25	63.0(10)
C22	C21	C20	C19	53.3(11)
C5	C6	O6	C30	176.1(19)
C6	C5	C8	C9	-176.7(15)
C6	C5	C4	C3	177.2(13)
C24	C23	C22	C21	-178.1(7)
C8	C5	C6	O4	4(3)
C8	C5	C6	O6	-172.6(17)
C8	C5	C4	C3	0.2(12)
C8	C5	C7	O5	170.4(15)
C8	C5	C7	O3	-8(3)
C25	O2	C1	O1	1.9(12)
C25	O2	C1	C2	180.0(6)
C4	C3	C2	C1	-180.0(7)
C4	C3	C2	C9	1.5(11)
C4	C5	C6	O4	-173(2)
C4	C5	C6	O6	11(3)
C4	C5	C8	C9	-0.2(11)
C4	C5	C7	O5	-10(3)
C4	C5	C7	O3	172(2)
C21	C20	C19	C18	176.6(8)
C18	C17	C16	C15	-70.3(11)
C19	C18	C17	C16	-170.2(8)
C17	C18	C19	C20	-74.5(11)
C17	C16	C15	C14	-170.8(11)
C17	C16	C15	C26	177.9(11)
C16	C15	C14	C13	-74.2(18)
C16	C15	C26	C27	-166.4(15)
C10	O5	C7	C5	169.0(15)
C10	O5	C7	O3	-13(4)
C10	C11	C12	C13	-178.8(16)
C28	C27	C26	C15	68(2)
C28	C29	C30	O6	62(3)

Atom	Atom	Atom	Atom	Angle/˚
C14	C13	C12	C11	-179.0(15)
C12	C13	C14	C15	-68.3(19)
C27	C28	C29	C30	-172.1(18)
C29	C28	C27	C26	70(2)
C29	C30	O6	C6	-167(2)
C7	O5	C10	C11	-167(2)
C7	C5	C8	C9	179.5(13)
C7	C5	C4	C3	-179.5(15)

Table 12: Hydrogen Fractional Atomic Coordinates (×10[4]) and Equivalent Isotropic Displacement Parameters (Å[2]×10[3]) for **WFY403_150K.** U_{eq} is defined as 1/3 of the trace of the orthogonalised U_{ij}.

Atom	x	y	z	U_{eq}
H3	5698.21	7059.46	2944.92	45
H23A	4846.12	5701.91	3974.36	49
H23B	4532.29	5150.23	4510	49
H22A	8414.56	5499.71	4775.28	53
H22B	8735.48	6080.83	4250.61	53
H9	-592.46	8270.72	3025.85	55
H24A	4873.52	6451.47	5006.78	59
H24B	2677.74	6481.86	4604.81	59
H8	-856.91	8309.26	2104.6	56
H25A	4493.33	7842.14	4591.29	56
H25B	6997.58	7400.39	4475.45	56
H4	5474.68	7088.96	2021.57	49
H21A	10532.86	4654.98	4177.37	57
H21B	8008.79	4204.05	4237.34	57
H20A	6767.55	4733.37	3416.32	55
H20B	9222.95	4237.39	3339.23	55
H18A	7625.63	5423.58	2520.91	65
H18B	9508.97	6207.09	2484.82	65
H19A	8683.94	6132.69	3377.39	57
H19B	11188.83	5657.68	3334.25	57
H17A	10494.01	4377.12	2457.49	65
H17B	12523.32	5103.81	2519.15	65
H16A	11231.51	5723.06	1666.81	69
H16B	12380.85	4761.48	1621.81	69
H10A	2288.24	7021.02	493.99	38
H10B	3538.35	7961.41	401.09	38
H15A	7483.54	5150.06	1581.83	85
H15B	8436.52	4166.21	1627.26	85
H15C	7361.77	5073.35	1593.92	85
H15D	8562.31	4111.58	1527.99	85
H11A	7147.04	7327.2	597.3	50
H11B	5932.19	6894.46	84.16	50
H13A	9660.89	5946.51	745.54	38
H13B	8512.07	5526.23	221.25	38
H28A	4933.63	5597.85	983.87	58
H28B	3901.99	5387.21	404	58
H14A	10012.04	4358.96	716.75	52
H14B	7200.32	4289.63	756.51	52
H12A	5967.13	6093.55	1110.12	46
H12B	4804.44	5659.42	592.04	46
H27A	7713.33	4945.15	151.54	64
H27B	6504.06	4318.74	577.58	64
H26A	10023.69	5586.36	860.28	54
H26B	10595.64	4565.58	752.26	54
H29A	7518.07	6658.06	582.98	66
H29B	6149.8	6497.29	33.53	66

Atom	x	y	z	U_{eq}
H30A	2773.78	7040.33	398.37	56
H30B	4866.91	7751.33	407.29	56

Table 13: Atomic Occupancies for all atoms that are not fully occupied in **WFY403_150K**.

Atom	Occupancy
O5	0.5
O4	0.5
C6	0.5
C10	0.5
H10A	0.5
H10B	0.5
H15A	0.5
H15B	0.5
H15C	0.5
H15D	0.5
C11	0.5
H11A	0.5
H11B	0.5
C13	0.5
H13A	0.5
H13B	0.5
C28	0.5
H28A	0.5
H28B	0.5
C14	0.5
H14A	0.5
H14B	0.5
C12	0.5
H12A	0.5
H12B	0.5
C27	0.5
H27A	0.5
H27B	0.5
C26	0.5
H26A	0.5
H26B	0.5
C29	0.5
H29A	0.5
H29B	0.5
C30	0.5
H30A	0.5
H30B	0.5
O6	0.5
O3	0.5
C7	0.5

WFY452_150K

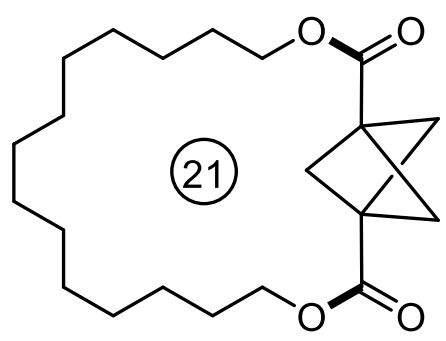

3ab (CCDC 2516792)

R_1=4.71%

Submitted by: **Fengyuan Wang**
University of Basel
Solved by: **Alessandro Prescimone**

Sample ID: **WFY452**

Crystal Data and Experimental

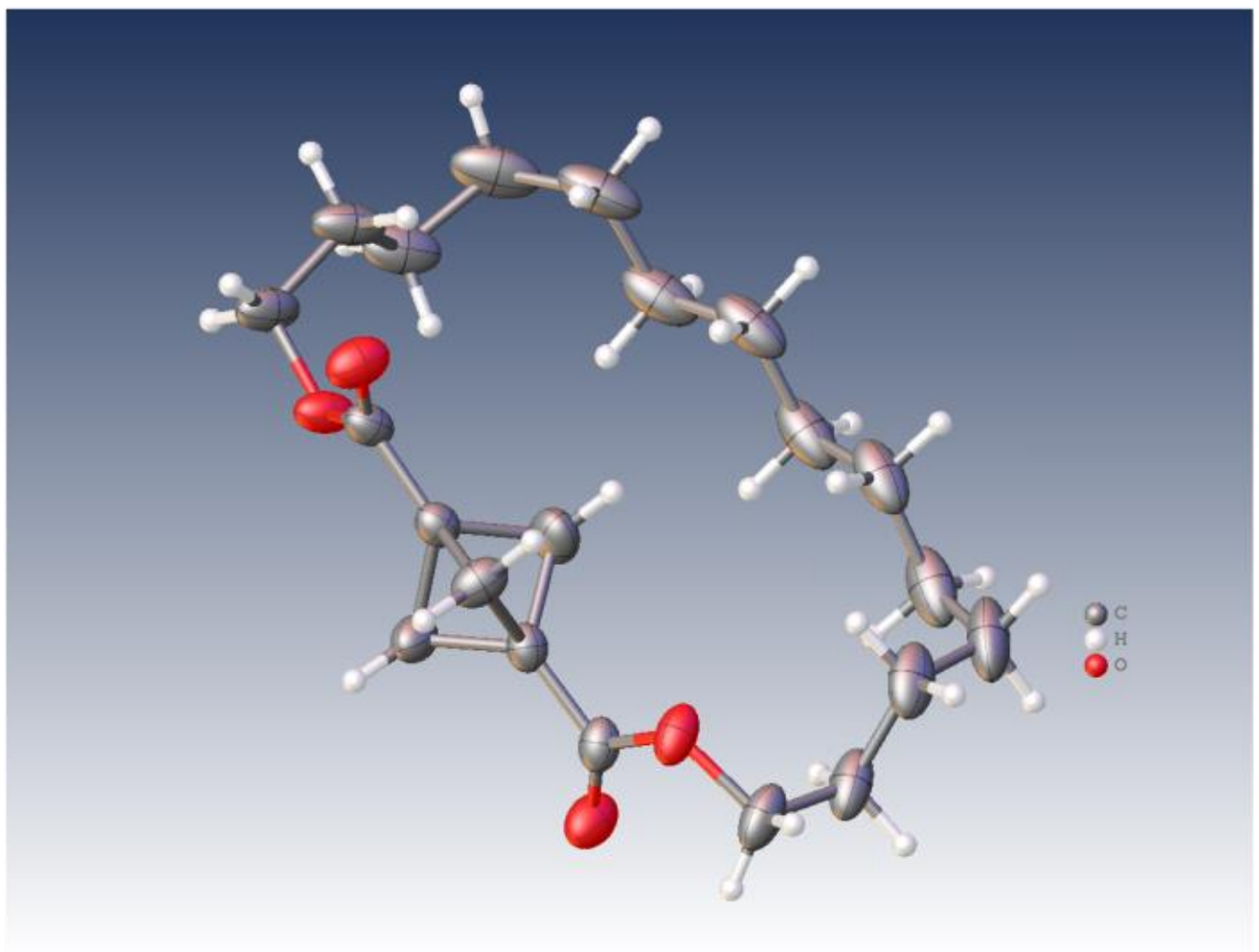

**Compound**	**WFY452_150K**
Formula	$C_{21}H_{34}O_4$
$D_{calc.}$/ g cm^{-3}	1.159
μ/mm^{-1}	0.413
Formula Weight	350.48
Colour	colourless
Shape	plate-shaped
Size/mm	0.40×0.32×0.15
T/K	150
Crystal System	monoclinic
Space Group	$I2/a$
a/Å	10.6381(4)
b/Å	16.4711(5)
c/Å	12.2055(5)
α/°	90
β/°	110.136(3)
γ/°	90
V/Å^3	2007.94(13)
Z	4
Z'	0.5
Wavelength/Å	1.34143
Radiation type	GaK_α
Θ_{min}/°	4.089
Θ_{max}/°	59.566
Index range h	-12 ≥ h ≥ 13
Index range k	-16 ≥ k ≥ 21
Index range l	-14 ≥ l ≥ 15
Measured Refl's.	15476
Indep't Refl's	2224
Refl's I≥2σ(I)	2034
R_{int}	0.0171
Parameters	114
Restraints	0
Largest Peak/eÅ^3	0.170
Deepest Hole/eÅ^3	-0.231
GooF	1.039
R_1 (I≥2σ(I) / all)	0.0471 / 0.0505
wR_2 (I≥2σ(I) / all)	0.1244 / 0.1275

Experimental. Single colourless plate-shaped crystals of **WFY452_150K** were used as supplied. A suitable crystal with dimensions 0.40 × 0.32 × 0.15 mm was selected and mounted on a STOE STADIVARI diffractometer. The crystal was kept at a steady T = 150 K during data collection. The structure was solved with the ShelXT 2018/2 (Sheldrick, 2018) solution program using dual methods and by using Olex2 1.5 (Dolomanov et al., 2009) as the graphical interface. The model was refined with ShelXL 2019/3 (Sheldrick, 2015) using full matrix least squares minimisation on $|F|^2$.

Crystal Data. $C_{21}H_{34}O_4$, M_r = 350.48, monoclinic, $I2/a$ (No. 15), a = 10.6381(4) Å, b = 16.4711(5) Å, c = 12.2055(5) Å, β = 110.136(3)°, $\alpha = \gamma$ = 90°, V = 2007.94(13) Å^3, T = 150 K, Z = 4, Z' = 0.5, $\mu(GaK_\alpha)$ = 0.413, 15476 reflections measured, 2224 unique (R_{int} = 0.0171) which were used in all calculations. The final wR_2 was 0.1275 (all data) and R_1 was 0.0471 (I≥2σ(I)).

Structure Quality Indicators

**Reflections:**	d min (GaKα) 2Θ=119.1° 0.78	I/σ(I) 129.4	Rint m=7.11 1.71%	Full 107.2° 99.9
**Refinement:**	Shift 0.000	Max Peak 0.2	Min Peak -0.2	GooF 1.039

A colourless plate-shaped crystal with dimensions 0.40 × 0.32 × 0.15 mm was mounted. Data were collected using a STOE STADIVARI diffractometer equipped with an Oxford Cryosystems low-temperature device operating at T = 150 K.

Data were measured using rotation method, ω scans with GaK_{α} radiation. The diffraction pattern was indexed and the total number of runs and images was based on the strategy calculation from the program X-Area Pilatus3_SV 1.31.186.0 (STOE, 2022) . The maximum resolution achieved was Θ= 59.566° (0.78 Å).

The unit cell was refined using X-Area Pilatus3_SV 1.31.186.0 (STOE, 2022) on 47099 reflections, 304% of the observed reflections.

Data reduction, scaling and absorption corrections were performed using X-Area Pilatus3_SV 1.31.186.0 (STOE, 2022). The final completeness is 99.90 % out to 59.566° in Θ. A multi-scan absorption correction was performed using STOE. The absorption coefficient μ of this material is 0.413 mm^{-1} at this wavelength (λ = 1.34143Å) and the minimum and maximum transmissions are 0.819 and 0.906.

The structure was solved in the space group $I2/a$ (# 15) by ShelXT 2018/2 (Sheldrick, 2018) using dual methods. It was refined by full matrix least squares minimisation on $|F|^2$ using version 2019/3 of ShelXL 2019/3 (Sheldrick, 2015). All non-hydrogen atoms were refined anisotropically.

Hydrogen atom positions were calculated geometrically and refined using the riding model.

_exptl_absorpt_process_details: STOE X-Red32, absorption correction by Gaussian integration, analogous toP. Coppens in: F. R. Ahmed (Editor), "Crystallographic Computing", Munksgaard,Copenhagen (1970), 255 - 270. Afterwards scaling of reflection intensities was performed within STOE LANA. J. Koziskova, F. Hahn, J. Richter, J. Kozisek, Acta Chimica Slovaca, vol. 9, no. 2, 2016, pp. 136 - 140. Finally, a spherical absorption correction was done within STOE LANA.

The value of Z' is 0.5. This means that only half of the formula unit is present in the asymmetric unit, with the other half consisting of symmetry equivalent atoms. The moiety formula is C21 H34 O4.

Data Plots: Diffraction Data

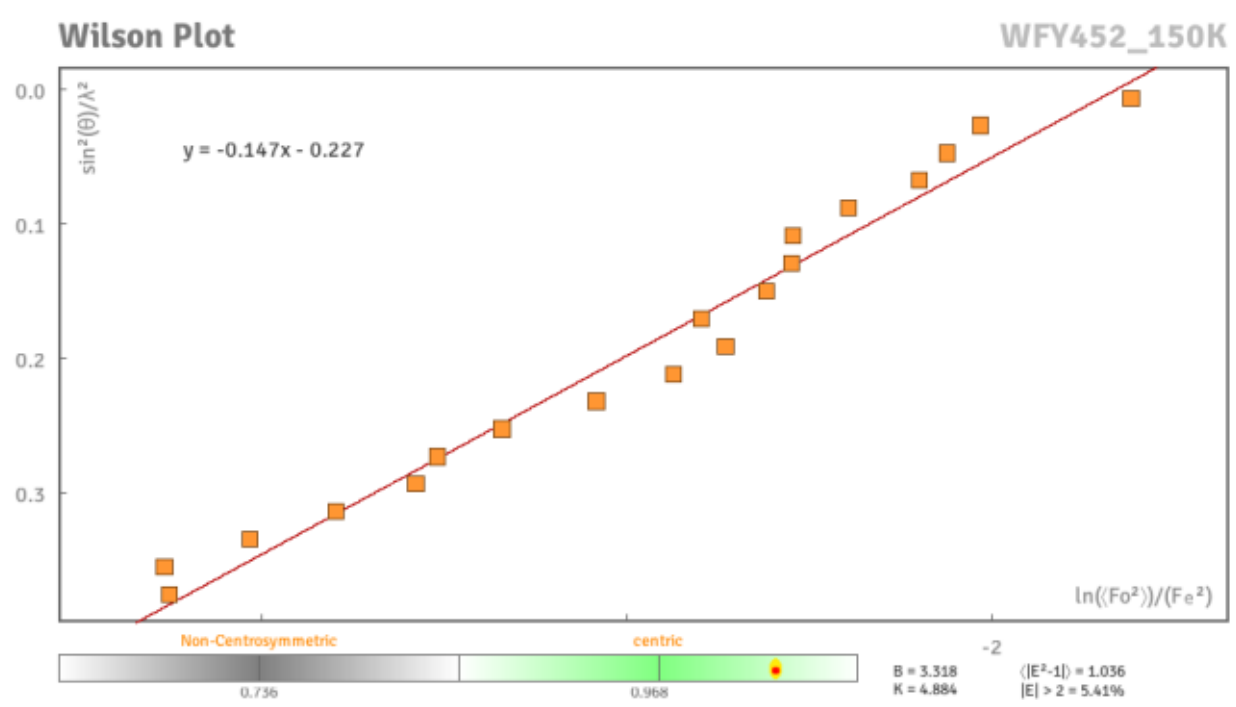

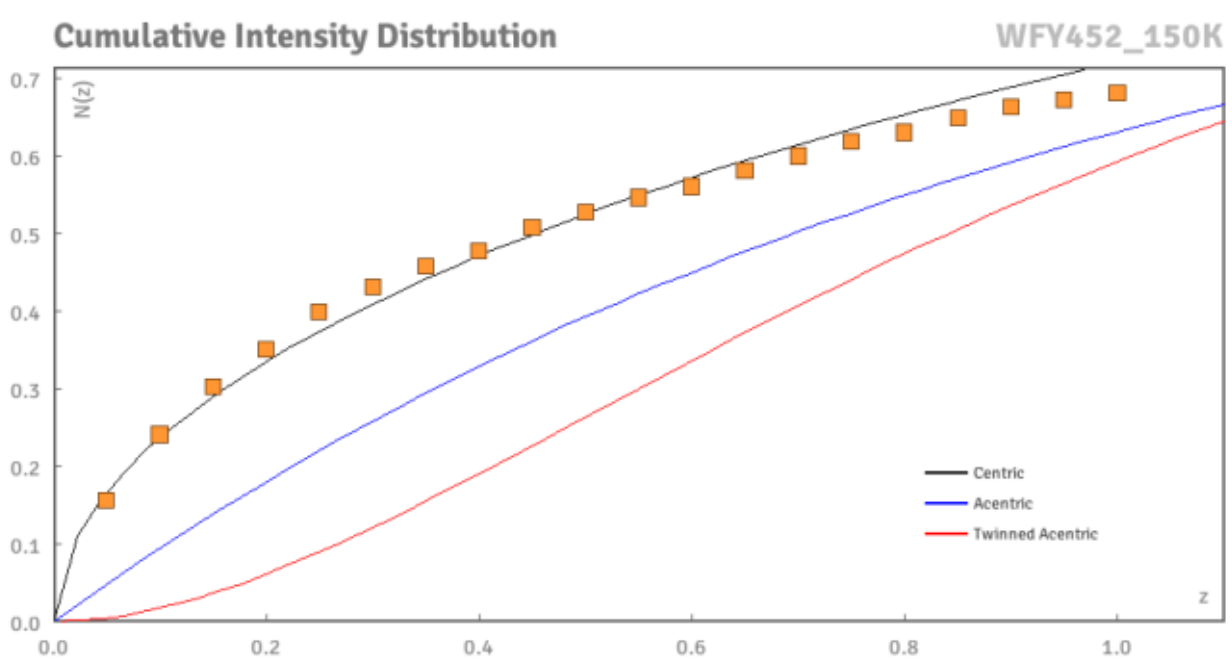

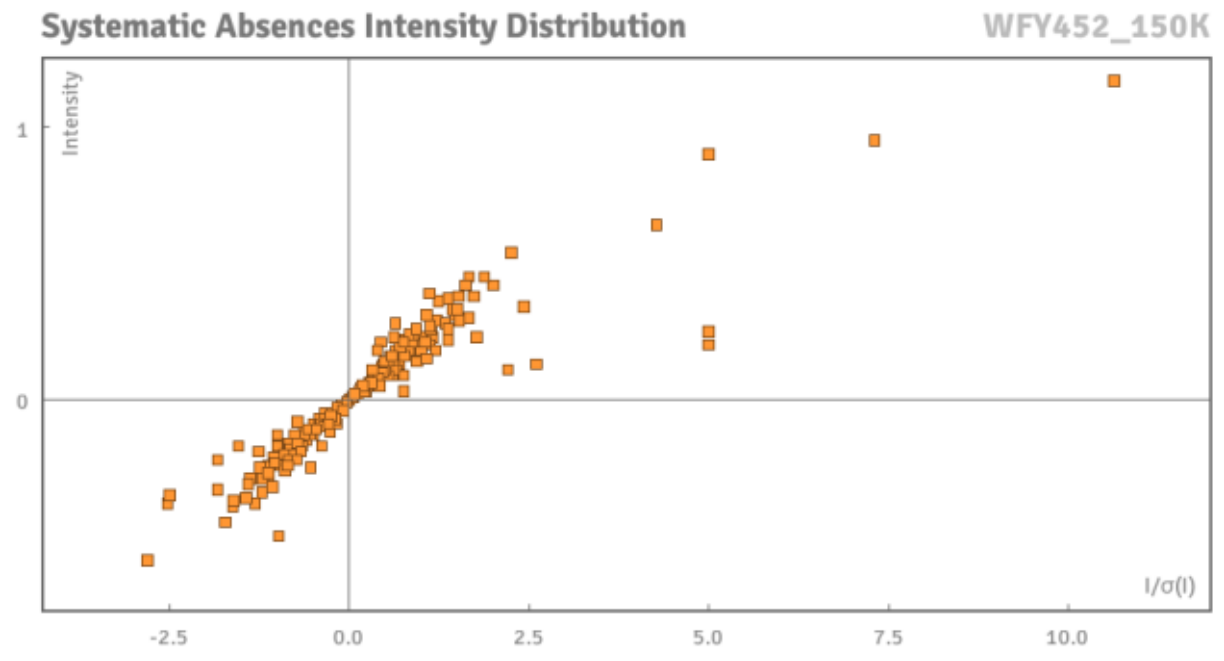

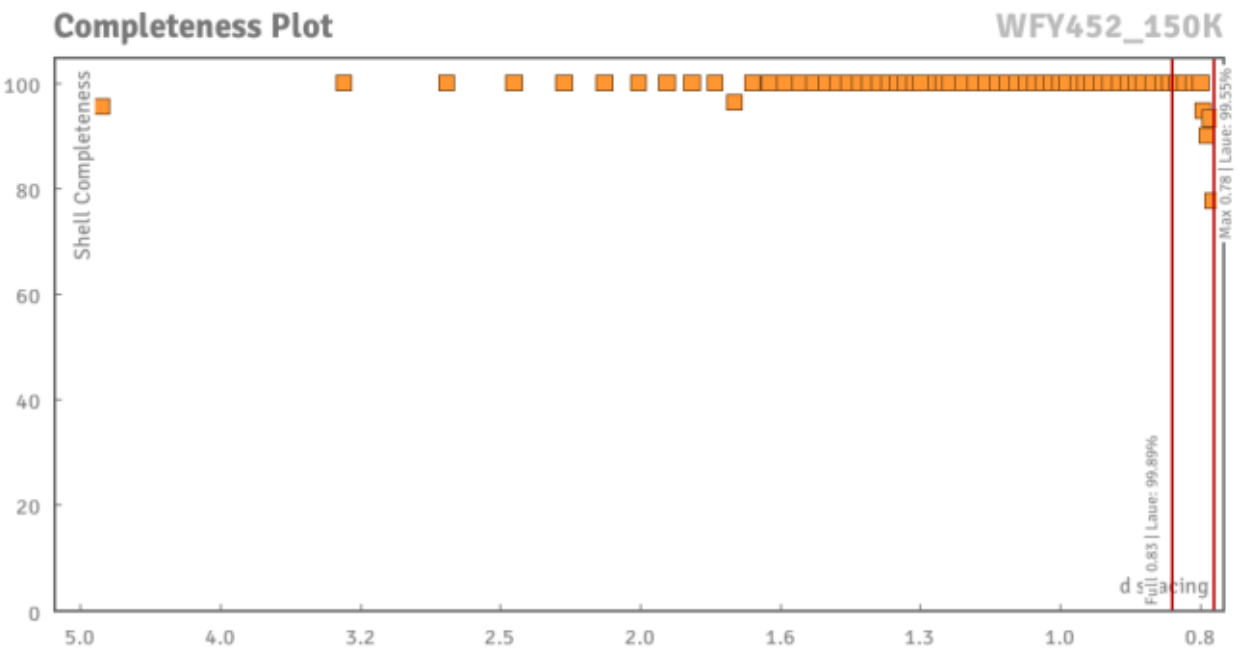

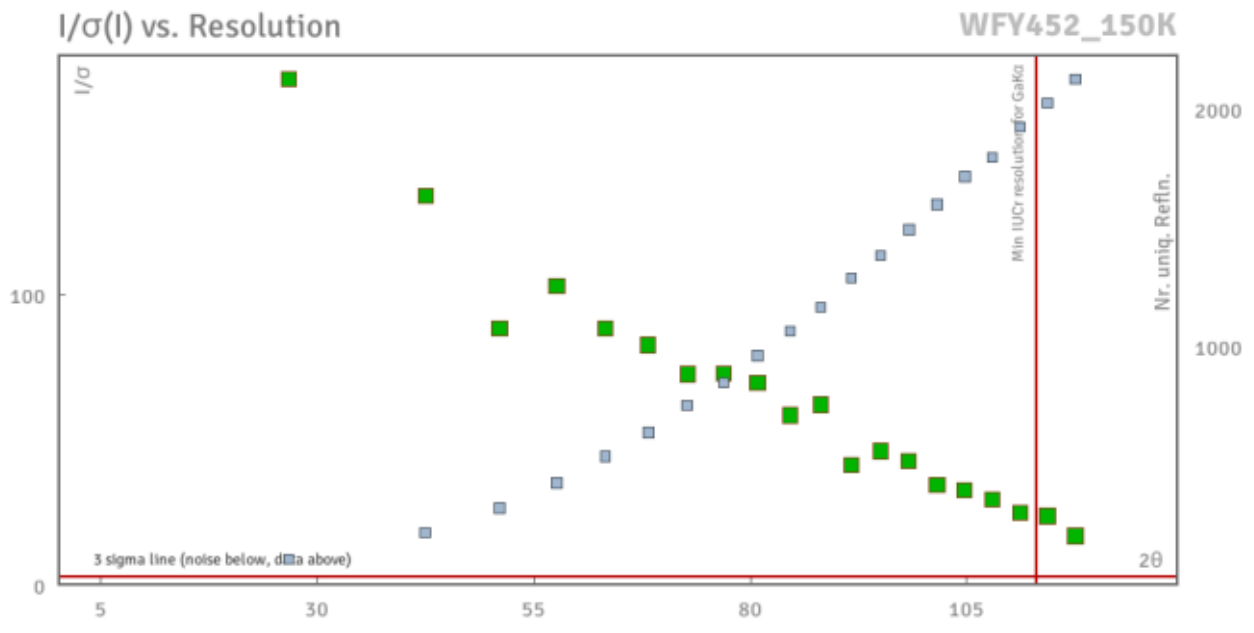

Data Plots: Refinement and Data

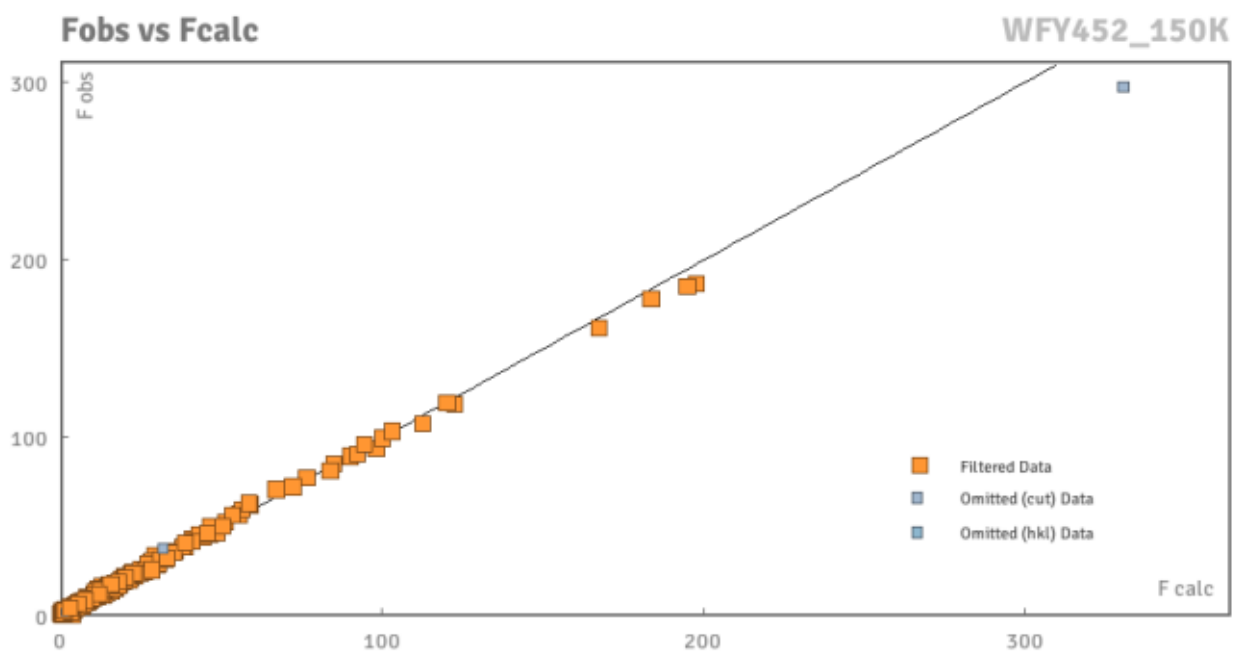

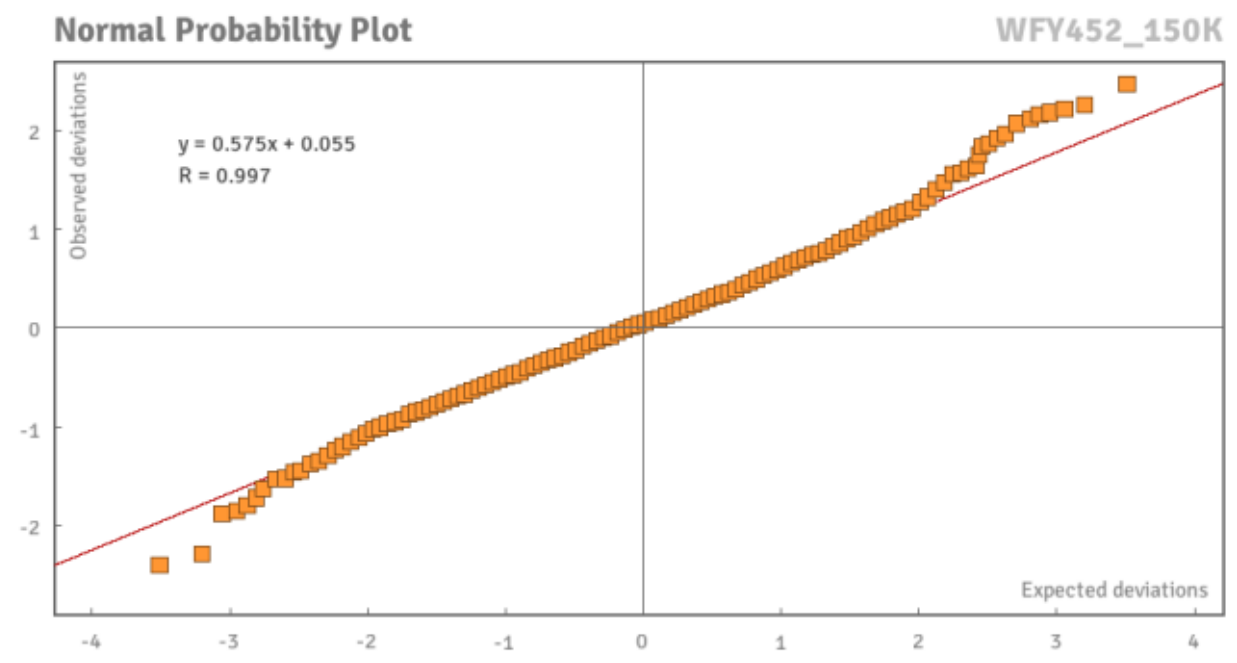

Reflection Statistics

Total reflections (after filtering)	15818	Unique reflections	2224
Completeness	0.996	Mean I/σ	65.08
hkl_{max} collected	(13, 21, 15)	hkl_{min} collected	(-12, -16, -14)
hkl_{max} used	(12, 21, 15)	hkl_{min} used	(-13, 0, 0)
Lim d_{max} collected	100.0	Lim d_{min} collected	0.67
d_{max} used	9.41	d_{min} used	0.78
Friedel pairs	844	Friedel pairs merged	1
Inconsistent equivalents	57	R_{int}	0.0171
R_{sigma}	0.0077	Intensity transformed	0
Omitted reflections	0	Omitted by user (OMIT hkl)	2
Multiplicity	(1114, 1183, 1031, 781, 487, 314, 155, 60, 21, 5)	Maximum multiplicity	17
Removed systematic absences	342	Filtered off (Shel/OMIT)	0

Table 14: Fractional Atomic Coordinates (×10⁴) and Equivalent Isotropic Displacement Parameters (Å²×10³) for **WFY452_150K.** U_{eq} is defined as 1/3 of the trace of the orthogonalised U_{ij}.

Atom	x	y	z	U_{eq}
O2	5258.8(9)	2775.9(6)	2522.9(7)	42.8(3)
O1	4263.8(9)	2505.3(7)	3837.8(8)	48.8(3)
C1	5252.0(13)	2606.7(7)	3592.0(10)	35.4(3)

Atom	x	y	z	U_{eq}
C2	6636.4(12)	2587.1(7)	4451.1(10)	32.1(3)
C5	3961.3(14)	2926.9(10)	1625.2(11)	48.5(4)
C3	7084.4(12)	2220.9(9)	5687.9(11)	42.0(3)
C6	3586.5(15)	3807.0(10)	1657.5(12)	52.5(4)
C4	7500	3339.1(11)	5000	50.6(5)
C11	6742.8(18)	5664.5(9)	4718.2(16)	60.4(5)
C7	4509.8(17)	4395.5(11)	1340.3(13)	59.2(5)
C9	4734.9(19)	5525.2(10)	2843.2(17)	66.7(5)
C10	6245.6(18)	5575.2(9)	3403.8(16)	62.6(5)
C8	4248(2)	5284.3(12)	1559.6(17)	72.4(6)

Table 15: Anisotropic Displacement Parameters (×10^4) for **WFY452_150K.** The anisotropic displacement factor exponent takes the form: $-2\pi^2[h^2a^{*2} \times U_{11}+ ... +2hka^* \times b^* \times U_{12}]$

Atom	U_{11}	U_{22}	U_{33}	U_{23}	U_{13}	U_{12}
O2	40.6(5)	59.9(6)	30.4(4)	10.1(4)	15.5(4)	19.5(4)
O1	39.1(5)	74.2(7)	34.6(5)	9.4(4)	14.5(4)	4.3(4)
C1	41.7(7)	37.1(6)	29.0(6)	3.9(4)	14.5(5)	11.1(5)
C2	38.6(6)	31.2(5)	29.0(5)	1.8(4)	14.7(5)	4.0(4)
C5	45.0(7)	72.6(9)	28.2(6)	9.9(6)	13.2(5)	23.6(7)
C3	35.0(6)	56.0(8)	34.2(6)	10.5(5)	10.9(5)	-2.0(5)
C6	50.7(8)	74.0(10)	40.5(7)	23.2(7)	25.3(6)	31.8(7)
C4	60.5(12)	31.1(9)	54.0(11)	0	11.7(9)	0
C11	87.7(12)	36.2(7)	86.8(12)	8.3(7)	68.0(11)	8.8(7)
C7	65.3(9)	79.3(11)	46.3(8)	27.6(7)	36.5(7)	31.2(8)
C9	85.9(12)	51.7(8)	89.5(13)	29.5(8)	64.7(11)	32.1(8)
C10	85.7(12)	45.0(8)	85.6(12)	19.1(7)	66.0(11)	17.8(7)
C8	83.8(12)	75.0(11)	76.7(12)	47.2(10)	51.2(10)	40.5(10)

Table 16: Bond Lengths in Å for **WFY452_150K.**

Atom	Atom	Length/Å
O2	C1	1.3368(14)
O2	C5	1.4582(15)
O1	C1	1.2002(16)
C1	C2	1.4854(17)
C2	C2[1]	1.864(2)
C2	C3[1]	1.5503(16)
C2	C3	1.5409(16)
C2	C4	1.5502(19)
C5	C6	1.508(2)
C6	C7	1.522(2)
C11	C11[1]	1.518(4)
C11	C10	1.514(3)
C7	C8	1.531(3)
C9	C10	1.517(3)
C9	C8	1.524(3)

----
[1]3/2-x,+y,1-z

Table 17: Bond Angles in ° for **WFY452_150K.**

Atom	Atom	Atom	Angle/°
C1	O2	C5	116.57(10)
O2	C1	C2	110.88(10)
O1	C1	O2	124.93(11)
O1	C1	C2	124.15(11)
C1	C2	C2[1]	178.46(9)
C1	C2	C3	126.67(10)
C1	C2	C3[1]	128.72(10)
C1	C2	C4	125.72(9)
C3[1]	C2	C2[1]	52.67(7)
C3	C2	C2[1]	53.14(7)
C3	C2	C3[1]	88.16(11)
C3	C2	C4	87.25(8)
C4	C2	C2[1]	53.03(6)
C4	C2	C3[1]	86.92(9)
O2	C5	C6	109.56(13)
C2	C3	C2[1]	74.19(9)
C5	C6	C7	113.96(12)
C2[1]	C4	C2	73.94(12)
C10	C11	C11[1]	114.16(15)

Atom	Atom	Atom	Angle/°
C6	C7	C8	113.10(12)
C10	C9	C8	114.23(13)
C11	C10	C9	114.38(13)

Atom	Atom	Atom	Angle/°
C9	C8	C7	114.26(12)

----
[1]3/2-x,+y,1-z

Table 18: Torsion Angles in ° for **WFY452_150K**.

Atom	Atom	Atom	Atom	Angle/°
O2	C1	C2	C3	-164.25(12)
O2	C1	C2	C3[1]	-41.18(17)
O2	C1	C2	C4	78.26(13)
O2	C5	C6	C7	65.06(15)
O1	C1	C2	C3[1]	140.68(14)
O1	C1	C2	C3	17.6(2)
O1	C1	C2	C4	-99.88(15)
C1	O2	C5	C6	85.57(14)
C1	C2	C3	C2[1]	-178.09(10)
C1	C2	C4	C2[1]	178.89(15)
C5	O2	C1	O1	6.49(19)
C5	O2	C1	C2	-171.63(11)
C5	C6	C7	C8	-171.09(14)
C3[1]	C2	C3	C2[1]	42.77(11)
C3	C2	C4	C2[1]	44.31(6)
C3[1]	C2	C4	C2[1]	-43.99(6)
C6	C7	C8	C9	73.6(2)
C4	C2	C3	C2[1]	-44.23(7)
C11[1]	C11	C10	C9	176.59(9)
C10	C9	C8	C7	71.34(19)
C8	C9	C10	C11	-169.56(13)

----
[1]3/2-x,+y,1-z

Table 19: Hydrogen Fractional Atomic Coordinates (×10^4) and Equivalent Isotropic Displacement Parameters (Å^2×10^3) for **WFY452_150K.** U_{eq} is defined as 1/3 of the trace of the orthogonalised U_{ij}.

Atom	x	y	z	U_{eq}
H5A	3272.95	2576.81	1761.37	58
H5B	4004.4	2793.69	847.98	58
H3A	7055.02	1621.42	5732.88	50
H3B	6747.99	2496.05	6253.47	50
H6A	3594.52	3937.78	2451.58	63
H6B	2661.28	3887.54	1109.95	63
H4A	7813.77	3670.32	4467.92	61
H4B	7186.22	3670.32	5532.08	61
H11A	6375.63	5213.87	5052.79	72
H11B	6390.57	6178.58	4918.38	72
H7A	4393.09	4325.83	505.34	71
H7B	5449.87	4259.85	1802.96	71
H9A	4347.15	6060.07	2917.17	80
H9B	4394.53	5125.03	3278.38	80
H10A	6643.15	5078.5	3202.51	75
H10B	6568.25	6043.85	3066.73	75
H8A	4694.08	5634.99	1145.1	87
H8B	3273.49	5388.73	1221.06	87

Table 20: Atomic Occupancies for all atoms that are not fully occupied in **WFY452_150K**.

Atom	Occupancy
H4A	0.5
H4B	0.5

WFY490_150K

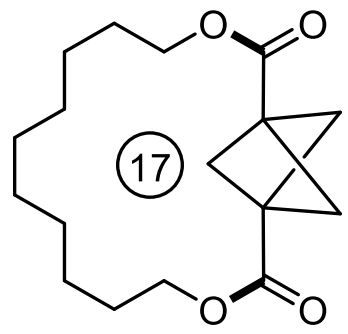

3ad (CCDC 2514450)

R_1=4.01%

Submitted by:	**Fengyuan Wang**
	University of Basel
Solved by:	**Alessandro Prescimone**
Sample ID:	**WFY490**

Crystal Data and Experimental

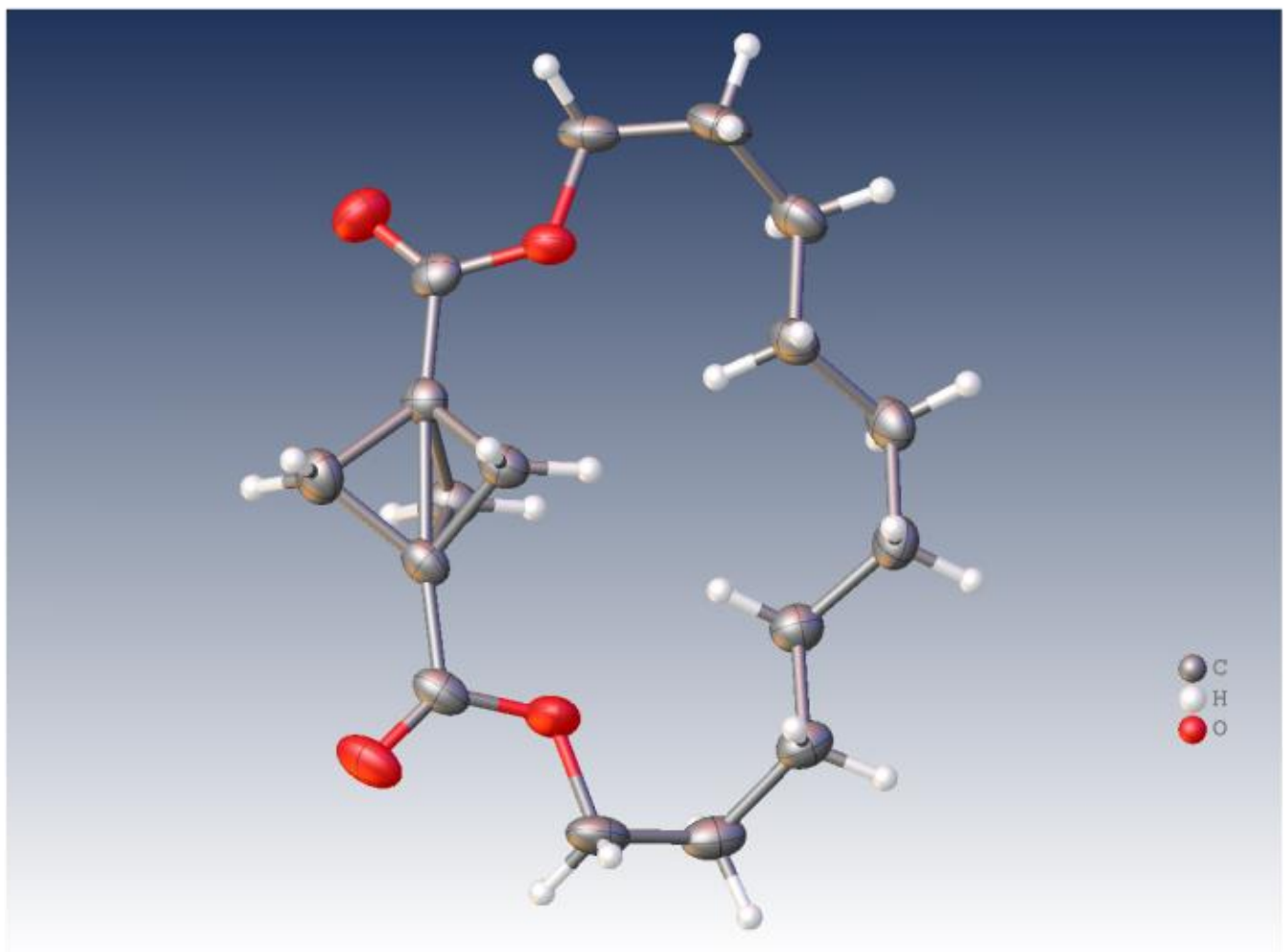

Experimental. Single colourless needle-shaped crystals of **WFY490_150K** were used as supplied. A suitable crystal with dimensions 0.40 × 0.20 × 0.10 mm was selected and mounted on a STOE STADIVARI diffractometer. The crystal was kept at a steady T = 150 K during data collection. The structure was solved with the ShelXT 2018/2 (Sheldrick, 2018) solution program using dual methods and by using Olex2 1.5 (Dolomanov et al., 2009) as the graphical interface. The model was refined with ShelXL 2019/3 (Sheldrick, 2015) using full matrix least squares minimisation on $|F|^2$.

Crystal Data. $C_{17}H_{26}O_4$, M_r = 294.38, monoclinic, $P2_1/c$ (No. 14), a = 5.3580(4) Å, b = 16.3306(9) Å, c = 18.7972(14) Å, β = 97.039(6)°, $\alpha = \gamma$ = 90°, V = 1632.3(2) Å^3, T = 150 K, Z = 4, Z' = 1, $\mu(GaK_\alpha)$ = 0.448, 16032 reflections measured, 3582 unique (R_{int} = 0.0321) which were used in all calculations. The final wR_2 was 0.1095 (all data) and R_1 was 0.0401 (I≥2σ(I)).

**Compound**	**WFY490_150K**
Formula	$C_{17}H_{26}O_4$
$D_{calc.}$/ g cm^{-3}	1.198
μ/mm^{-1}	0.448
Formula Weight	294.38
Colour	colourless
Shape	needle-shaped
Size/mm	0.40×0.20×0.10
T/K	150
Crystal System	monoclinic
Space Group	$P2_1/c$
a/Å	5.3580(4)
b/Å	16.3306(9)
c/Å	18.7972(14)
α/°	90
β/°	97.039(6)
γ/°	90
V/Å^3	1632.3(2)
Z	4
Z'	1
Wavelength/Å	1.34143
Radiation type	GaK_α
Θ_{min}/°	3.129
Θ_{max}/°	59.608
Index range h	-3 ≥ h ≥ 6
Index range k	-20 ≥ k ≥ 20
Index range l	-23 ≥ l ≥ 23
Measured Refl's.	16032
Indep't Refl's	3582
Refl's I≥2σ(I)	3112
R_{int}	0.0321
Parameters	191
Restraints	0
Largest Peak/eÅ^3	0.242
Deepest Hole/eÅ^3	-0.238
GooF	1.054
R_1 (I≥2σ(I) / all)	0.0401 / 0.0468
wR_2 (I≥2σ(I) / all)	0.1051 / 0.1095

Structure Quality Indicators

**Reflections:**	d min (GaKα) 2Θ=119.2° 0.78	I/σ(I) 54.4	Rint m=4.57 3.21%	Full 107.2° 99.5
**Refinement:**	Shift 0.001	Max Peak 0.2	Min Peak -0.2	GooF 1.054

A colourless needle-shaped crystal with dimensions 0.40 × 0.20 × 0.10 mm was mounted. Data were collected using a STOE STADIVARI diffractometer equipped with an Oxford Cryosystems low-temperature device operating at T = 150 K.

Data were measured using rotation method, ω scans with GaK_{α} radiation. The diffraction pattern was indexed and the total number of runs and images was based on the strategy calculation from the program X-Area Pilatus3_SV 1.31.186.0 (STOE, 2022) . The maximum resolution achieved was Θ= 59.608° (0.78 Å).

The unit cell was refined using X-Area Pilatus3_SV 1.31.186.0 (STOE, 2022) on 25634 reflections, 160% of the observed reflections.

Data reduction, scaling and absorption corrections were performed using X-Area Pilatus3_SV 1.31.186.0 (STOE, 2022). The final completeness is 99.50 % out to 59.608° in Θ. A multi-scan absorption correction was performed using STOE. The absorption coefficient μ of this material is 0.448 mm^{-1} at this wavelength (λ = 1.34143Å) and the minimum and maximum transmissions are 0.667 and 0.849.

The structure was solved in the space group $P2_1/c$ (# 14) by ShelXT 2018/2 (Sheldrick, 2018) using dual methods. It was refined by full matrix least squares minimisation on $|F|^2$ using version 2019/3 of ShelXL 2019/3 (Sheldrick, 2015). All non-hydrogen atoms were refined anisotropically.

Hydrogen atom positions were calculated geometrically and refined using the riding model.

_exptl_absorpt_process_details: STOE X-Red32, absorption correction by Gaussian integration, analogous toP. Coppens in: F. R. Ahmed (Editor), "Crystallographic Computing", Munksgaard,Copenhagen (1970), 255 - 270. Afterwards scaling of reflection intensities was performed within STOE LANA. J. Koziskova, F. Hahn, J. Richter, J. Kozisek, Acta Chimica Slovaca, vol. 9, no. 2, 2016, pp. 136 - 140. Finally, a spherical absorption correction was done within STOE LANA.

There is a single formula unit in the asymmetric unit, which is represented by the reported sum formula. In other words: Z is 4 and Z' is 1. The moiety formula is C17 H26 O4.

Data Plots: Diffraction Data

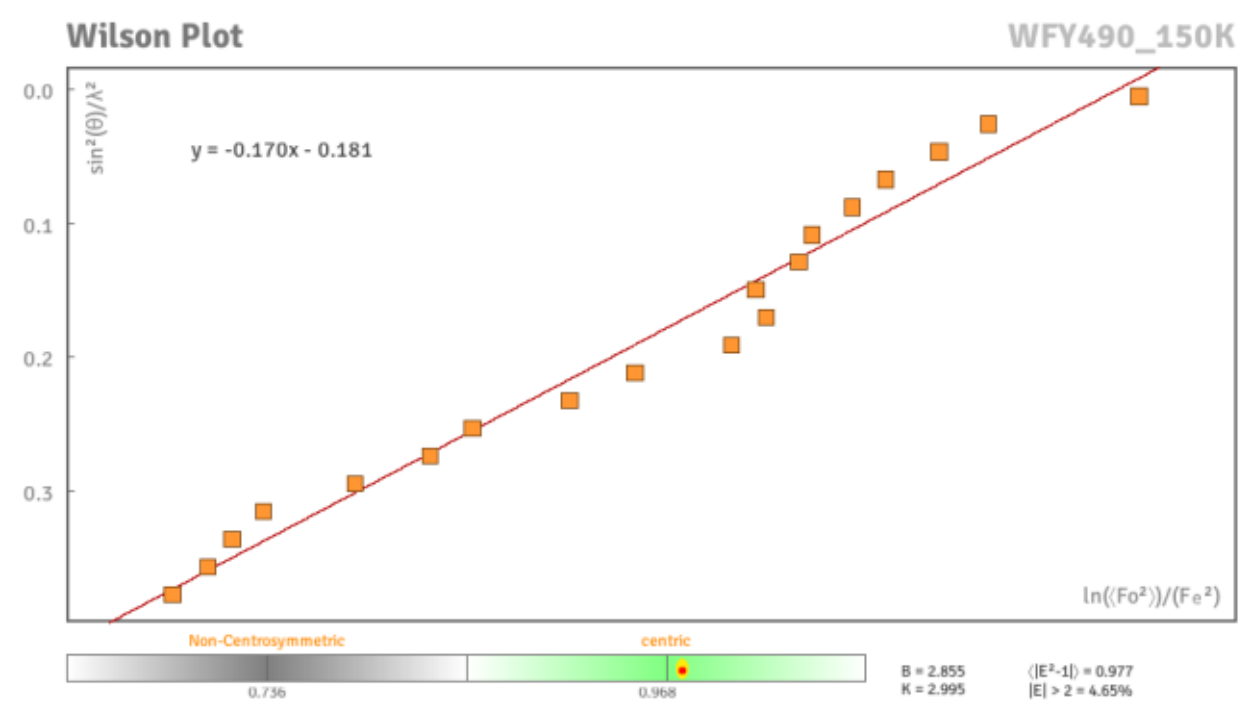

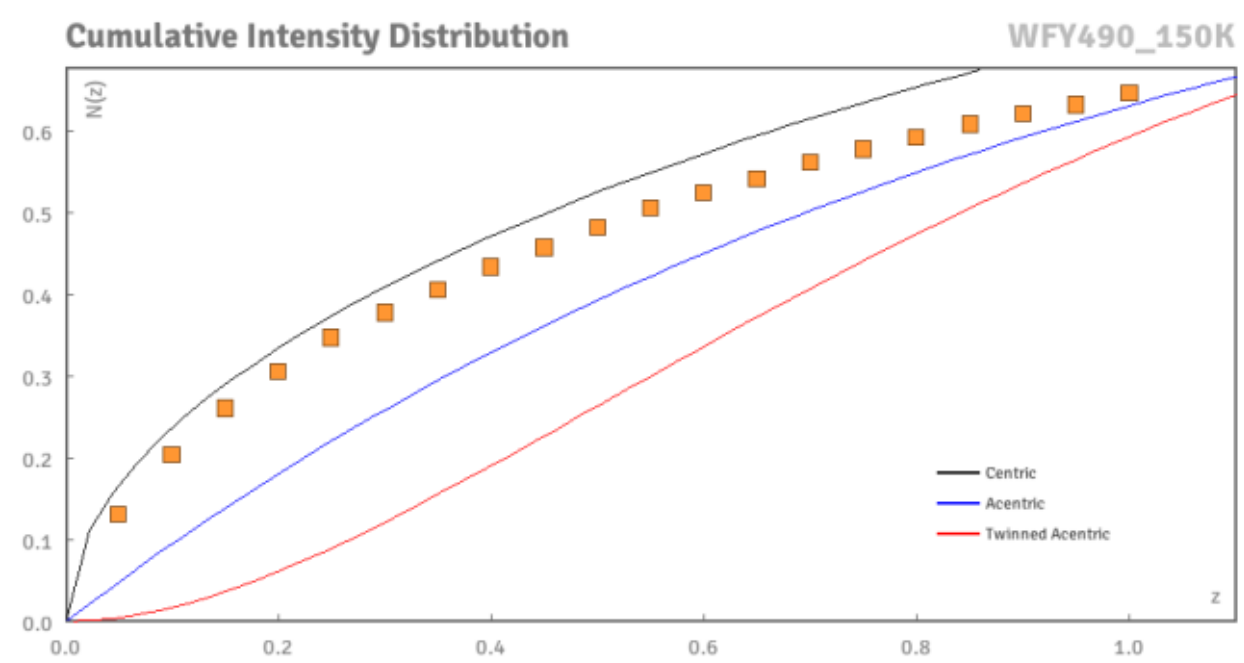

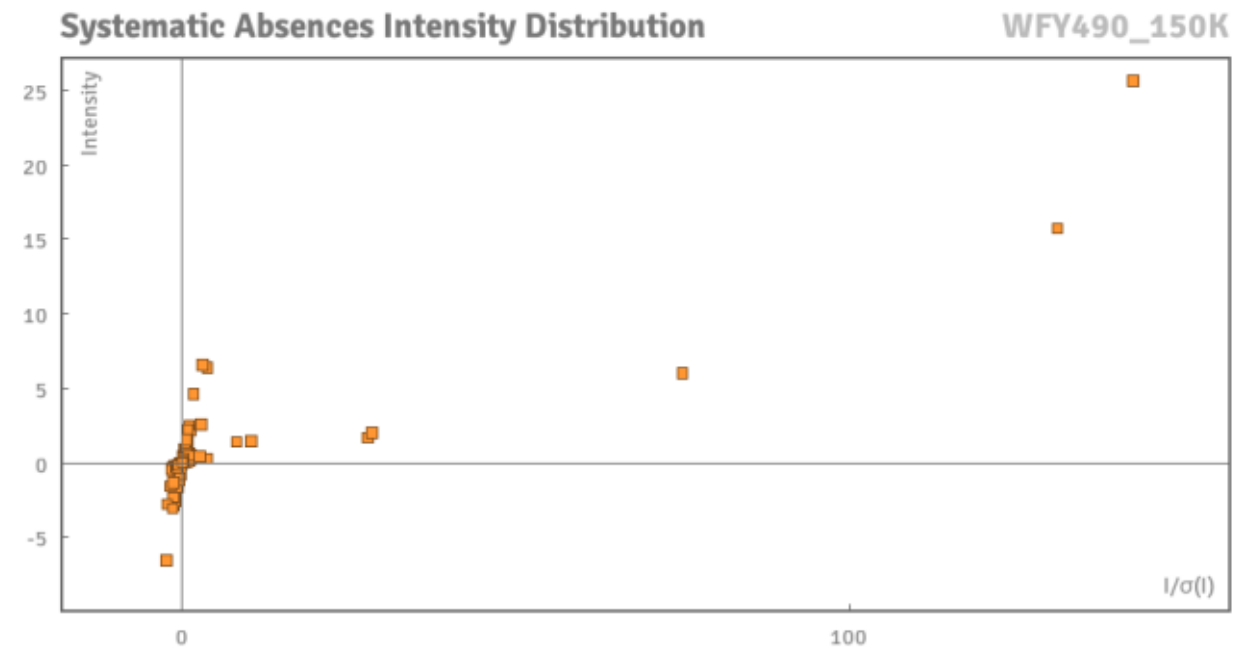

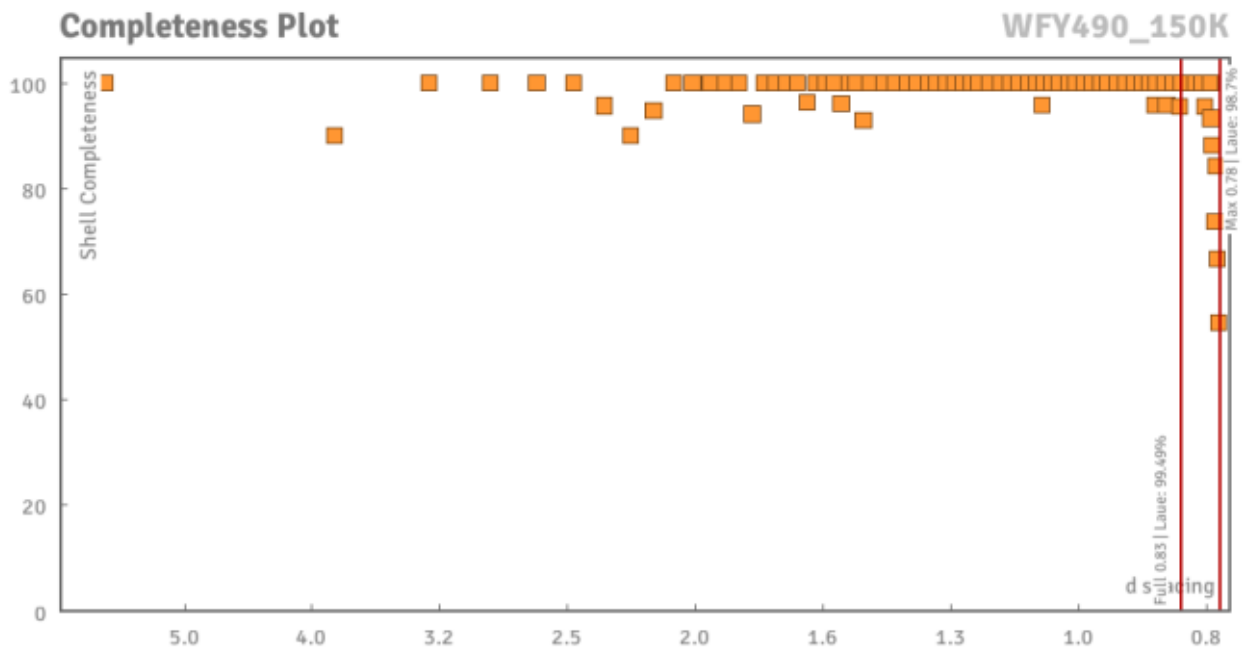

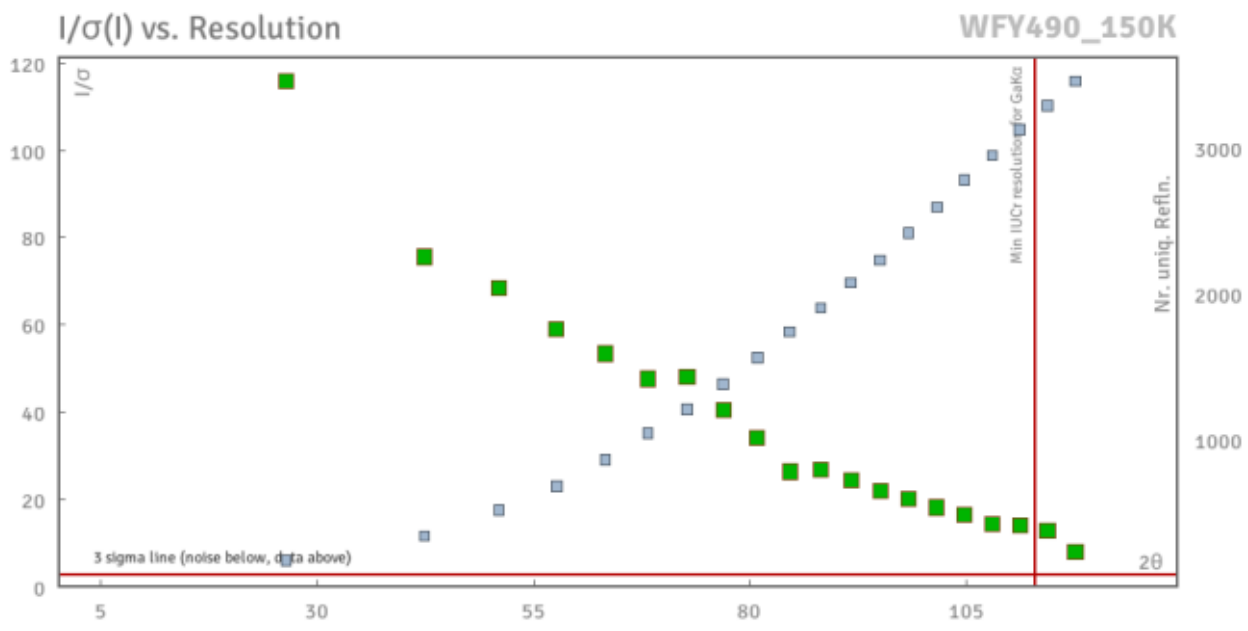

Data Plots: Refinement and Data

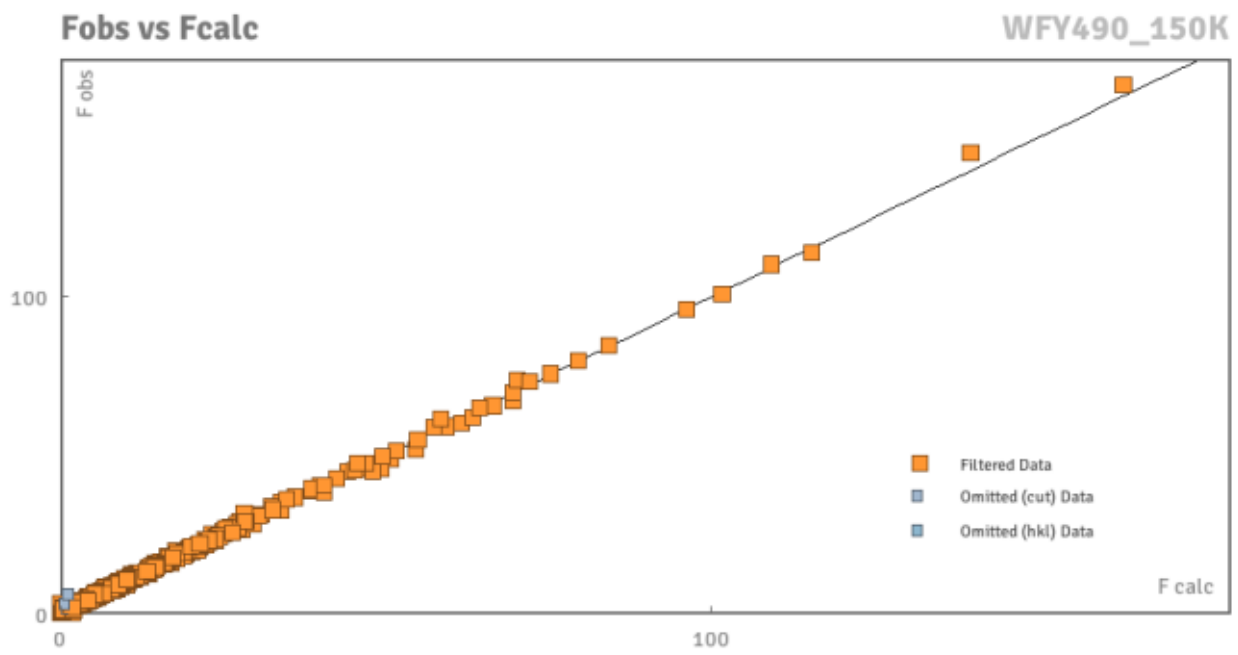

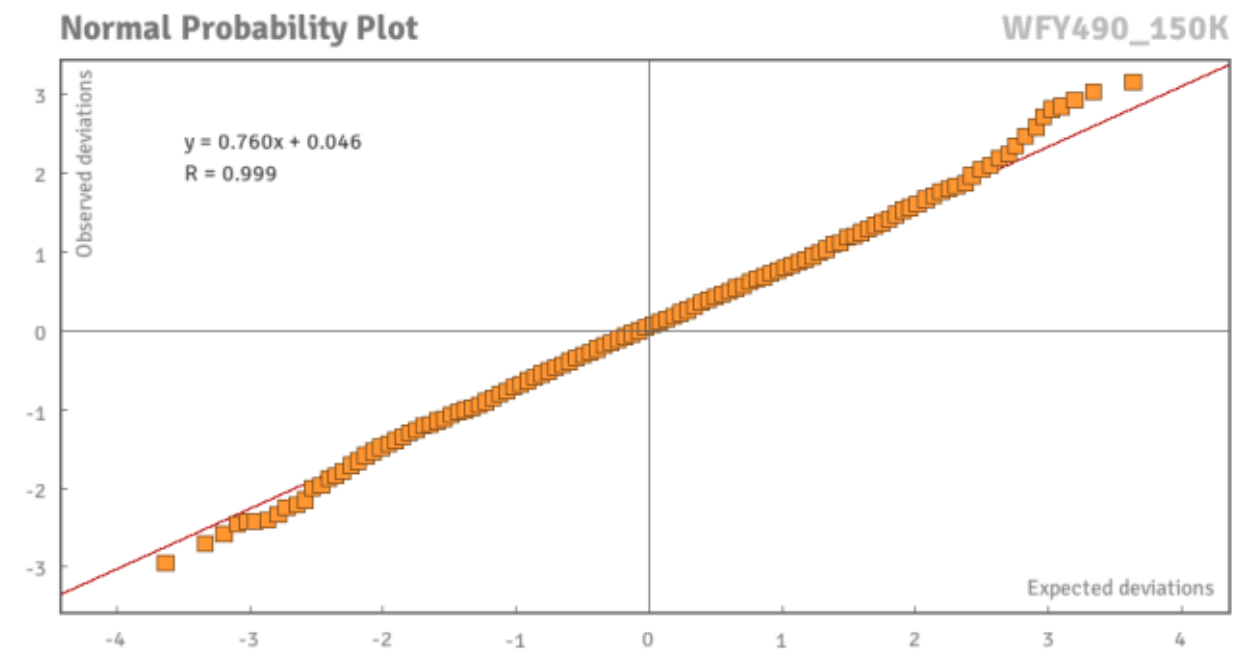

Reflection Statistics

Total reflections (after filtering)	16375	Unique reflections	3582
Completeness	0.987	Mean I/σ	37.47
hkl_{max} collected	(6, 20, 23)	hkl_{min} collected	(-3, -20, -23)
hkl_{max} used	(6, 20, 23)	hkl_{min} used	(-6, 0, 0)
Lim d_{max} collected	100.0	Lim d_{min} collected	0.67
d_{max} used	12.29	d_{min} used	0.78
Friedel pairs	799	Friedel pairs merged	1
Inconsistent equivalents	211	R_{int}	0.0321
R_{sigma}	0.0184	Intensity transformed	0
Omitted reflections	0	Omitted by user (OMIT hkl)	4
Multiplicity	(2724, 2350, 1450, 724, 276, 49, 5)	Maximum multiplicity	13
Removed systematic absences	343	Filtered off (Shel/OMIT)	0

Table 21: Fractional Atomic Coordinates (×10^4) and Equivalent Isotropic Displacement Parameters (Å^2×10^3) for **WFY490_150K.** U_{eq} is defined as 1/3 of the trace of the orthogonalised U_{ij}.

Atom	x	y	z	U_{eq}
O4	5418.5(15)	4256.7(5)	7014.9(5)	35.6(2)
O1	11434.2(17)	956.1(6)	7701.9(6)	48.9(3)
O3	5090.9(19)	4243.1(6)	8196.9(5)	48.5(3)

Atom	x	y	z	U_{eq}
O2	9005(2)	1219.0(6)	6668.1(6)	52.3(3)
C5	5914(2)	3968.3(7)	7680.8(7)	31.7(3)
C3	9161(2)	2988.8(7)	7072.4(7)	30.4(2)
C2	8977.1(19)	2200.0(7)	7522.4(6)	30.5(2)
C4	7436.4(19)	3205.3(7)	7648.0(6)	29.6(2)
C7	6115.7(19)	2366.8(7)	7481.1(7)	32.0(3)
C1	10016(2)	1389.8(7)	7334.3(7)	33.5(3)
C14	5522(2)	1751.8(7)	5311.2(7)	36.5(3)
C11	4134(2)	3673.6(7)	5500.5(7)	36.2(3)
C8	3572(2)	4905.1(7)	6874.5(8)	37.4(3)
C12	3206(2)	3084.7(8)	4897.2(7)	39.1(3)
C15	7470(2)	1116.6(7)	5148.0(7)	37.4(3)
C13	5120(2)	2427.6(8)	4752.0(7)	37.7(3)
C16	7745(2)	381.2(7)	5660.6(8)	41.0(3)
C9	3343(2)	5081.8(7)	6082.6(8)	42.7(3)
C10	2330(2)	4383.5(8)	5582.5(8)	41.0(3)
C17	9640(2)	473.9(7)	6314.2(8)	42.4(3)
C6	9329(2)	2746.9(8)	8203.4(7)	40.1(3)

Table 22: Anisotropic Displacement Parameters (×10[4]) for **WFY490_150K.** The anisotropic displacement factor exponent takes the form: $-2\pi^2[h^2a^{*2} \times U_{11}+ ... +2hka^* \times b^* \times U_{12}]$

Atom	U_{11}	U_{22}	U_{33}	U_{23}	U_{13}	U_{12}
O4	37.3(4)	27.7(4)	40.9(5)	-2.8(3)	2.1(3)	9.3(3)
O1	38.8(5)	38.5(5)	65.6(7)	13.0(5)	-9.3(4)	8.7(4)
O3	54.6(6)	48.3(6)	43.9(6)	-10.6(4)	10.8(4)	9.3(4)
O2	70.8(7)	36.5(5)	46.0(6)	-4.2(4)	-7.2(5)	27.5(4)
C5	28.6(5)	28.6(6)	37.0(6)	-6.8(5)	0.5(4)	-3.2(4)
C3	27.5(5)	26.6(5)	36.9(6)	2.6(5)	3.3(4)	0.7(4)
C2	25.0(5)	30.8(6)	34.7(6)	6.2(5)	-0.2(4)	2.5(4)
C4	26.0(5)	29.8(6)	31.7(6)	-2.0(4)	-0.9(4)	-0.6(4)
C7	25.5(5)	28.2(5)	41.9(6)	1.3(5)	3.3(4)	-0.1(4)
C1	25.3(5)	28.8(6)	46.3(7)	10.4(5)	3.9(4)	1.6(4)
C14	36.4(6)	34.0(6)	39.5(7)	-1.7(5)	6.9(5)	0.3(5)
C11	33.3(6)	31.9(6)	40.4(7)	3.0(5)	-7.1(5)	4.5(4)
C8	32.6(6)	21.9(5)	57.3(8)	-3.8(5)	3.5(5)	5.1(4)
C12	36.6(6)	39.0(7)	38.4(7)	4.1(5)	-8.9(5)	-1.3(5)
C15	39.2(6)	32.0(6)	42.4(7)	-6.7(5)	10.3(5)	-3.5(5)
C13	40.7(6)	39.9(7)	31.2(6)	-3.2(5)	-1.4(5)	-3.1(5)
C16	42.6(6)	26.5(6)	56.3(8)	-5.3(5)	15.8(6)	-1.2(5)
C9	41.8(6)	24.5(6)	60.8(9)	8.8(6)	2.8(6)	6.3(5)
C10	35.9(6)	35.8(6)	48.5(8)	8.1(6)	-5.3(5)	8.1(5)
C17	46.2(7)	26.5(6)	56.6(8)	2.1(5)	14.5(6)	11.0(5)
C6	36.6(6)	47.0(7)	34.7(7)	1.4(5)	-4.2(5)	4.9(5)

Table 23: Bond Lengths in Å for **WFY490_150K**.

Atom	Atom	Length/Å
O4	C5	1.3332(15)
O4	C8	1.4510(13)
O1	C1	1.1949(15)
O3	C5	1.2007(15)
O2	C1	1.3317(16)
O2	C17	1.4472(15)
C5	C4	1.4947(15)
C3	C2	1.5508(15)

Atom	Atom	Length/Å
C3	C4	1.5475(15)
C2	C4	1.8656(16)
C2	C7	1.5497(14)
C2	C1	1.4946(16)
C2	C6	1.5534(18)
C4	C7	1.5556(15)
C4	C6	1.5555(16)
C14	C15	1.5293(16)

Atom	Atom	Length/Å
C14	C13	1.5210(18)
C11	C12	1.5232(18)
C11	C10	1.5290(16)
C8	C9	1.506(2)
C12	C13	1.5320(18)
C15	C16	1.5355(19)
C16	C17	1.503(2)
C9	C10	1.534(2)

Table 24: Bond Angles in ° for **WFY490_150K.**

Atom	Atom	Atom	Angle/°
C5	O4	C8	118.78(9)
C1	O2	C17	121.12(10)
O4	C5	C4	107.61(9)
O3	C5	O4	125.17(11)
O3	C5	C4	126.96(12)
C4	C3	C2	74.05(8)
C3	C2	C4	52.89(6)
C3	C2	C6	87.85(9)
C7	C2	C3	87.44(8)
C7	C2	C4	53.22(6)
C7	C2	C6	87.69(9)
C1	C2	C3	123.77(10)
C1	C2	C4	172.94(10)
C1	C2	C7	122.60(10)
C1	C2	C6	133.86(10)
C6	C2	C4	53.18(7)
C5	C4	C3	126.52(10)
C5	C4	C2	172.32(9)
C5	C4	C7	120.33(9)
C5	C4	C6	133.45(10)
C3	C4	C2	53.06(6)
C3	C4	C7	87.35(8)
C3	C4	C6	87.89(8)
C7	C4	C2	52.93(6)
C6	C4	C2	53.07(7)
C6	C4	C7	87.40(9)
C2	C7	C4	73.85(7)
O1	C1	O2	125.19(12)
O1	C1	C2	128.09(13)
O2	C1	C2	106.70(9)
C13	C14	C15	113.32(11)
C12	C11	C10	113.64(10)
O4	C8	C9	106.90(10)
C11	C12	C13	114.22(10)
C14	C15	C16	114.83(10)
C14	C13	C12	115.23(10)
C17	C16	C15	115.94(10)
C8	C9	C10	116.29(11)
C11	C10	C9	116.41(10)
O2	C17	C16	106.69(10)
C2	C6	C4	73.75(8)

Table 25: Torsion Angles in ° for **WFY490_150K.**

Atom	Atom	Atom	Atom	Angle/°
O4	C5	C4	C3	26.00(14)
O4	C5	C4	C7	-85.51(12)
O4	C5	C4	C6	155.68(12)
O4	C8	C9	C10	63.89(13)
O3	C5	C4	C3	-159.72(12)
O3	C5	C4	C7	88.77(15)
O3	C5	C4	C6	-30.0(2)
C5	O4	C8	C9	-178.01(10)
C5	C4	C7	C2	175.57(11)
C5	C4	C6	C2	174.68(13)
C3	C2	C4	C7	119.71(10)
C3	C2	C4	C6	-120.50(10)
C3	C2	C7	C4	-43.90(8)
C3	C2	C1	O1	126.20(14)
C3	C2	C1	O2	-55.33(14)
C3	C2	C6	C4	43.44(7)
C3	C4	C7	C2	44.02(8)
C3	C4	C6	C2	-43.56(8)
C2	C3	C4	C5	-170.43(11)
C2	C3	C4	C7	-43.93(7)
C2	C3	C4	C6	43.57(8)
C4	C3	C2	C7	44.14(8)
C4	C3	C2	C1	172.36(11)
C4	C3	C2	C6	-43.64(8)

Atom	Atom	Atom	Atom	Angle/°
C7	C2	C4	C3	-119.71(10)
C7	C2	C4	C6	119.79(11)
C7	C2	C1	O1	-122.47(14)
C7	C2	C1	O2	56.00(14)
C7	C2	C6	C4	-44.08(8)
C7	C4	C6	C2	43.88(8)
C1	O2	C17	C16	166.67(11)
C1	C2	C7	C4	-173.08(12)
C1	C2	C6	C4	-179.23(13)
C14	C15	C16	C17	-89.54(13)
C11	C12	C13	C14	73.31(14)
C8	O4	C5	O3	-5.95(17)
C8	O4	C5	C4	168.46(9)
C8	C9	C10	C11	-74.21(15)
C12	C11	C10	C9	-169.18(12)
C15	C14	C13	C12	-179.29(10)
C15	C16	C17	O2	54.13(14)
C13	C14	C15	C16	-174.41(11)
C10	C11	C12	C13	173.33(11)
C17	O2	C1	O1	-2.2(2)
C17	O2	C1	C2	179.30(11)
C6	C2	C4	C3	120.50(10)
C6	C2	C4	C7	-119.79(11)
C6	C2	C7	C4	44.05(8)
C6	C2	C1	O1	0.8(2)
C6	C2	C1	O2	179.24(12)
C6	C4	C7	C2	-43.98(8)

Table 26: Hydrogen Fractional Atomic Coordinates (×10[4]) and Equivalent Isotropic Displacement Parameters (Å[2]×10[3]) for **WFY490_150K.** U_{eq} is defined as 1/3 of the trace of the orthogonalised U_{ij}.

Atom	x	y	z	U_{eq}
H3A	10823.58	3261.61	7121.13	36
H3B	8342.27	2967.92	6571.2	36
H7A	5255.78	2127.32	7869.25	38
H7B	5136.38	2312.01	7001.5	38
H14A	3899.44	1471.78	5341.51	44
H14B	6071.14	2001.12	5784.56	44
H11A	5780.37	3899.22	5409.5	43
H11B	4396.94	3365.23	5957	43
H8A	4126.74	5401.4	7152.46	45
H8B	1930.78	4728.65	7012.63	45
H12A	2747.34	3403.92	4452.65	47
H12B	1662.61	2811.12	5017.5	47
H15A	9124.54	1390.54	5163.65	45
H15B	7006.53	911.77	4653.81	45
H13A	4561.95	2174.28	4280.79	45
H13B	6755.2	2697.63	4717.72	45
H16A	6084.2	268.82	5819.73	49
H16B	8209.54	-104.01	5389.7	49
H9A	5024.89	5237.67	5960.21	51
H9B	2227.07	5561.85	5982.99	51
H10A	805.4	4159.62	5760.49	49
H10B	1807.19	4617.37	5101.44	49
H17A	9564.91	-1.83	6637.32	51
H17B	11360.01	511.2	6175.42	51
H6A	11001.68	3004.86	8319.02	48
H6B	8640.41	2528.14	8630.29	48

WFY375_150K

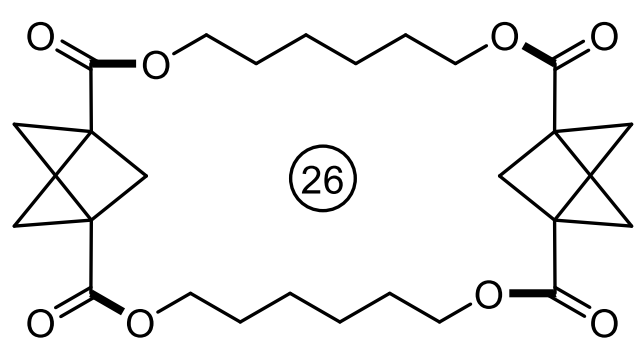

***R₁*=6.30%**

Submitted by: **Fengyuan Wang**
University of Basel
Solved by: **Alessandro Prescimone**

Sample ID: **WFY375**

Crystal Data and Experimental

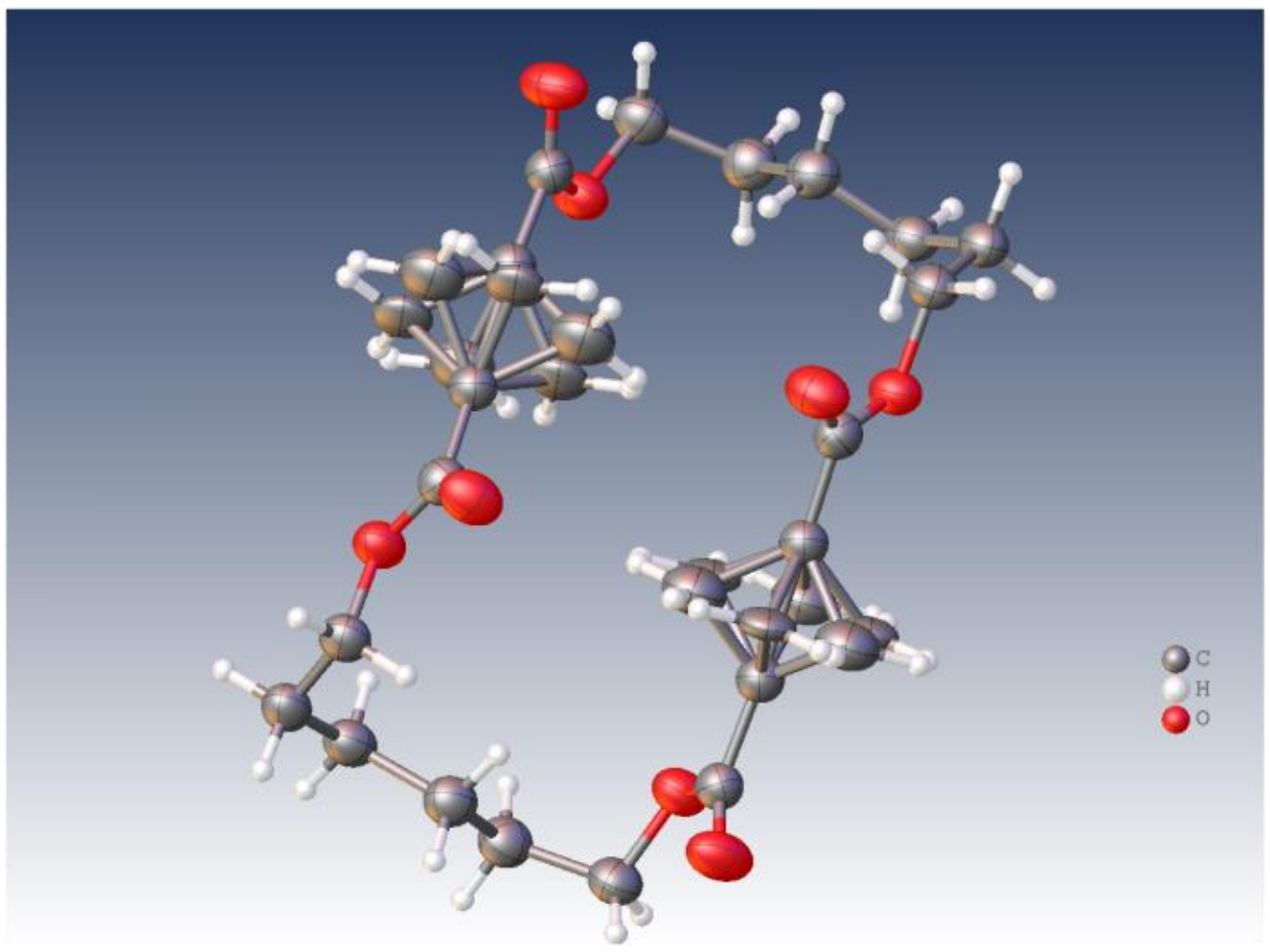

**Compound**	**WFY375_150K**
Formula	$C_{26}H_{36}O_8$
$D_{calc.}$/ g cm^{-3}	1.272
μ/mm^{-1}	0.770
Formula Weight	476.55
Colour	colourless
Shape	plate-shaped
Size/mm	0.30×0.18×0.05
T/K	150
Crystal System	monoclinic
Space Group	$C2/c$
a/Å	17.2246(9)
b/Å	5.8885(2)
c/Å	26.9270(14)
α/°	90
β/°	114.308(4)
γ/°	90
V/Å^3	2489.0(2)
Z	4
Z'	0.5
Wavelength/Å	1.54186
Radiation type	Cu K_α
Θ_{min}/°	3.602
Θ_{max}/°	74.601
Index range h	-18 ≥ h ≥ 21
Index range k	-7 ≥ k ≥ 4
Index range l	-33 ≥ l ≥ 33
Measured Refl's.	11492
Indep't Refl's	2509
Refl's I≥2σ(I)	2121
R_{int}	0.0229
Parameters	157
Restraints	27
Largest Peak/eÅ^3	0.450
Deepest Hole/eÅ^3	-0.563
GooF	1.034
R_1 (I≥2σ(I) / all)	0.0630 / 0.0722
wR_2 (I≥2σ(I) / all)	0.1654 / 0.1751

Experimental. Single colourless plate-shaped crystals of **WFY375_150K** were used as supplied. A suitable crystal with dimensions 0.30 × 0.18 × 0.05 mm was selected and mounted on a STOE STADIVARI Cu diffractometer. The crystal was kept at a steady T = 150 K during data collection. The structure was solved with the ShelXT 2018/2 (Sheldrick, 2018) solution program using dual methods and by using Olex2 1.5 (Dolomanov et al., 2009) as the graphical interface. The model was refined with ShelXL 2019/3 (Sheldrick, 2015) using full matrix least squares minimisation on $|F|^2$.

Crystal Data. $C_{26}H_{36}O_8$, M_r = 476.55, monoclinic, $C2/c$ (No. 15), a = 17.2246(9) Å, b = 5.8885(2) Å, c = 26.9270(14) Å, β = 114.308(4)°, $\alpha = \gamma$ = 90°, V = 2489.0(2) Å^3, T = 150 K, Z = 4, Z' = 0.5, μ(Cu K_α) = 0.770, 11492 reflections measured, 2509 unique (R_{int} = 0.0229) which were used in all calculations. The final wR_2 was 0.1751 (all data) and R_1 was 0.0630 (I≥2σ(I)).

Structure Quality Indicators

**Reflections:**	d min (CuKα) 2Θ=149.2° 0.80	I/σ(I) 69.9	Rint m=4.80 2.29%	Full 135.4° 98% to 149.2° 99.0
**Refinement:**	Shift 0.000	Max Peak 0.5	Min Peak -0.6	GooF 1.034

A colourless plate-shaped crystal with dimensions 0.30 × 0.18 × 0.05 mm was mounted. Data were collected using a STOE STADIVARI Cu diffractometer equipped with an Oxford Cryosystems low-temperature device operating at T = 150 K.

Data were measured using rotation method, ω scans with Cu K_α radiation. The diffraction pattern was indexed and the total number of runs and images was based on the strategy calculation from the program X-Area Pilatus3_SV 1.31.170.0 (STOE, 2020) . The maximum resolution achieved was Θ= 74.601° (0.80 Å).

The unit cell was refined using X-Area Pilatus3_SV 1.31.170.0 (STOE, 2020) on 13979 reflections, 122% of the observed reflections.

Data reduction, scaling and absorption corrections were performed using X-Area Pilatus3_SV 1.31.170.0 (STOE, 2020). The final completeness is 99.00 % out to 74.601° in Θ. A multi-scan absorption correction was performed using STOE. The absorption coefficient μ of this material is 0.770 mm^{-1} at this wavelength (λ = 1.54186Å) and the minimum and maximum transmissions are 0.000 and 0.003.

The structure was solved in the space group $C2/c$ (# 15) by ShelXT 2018/2 (Sheldrick, 2018) using dual methods. It was refined by full matrix least squares minimisation on $|F|^2$ using version 2019/3 of ShelXL 2019/3 (Sheldrick, 2015). All non-hydrogen atoms were refined anisotropically.

Hydrogen atom positions were calculated geometrically and refined using the riding model.

_exptl_absorpt_process_details: STOE X-Red32, absorption correction by Gaussian integration, analogous toP. Coppens in: F. R. Ahmed (Editor), "Crystallographic Computing", Munksgaard,Copenhagen (1970), 255 - 270. Afterwards scaling of reflection intensities was performed within STOE LANA. J. Koziskova, F. Hahn, J. Richter, J. Kozisek, Acta Chimica Slovaca, vol. 9, no. 2, 2016, pp. 136 - 140. Finally, a spherical absorption correction was done within STOE LANA.

The value of Z' is 0.5. This means that only half of the formula unit is present in the asymmetric unit, with the other half consisting of symmetry equivalent atoms. The moiety formula is C26 H36 O8.

Data Plots: Diffraction Data

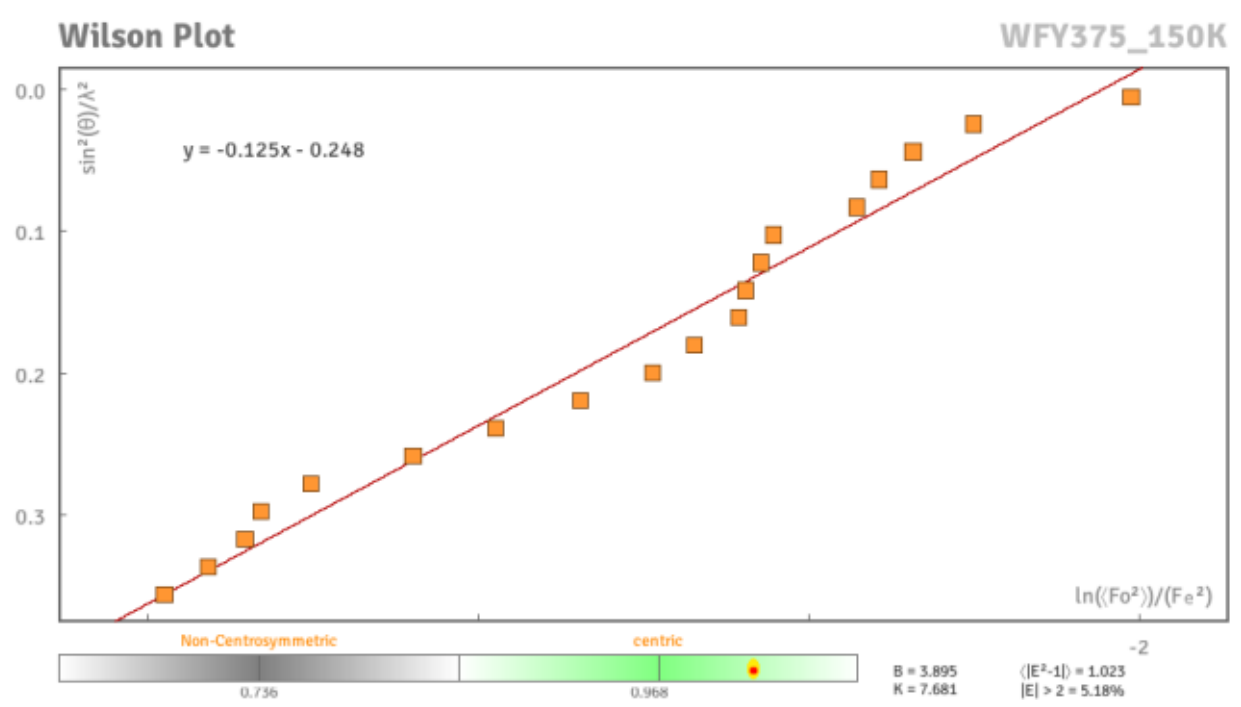

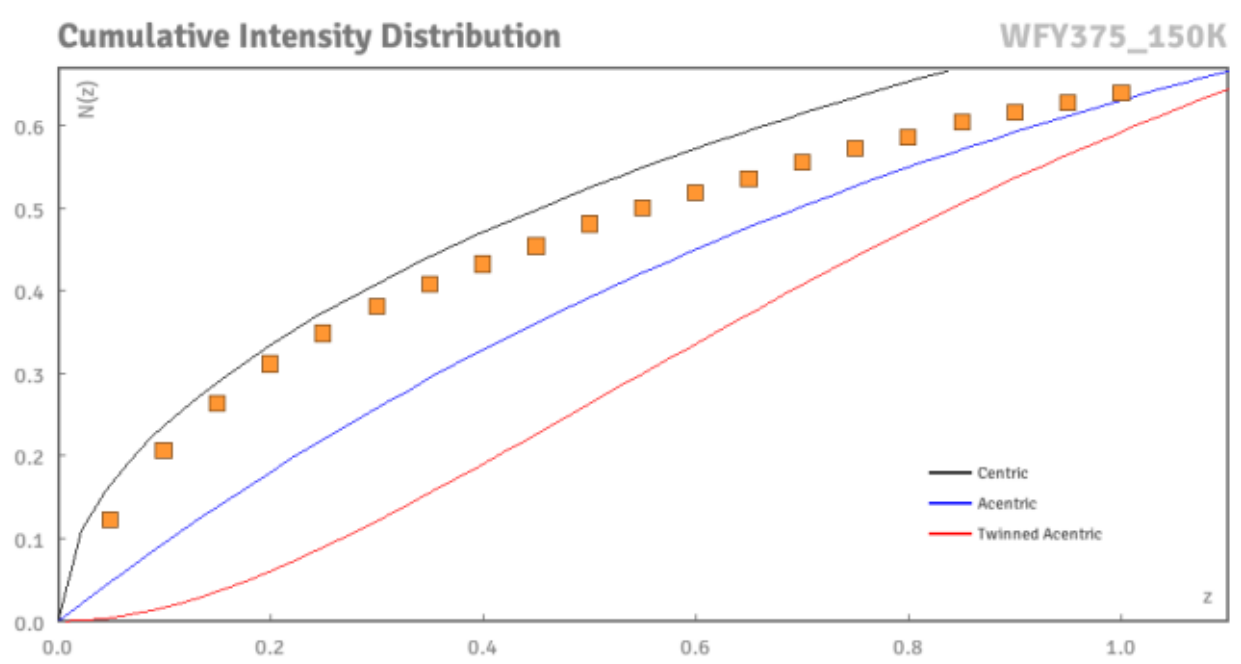

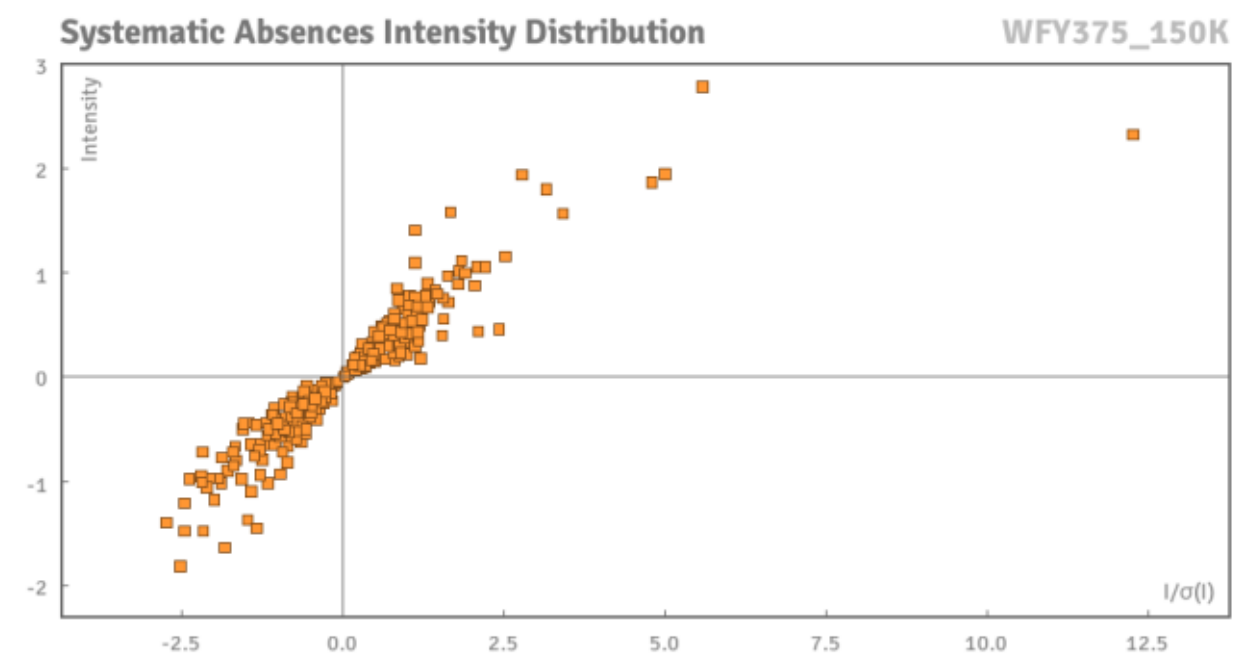

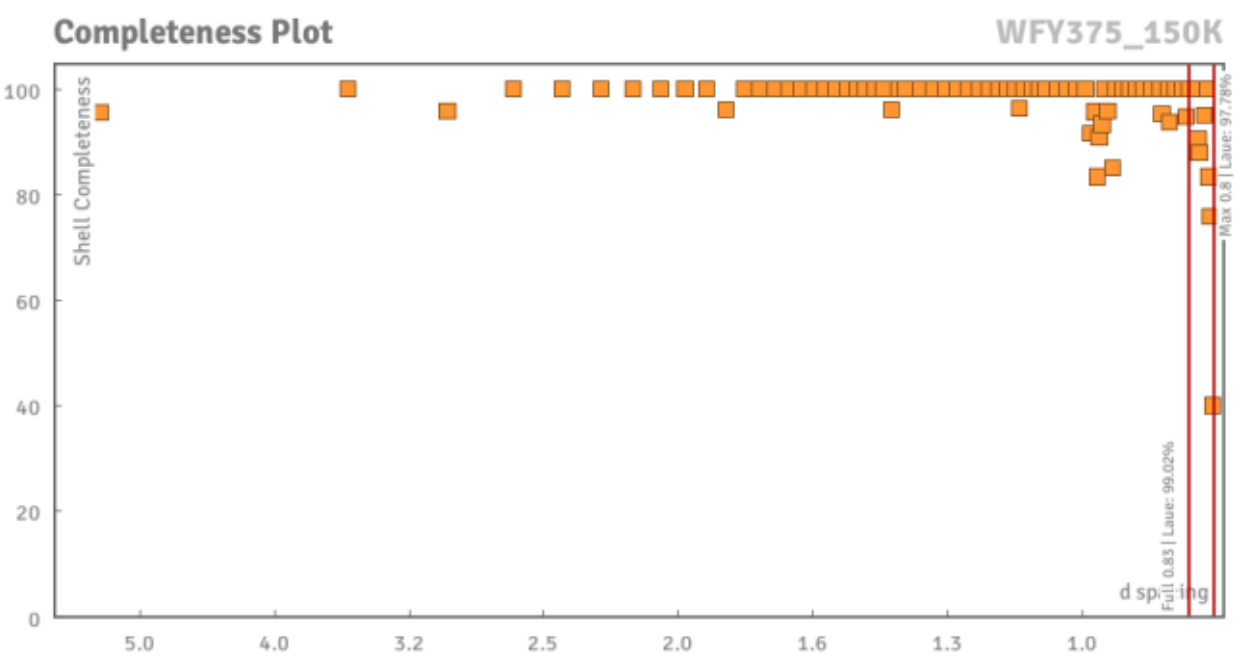

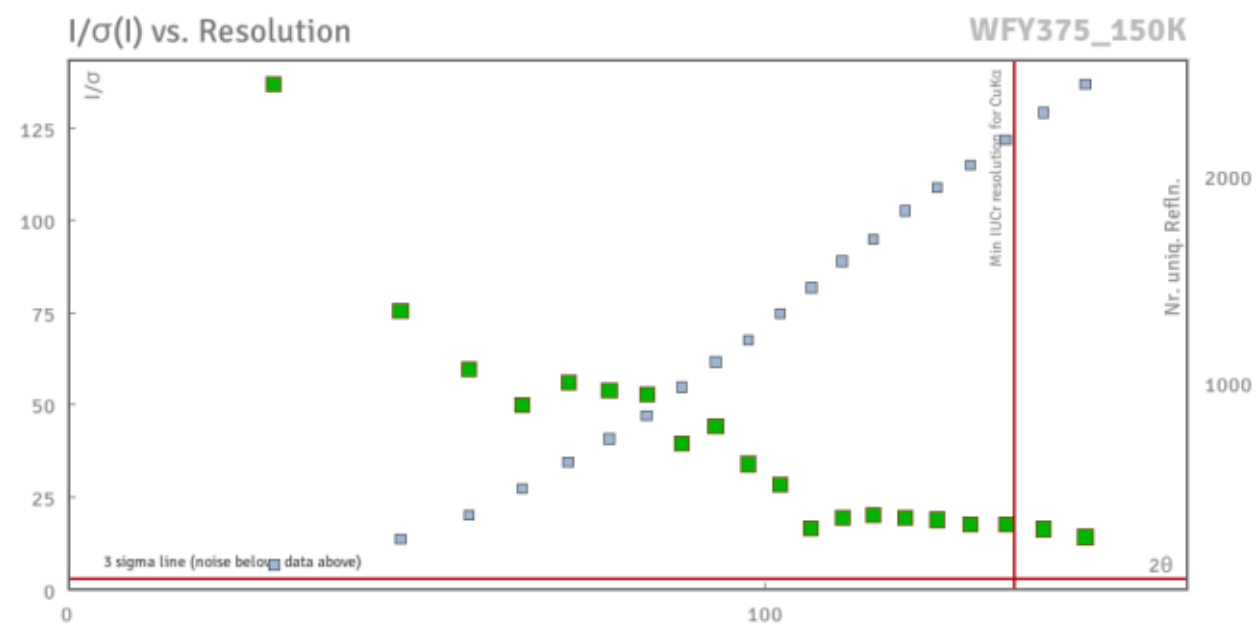

Data Plots: Refinement and Data

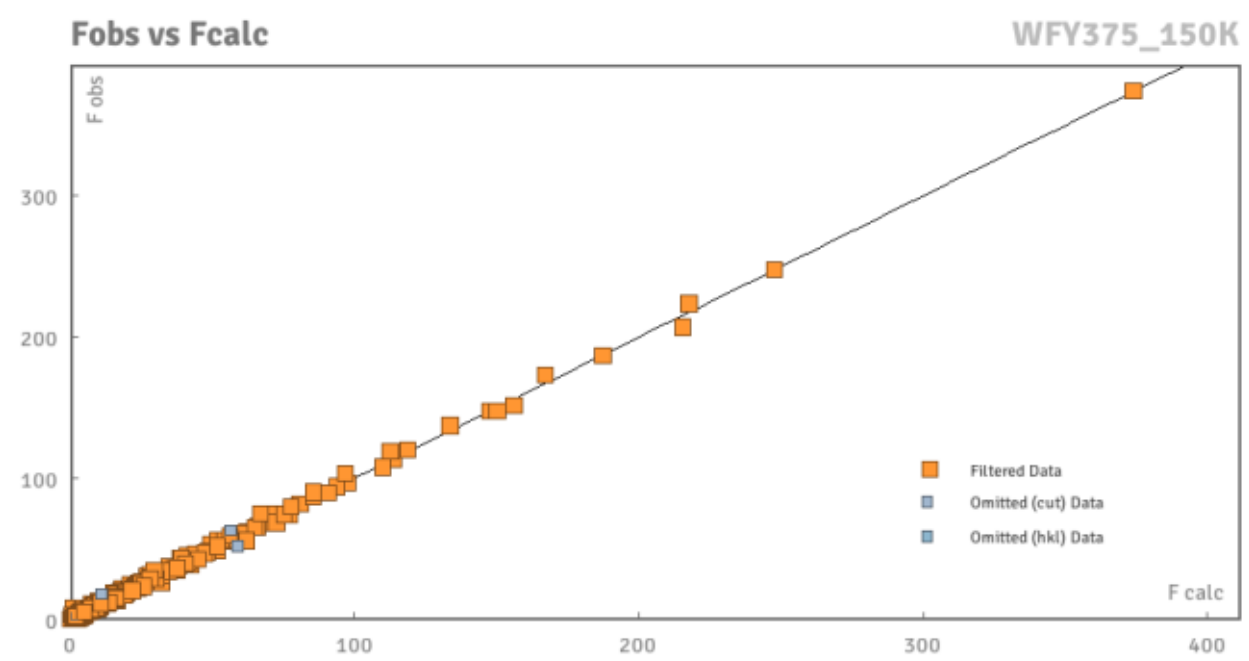

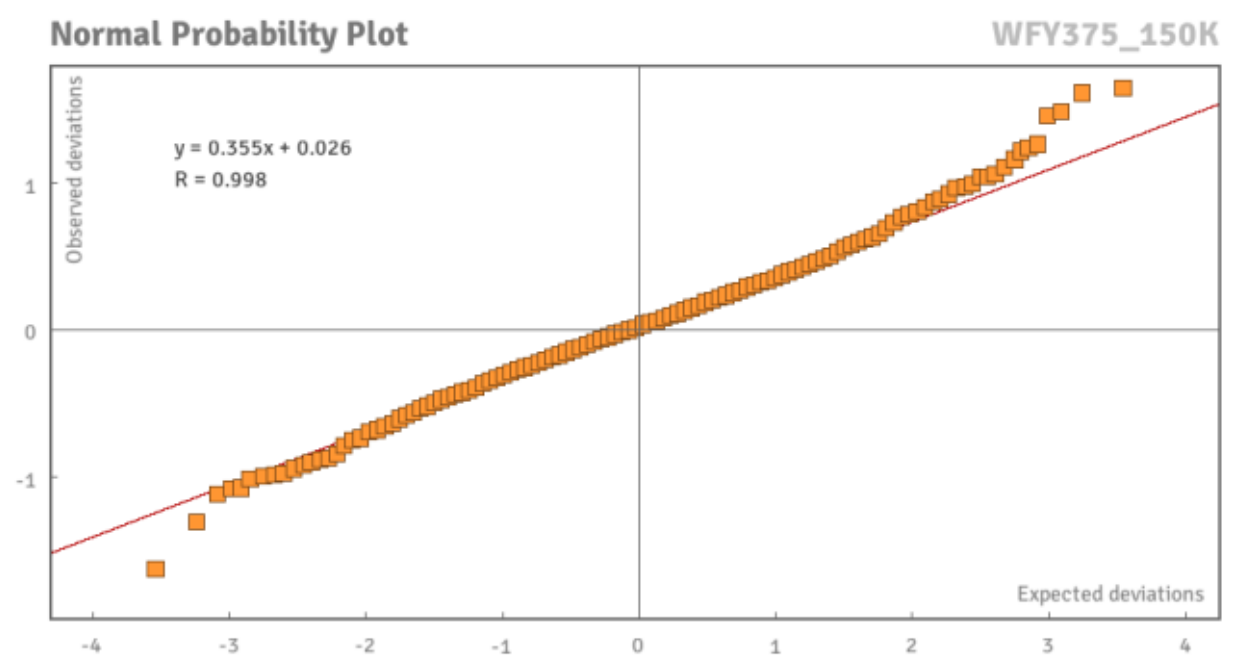

Reflection Statistics

Total reflections (after filtering)	12038	Unique reflections	2509
Completeness	0.978	Mean I/σ	39.59
hkl_{max} collected	(21, 4, 33)	hkl_{min} collected	(-18, -7, -33)
hkl_{max} used	(19, 7, 33)	hkl_{min} used	(-21, 0, 0)
Lim d_{max} collected	100.0	Lim d_{min} collected	0.77
d_{max} used	12.27	d_{min} used	0.8
Friedel pairs	337	Friedel pairs merged	1
Inconsistent equivalents	58	R_{int}	0.0229
R_{sigma}	0.0143	Intensity transformed	0
Omitted reflections	0	Omitted by user (OMIT hkl)	10
Multiplicity	(1509, 1240, 845, 570, 312, 179, 70, 15)	Maximum multiplicity	15
Removed systematic absences	546	Filtered off (Shel/OMIT)	0

Table 27: Fractional Atomic Coordinates (×10^4) and Equivalent Isotropic Displacement Parameters (Å^2×10^3) for **WFY375_150K**. U_{eq} is defined as 1/3 of the trace of the orthogonalised U_{ij}.

Atom	x	y	z	U_{eq}
O2	3870.6(10)	4119(3)	3305.3(6)	47.5(4)
O3	6952.2(11)	540(3)	3920.2(6)	50.2(4)
O1	4249.1(12)	7410(3)	3047.4(7)	60.0(5)

Atom	x	y	z	U_{eq}
O4	7633.3(12)	3766(3)	4304.3(7)	63.7(5)
C1	3936.5(14)	5556(4)	2938.8(9)	44.8(5)
C9	7216.3(13)	2662(4)	3910.7(9)	45.2(5)
C2	3572.1(14)	4579(4)	2377.3(9)	45.1(5)
C10	6891.9(13)	3488(4)	3337.0(8)	44.2(5)
C4	4185.5(15)	3064(4)	4234.6(9)	49.1(6)
C8	7031.2(15)	-424(5)	4437.4(9)	51.1(6)
C5	4707.1(15)	978(4)	4228.2(9)	48.9(5)
C3	4202.4(15)	4965(4)	3863.6(9)	48.2(5)
C6	5646.9(15)	1420(4)	4375.4(10)	50.9(6)
C7	6141.9(15)	-765(4)	4405.0(10)	51.9(6)
C11	6036(2)	4711(7)	3043.7(13)	69.7(6)
C13	6676(2)	2104(7)	2812.0(13)	69.7(6)
C12	7277(2)	5300(7)	3096.3(13)	69.7(6)
C14	6693(10)	6030(20)	3148(6)	55(2)
C15	6005(8)	2910(30)	2872(5)	55(2)
C16	7278(9)	3240(30)	2917(5)	55(2)

Table 28: Anisotropic Displacement Parameters (×10[4]) for **WFY375_150K.** The anisotropic displacement factor exponent takes the form: $-2\pi^2[h^2a^{*2} \times U_{11} + ... + 2hka^* \times b^* \times U_{12}]$

Atom	U_{11}	U_{22}	U_{33}	U_{23}	U_{13}	U_{12}
O2	48.7(8)	54.2(10)	37.1(8)	-3.2(6)	15.2(7)	-6.1(7)
O3	57.3(9)	54.5(10)	37.5(8)	1.5(7)	18.1(7)	-4.1(8)
O1	75.4(12)	55.5(11)	46.8(10)	-5.4(8)	23.0(9)	-11.9(9)
O4	73.4(12)	68.3(12)	39.7(9)	-1.7(8)	13.4(8)	-17.9(9)
C1	40.4(11)	50.0(13)	42.7(11)	-0.4(9)	15.7(9)	1.4(9)
C9	40.1(11)	54.7(13)	40.1(11)	-0.1(9)	15.8(9)	0.4(9)
C2	41.4(11)	53.1(13)	39.0(11)	-0.2(9)	14.8(9)	0.2(10)
C10	40.7(10)	51.8(13)	38.7(11)	0.5(9)	14.9(9)	-0.3(9)
C4	45.4(11)	62.4(15)	38.6(11)	-5.1(10)	16.5(9)	-7.4(10)
C8	54.4(13)	56.9(14)	40.1(11)	7.4(10)	17.6(10)	1.5(11)
C5	51.4(12)	54.4(13)	38.0(11)	-0.3(9)	15.5(9)	-7.3(10)
C3	48.1(12)	56.6(14)	38.1(11)	-5.8(9)	16.1(9)	-3.1(10)
C6	50.9(13)	54.9(14)	44.8(12)	-0.6(10)	17.5(10)	-4.7(10)
C7	54.1(13)	53.3(14)	47.2(13)	4.0(10)	19.9(10)	-3.3(11)
C11	72.2(12)	85.7(15)	45.4(10)	11.5(9)	18.4(9)	4.3(11)
C13	72.2(12)	85.7(15)	45.4(10)	11.5(9)	18.4(9)	4.3(11)
C12	72.2(12)	85.7(15)	45.4(10)	11.5(9)	18.4(9)	4.3(11)
C14	56(5)	75(7)	24(4)	18(4)	6(4)	10(5)
C15	56(5)	75(7)	24(4)	18(4)	6(4)	10(5)
C16	56(5)	75(7)	24(4)	18(4)	6(4)	10(5)

Table 29: Bond Lengths in Å for **WFY375_150K.**

Atom	Atom	Length/Å
O2	C1	1.340(3)
O2	C3	1.459(3)
O3	C9	1.334(3)
O3	C8	1.458(3)
O1	C1	1.199(3)
O4	C9	1.198(3)
C1	C2	1.493(3)
C9	C10	1.491(3)
C2	C10[1]	1.867(3)
C2	C11[1]	1.541(4)
C2	C13[1]	1.545(4)
C2	C12[1]	1.553(4)
C2	C14[1]	1.553(15)
C2	C15[1]	1.534(15)
C2	C16[1]	1.560(14)
C10	C11	1.535(4)
C10	C13	1.539(4)
C10	C12	1.534(4)
C10	C14	1.574(12)
C10	C15	1.564(11)

Atom	Atom	Length/Å
C10	C16	1.536(11)
C4	C5	1.526(3)
C4	C3	1.509(3)
C8	C7	1.511(3)
C5	C6	1.522(3)
C6	C7	1.528(4)

––––
[1]1-x,+y,1/2-z

Table 30: Bond Angles in ° for **WFY375_150K**.

Atom	Atom	Atom	Angle/°
C1	O2	C3	115.28(18)
C9	O3	C8	119.01(18)
O2	C1	C2	112.0(2)
O1	C1	O2	124.1(2)
O1	C1	C2	123.9(2)
O3	C9	C10	109.89(19)
O4	C9	O3	125.2(2)
O4	C9	C10	124.9(2)
C1	C2	C10[1]	177.5(2)
C1	C2	C11[1]	126.6(2)
C1	C2	C13[1]	129.9(2)
C1	C2	C12[1]	126.0(2)
C1	C2	C14[1]	123.6(5)
C1	C2	C15[1]	127.8(4)
C1	C2	C16[1]	128.6(4)
C11[1]	C2	C10[1]	52.47(15)
C11[1]	C2	C13[1]	87.2(2)
C11[1]	C2	C12[1]	86.1(2)
C11[1]	C2	C14[1]	49.9(6)
C11[1]	C2	C16[1]	104.8(4)
C13[1]	C2	C10[1]	52.60(15)
C13[1]	C2	C12[1]	87.0(2)
C13[1]	C2	C14[1]	106.3(4)
C13[1]	C2	C16[1]	44.2(7)
C12[1]	C2	C10[1]	52.33(15)
C12[1]	C2	C14[1]	43.7(6)
C12[1]	C2	C16[1]	49.5(7)
C14[1]	C2	C16[1]	87.3(7)
C15[1]	C2	C16[1]	88.3(8)
C9	C10	C2[1]	176.64(18)
C9	C10	C11	123.9(2)
C9	C10	C13	128.6(2)
C9	C10	C12	128.4(2)
C9	C10	C14	125.7(6)
C9	C10	C15	125.8(6)
C9	C10	C16	129.8(5)
C11	C10	C2[1]	52.79(16)
C11	C10	C13	87.6(2)
C13	C10	C2[1]	52.90(16)
C12	C10	C2[1]	53.24(15)
C12	C10	C11	87.0(2)
C12	C10	C13	87.9(2)
C15	C10	C14	86.5(8)
C16	C10	C14	87.4(8)
C16	C10	C15	88.1(8)
C3	C4	C5	114.70(19)
O3	C8	C7	107.63(19)
C6	C5	C4	115.3(2)
O2	C3	C4	108.81(19)
C5	C6	C7	112.4(2)
C8	C7	C6	114.9(2)
C10	C11	C2[1]	74.7(2)
C10	C13	C2[1]	74.5(2)
C10	C12	C2[1]	74.4(2)
C2[1]	C14	C10	73.3(6)
C2[1]	C15	C10	74.1(6)
C10	C16	C2[1]	74.1(6)

––––
[1]1-x,+y,1/2-z

Table 31: Torsion Angles in ° for **WFY375_150K**.

Atom	Atom	Atom	Atom	Angle/°
O2	C1	C2	C11[1]	-137.1(3)
O2	C1	C2	C13[1]	-14.4(4)
O2	C1	C2	C12[1]	107.1(3)
O2	C1	C2	C14[1]	160.9(8)
O2	C1	C2	C15[1]	-81.1(9)
O2	C1	C2	C16[1]	43.7(9)
O3	C9	C10	C11	89.8(3)
O3	C9	C10	C13	-28.4(3)
O3	C9	C10	C12	-153.4(3)
O3	C9	C10	C14	151.2(8)
O3	C9	C10	C15	36.1(9)
O3	C9	C10	C16	-87.1(10)
O3	C8	C7	C6	-67.1(3)
O1	C1	C2	C11[1]	43.0(4)
O1	C1	C2	C13[1]	165.7(3)
O1	C1	C2	C12[1]	-72.8(4)

Atom	Atom	Atom	Atom	Angle/°
O1	C1	C2	C14[1]	-19.0(8)
O1	C1	C2	C15[1]	99.0(9)
O1	C1	C2	C16[1]	-136.2(9)
O4	C9	C10	C11	-88.4(3)
O4	C9	C10	C13	153.5(3)
O4	C9	C10	C12	28.4(4)
O4	C9	C10	C14	-27.0(9)
O4	C9	C10	C15	-142.1(9)
O4	C9	C10	C16	94.7(10)
C1	O2	C3	C4	-172.86(18)
C9	O3	C8	C7	112.8(2)
C9	C10	C11	C2[1]	-179.9(2)
C9	C10	C13	C2[1]	176.2(2)
C9	C10	C12	C2[1]	-176.3(2)
C9	C10	C14	C2[1]	-176.2(2)
C9	C10	C15	C2[1]	176.7(2)
C9	C10	C16	C2[1]	179.6(3)
C4	C5	C6	C7	175.42(19)
C8	O3	C9	O4	11.7(3)
C8	O3	C9	C10	-166.46(18)
C5	C4	C3	O2	59.3(2)
C5	C6	C7	C8	168.3(2)
C3	O2	C1	O1	0.7(3)
C3	O2	C1	C2	-179.16(18)
C3	C4	C5	C6	57.5(3)
C11	C10	C13	C2[1]	43.3(2)
C11	C10	C12	C2[1]	-44.3(2)
C13	C10	C11	C2[1]	-43.4(2)
C13	C10	C12	C2[1]	43.5(2)
C12	C10	C11	C2[1]	44.6(2)
C12	C10	C13	C2[1]	-43.7(2)
C14	C10	C15	C2[1]	44.1(8)
C14	C10	C16	C2[1]	-44.1(8)
C15	C10	C14	C2[1]	-43.6(7)
C15	C10	C16	C2[1]	42.5(8)
C16	C10	C14	C2[1]	44.6(7)
C16	C10	C15	C2[1]	-43.4(8)

––––

[1]1-x,+y,1/2-z

Table 32: Hydrogen Fractional Atomic Coordinates (×10[4]) and Equivalent Isotropic Displacement Parameters (Å^2×10^3) for **WFY375_150K**. U_{eq} is defined as 1/3 of the trace of the orthogonalised U_{ij}.

Atom	x	y	z	U_{eq}
H4A	3586.65	2591.23	4128.2	59
H4B	4401.49	3651.33	4612.06	59
H8A	7355.67	618.99	4740.55	61
H8B	7336.36	-1893.63	4503.09	61
H5A	4661.02	-154.73	4486.39	59
H5B	4451.64	297.19	3859.5	59
H3A	3849.1	6248.6	3888.91	58
H3B	4793.41	5515.66	3974.49	58
H6A	5899.59	2201.11	4732.28	61
H6B	5701.4	2441.81	4099.46	61
H7A	6181.13	-1638.63	4728.64	62
H7B	5814.95	-1694.73	4079	62
H11A	5995.14	6248.18	3179.89	84
H11B	5520.05	3781.2	2963.93	84
H13A	6198.81	1014.24	2718.03	84
H13B	7167.32	1476.79	2755.93	84

Atom	x	y	z	U_{eq}
H12A	7805.28	4864.23	3058.25	84
H12B	7296.7	6863.75	3235.92	84
H14A	6223.94	6761.45	3211.93	66
H14B	7192.28	7037.79	3233.42	66
H15A	5932.26	1333.94	2730.12	66
H15B	5500.6	3481.4	2920.61	66
H16A	7292.21	1687.33	2782.29	66
H16B	7808.46	4107.23	2993.98	66

Table 33: Atomic Occupancies for all atoms that are not fully occupied in **WFY375_150K**.

Atom	Occupancy
C11	0.85
H11A	0.85
H11B	0.85
C13	0.85
H13A	0.85
H13B	0.85
C12	0.85
H12A	0.85
H12B	0.85
C14	0.15
H14A	0.15
H14B	0.15
C15	0.15
H15A	0.15
H15B	0.15
C16	0.15
H16A	0.15
H16B	0.15

Citations

O.V. Dolomanov and L.J. Bourhis and R.J. Gildea and J.A.K. Howard and H. Puschmann, Olex2: A complete structure solution, refinement and analysis program, *J. Appl. Cryst.*, (2009), **42**, 339-341.

STOE & Cie GmbH, X-Area, software package for collecting single-crystal or multi-domain crystal data on STOE area-detector diffractometers, for image processing, for the correction and scaling of reflection intensities and for outlier rejection, version 1.90, Darmstadt 2020

Sheldrick, G.M., Crystal structure refinement with ShelXL, *Acta Cryst.*, (2015), **C71**, 3-8.

Sheldrick, G.M., ShelXT-Integrated space-group and crystal-structure determination, *Acta Cryst.*, (2015), **A71**, 3-8.

X-Area Integrate 1.78.3.0

X-Area Pilatus3_SV 1.31.170.0 (STOE, 2020)

X-Area Recipe 1.36.0.0

Appendix B: NMR spectra of products

^{1}H NMR (500 MHz, $CDCl_3$, 298K) of **3a**

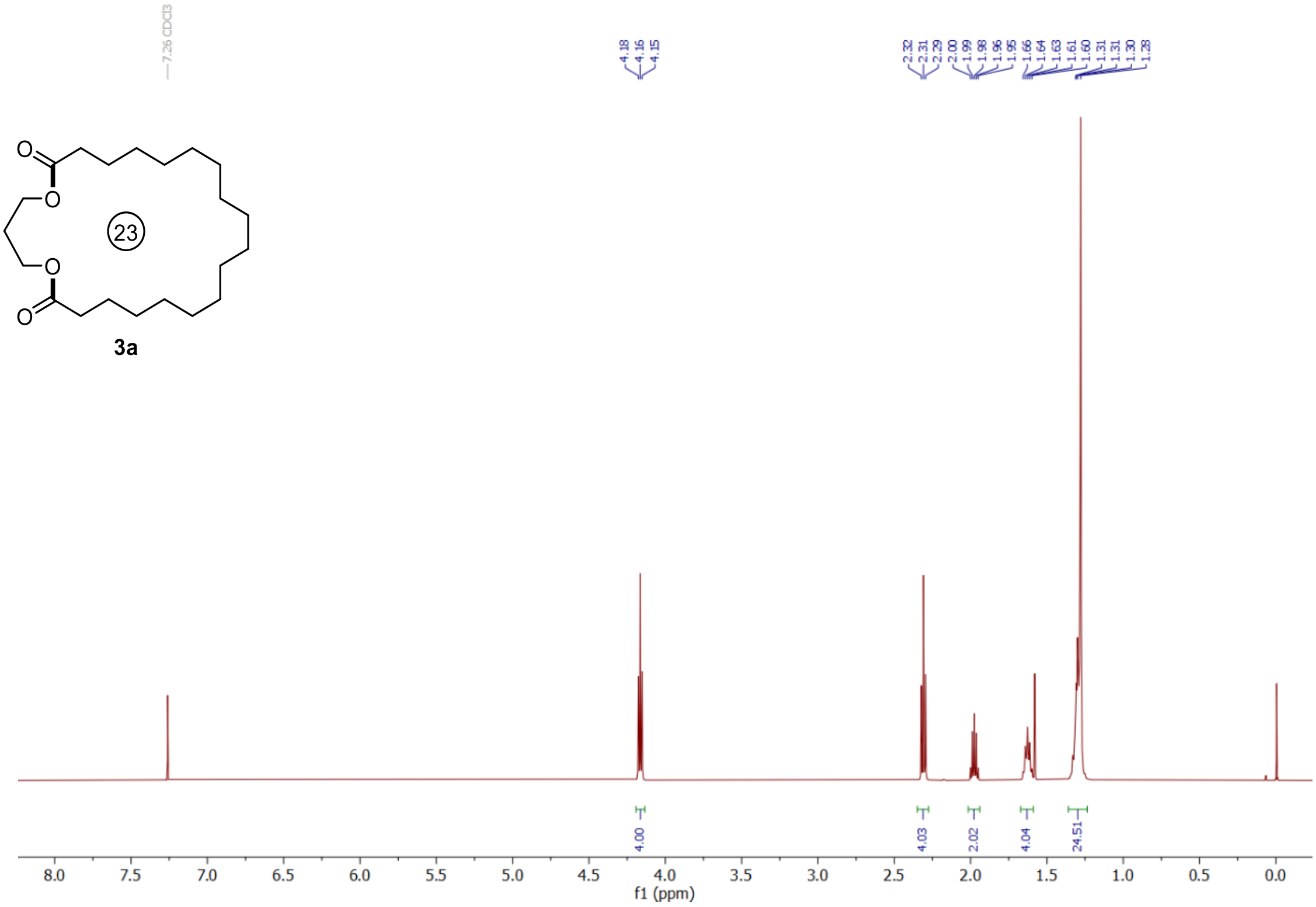

$^{13}C\{^{1}H\}$ NMR (126 MHz, $CDCl_3$, 298K) of **3a**

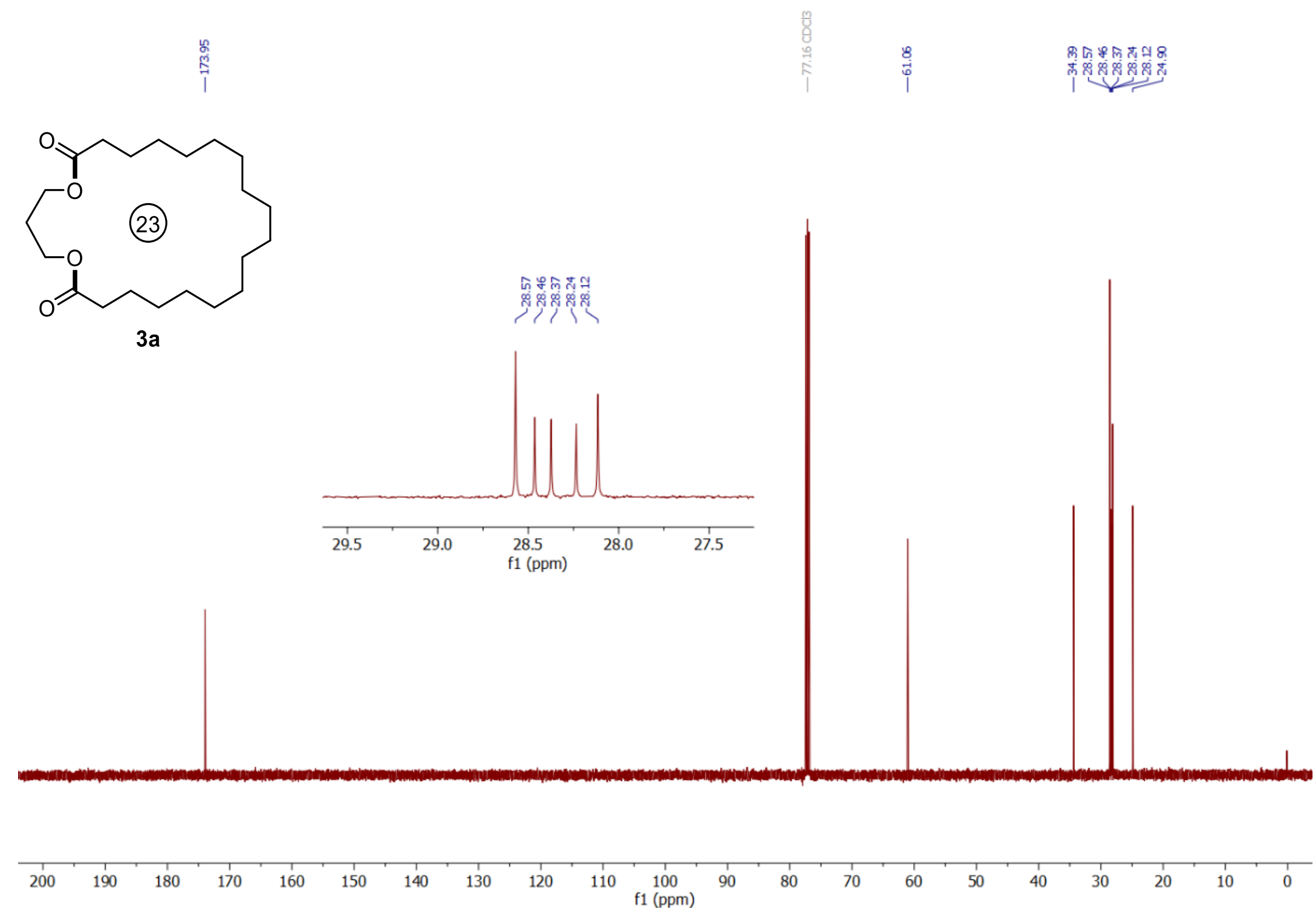

^{1}H NMR (500 MHz, $CDCl_3$, 298K) of **3b**

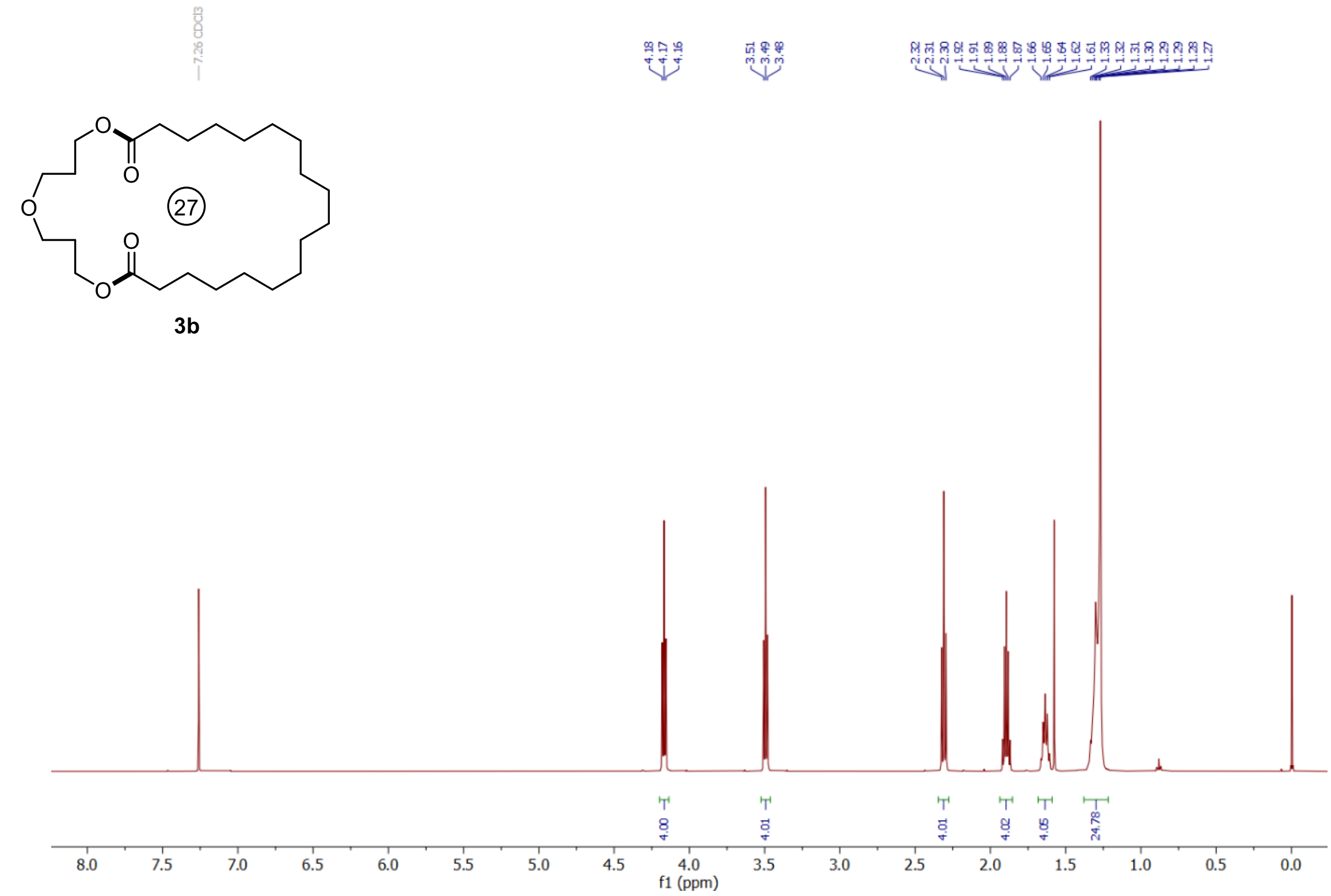

$^{13}C\{^{1}H\}$ NMR (126 MHz, $CDCl_3$, 298K) of **3b**

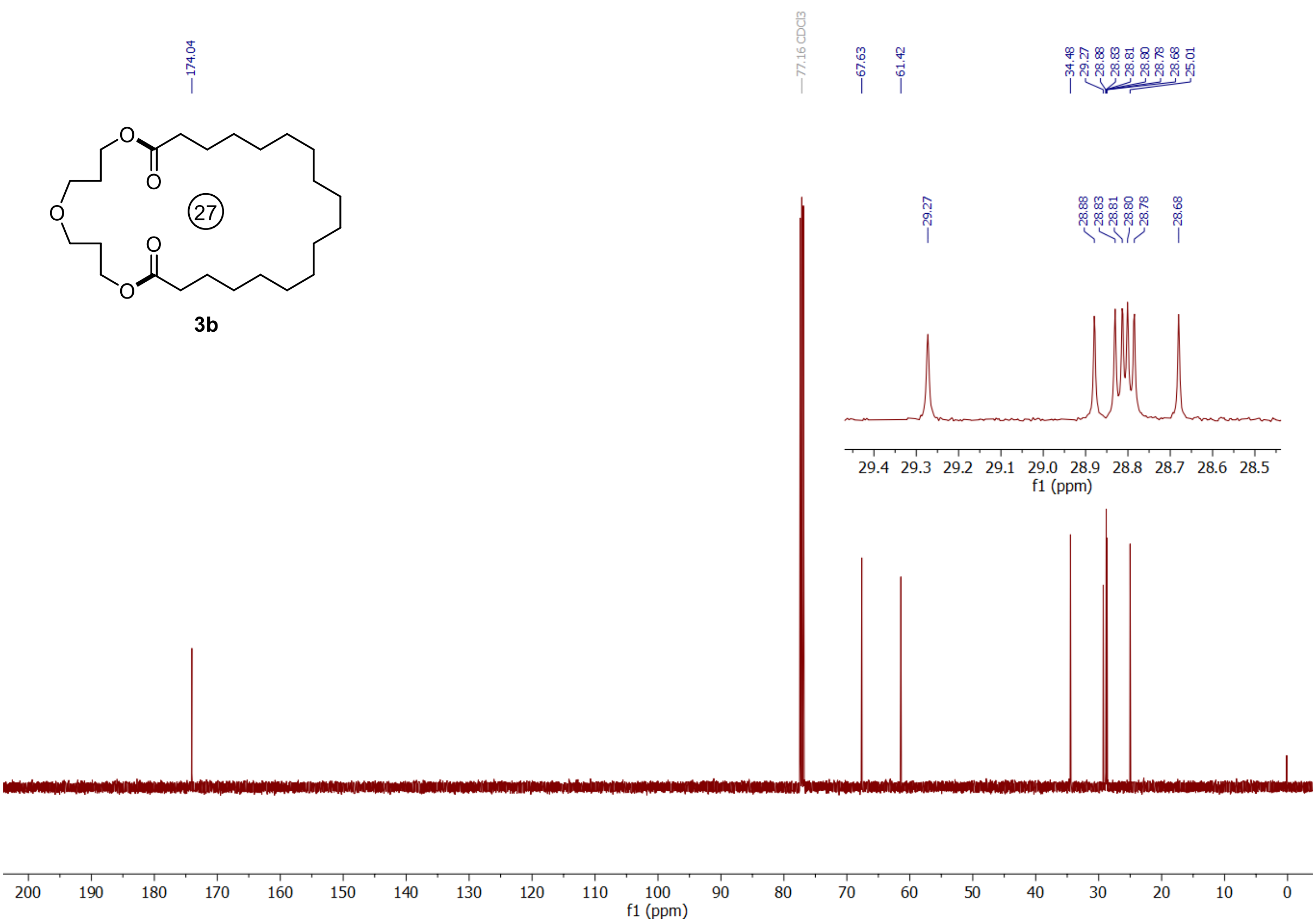

^{1}H NMR (500 MHz, $CDCl_3$, 298K) of **3e**

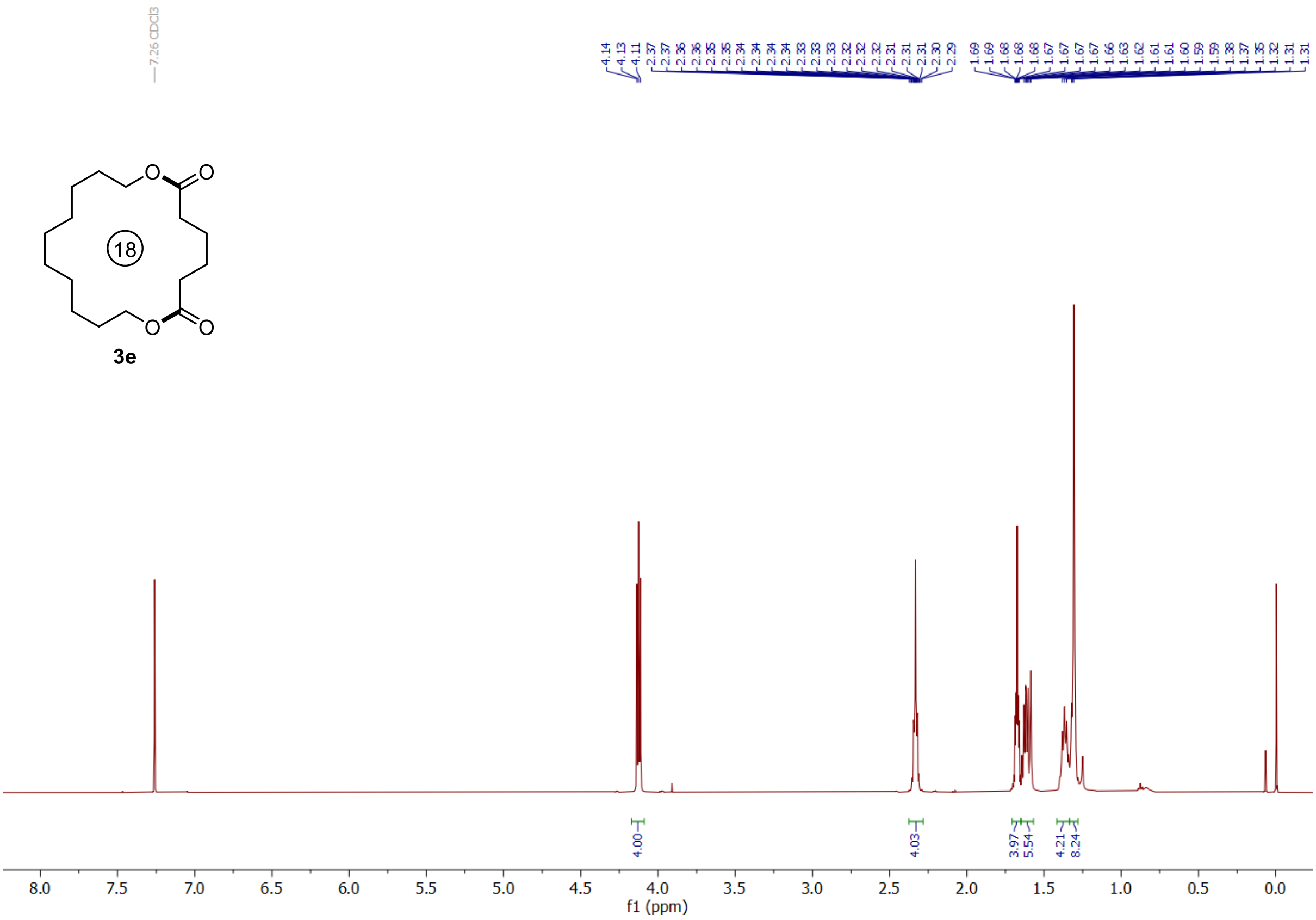

$^{13}C\{^{1}H\}$ NMR (126 MHz, $CDCl_3$, 298K) of **3e**

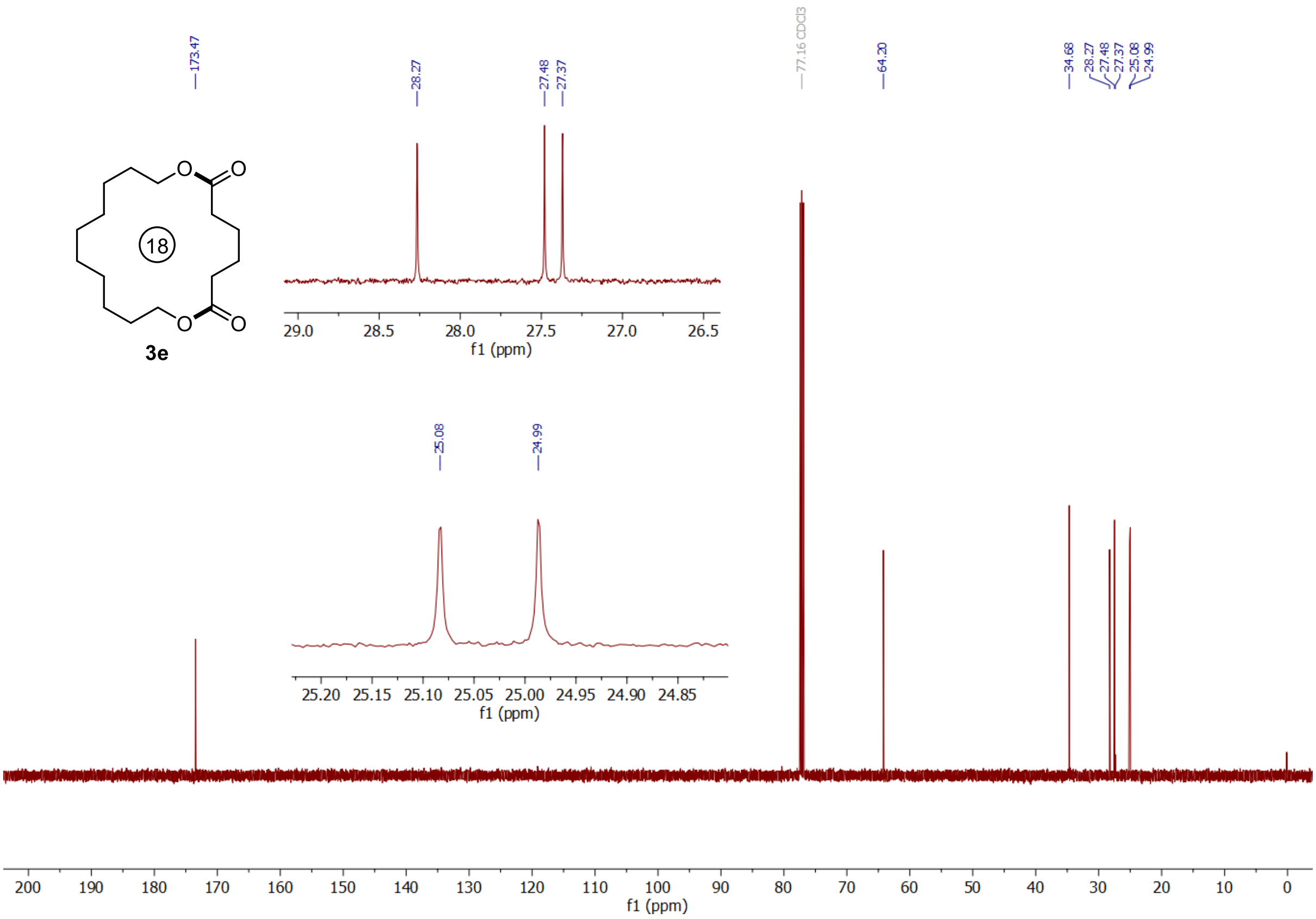

^{1}H NMR (500 MHz, $CDCl_3$, 298K) of **3f**

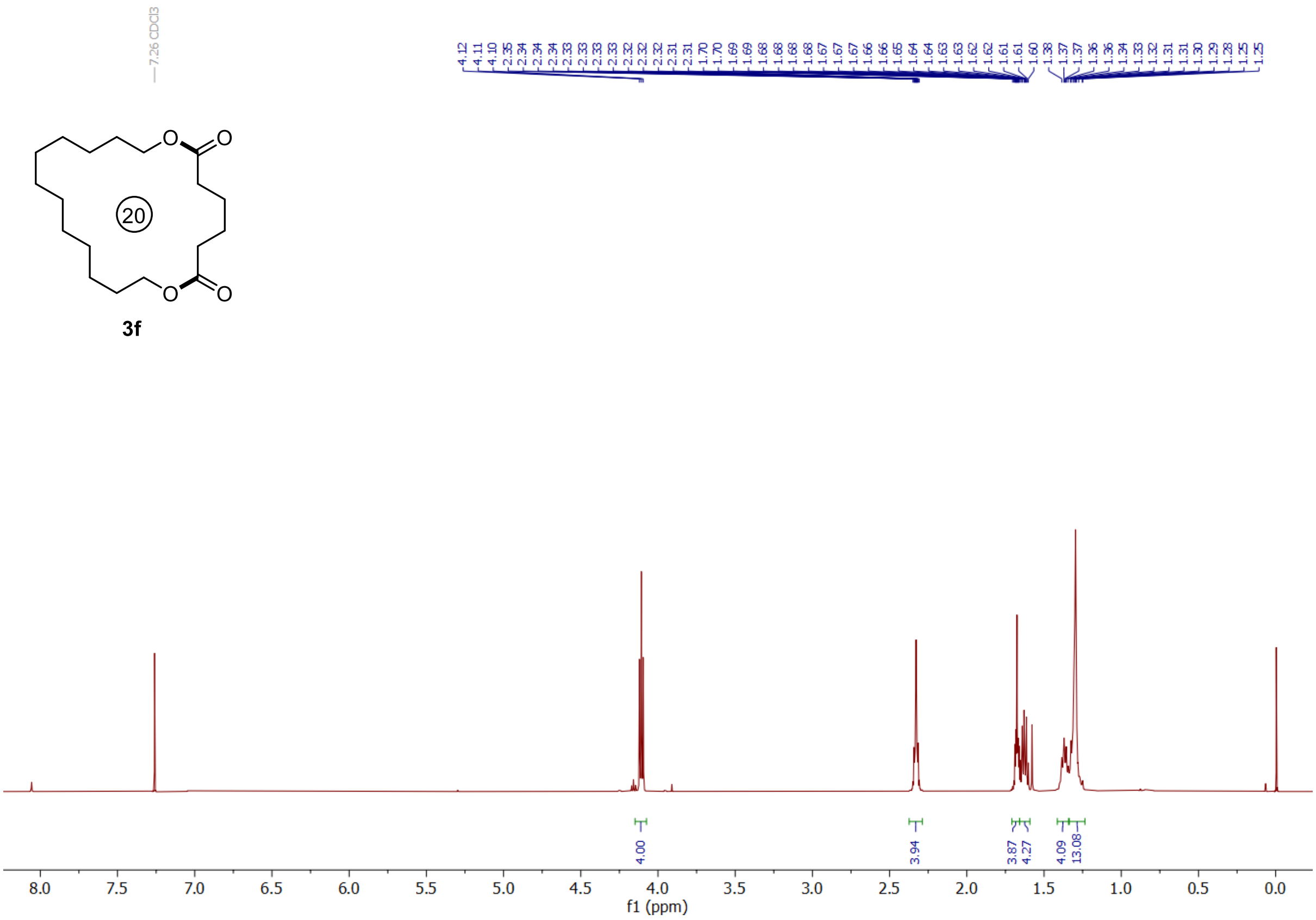

$^{13}C\{^1H\}$ NMR (126 MHz, $CDCl_3$, 298K) of **3f**

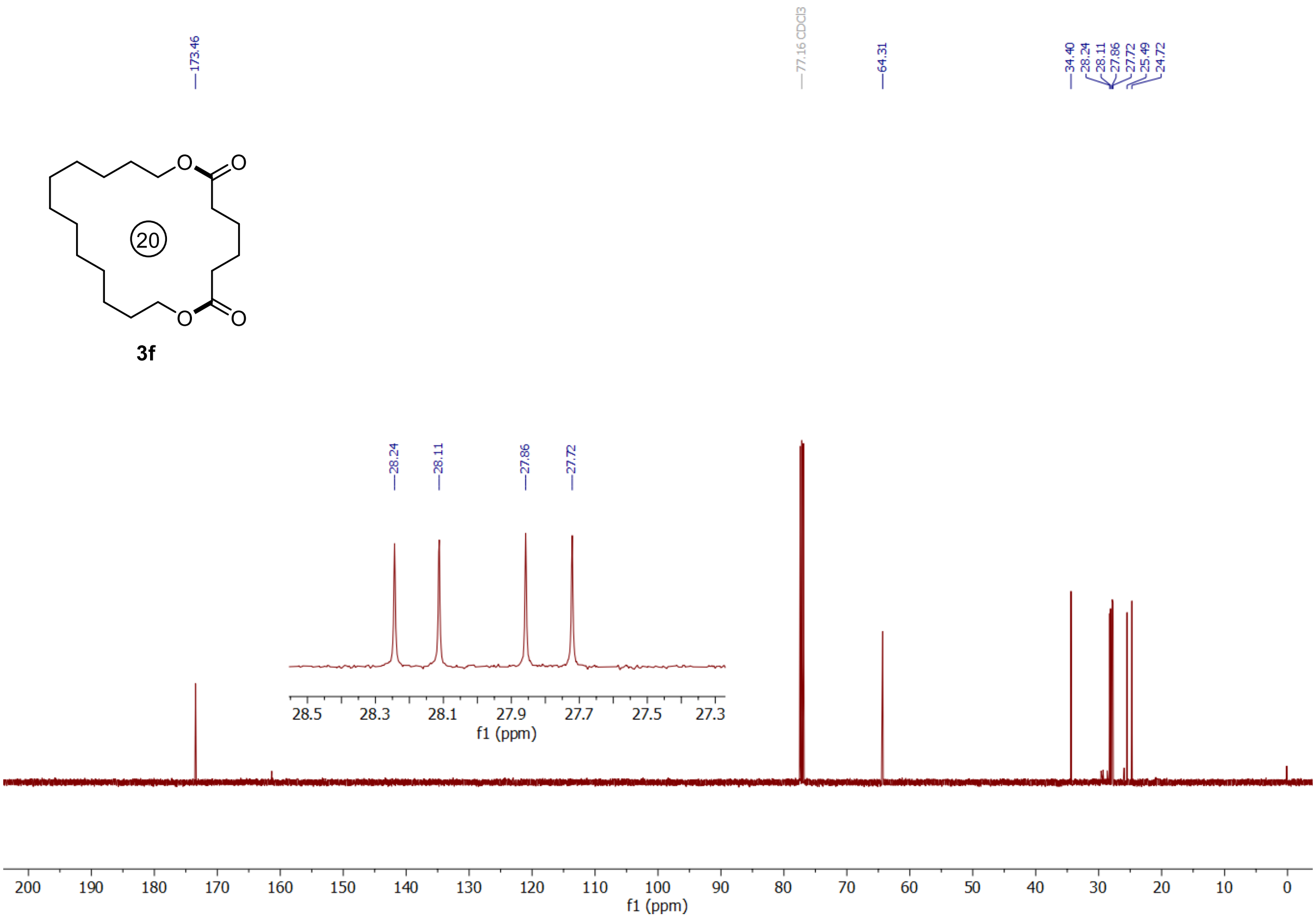

^{1}H NMR (500 MHz, $CDCl_3$, 298K) of **3g**

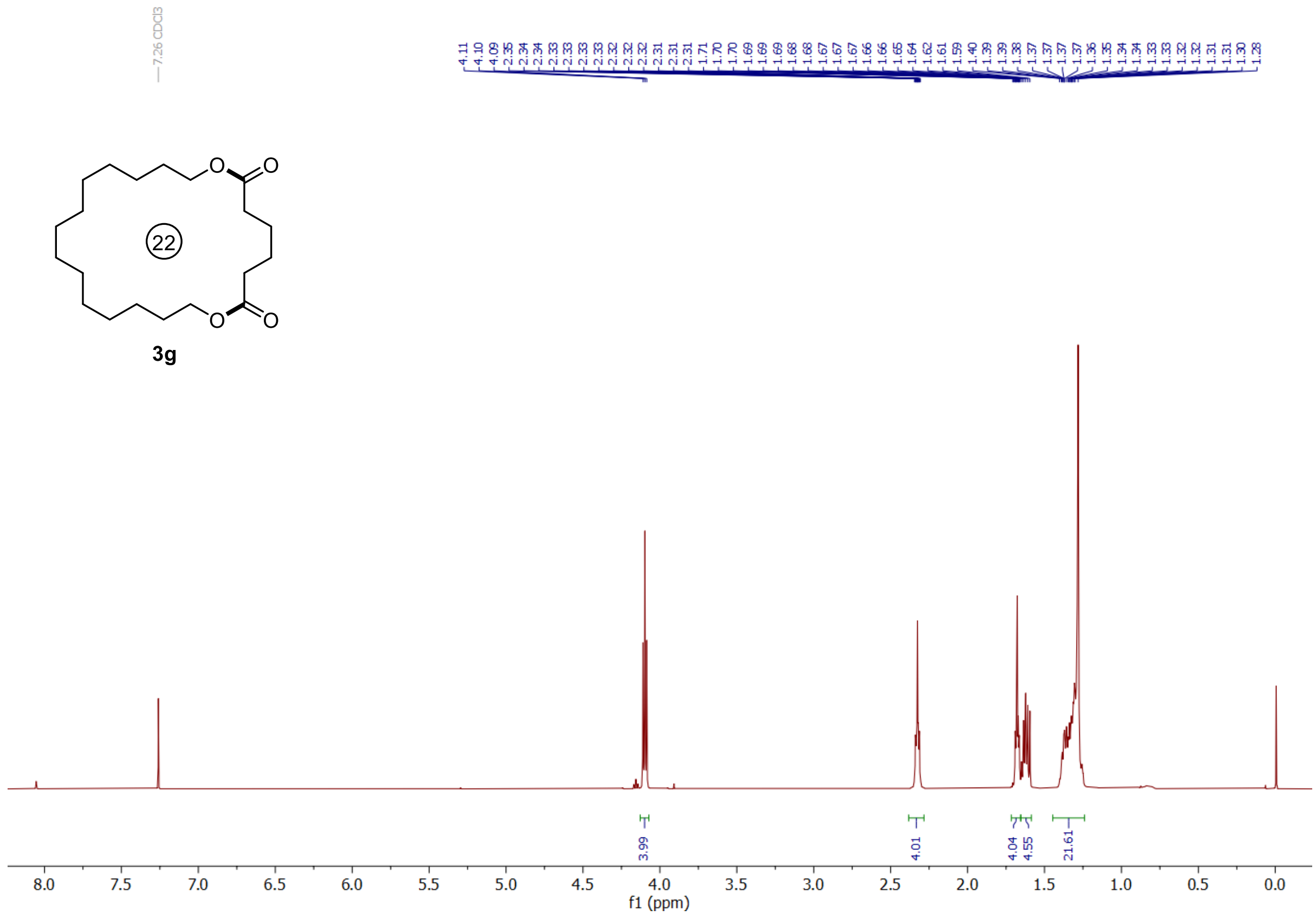

$^{13}C\{^{1}H\}$ NMR (126 MHz, $CDCl_3$, 298K) of **3g**

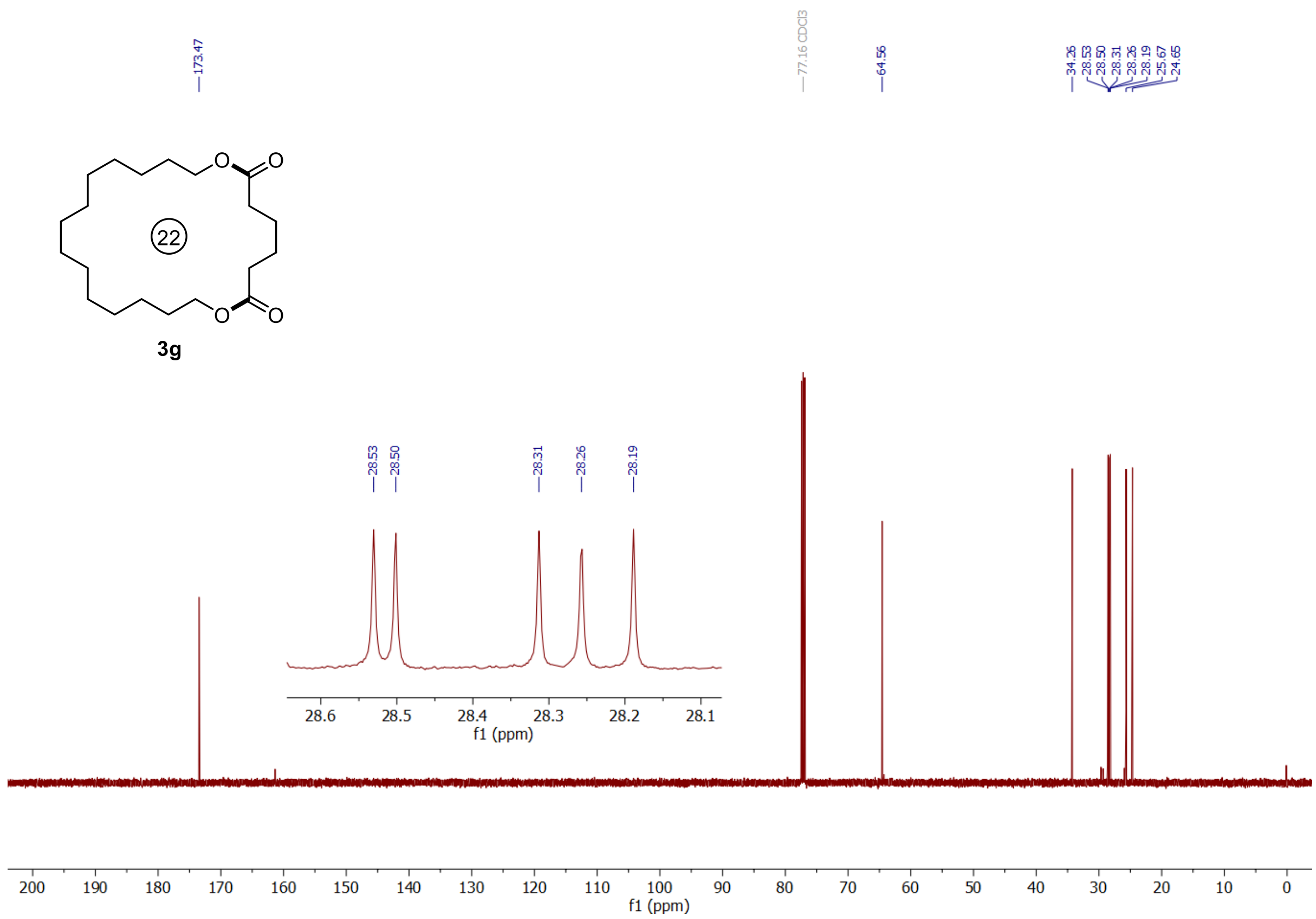

^{1}H NMR (500 MHz, $CDCl_3$, 298K) of **3h**

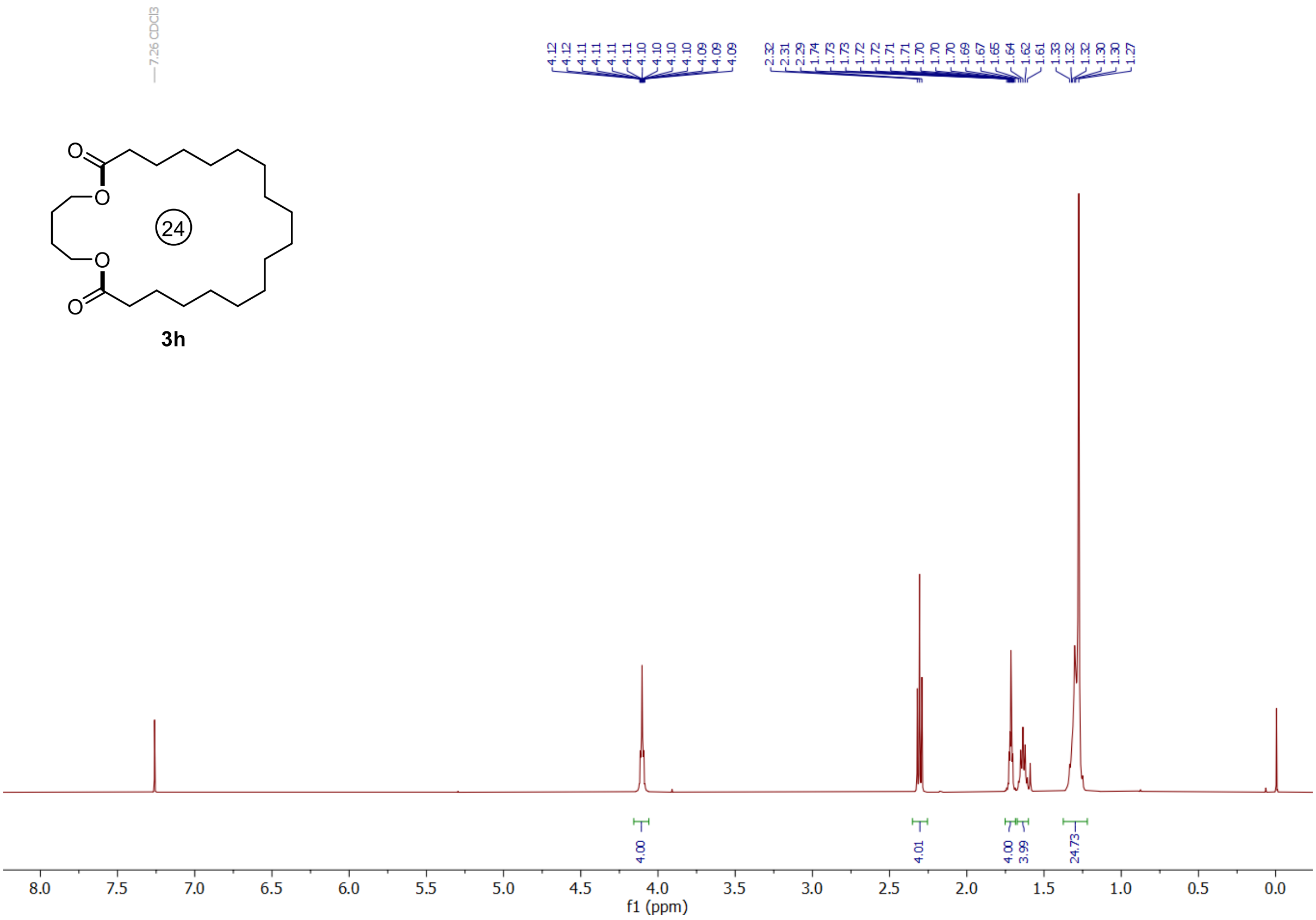

$^{13}C\{^1H\}$ NMR (126 MHz, $CDCl_3$, 298K) of **3h**

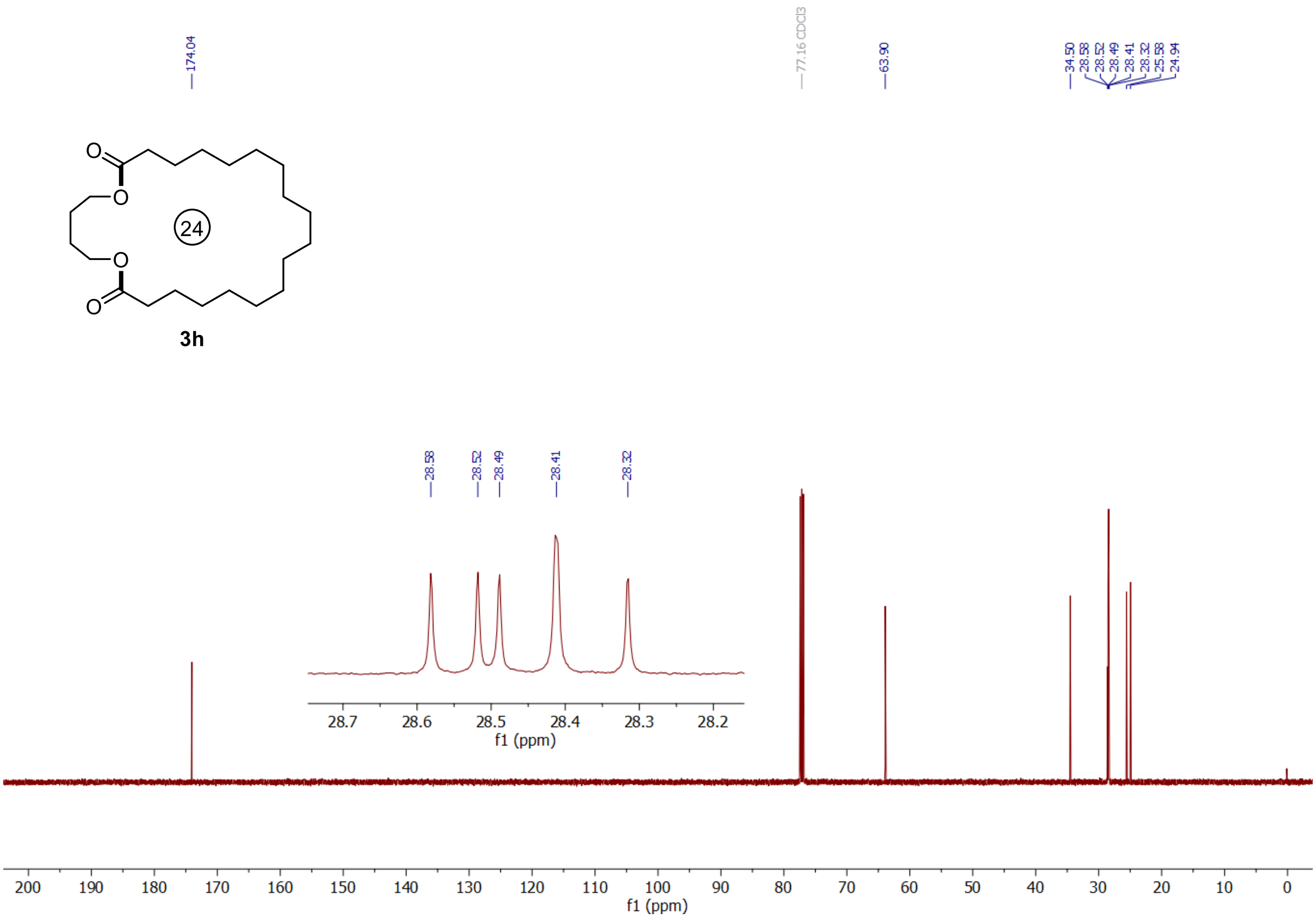

^{1}H NMR (500 MHz, $CDCl_3$, 298K) of **3i**

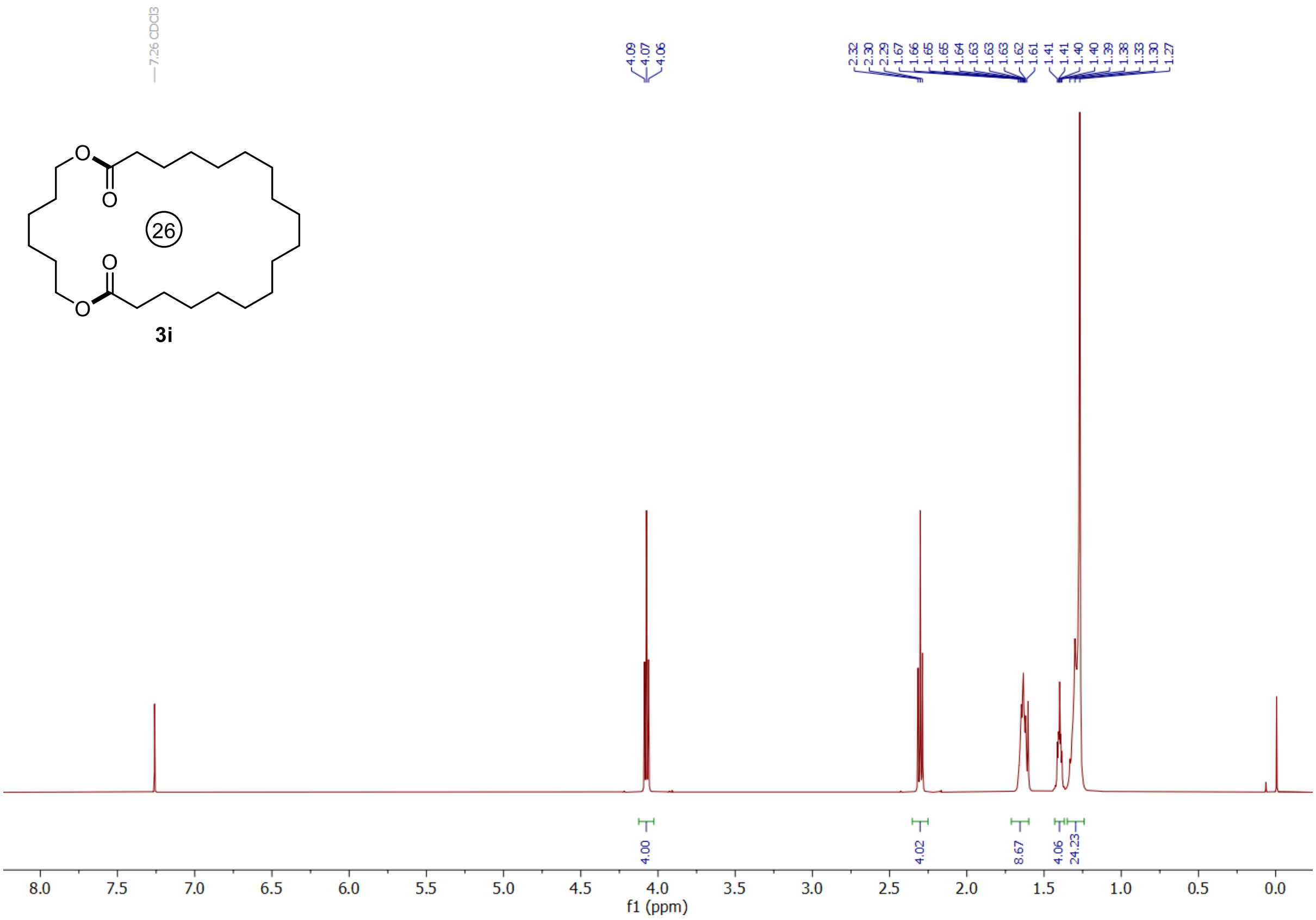

^{13}C{^{1}H} NMR (126 MHz, $CDCl_3$, 298K) of **3i**

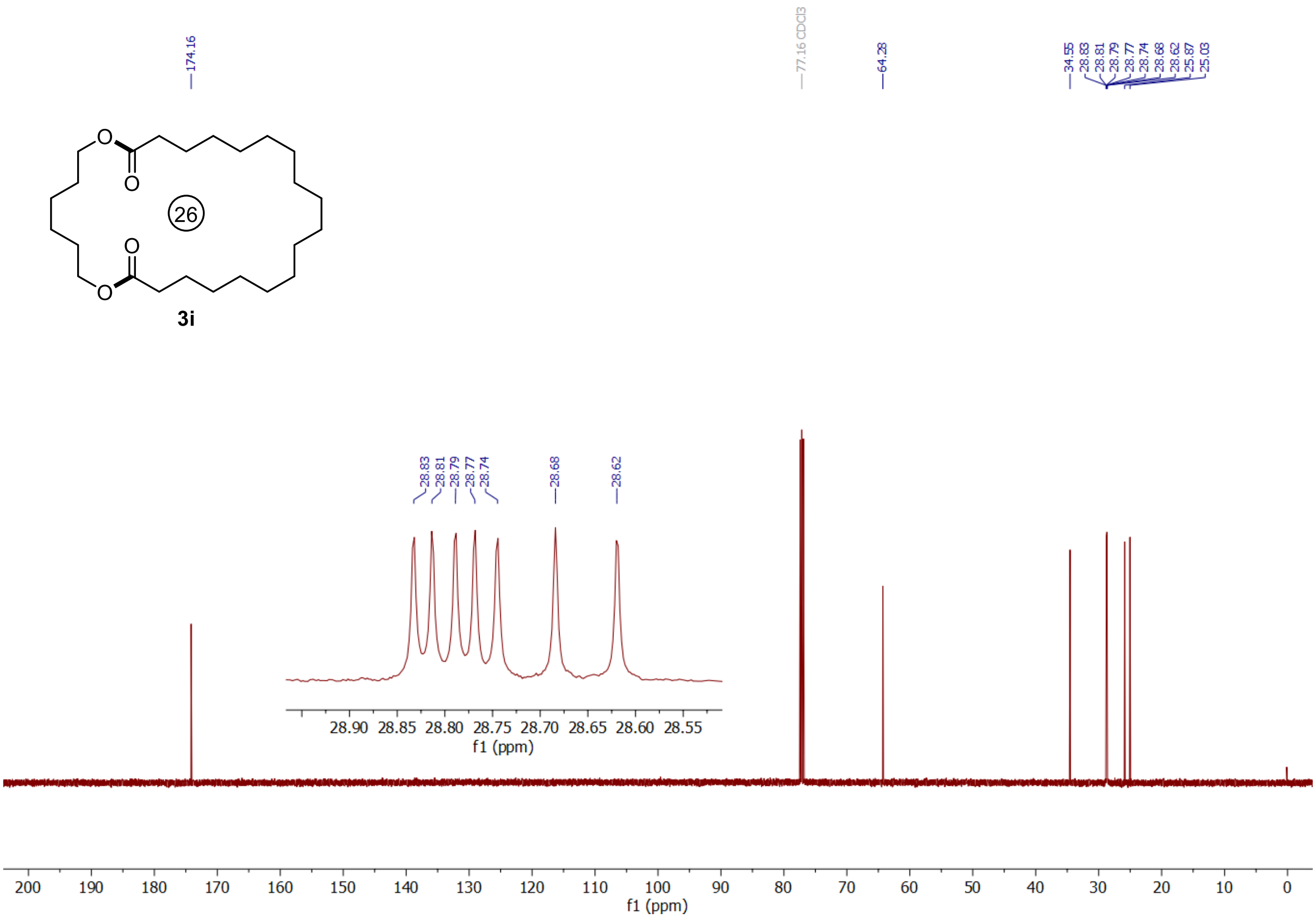

^{1}H NMR (500 MHz, $CDCl_3$, 298K) of **3j**

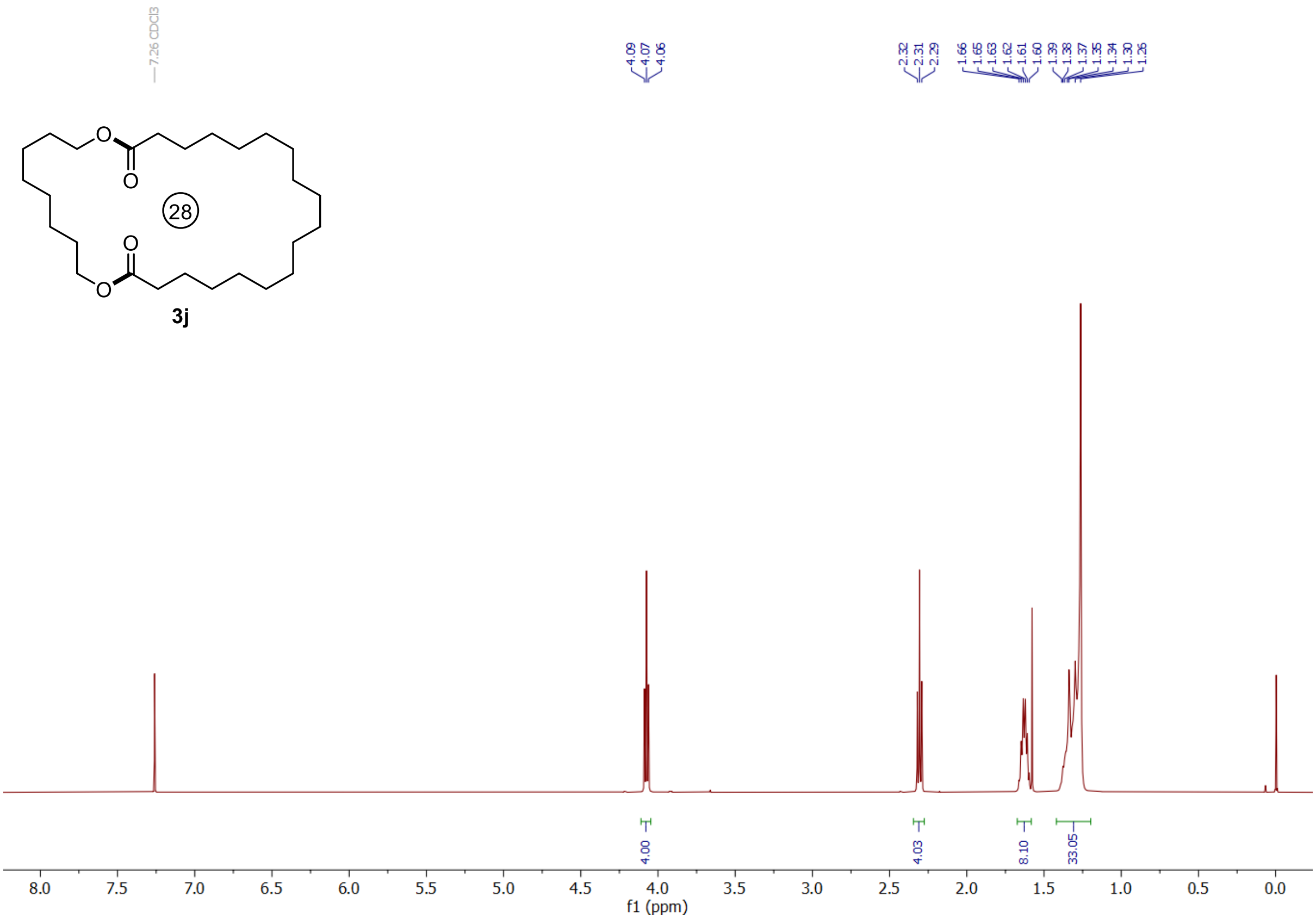

^{13}C{^{1}H} NMR (126 MHz, $CDCl_3$, 298K) of **3j**

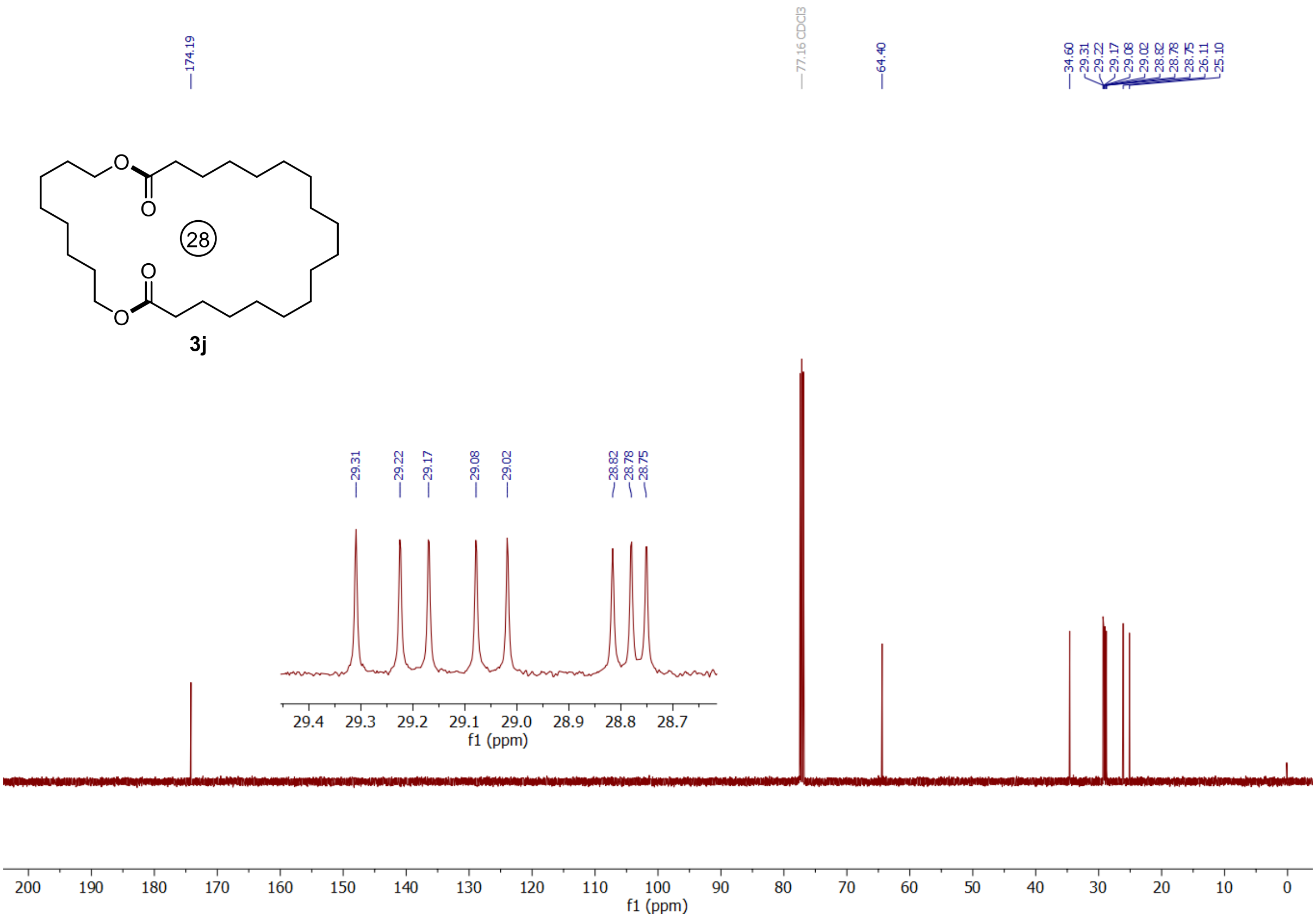

^{1}H NMR (500 MHz, $CDCl_3$, 298K) of **3k**

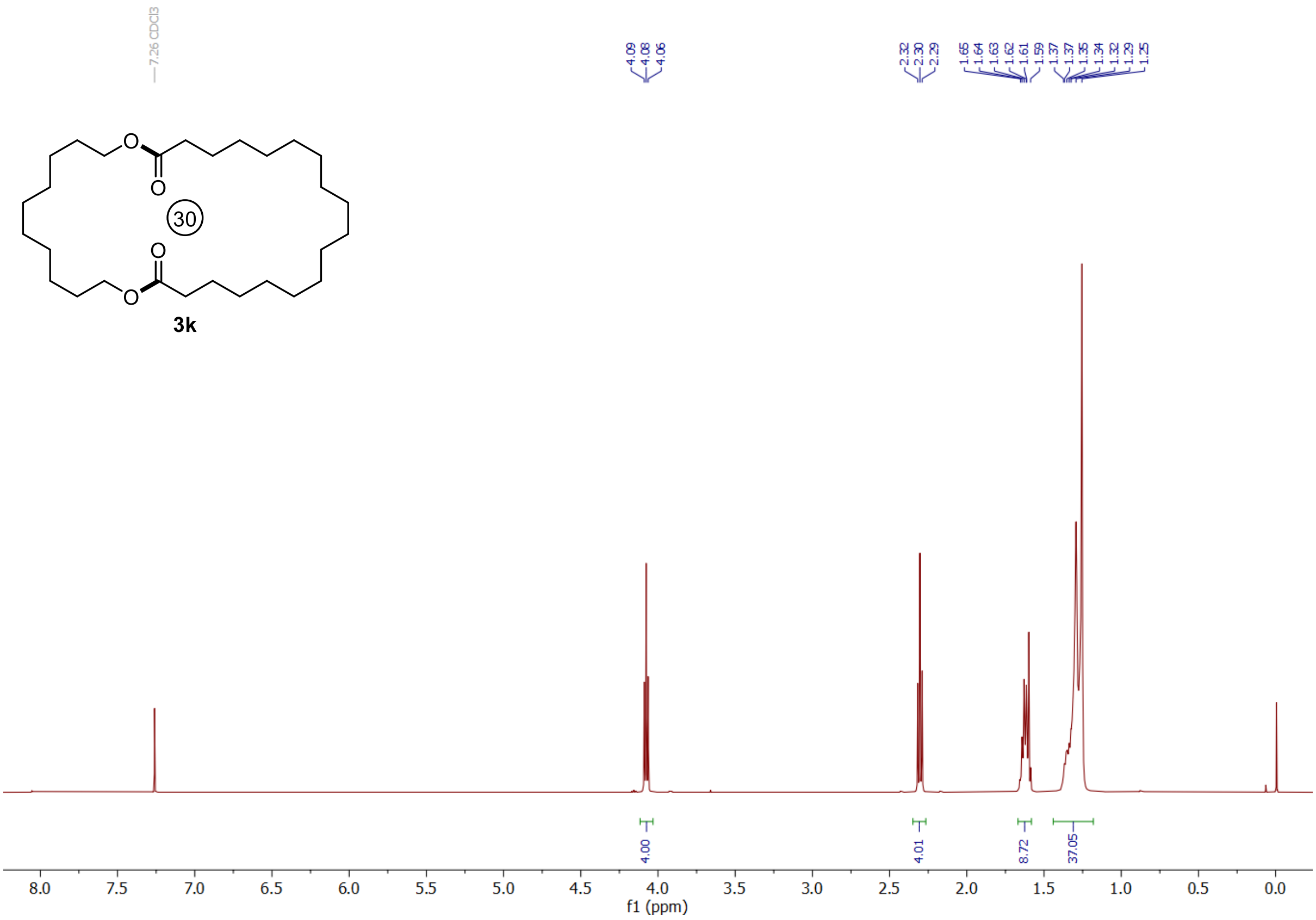

$^{13}C\{^{1}H\}$ NMR (126 MHz, $CDCl_3$, 298K) of **3k**

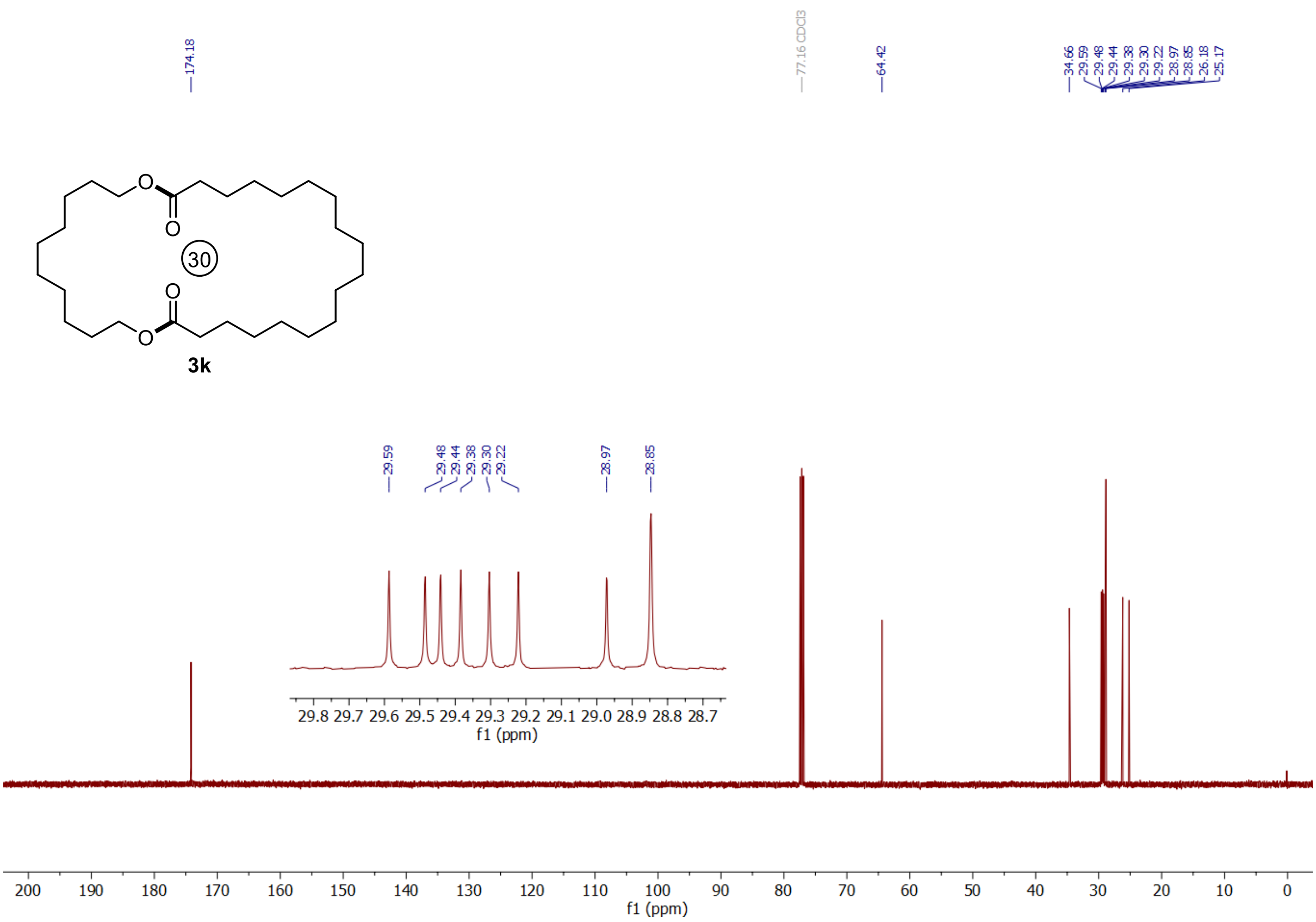

^{1}H NMR (500 MHz, $CDCl_3$, 298K) of **3l**

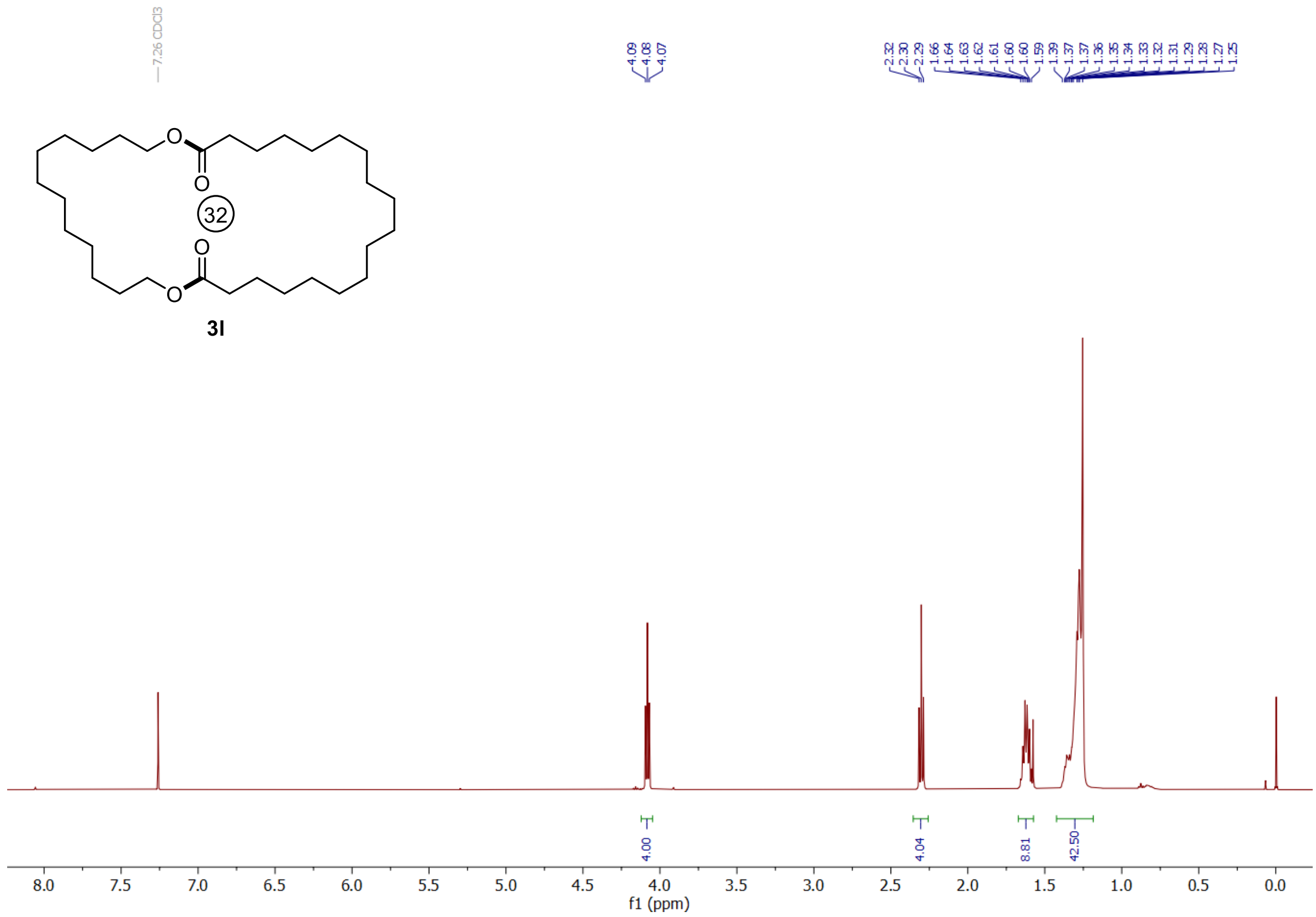

^{13}C{^{1}H} NMR (126 MHz, $CDCl_3$, 298K) of **3l**

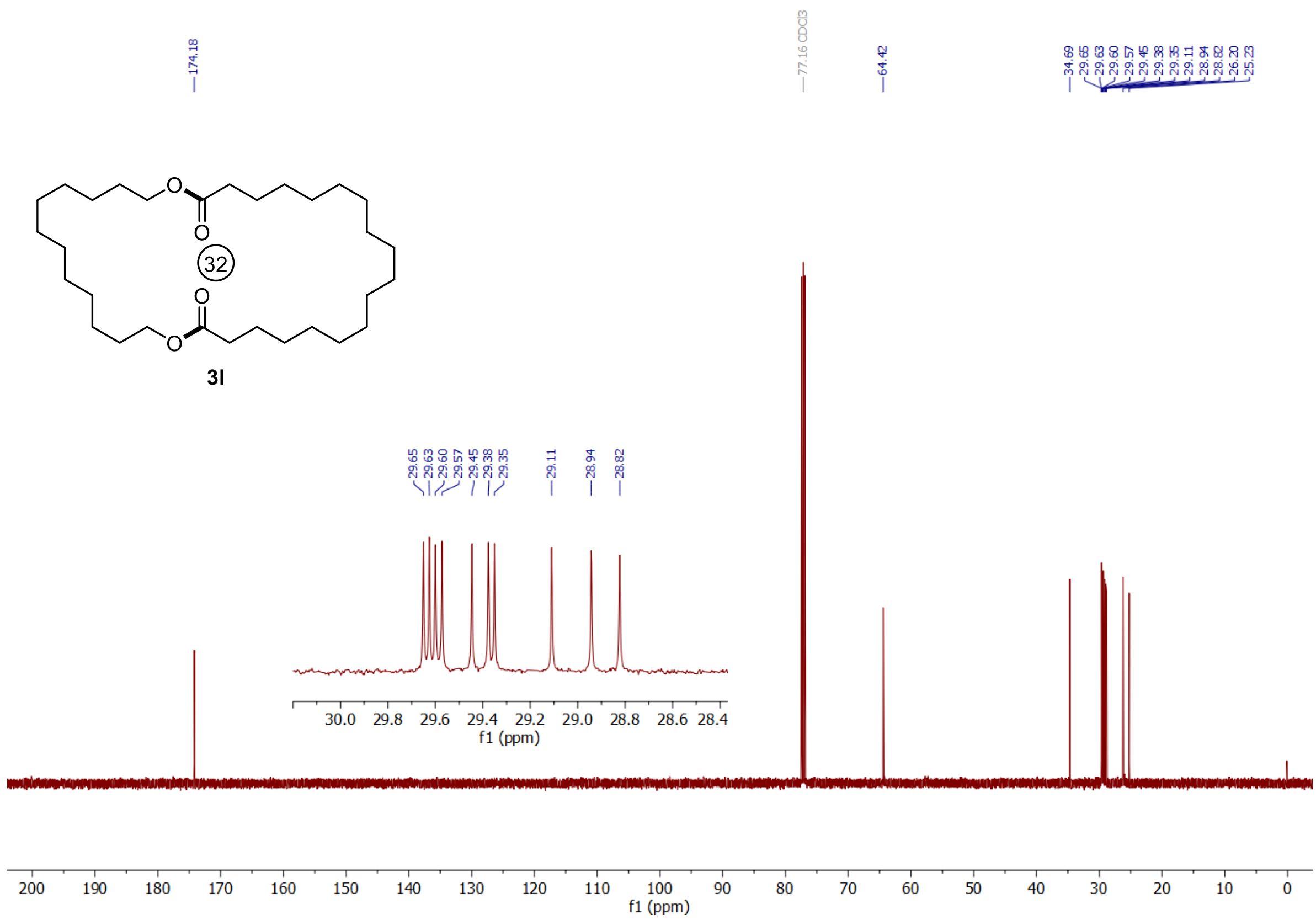

^{1}H NMR (500 MHz, $CDCl_3$, 298K) of **3m**

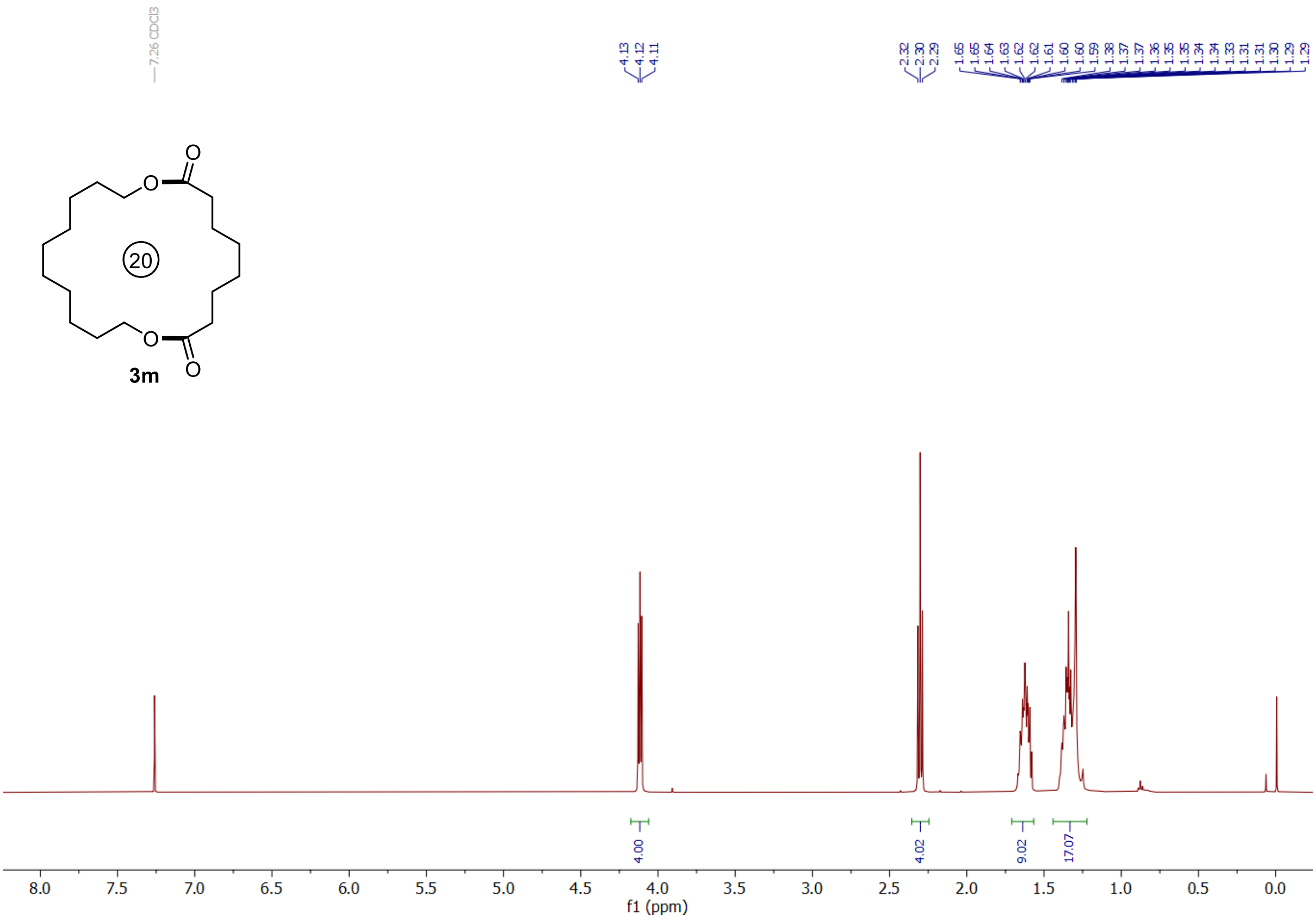

^{13}C{^{1}H} NMR (126 MHz, $CDCl_3$, 298K) of **3m**

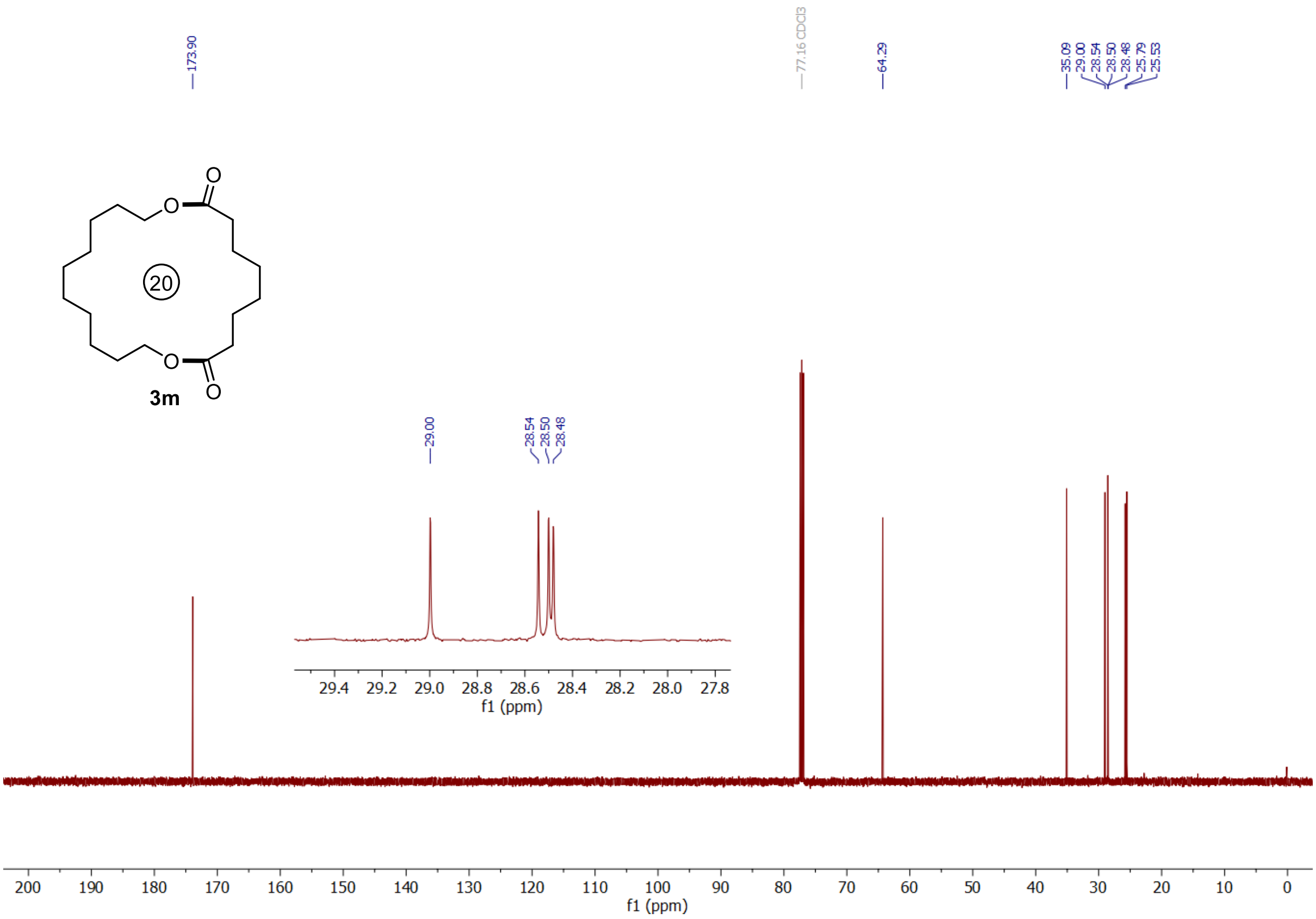

^{1}H NMR (500 MHz, $CDCl_3$, 298K) of **3n**

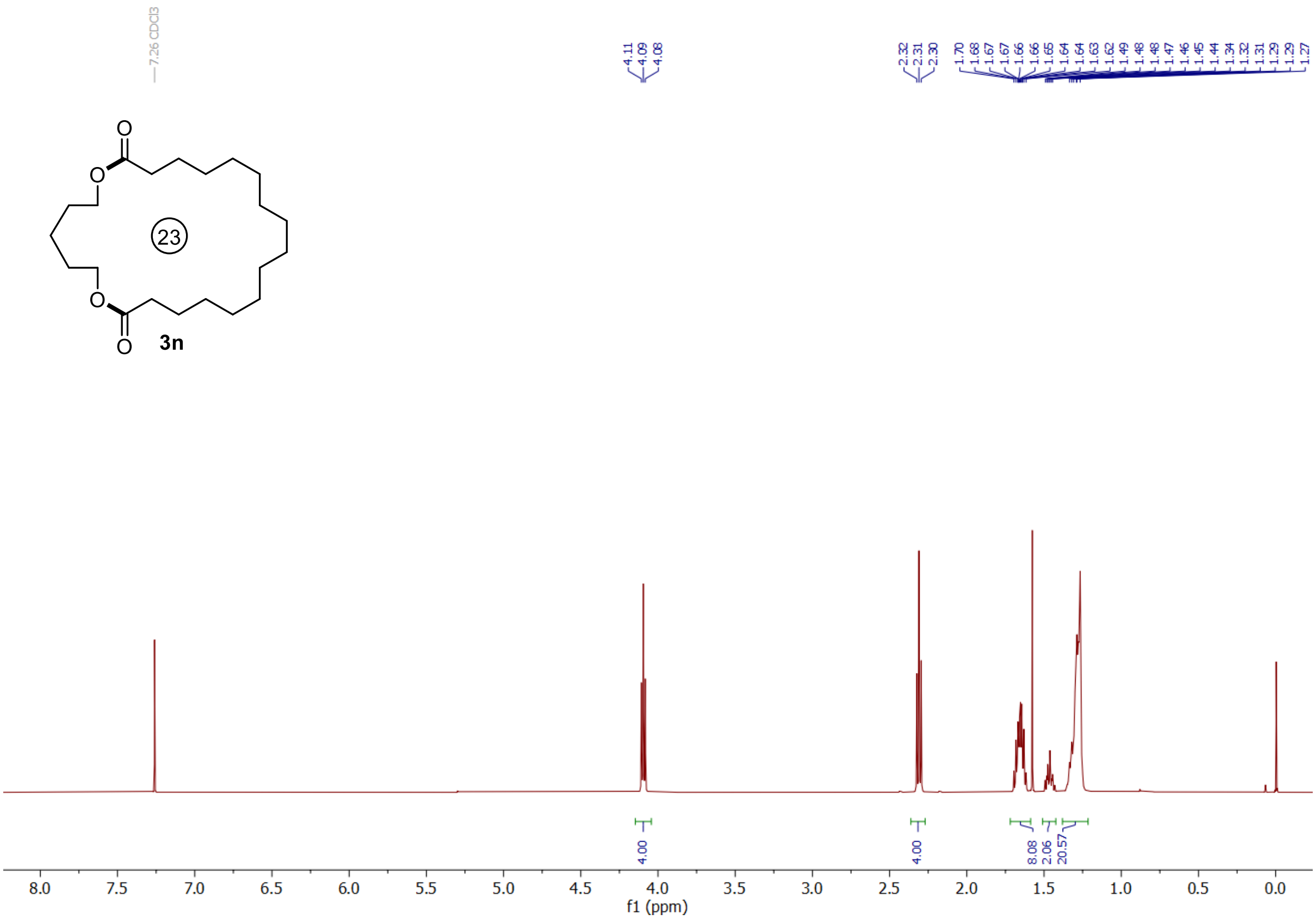

$^{13}C\{^{1}H\}$ NMR (126 MHz, $CDCl_3$, 298K) of **3n**

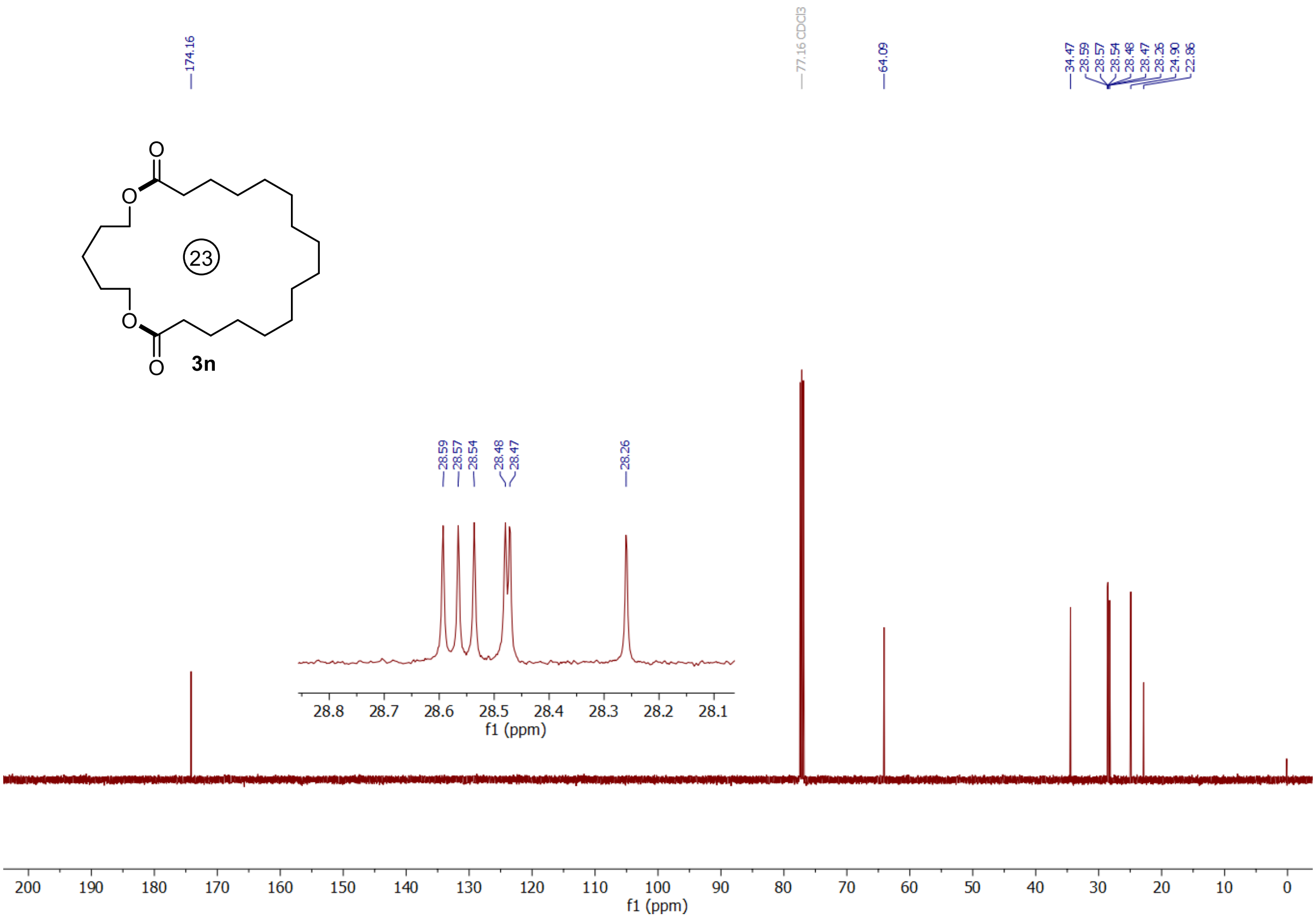

^1H NMR (500 MHz, $CDCl_3$, 298K) of **3o**

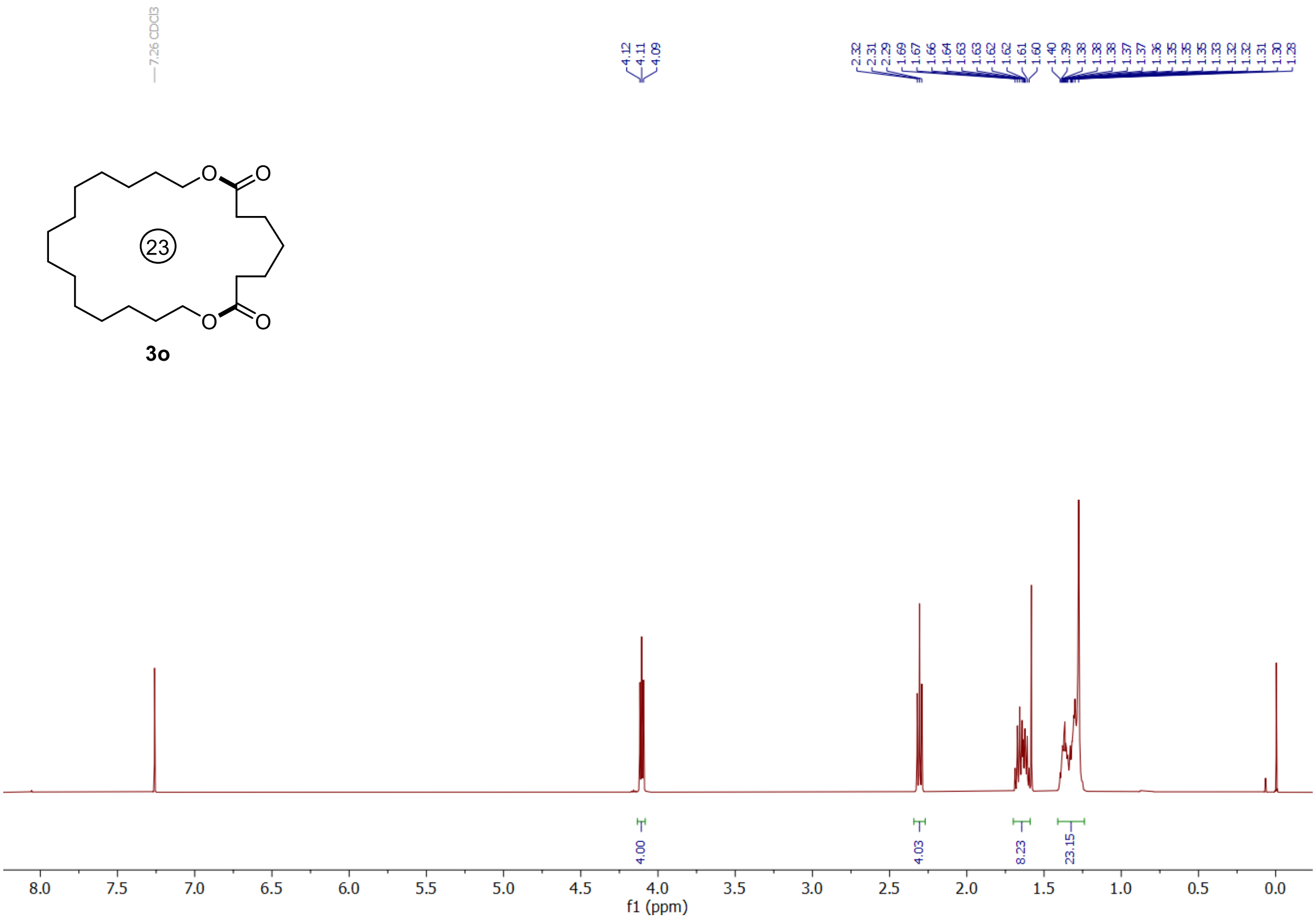

$^{13}C\{^1H\}$ NMR (126 MHz, $CDCl_3$, 298K) of **3o**

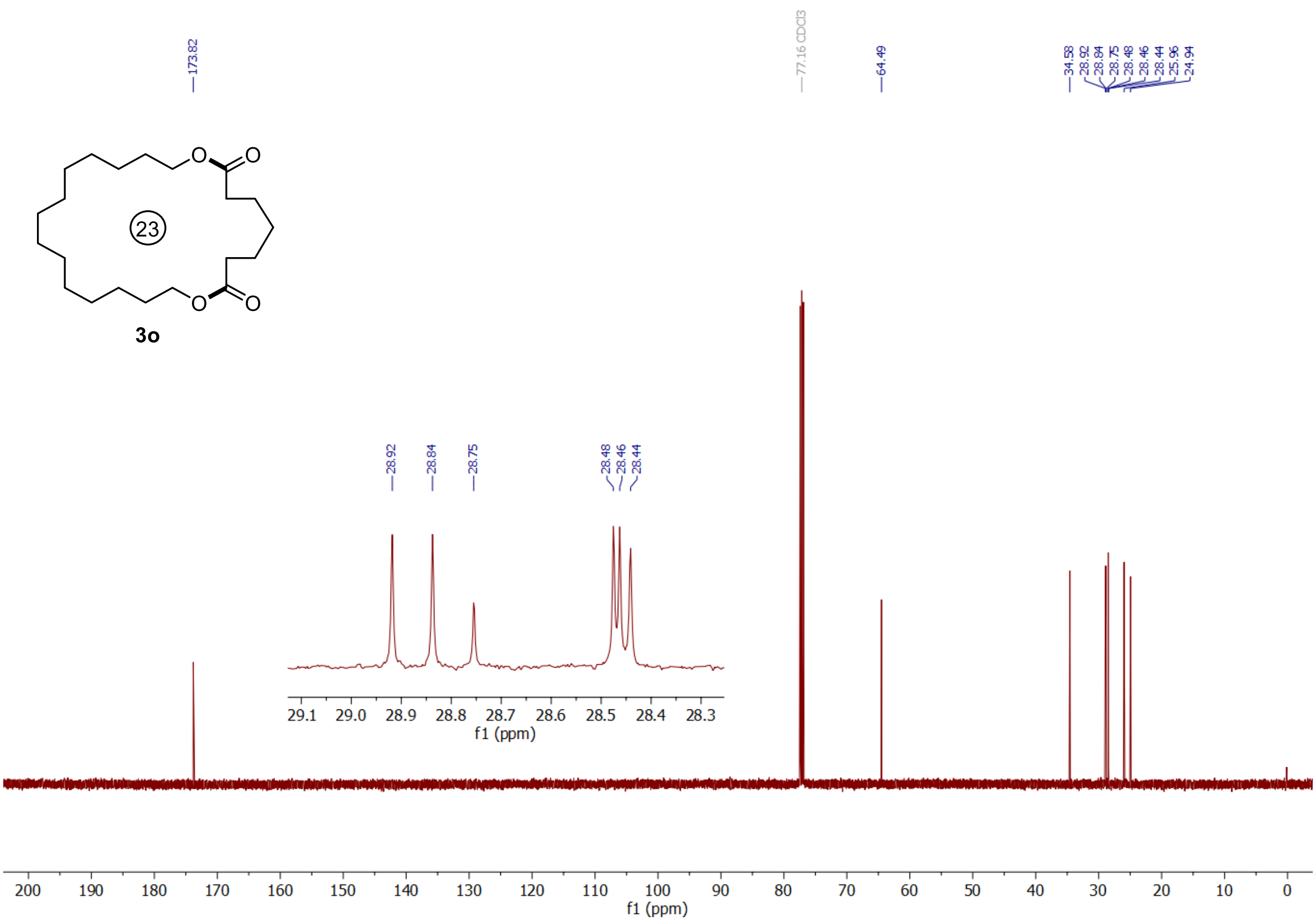

^{1}H NMR (500 MHz, $CDCl_3$, 298K) of **3p**

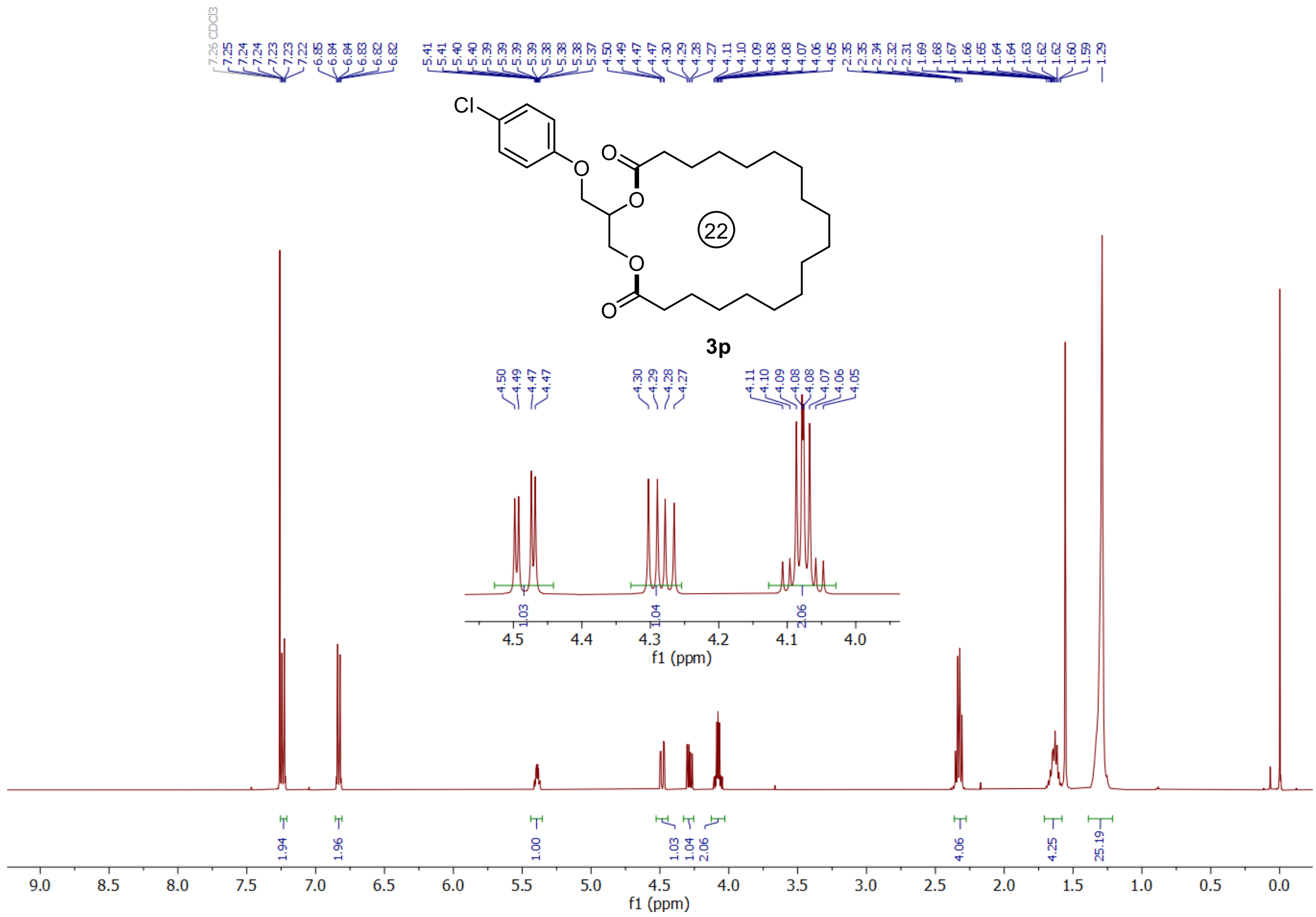

^{13}C{^{1}H} NMR (126 MHz, $CDCl_3$, 298K) of **3p**

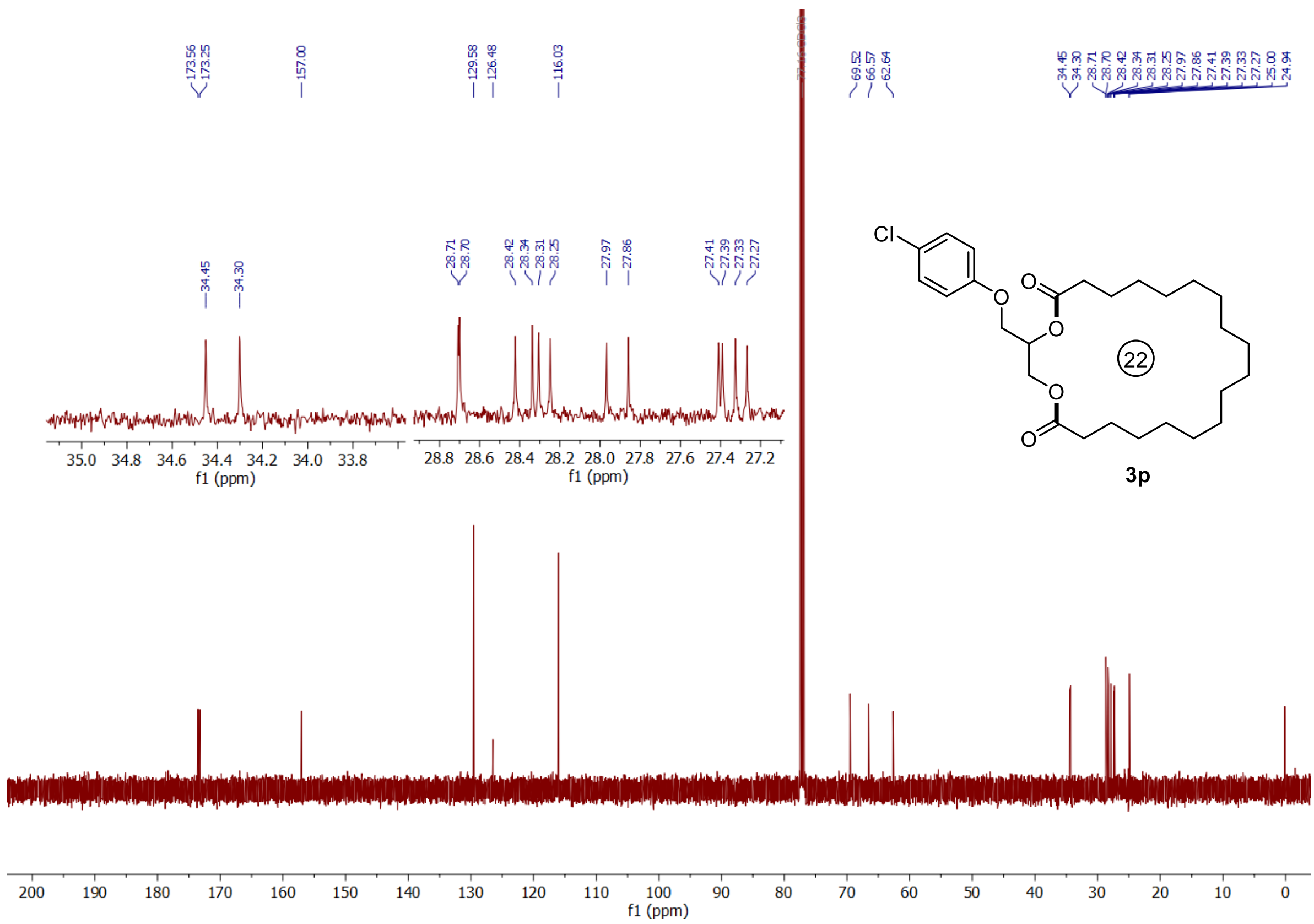

^{1}H NMR (500 MHz, $CDCl_3$, 298K) of **3q**

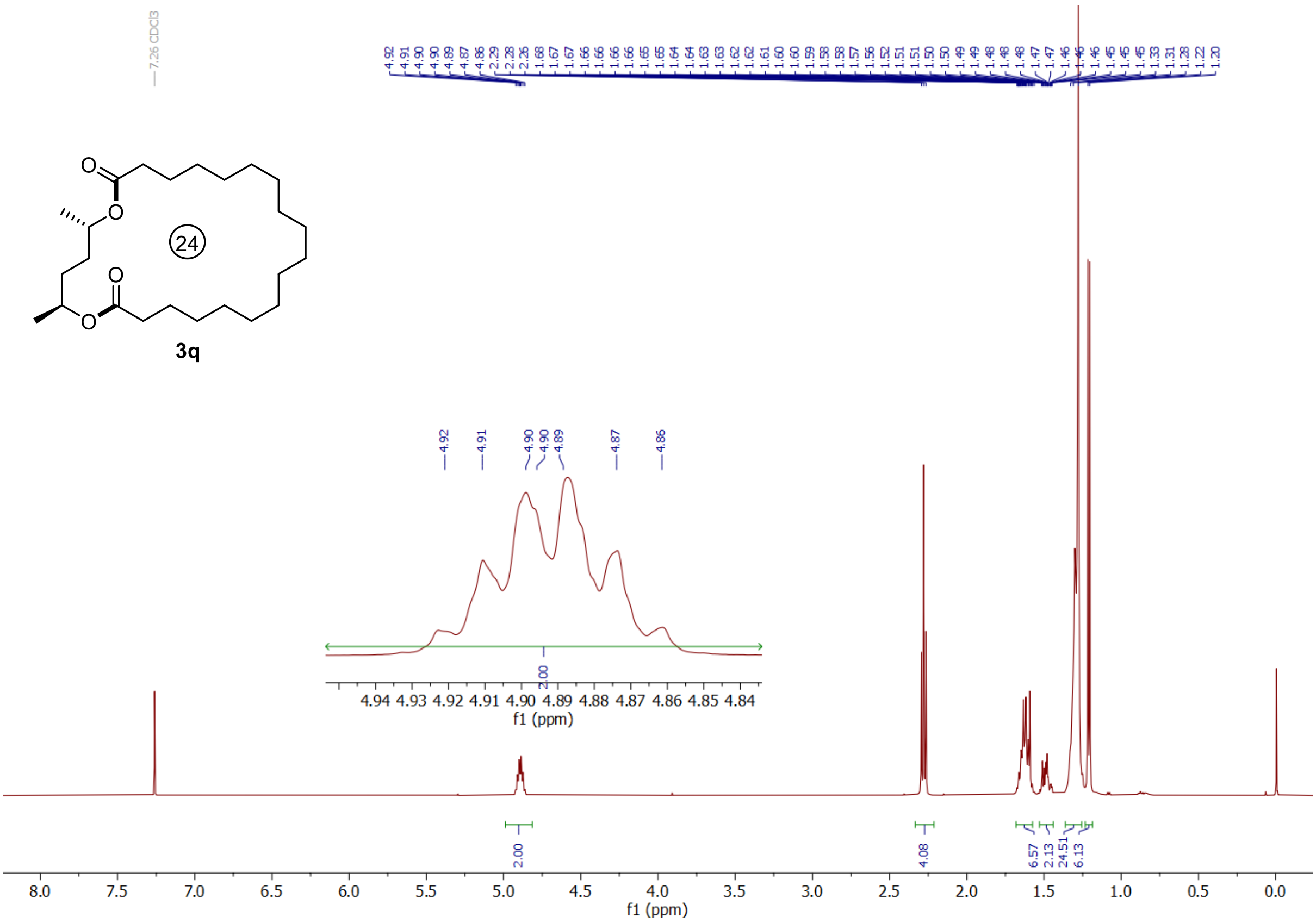

^{13}C{^{1}H} NMR (126 MHz, $CDCl_3$, 298K) of **3q**

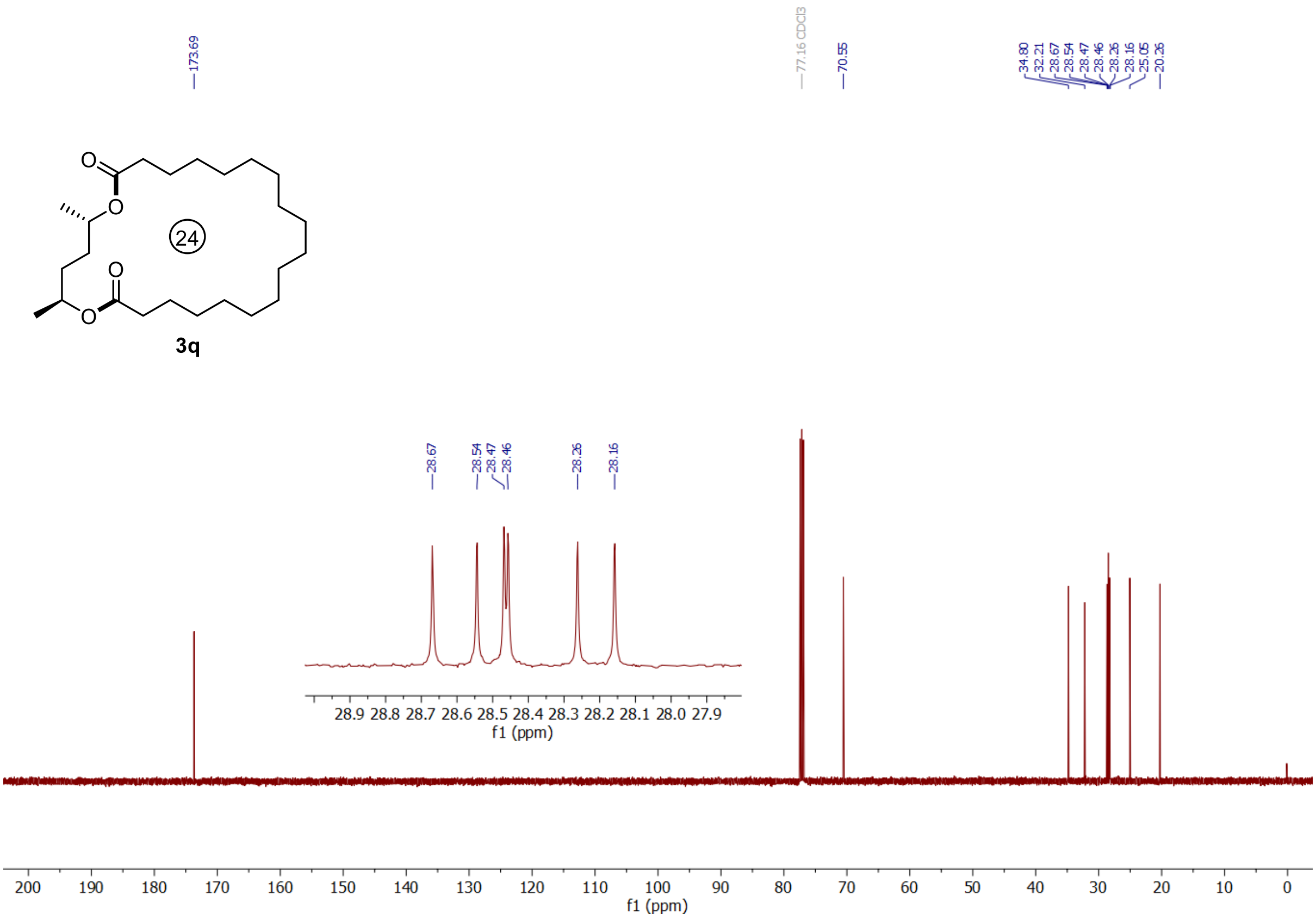

^{1}H NMR (500 MHz, $CDCl_3$, 298K) of **3r**

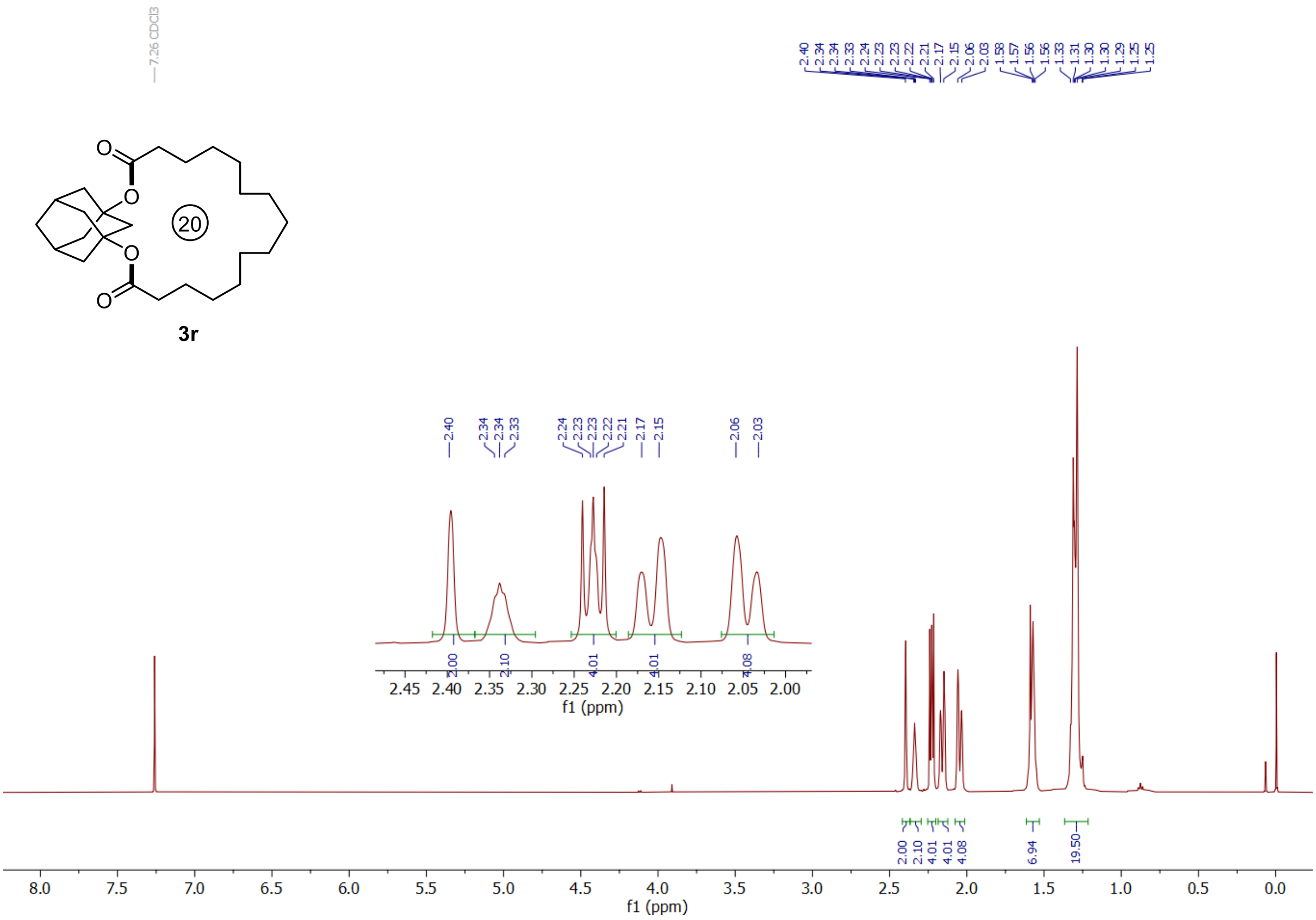

^{13}C{^{1}H} NMR (126 MHz, $CDCl_3$, 298K) of **3r**

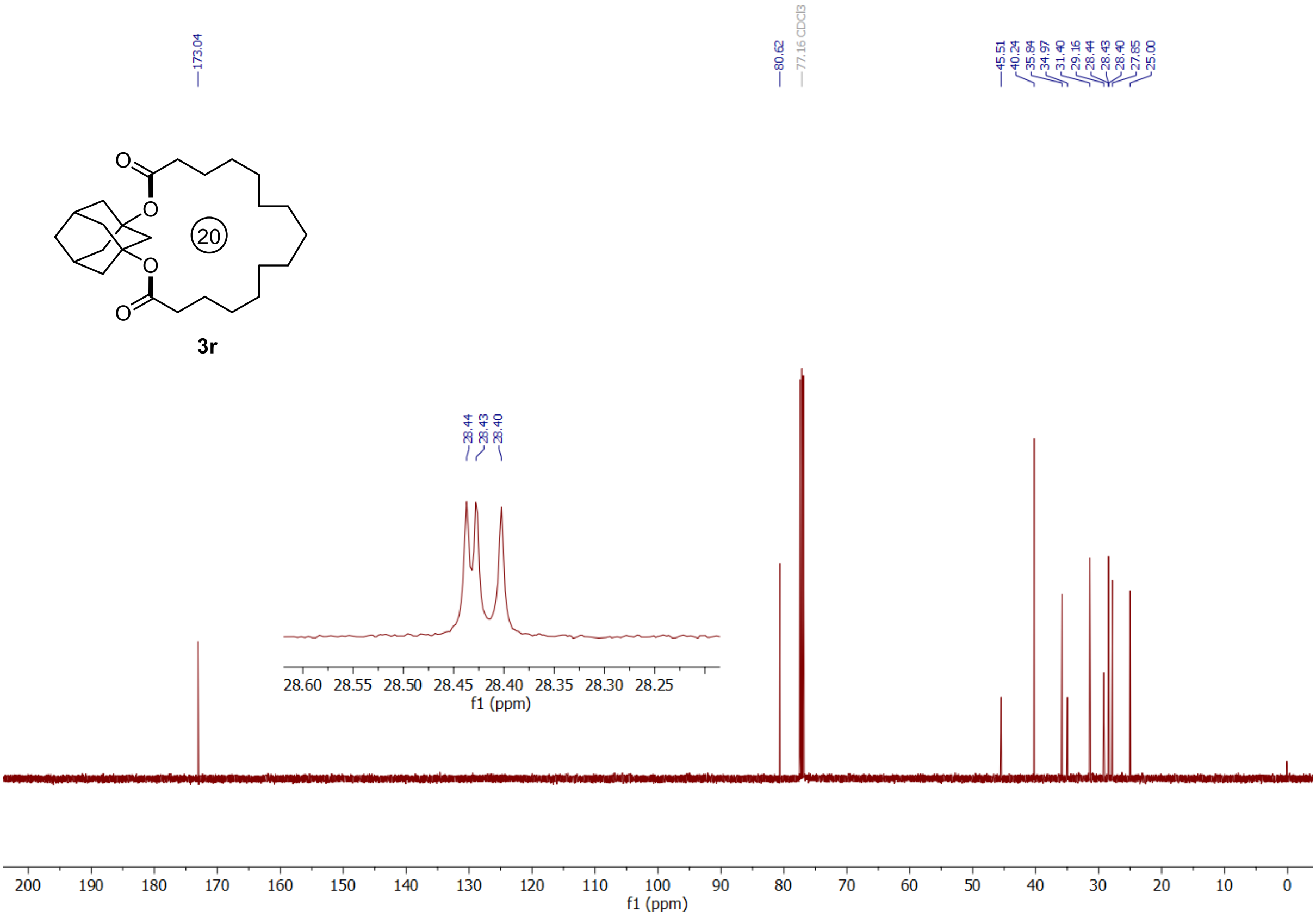

^{1}H NMR (500 MHz, $CDCl_3$, 298K) of **3s**

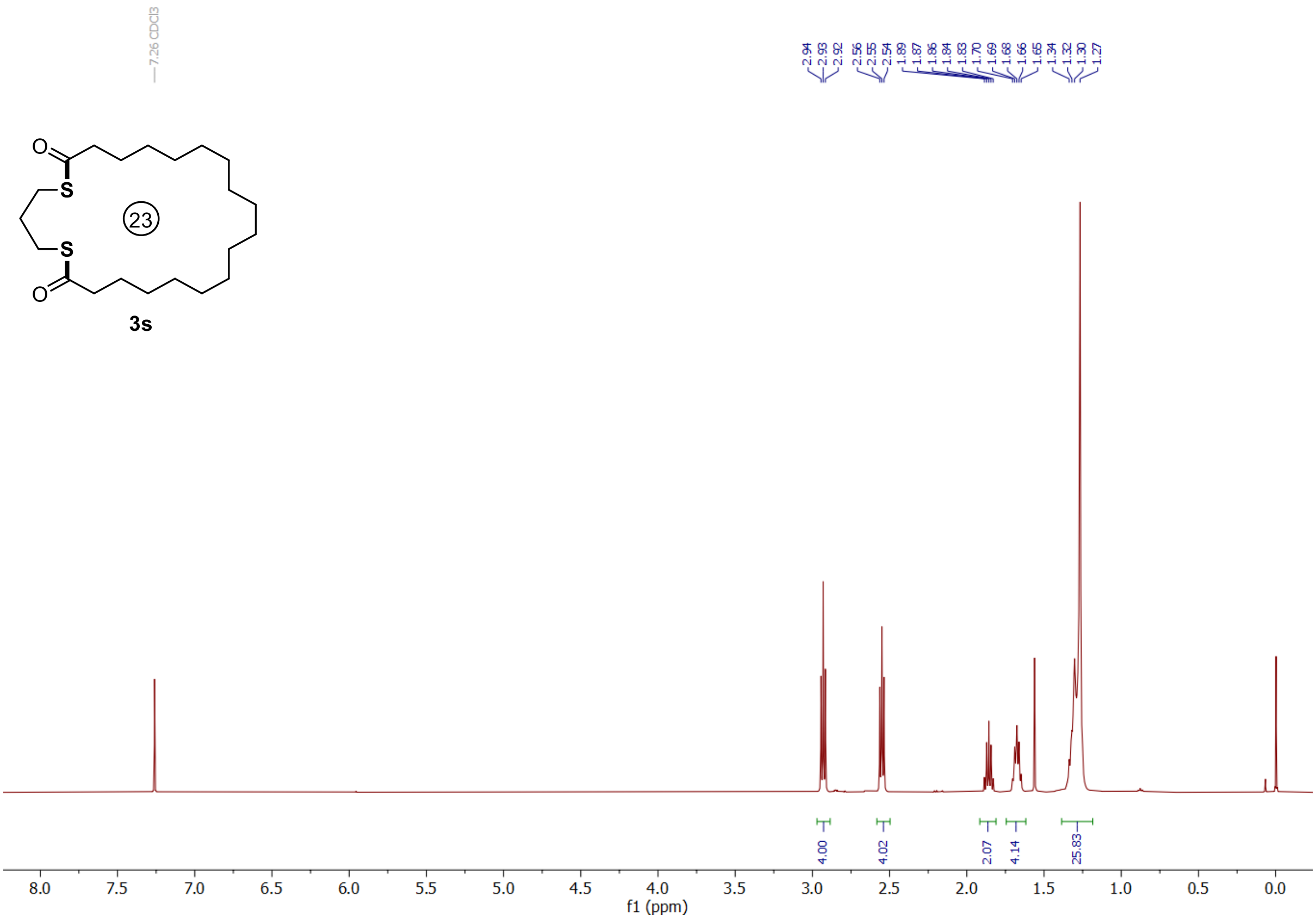

$^{13}C\{^1H\}$ NMR (126 MHz, $CDCl_3$, 298K) of **3s**

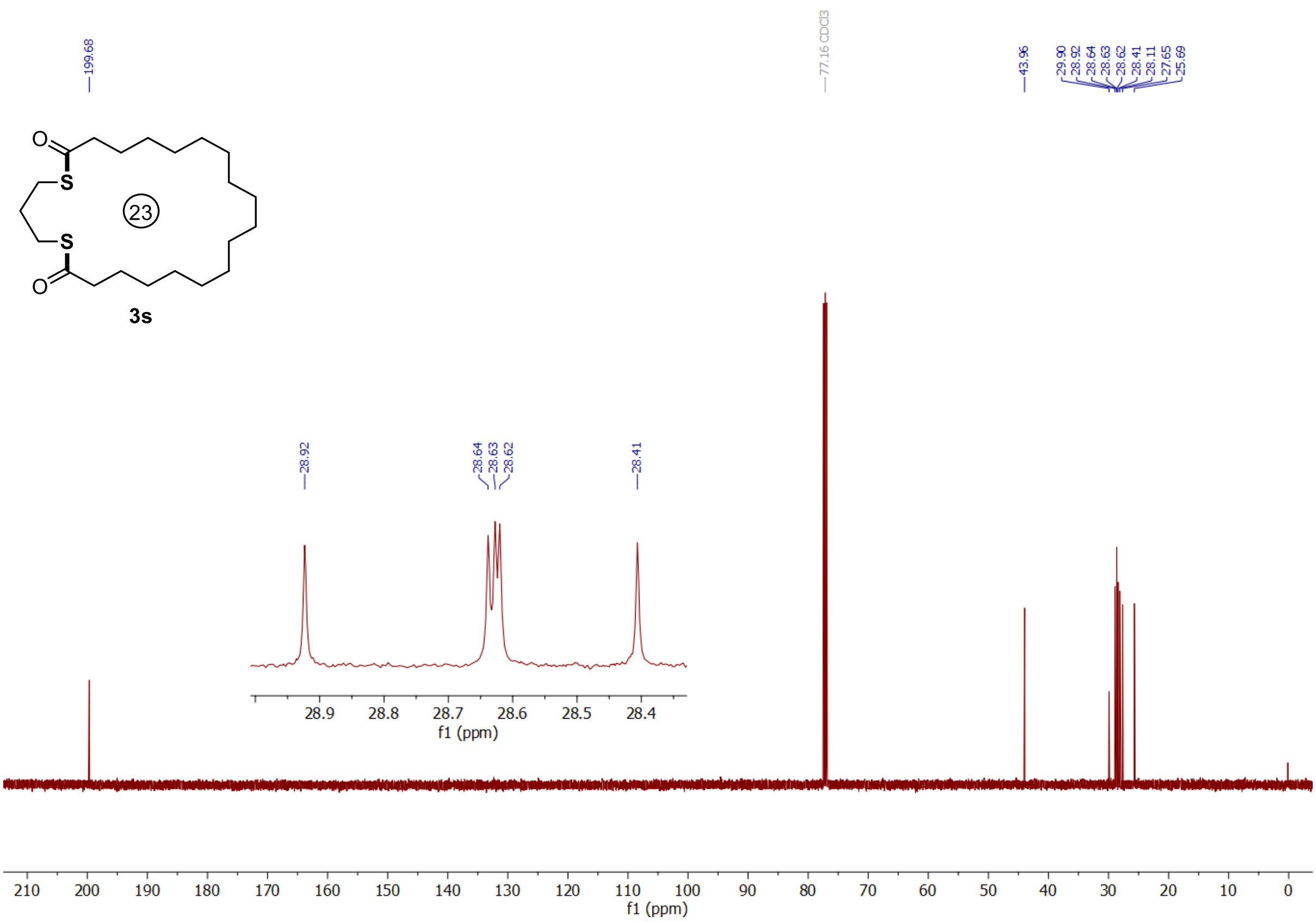

^{1}H NMR (500 MHz, $CDCl_3$, 298K) of **3t**

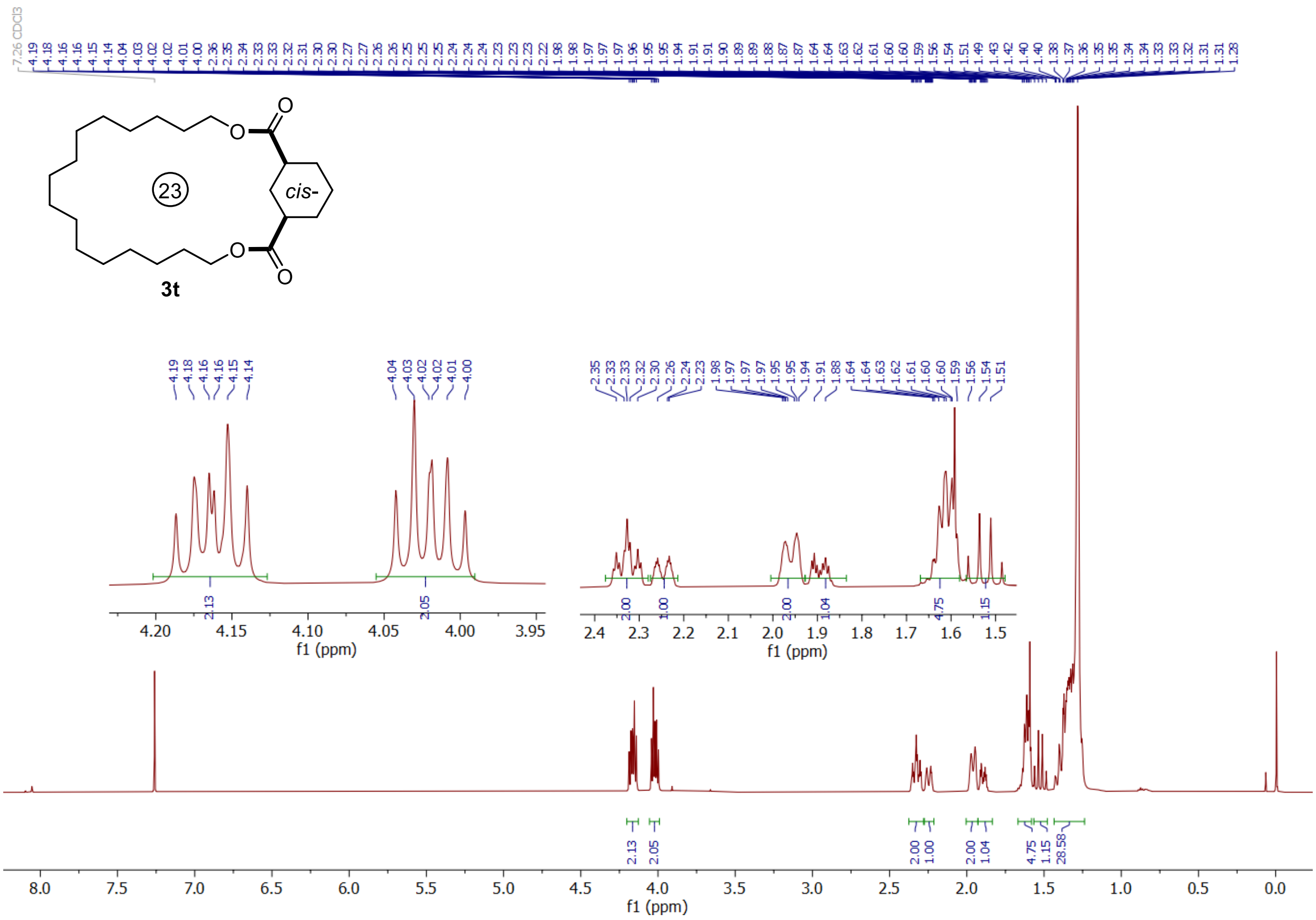

$^{13}C\{^{1}H\}$ NMR (126 MHz, $CDCl_3$, 298K) of **3t**

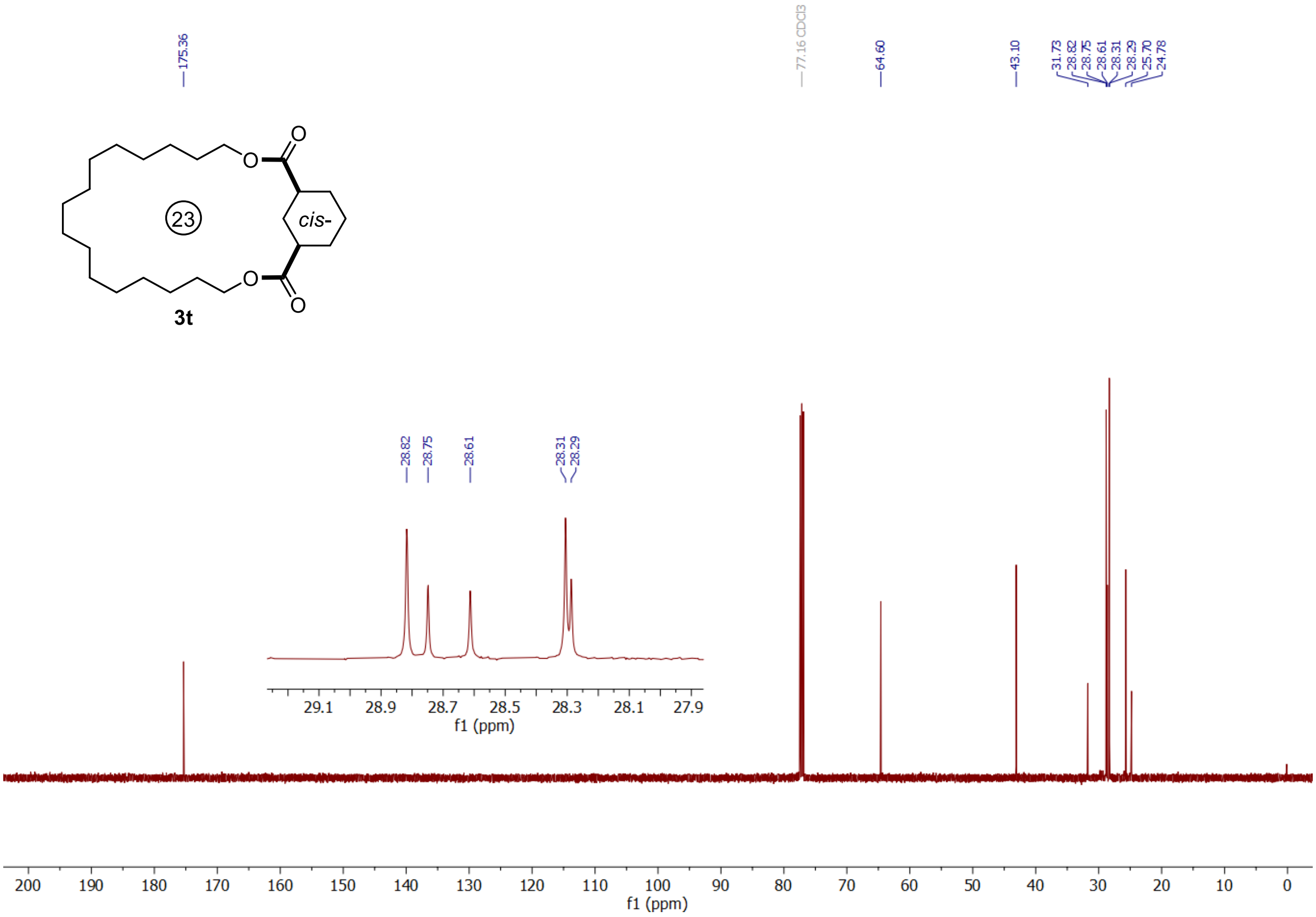

^{1}H NMR (500 MHz, $CDCl_3$, 298K) of **3u**

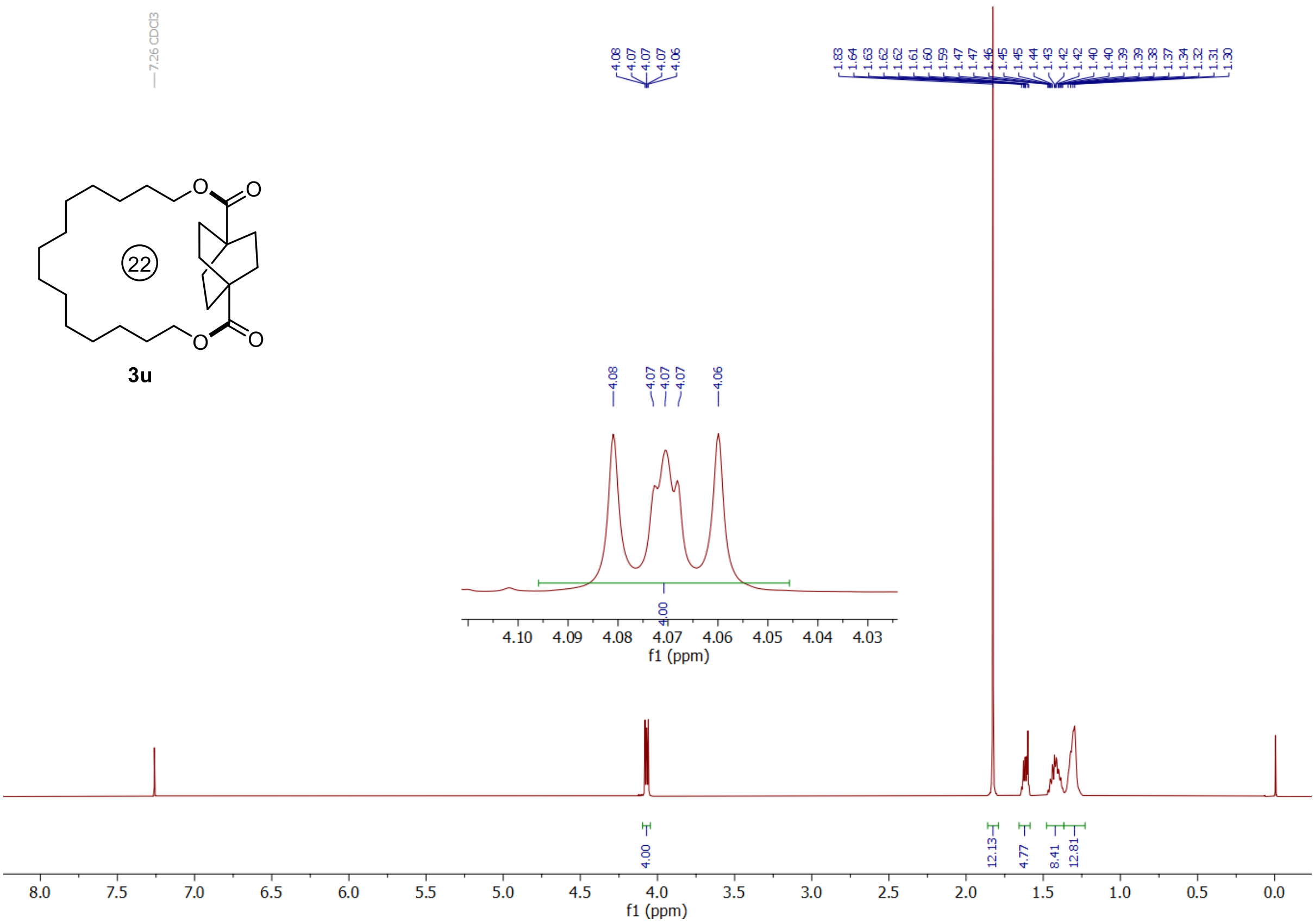

$^{13}C\{^1H\}$ NMR (126 MHz, $CDCl_3$, 298K) of **3u**

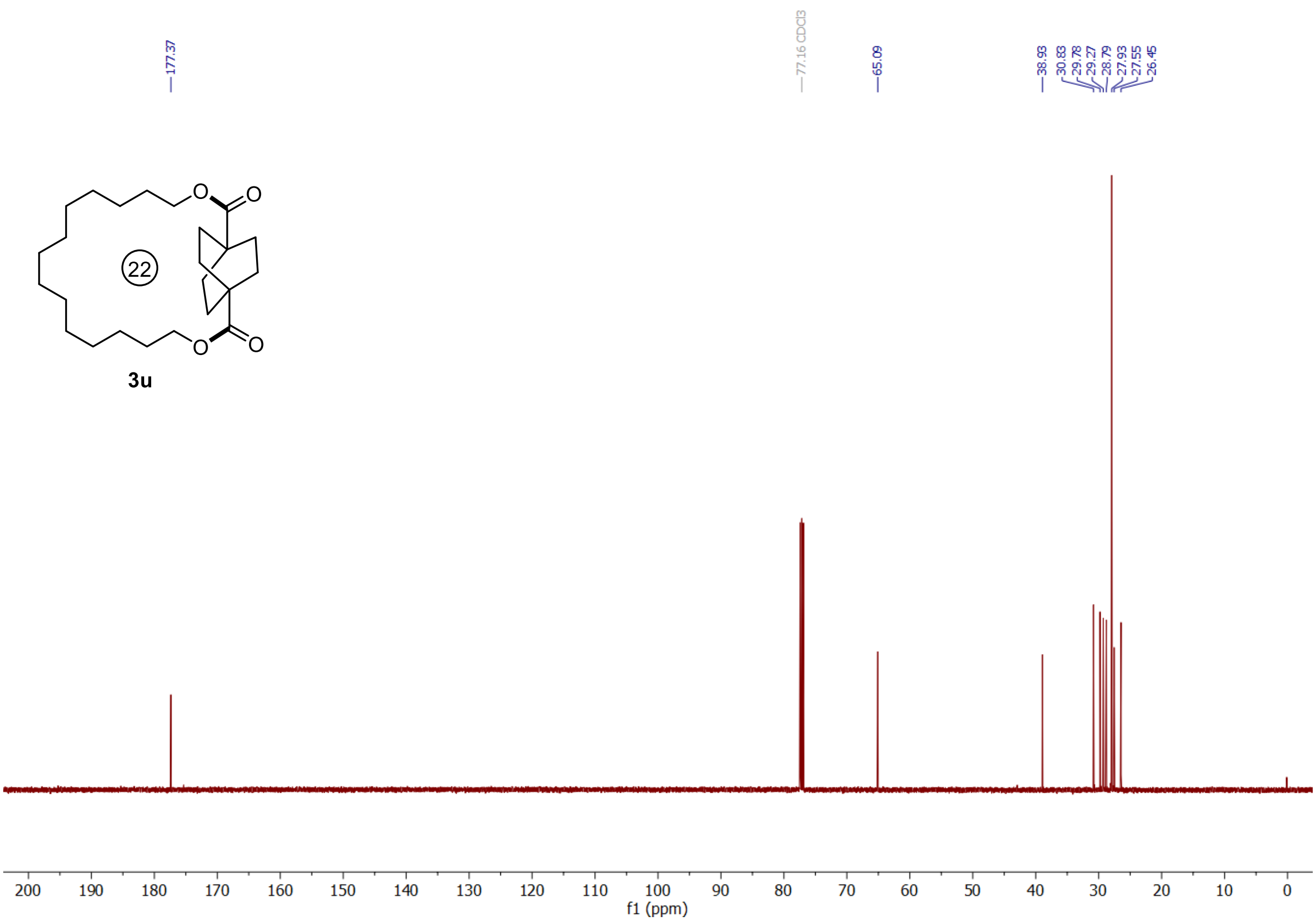

^{1}H NMR (500 MHz, $CDCl_3$, 298K) of **3v**

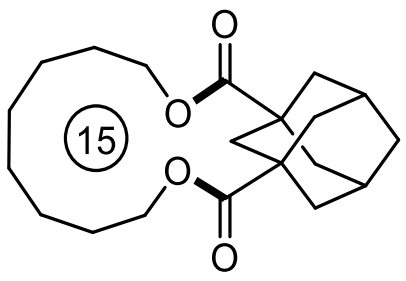

3v

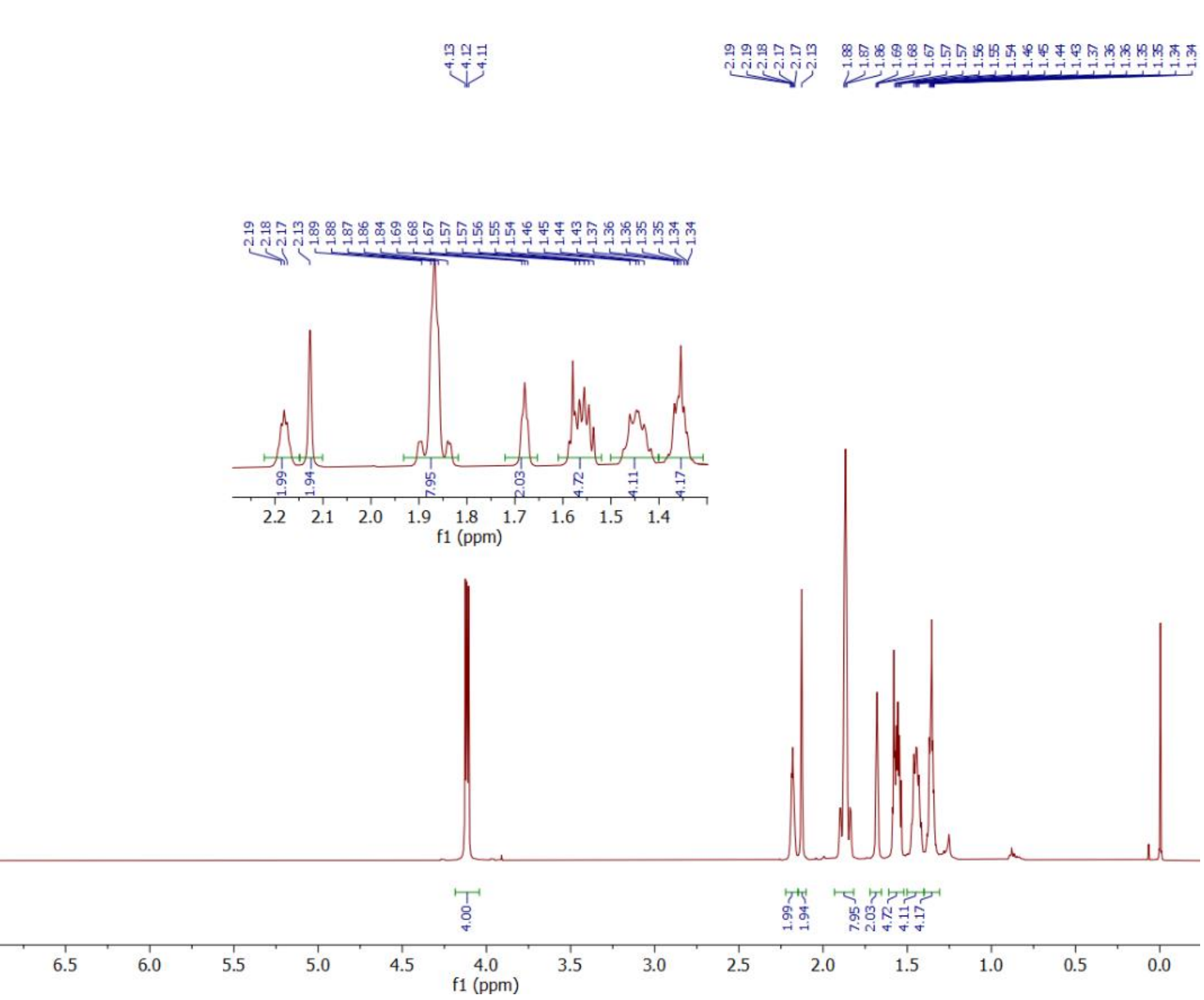

$^{13}C\{^{1}H\}$ NMR (126 MHz, $CDCl_3$, 298K) of **3v**

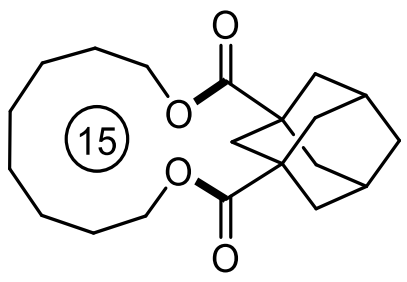

3v

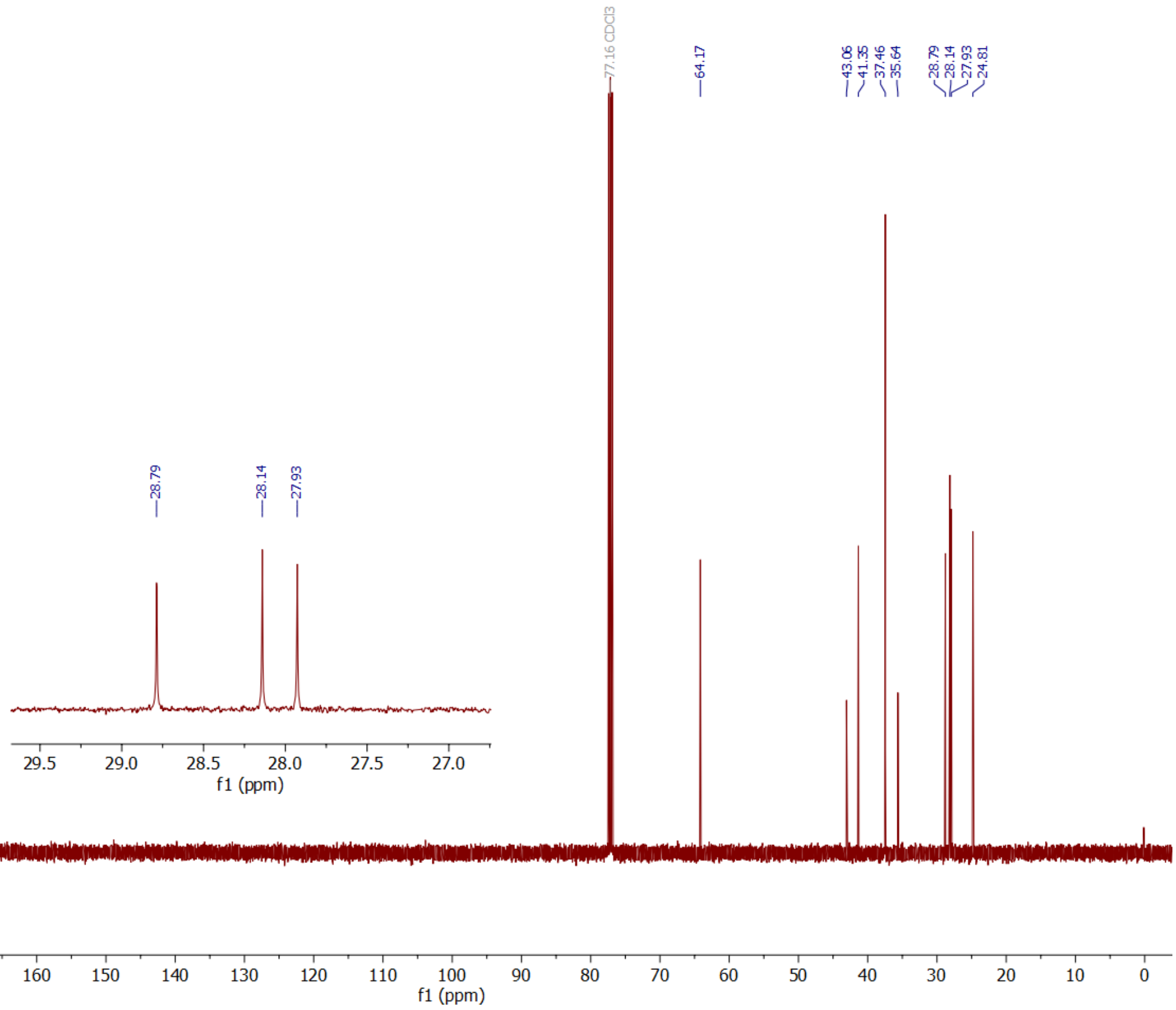

^{1}H NMR (500 MHz, $CDCl_3$, 298K) of **3w**

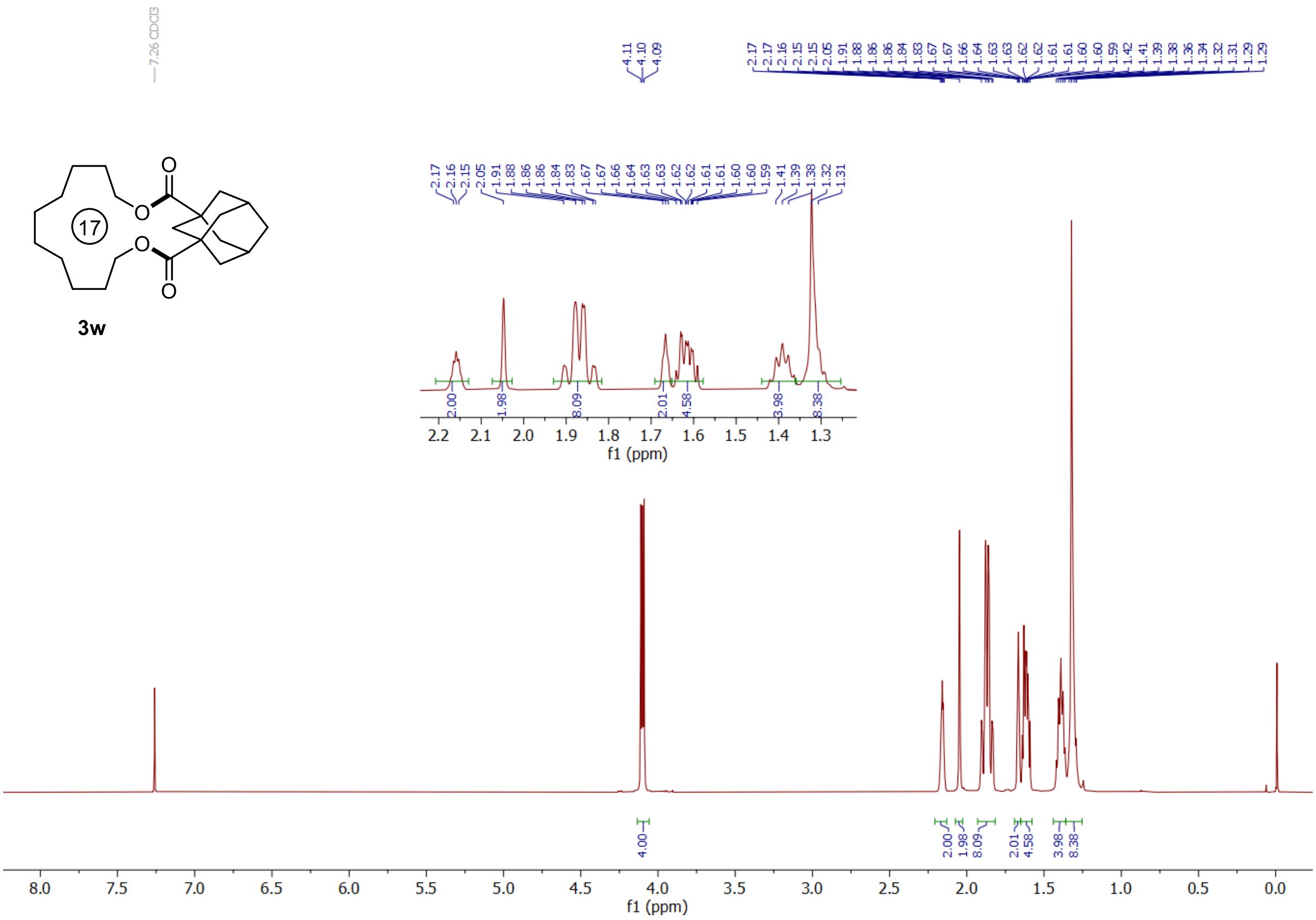

$^{13}C\{^1H\}$ NMR (126 MHz, $CDCl_3$, 298K) of **3w**

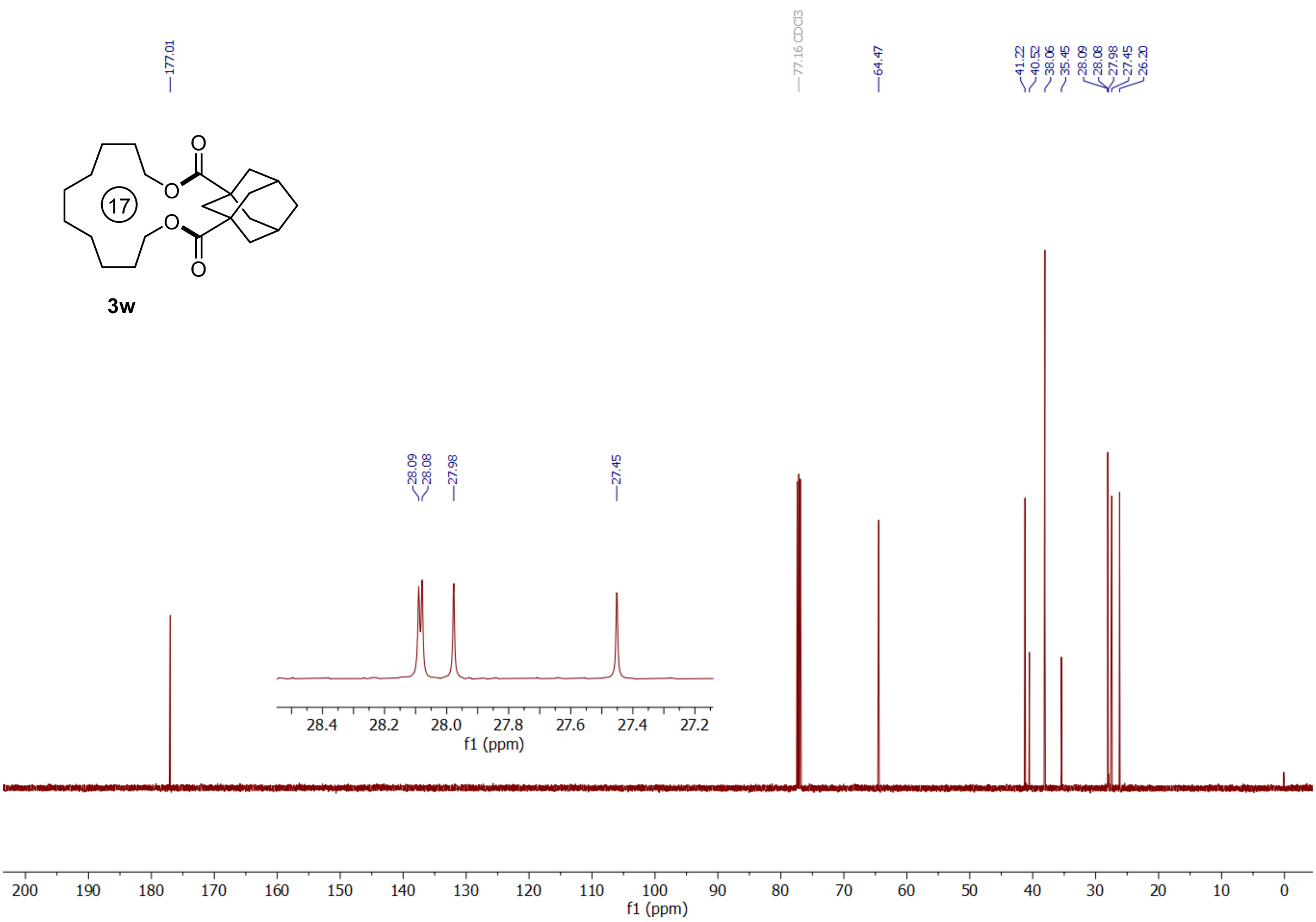

^{1}H NMR (500 MHz, $CDCl_3$, 298K) of **3x**

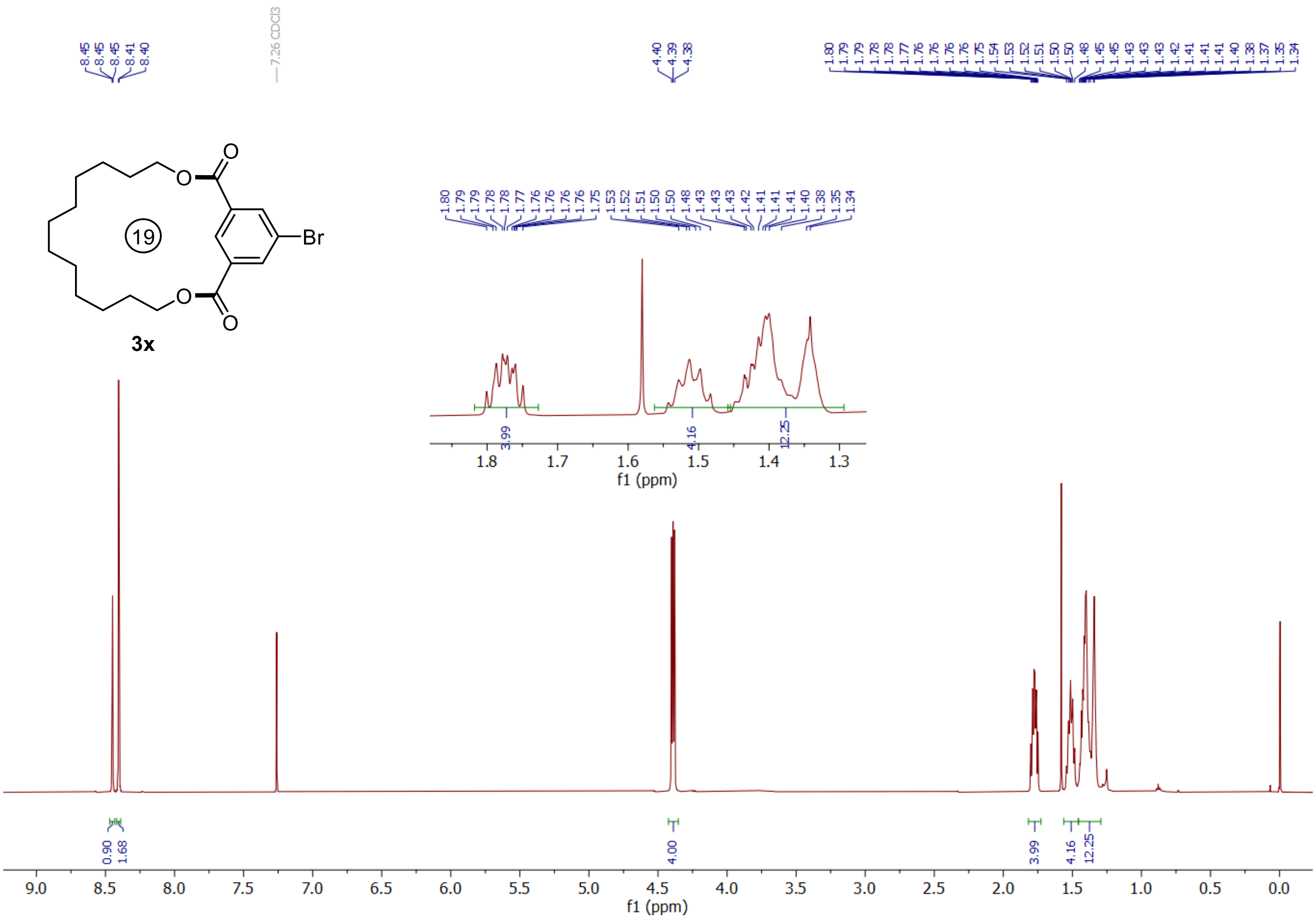

^{13}C{^{1}H} NMR (126 MHz, $CDCl_3$, 298K) of **3x**

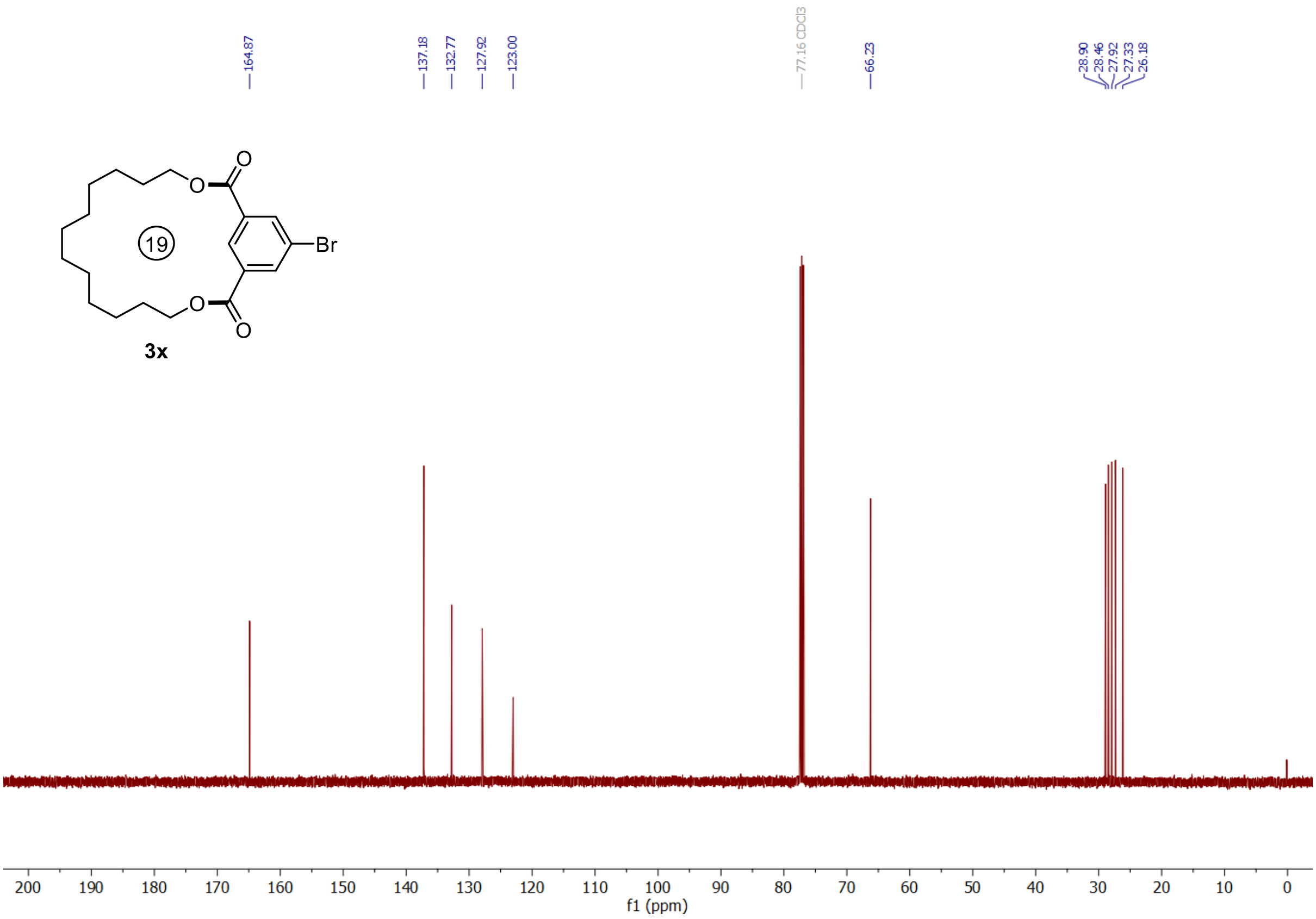

^{1}H NMR (500 MHz, $CDCl_3$, 298K) of **3y**

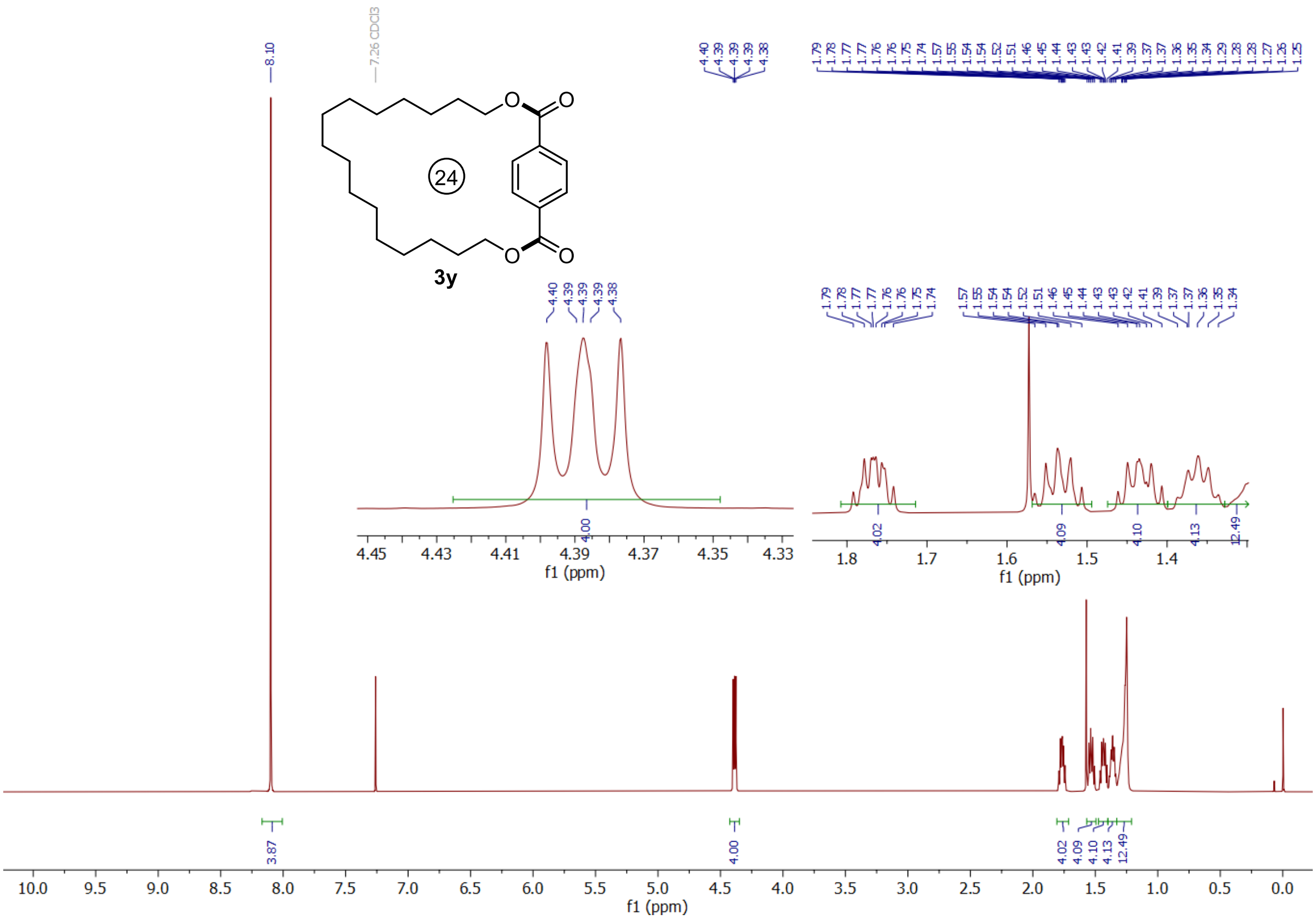

^{13}C{^{1}H} NMR (126 MHz, $CDCl_3$, 298K) of **3y**

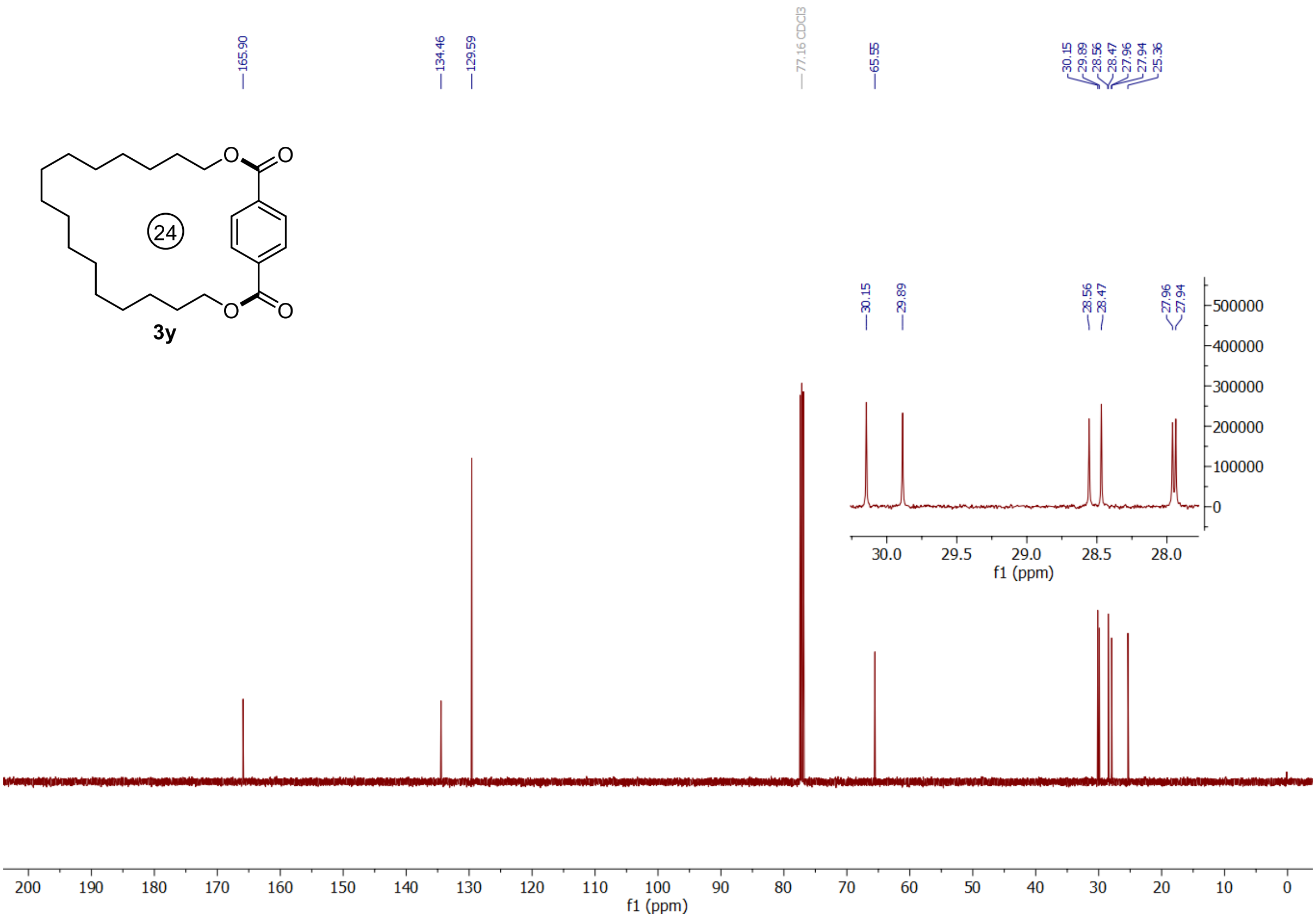

^{1}H NMR (500 MHz, $CDCl_3$, 298K) of **3z**

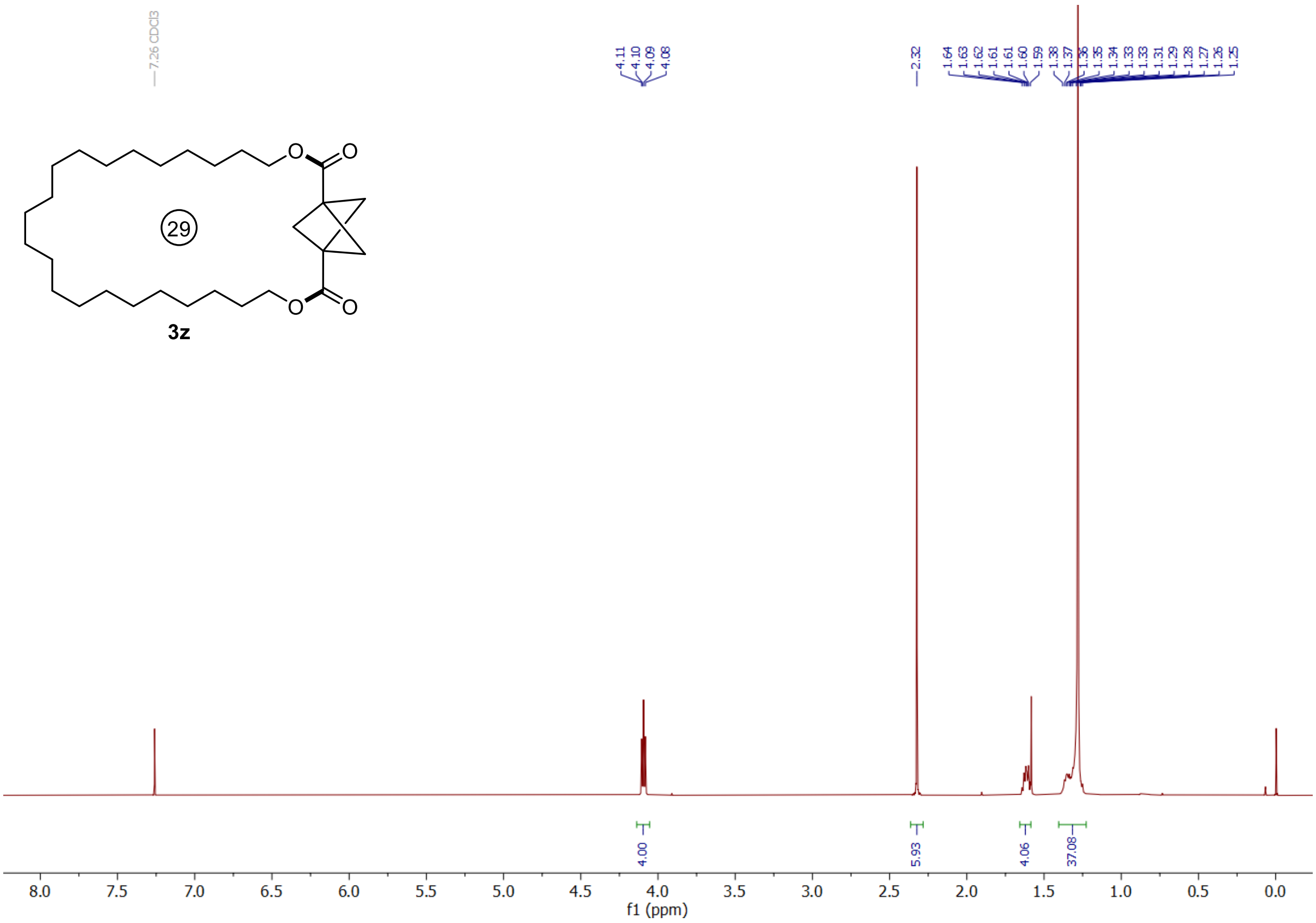

$^{13}C\{^{1}H\}$ NMR (126 MHz, $CDCl_3$, 298K) of **3z**

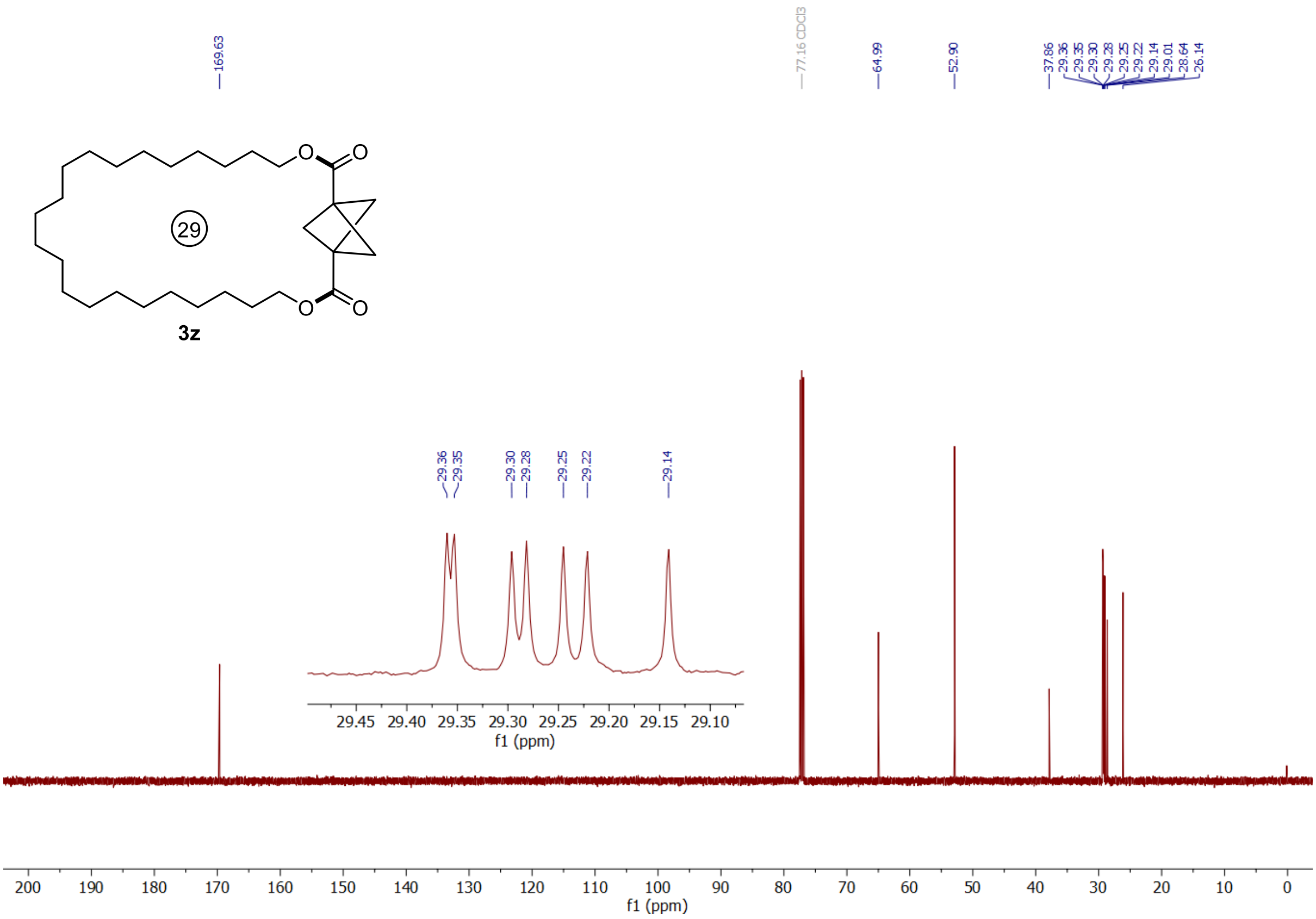

^{1}H NMR (500 MHz, $CDCl_3$, 298K) of **3aa**

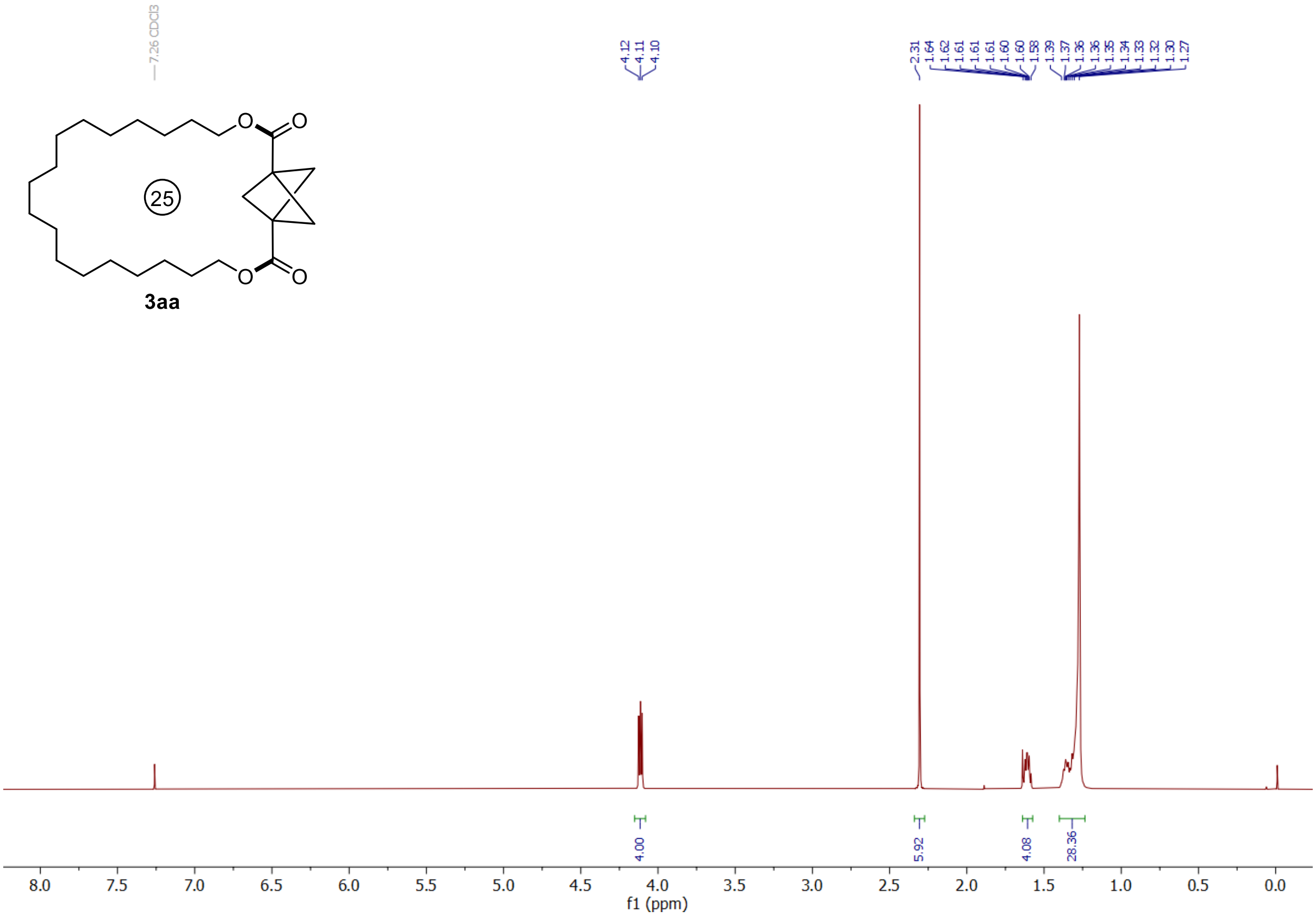

$^{13}C\{^{1}H\}$ NMR (126 MHz, $CDCl_3$, 298K) of **3aa**

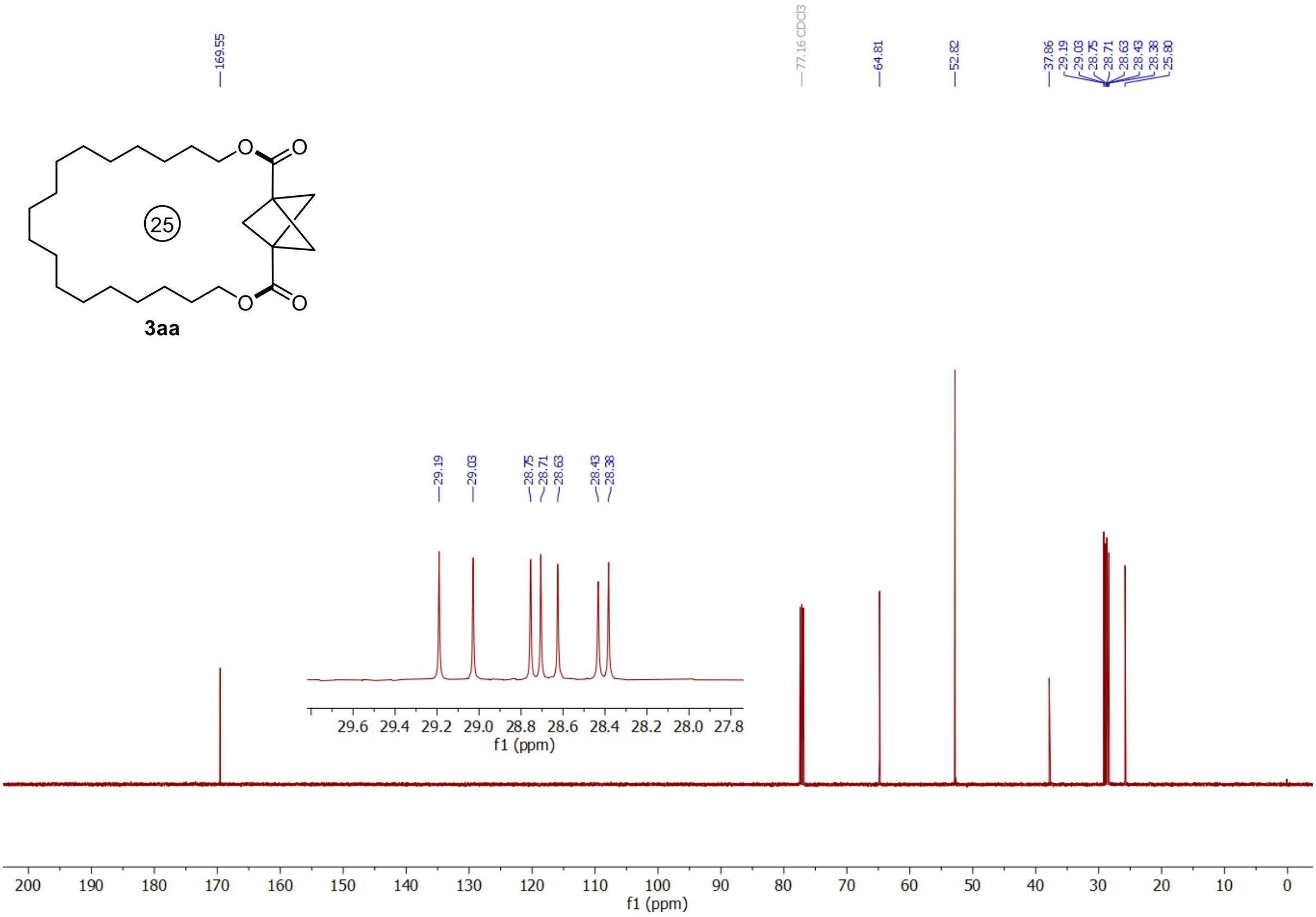

^{1}H NMR (500 MHz, $CDCl_3$, 298K) of **3ab**

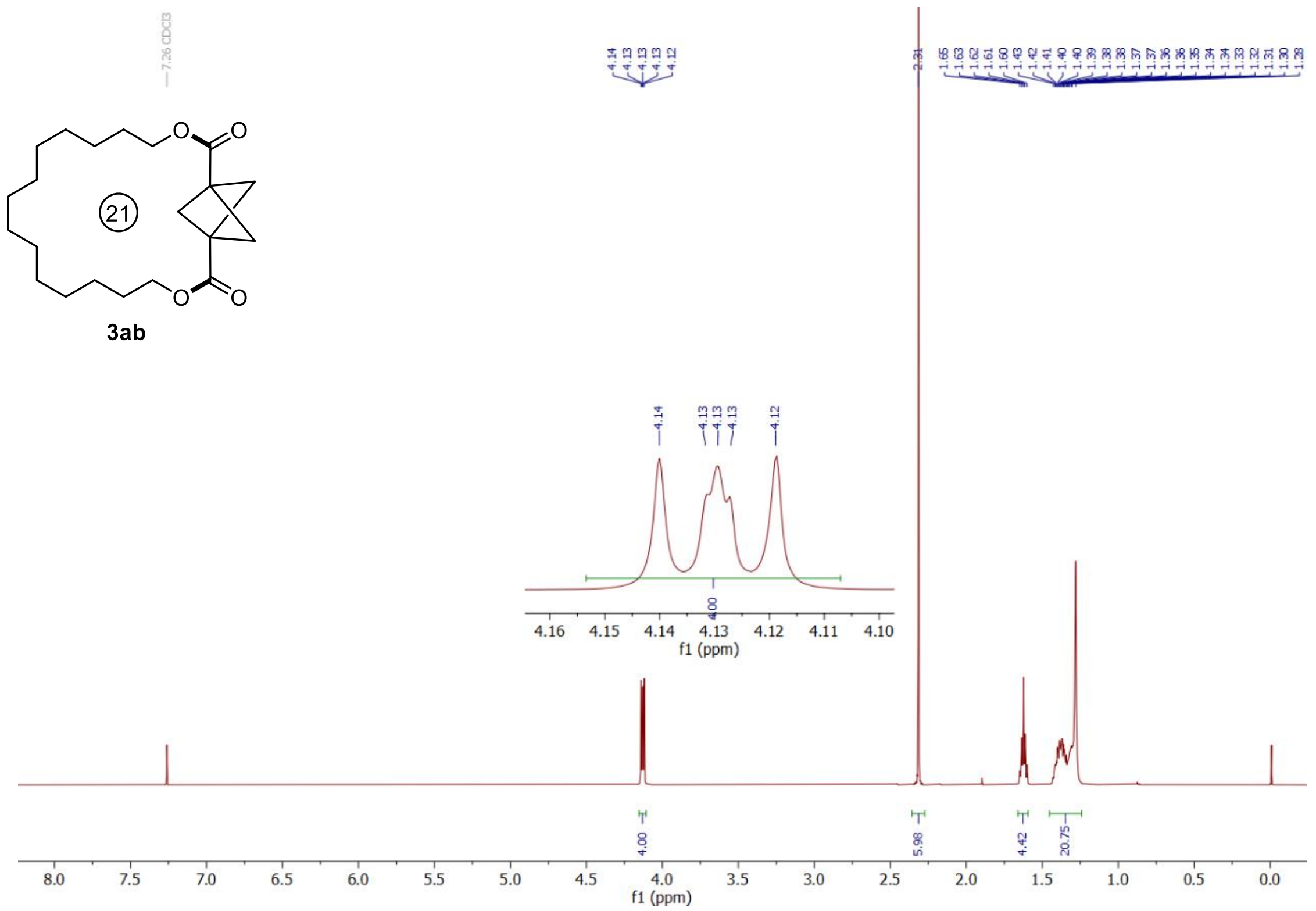

$^{13}C\{^{1}H\}$ NMR (126 MHz, $CDCl_3$, 298K) of **3ab**

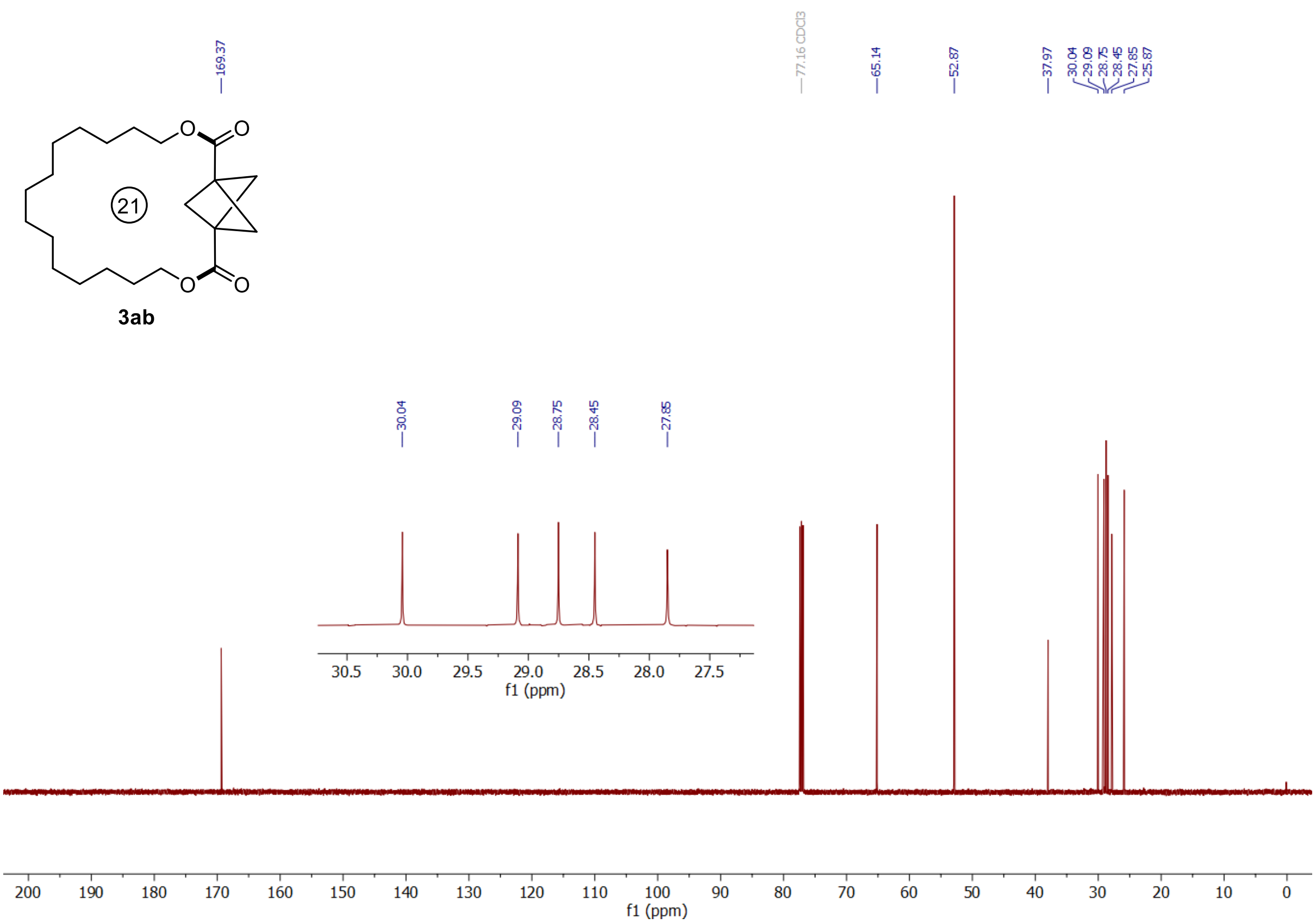

^{1}H NMR (500 MHz, $CDCl_3$, 298K) of **3ac**

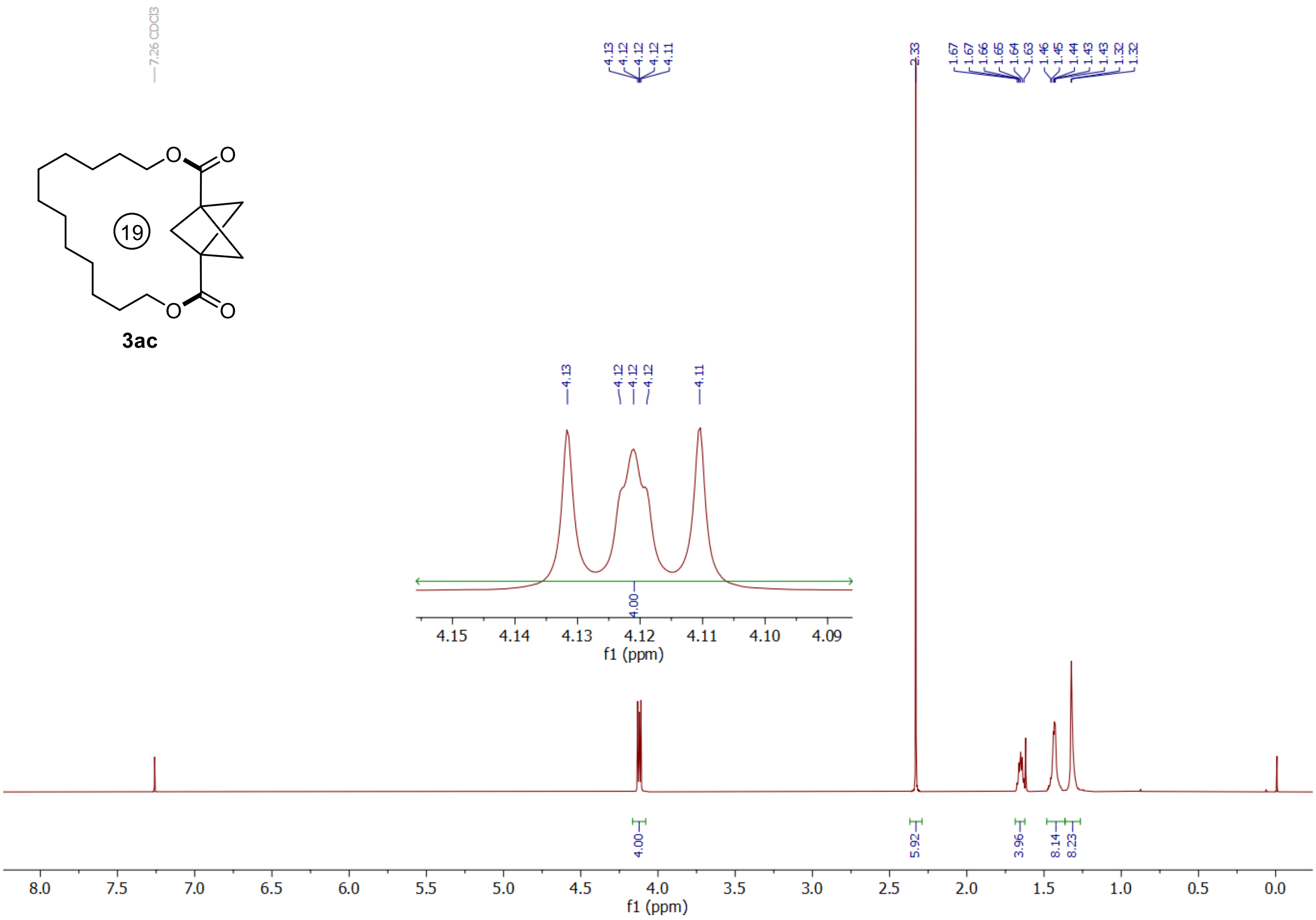

$^{13}C\{^{1}H\}$ NMR (126 MHz, $CDCl_3$, 298K) of **3ac**

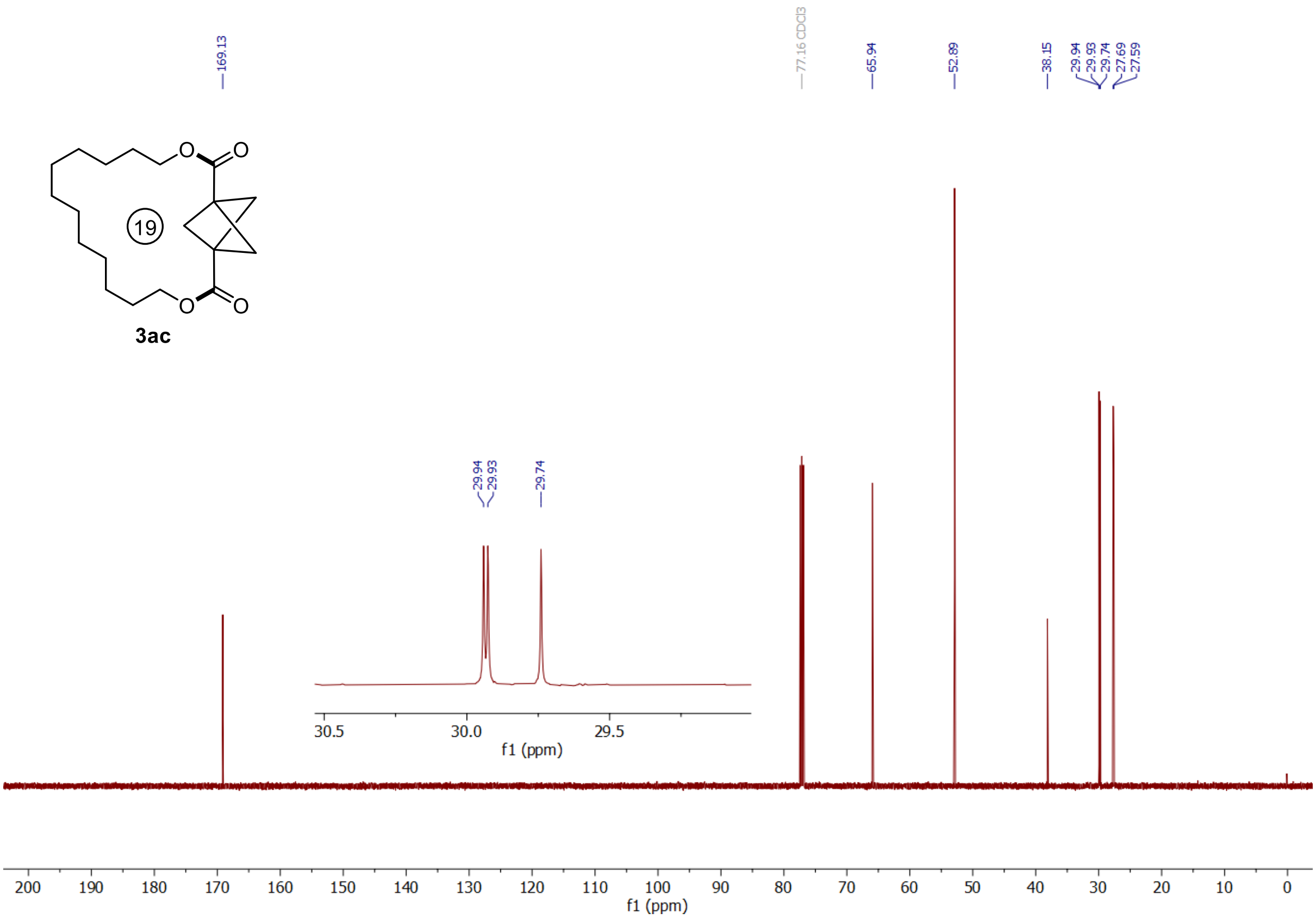

^{1}H NMR (500 MHz, $CDCl_3$, 298K) of **3ad**

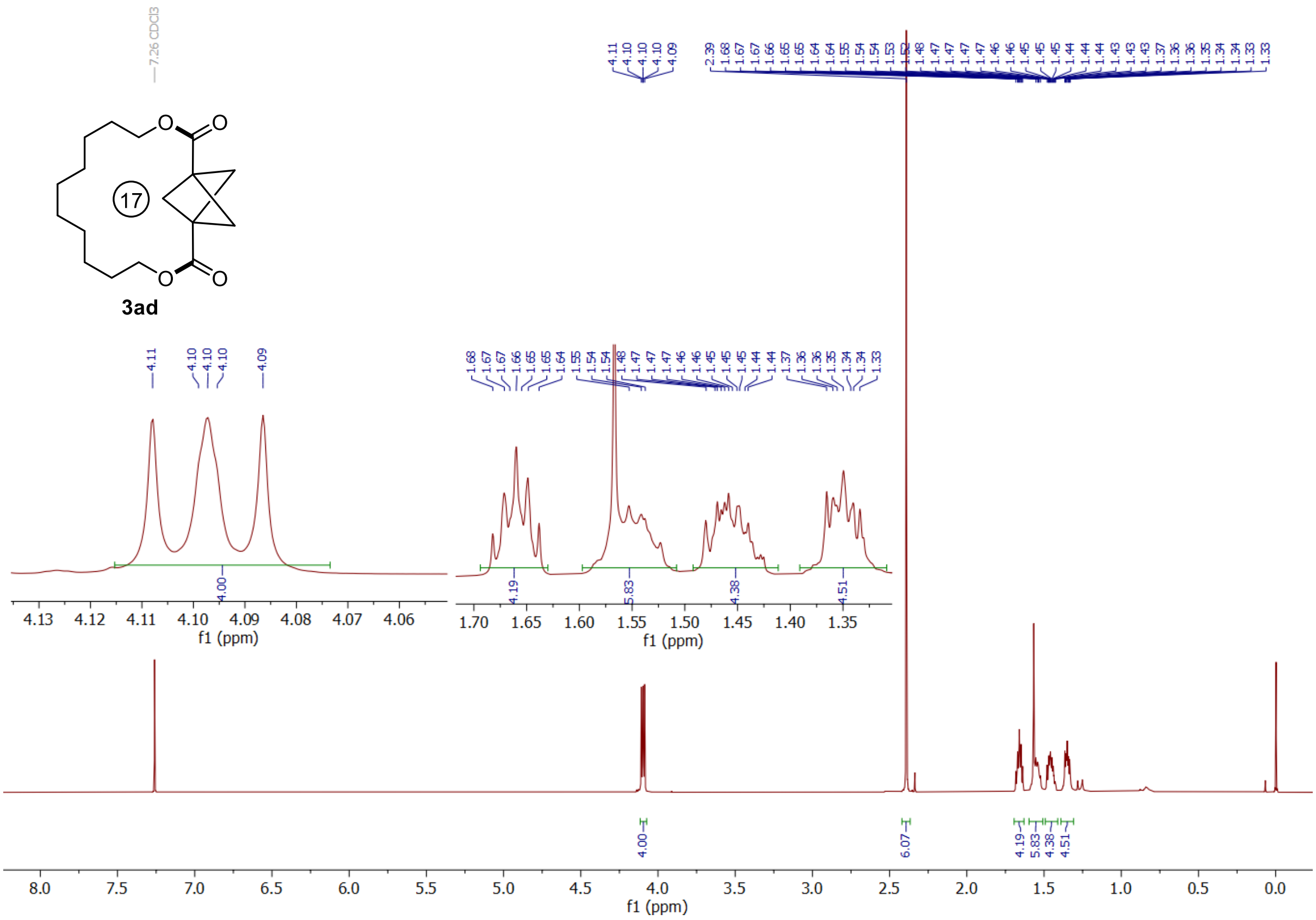

$^{13}C\{^{1}H\}$ NMR (126 MHz, $CDCl_3$, 298K) of **3ad**

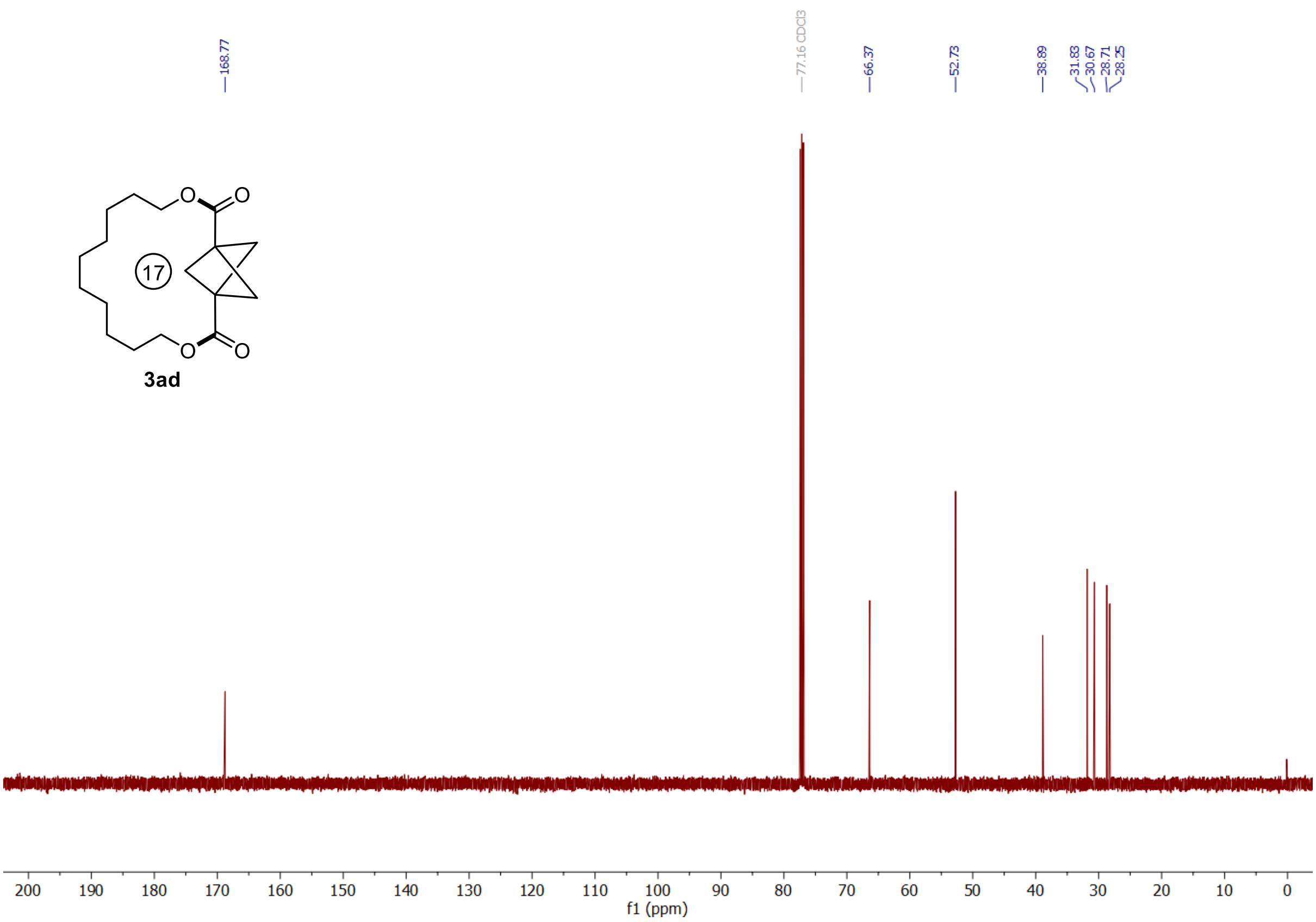

^{1}H NMR (500 MHz, $CDCl_3$, 298K) of **3ae**

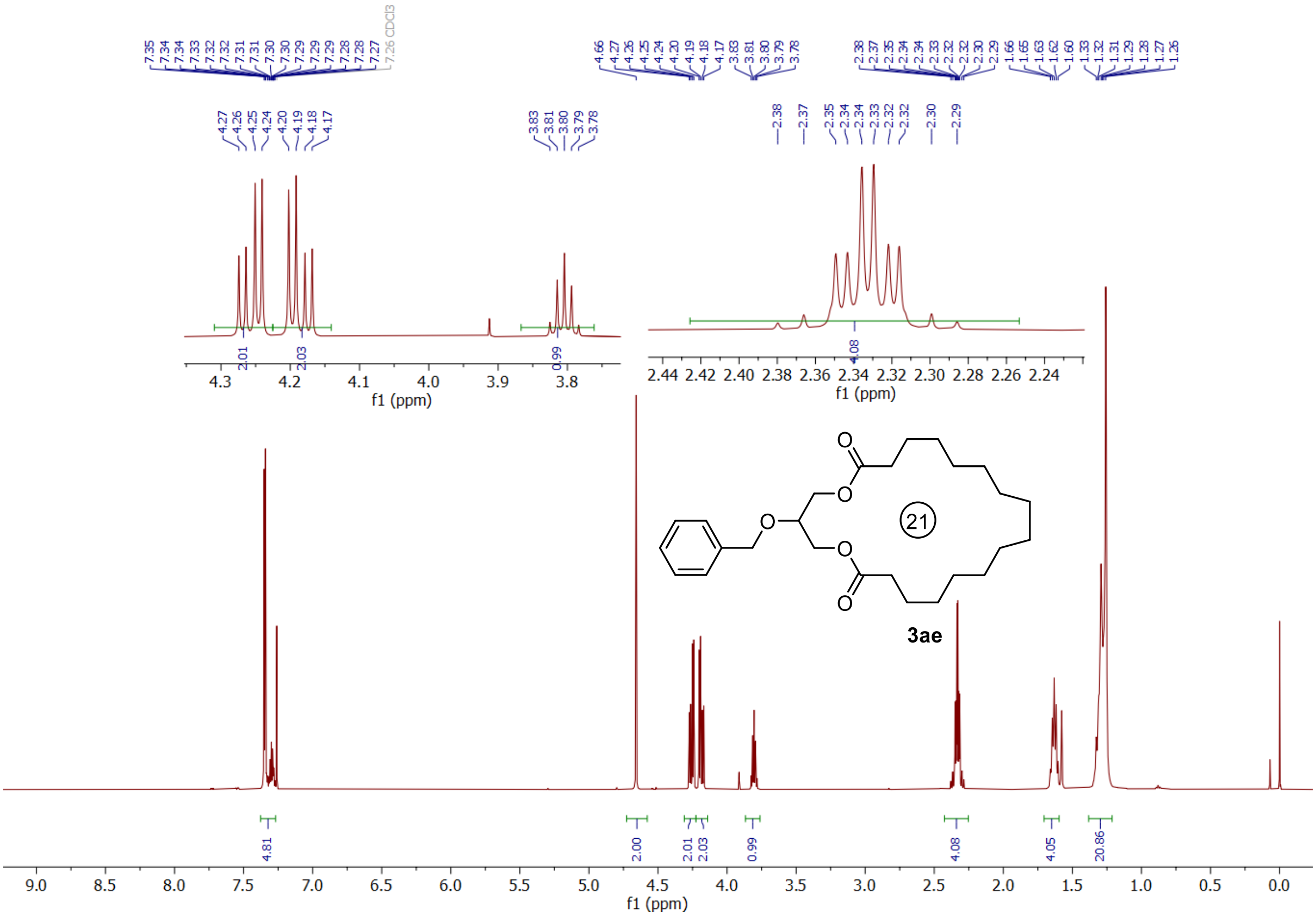

^{13}C{^{1}H} NMR (126 MHz, $CDCl_3$, 298K) of **3ae**

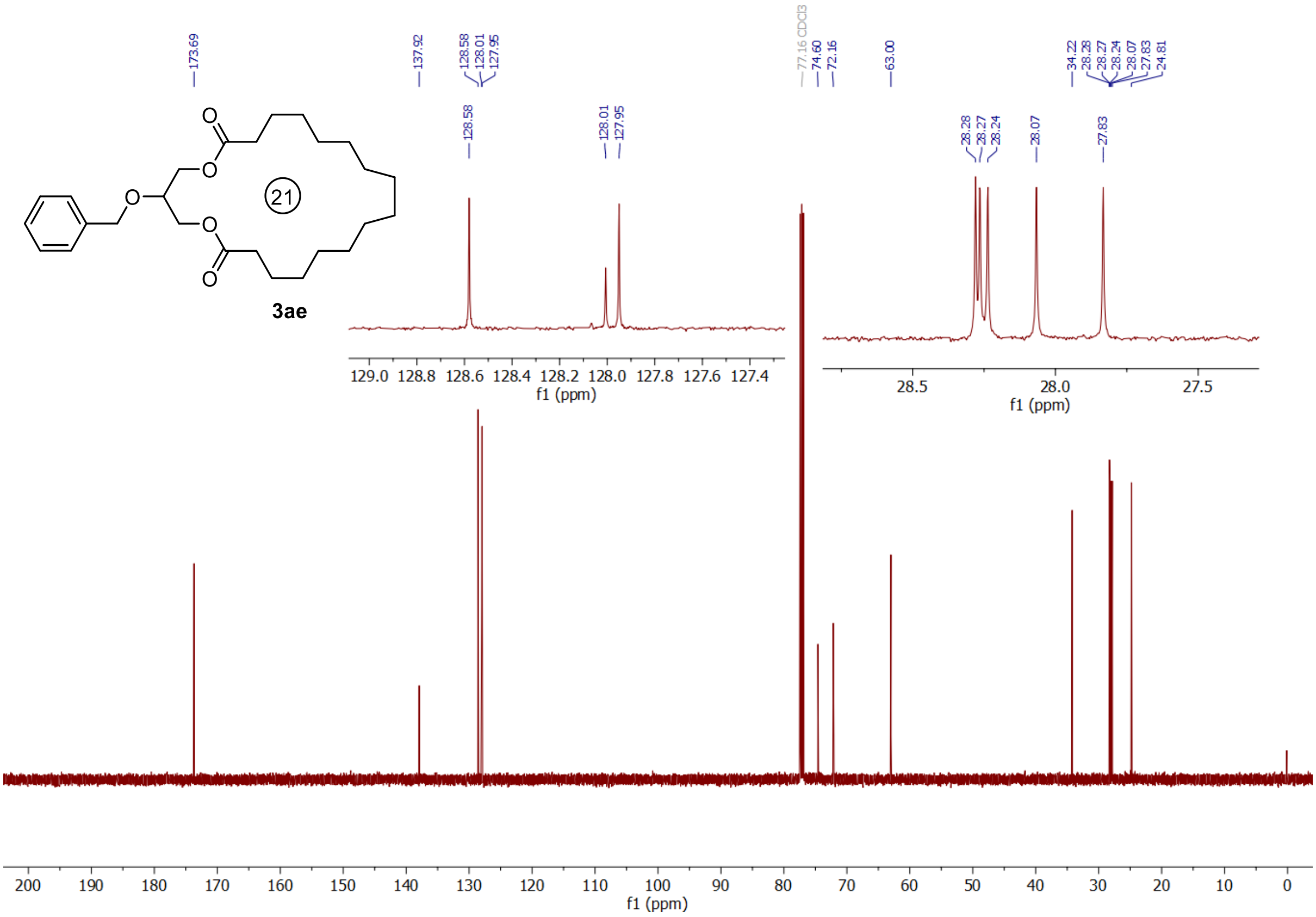

^{1}H NMR (500 MHz, $CDCl_3$, 298K) of **3af**

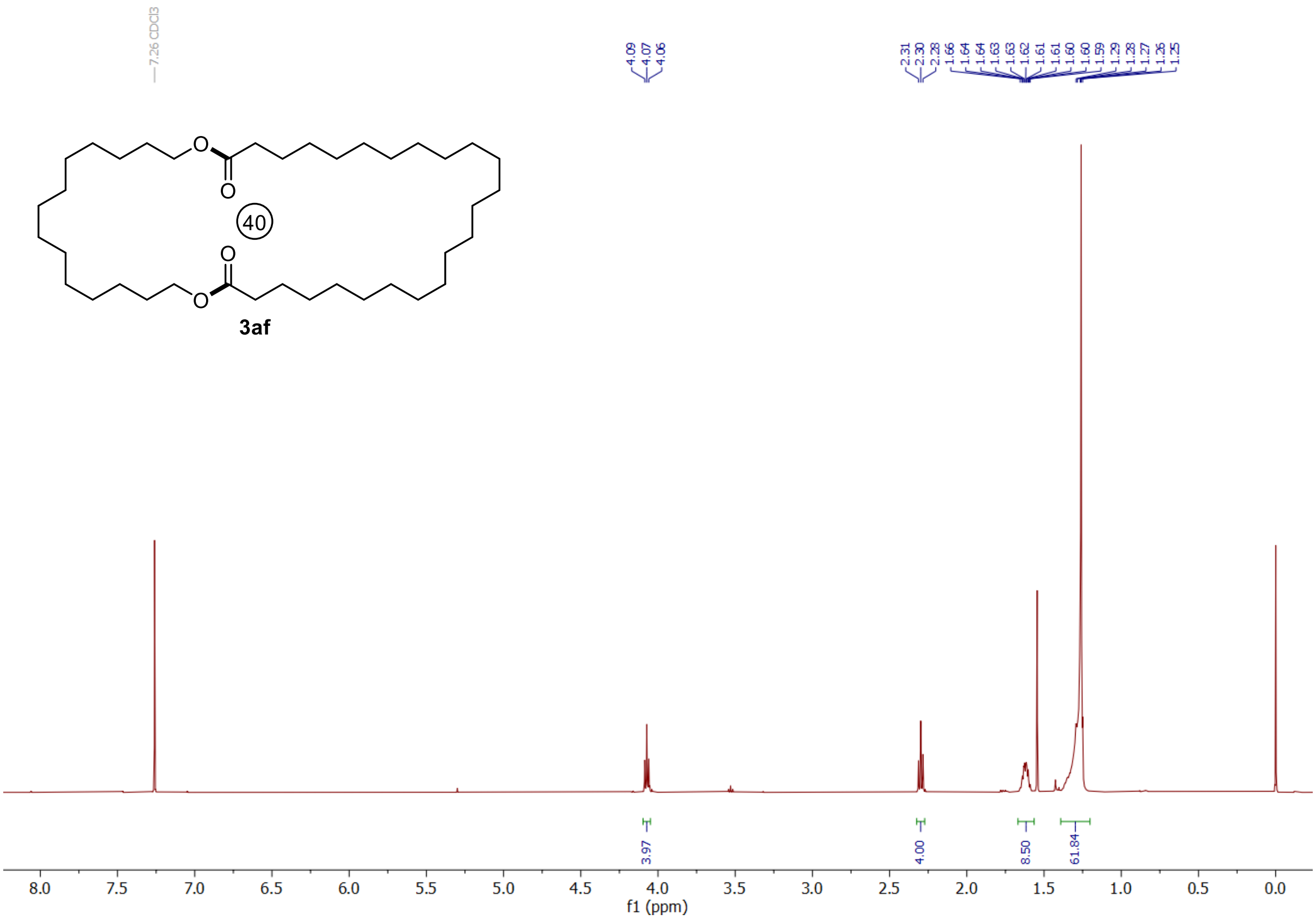

$^{13}C\{^{1}H\}$ NMR (126 MHz, $CDCl_3$, 298K) of **3af**

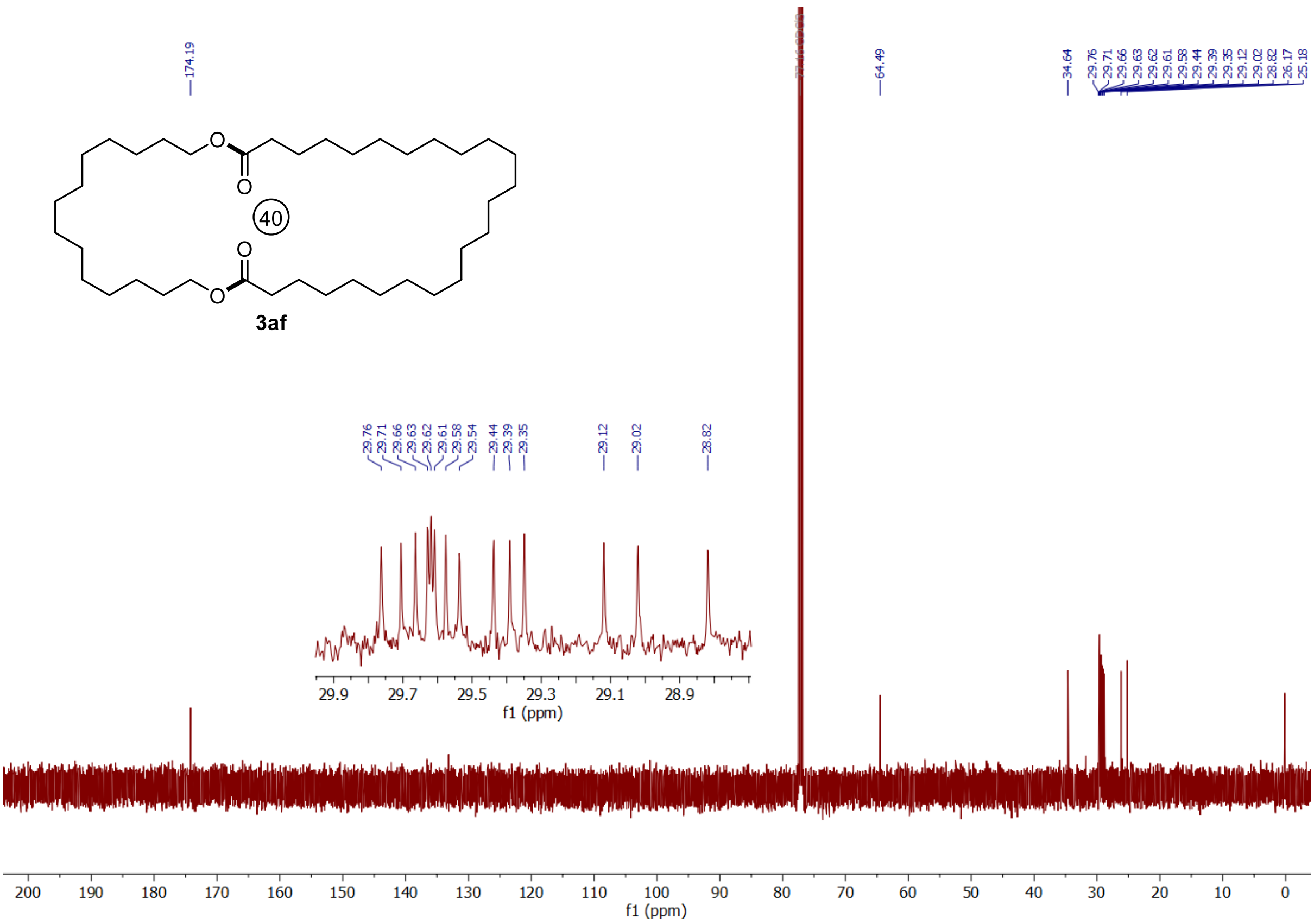

^{1}H NMR (500 MHz, $CDCl_3$, 298K) of **4a**

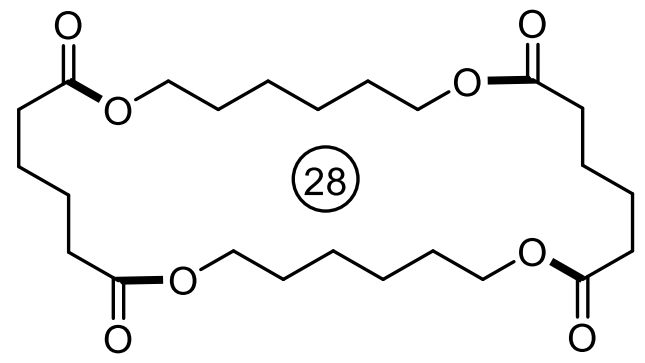

4a

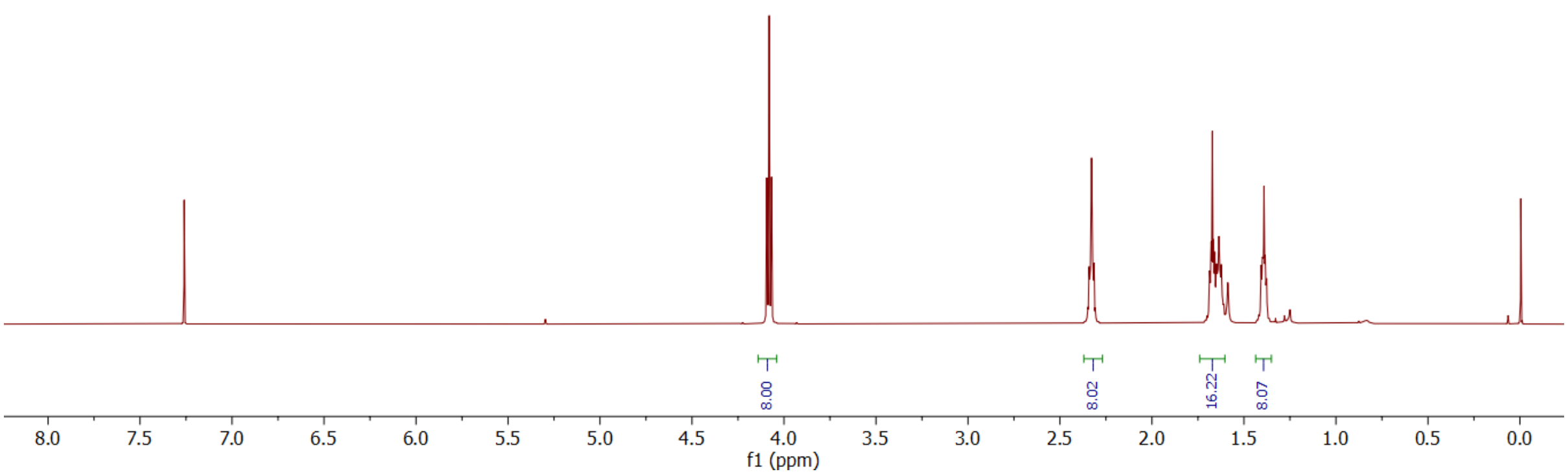

$^{13}C\{^{1}H\}$ NMR (126 MHz, $CDCl_3$, 298K) of **4a**

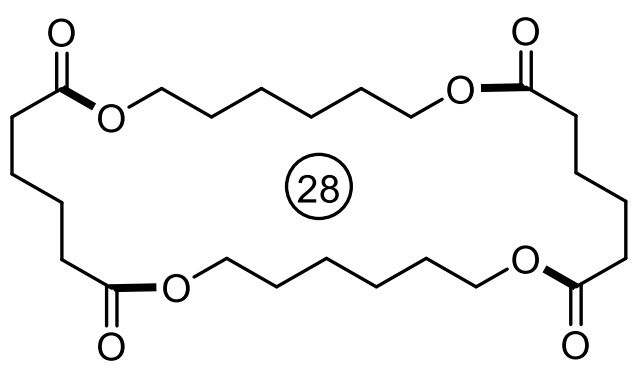

4a

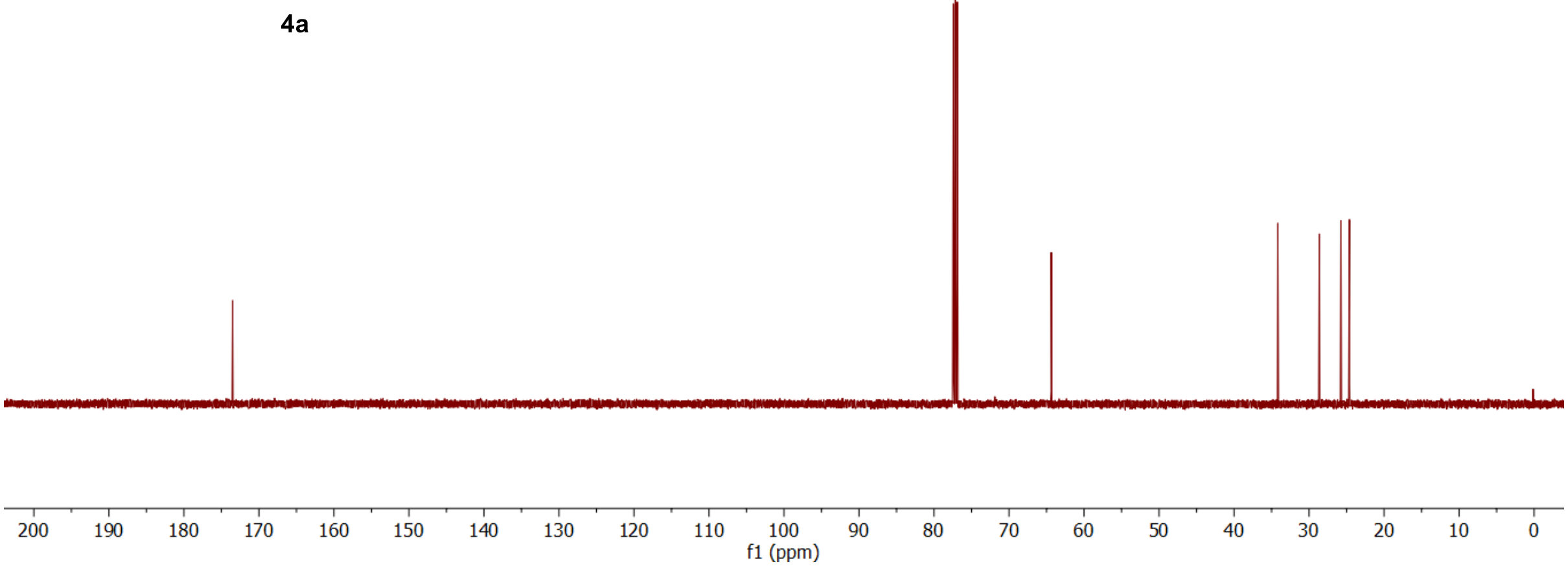

^{1}H NMR (500 MHz, $CDCl_3$, 298K) of **4b**

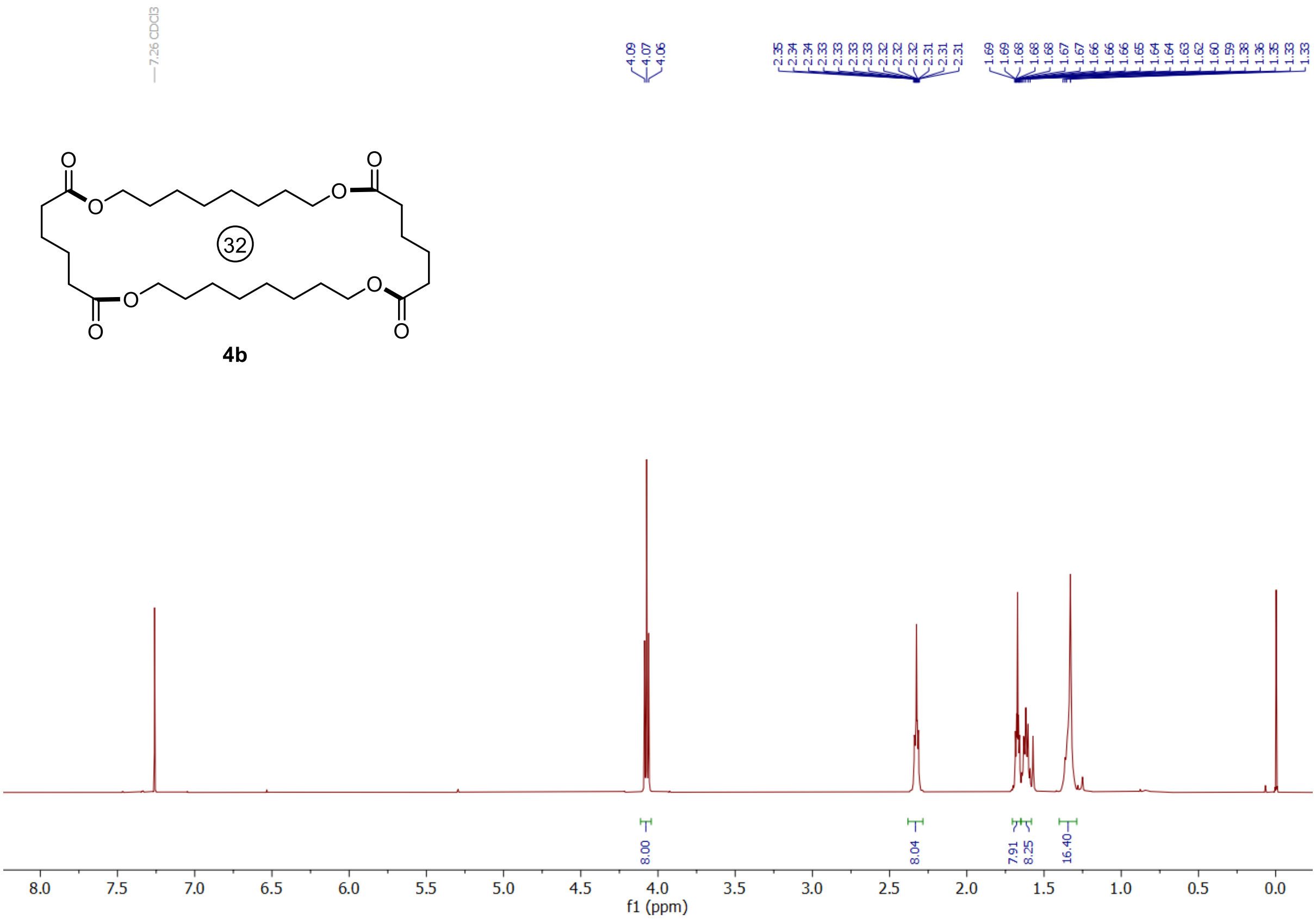

^{13}C{^{1}H} NMR (126 MHz, $CDCl_3$, 298K) of **4b**

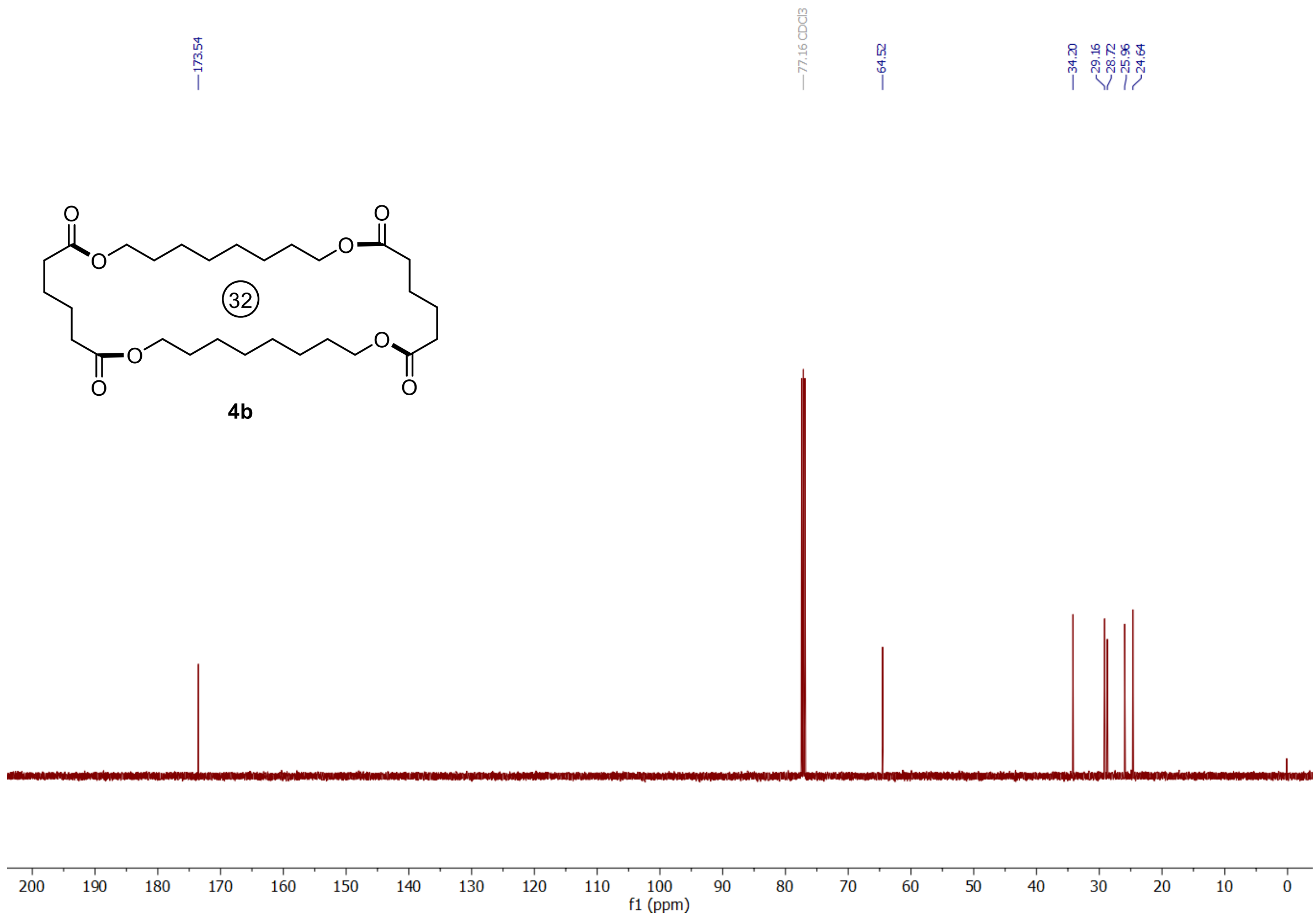

^{1}H NMR (500 MHz, $CDCl_3$, 298K) of **4c**

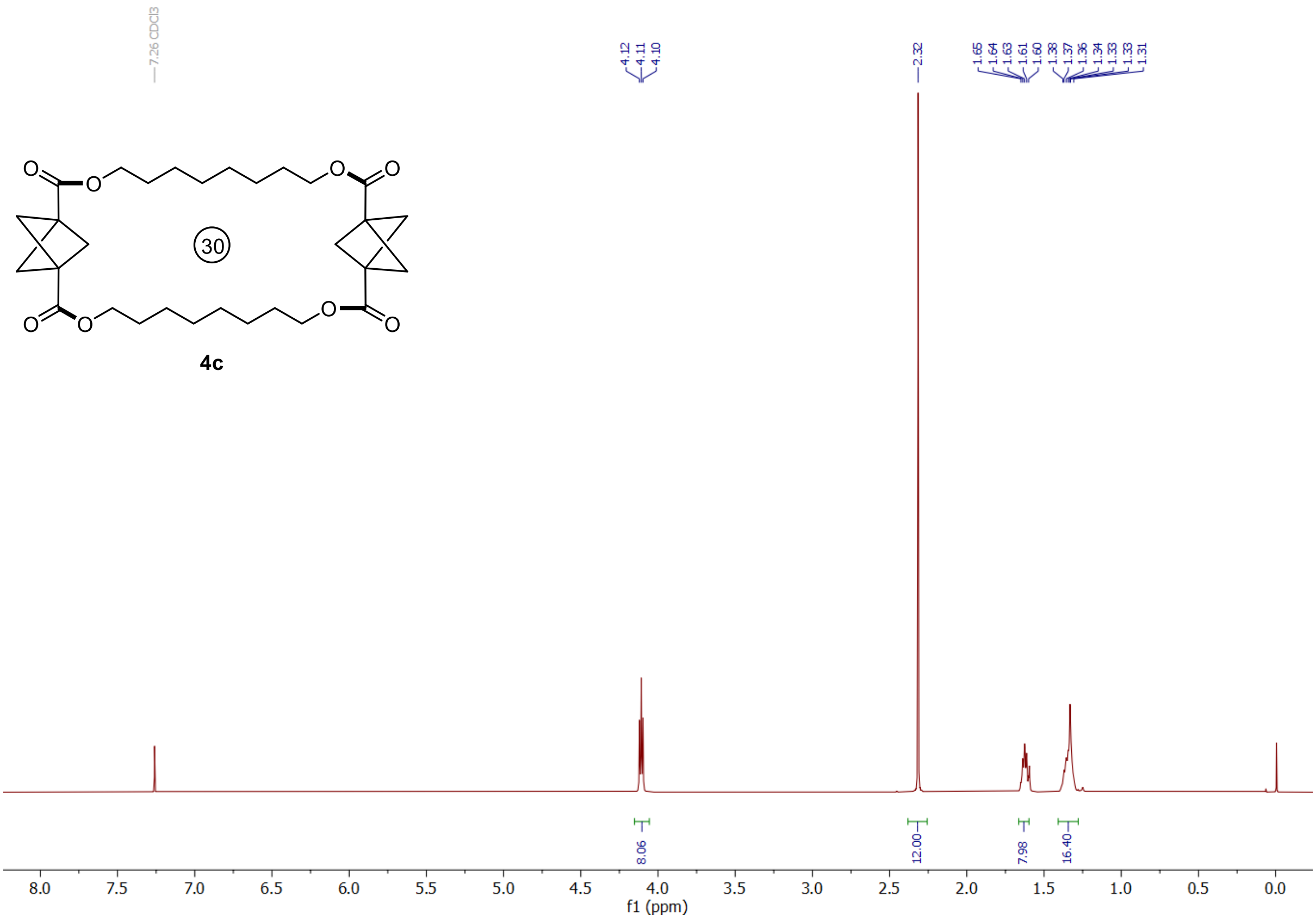

$^{13}C\{^1H\}$ NMR (126 MHz, $CDCl_3$, 298K) of **4c**

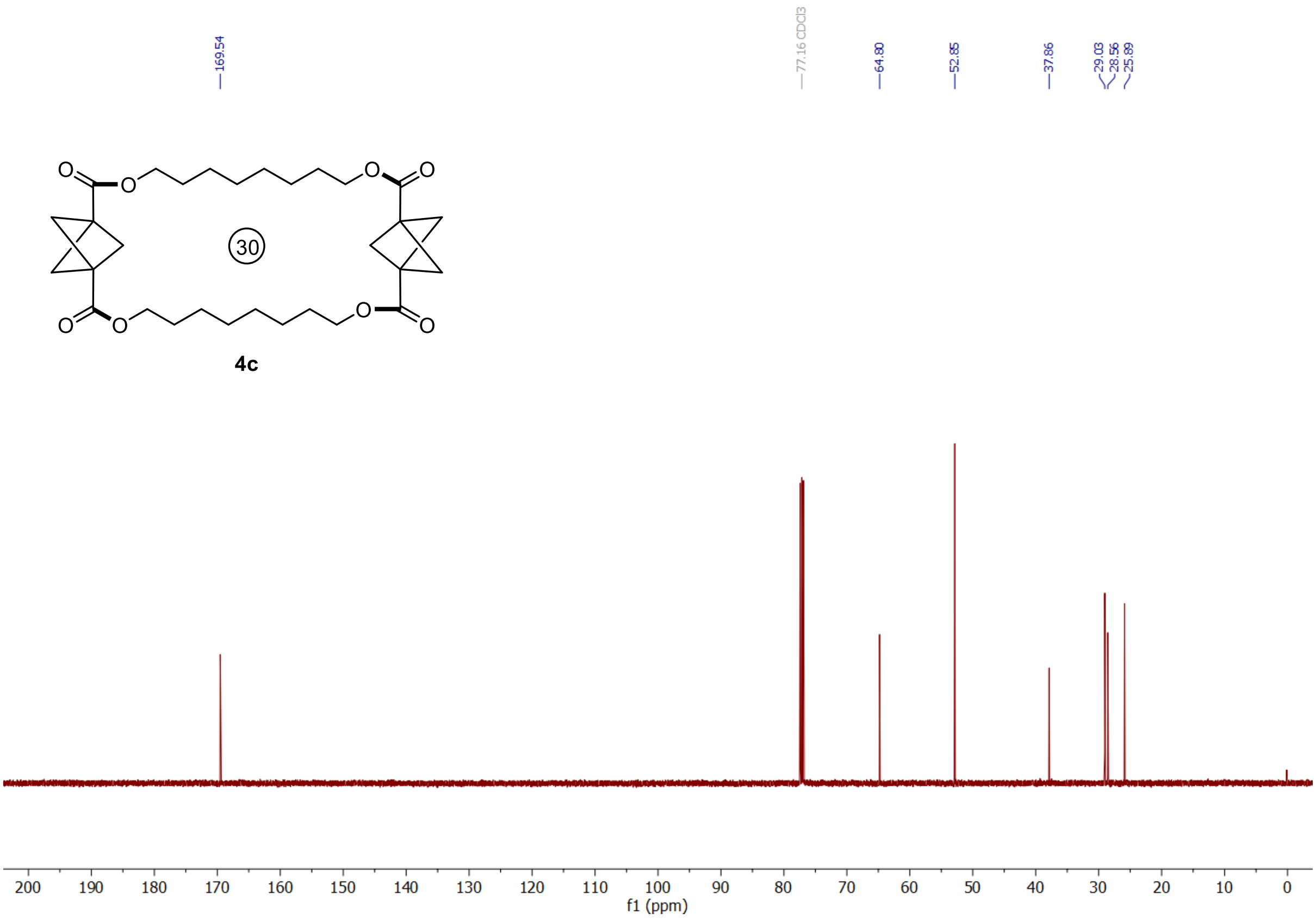

^{1}H NMR (500 MHz, $CDCl_3$, 298K) of **4d**

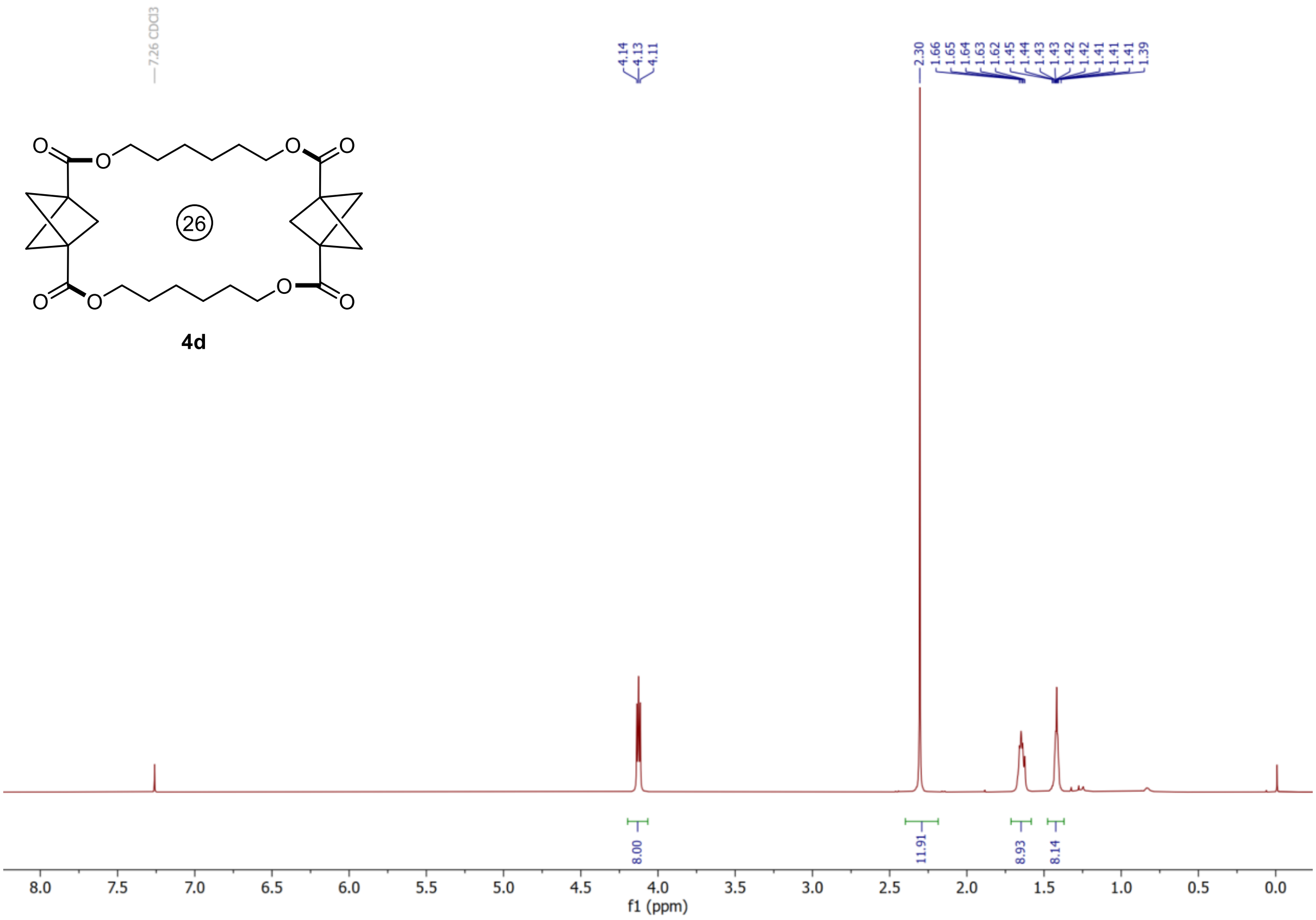

$^{13}C\{^{1}H\}$ NMR (126 MHz, $CDCl_3$, 298K) of **4d**

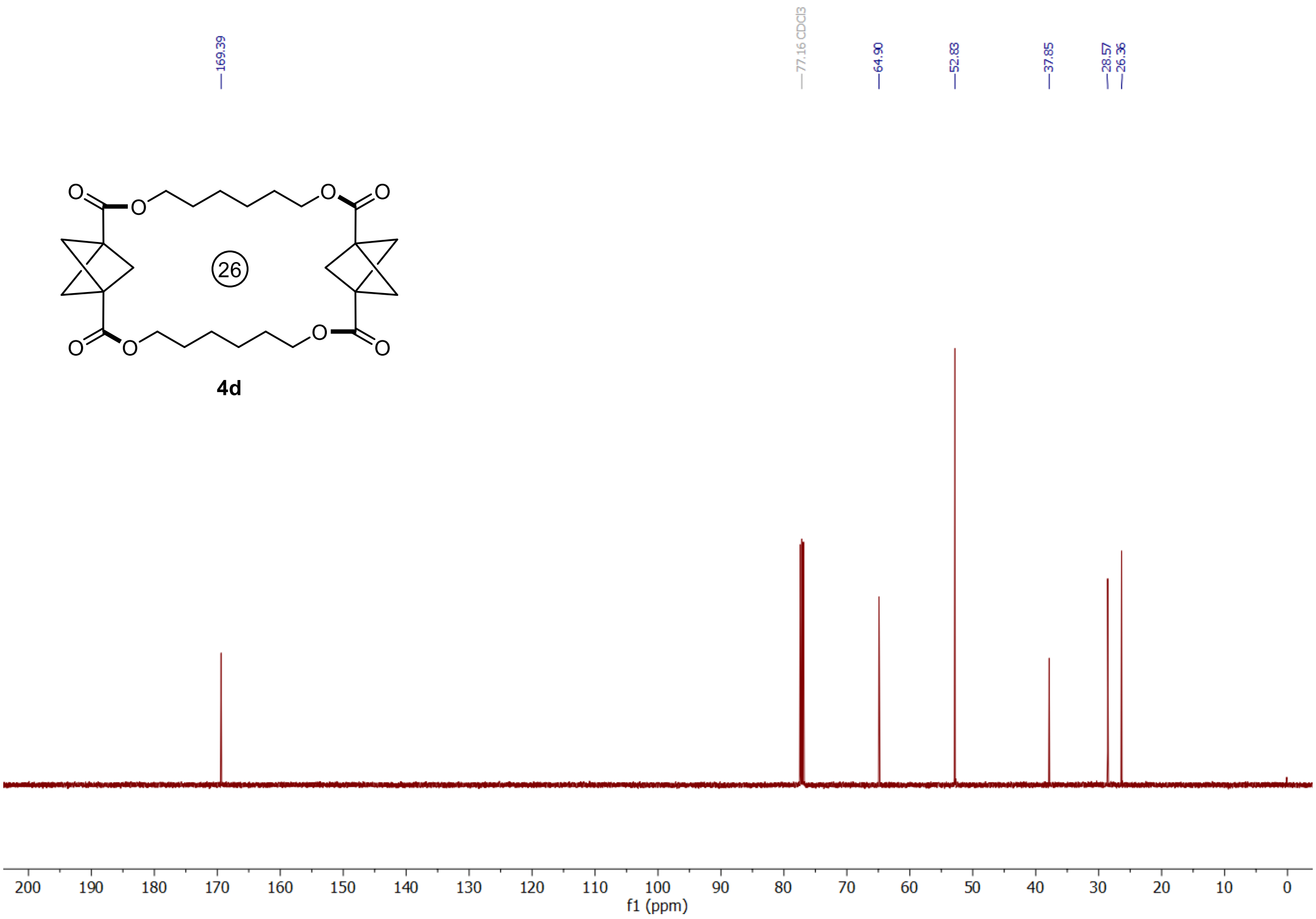

^{1}H NMR (500 MHz, $CDCl_3$, 298K) of **4e**

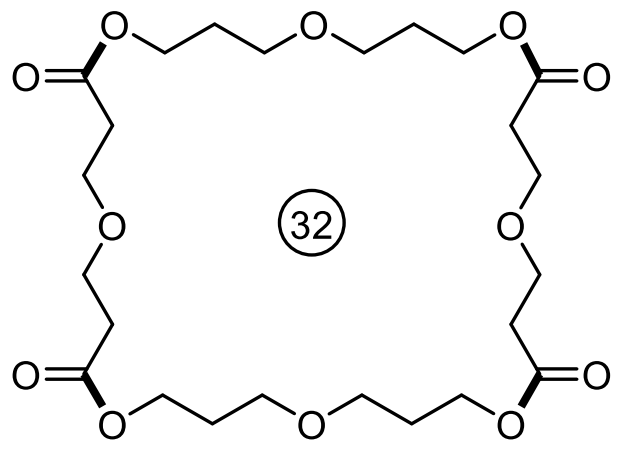

4e

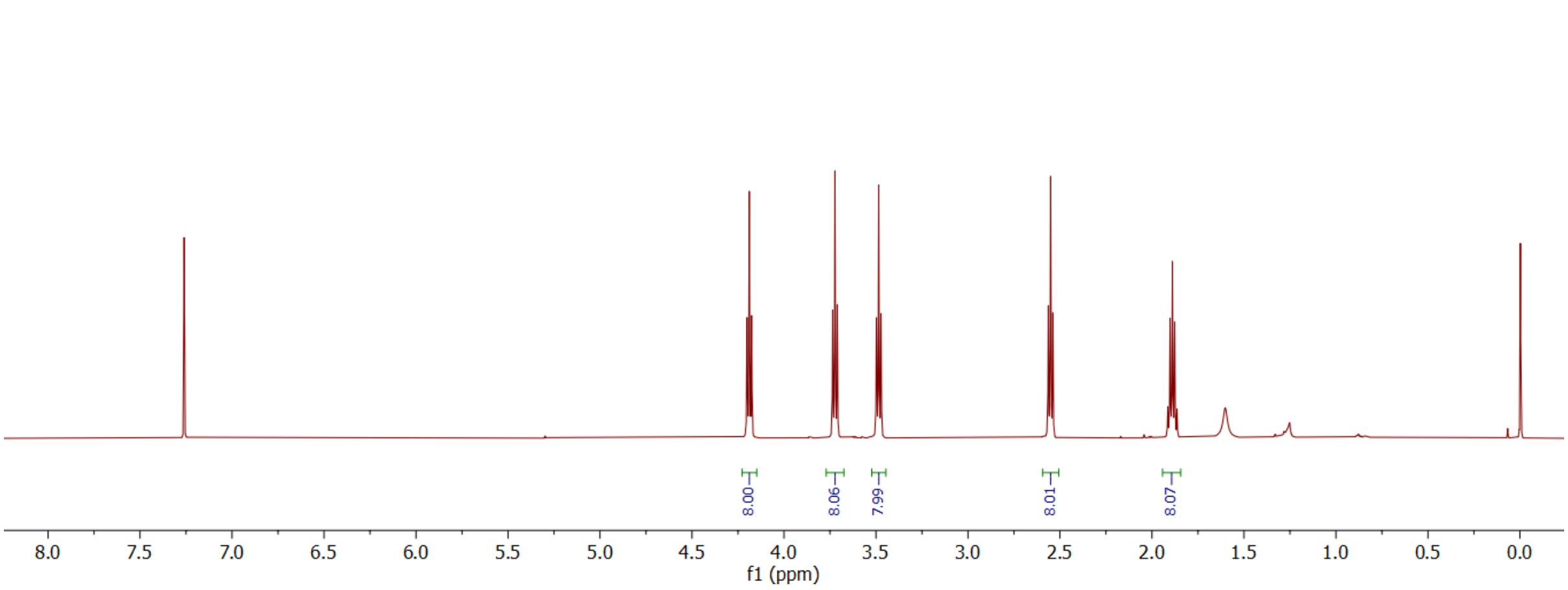

^{13}C{^{1}H} NMR (126 MHz, $CDCl_3$, 298K) of **4e**

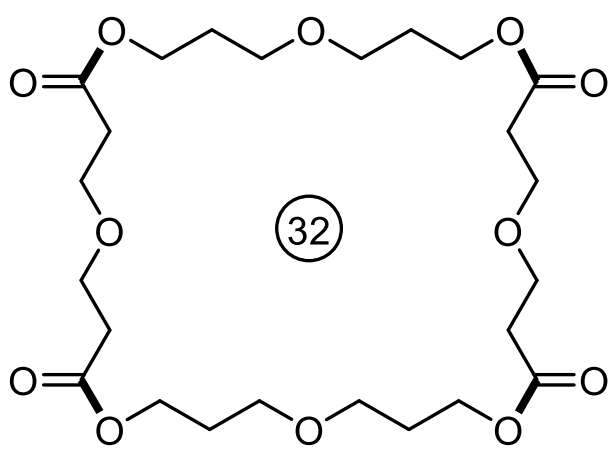

4e

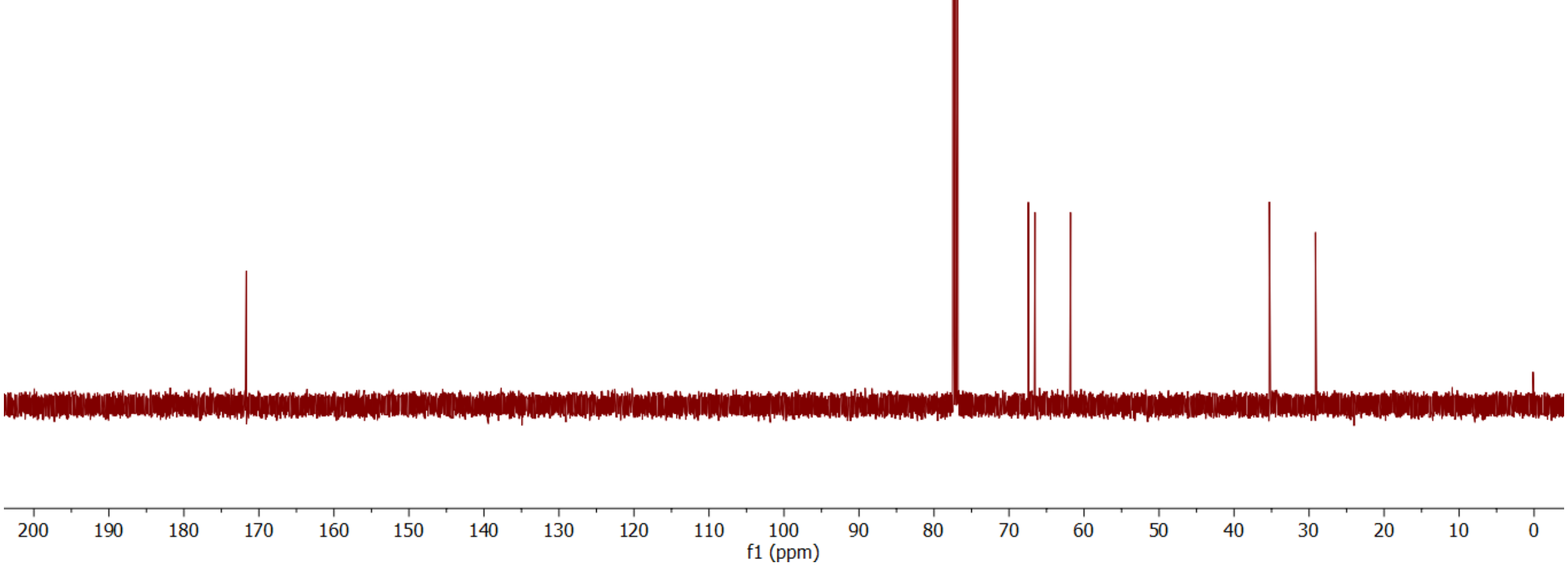

^{1}H NMR (500 MHz, $CDCl_3$, 298K) of **5**

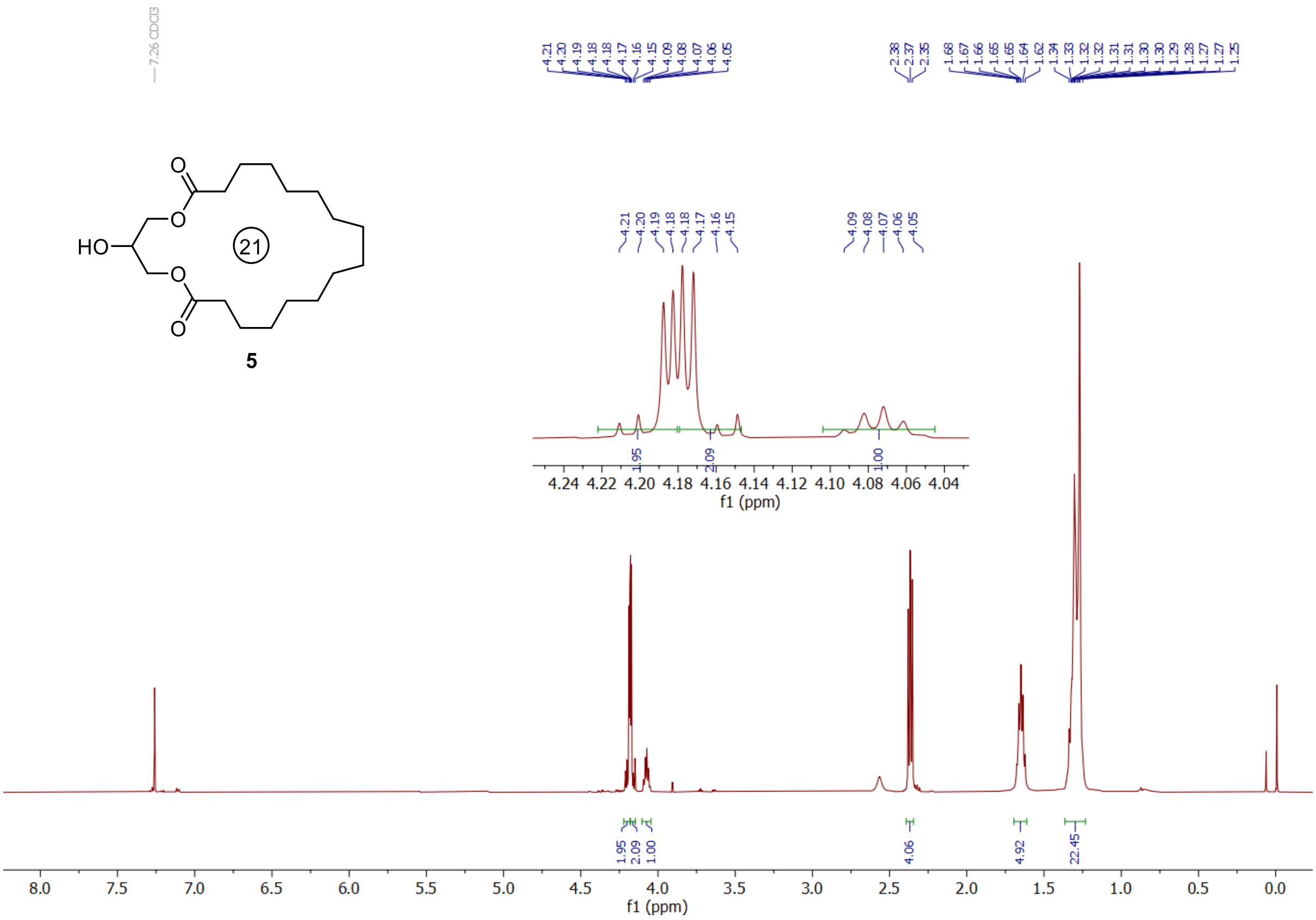

$^{13}C\{^{1}H\}$ NMR (126 MHz, $CDCl_3$, 298K) of **5**

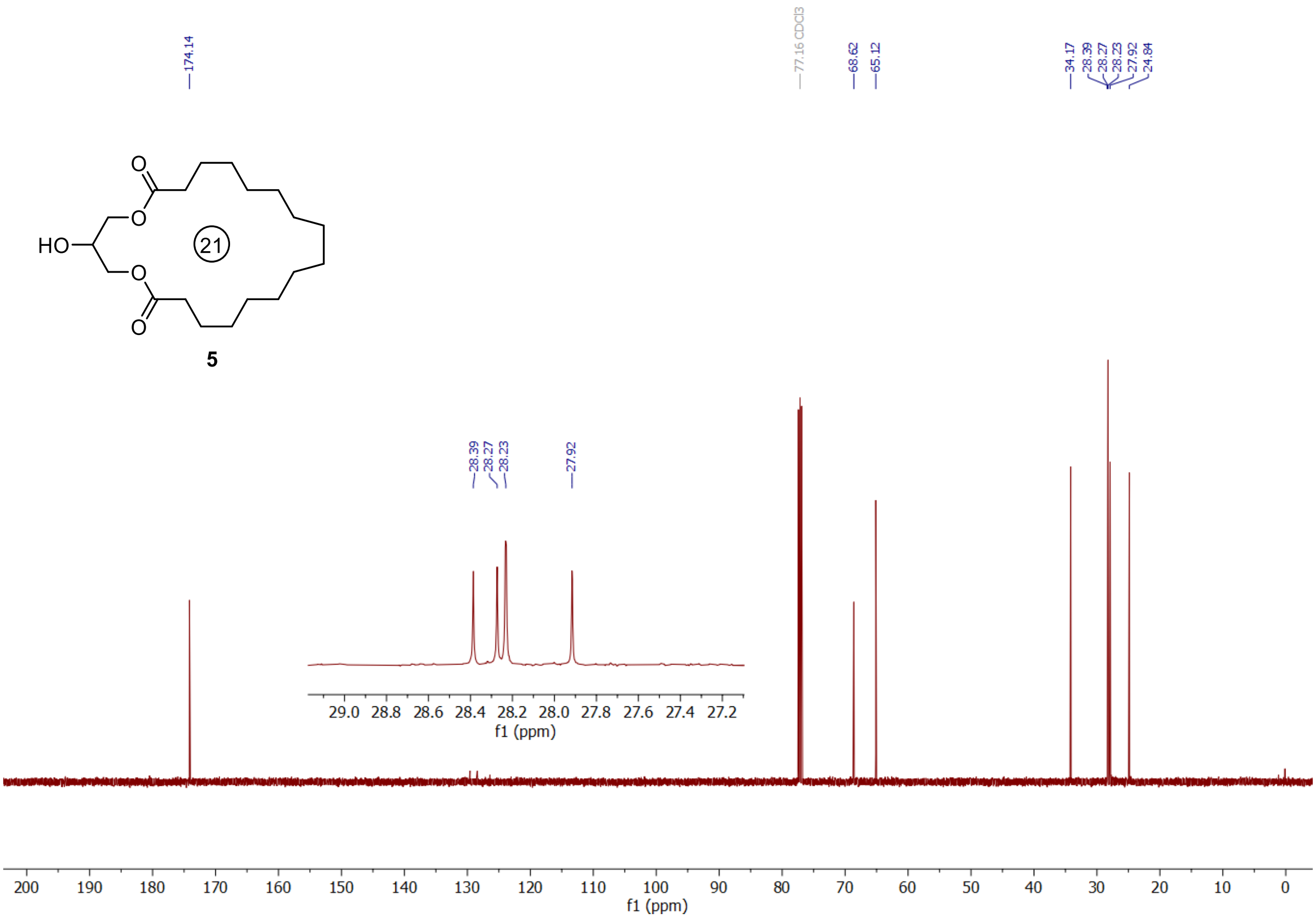

Supplement: Supplementary file 1 [file ANIE-65-e6206059-s001.pdf]
